# Unified synthesis of multiply arylated alkanes by catalytic deoxygenative transformation of diarylketones†

**DOI:** 10.1039/d2sc03720c

**Published:** 2022-08-22

**Authors:** Miki B. Kurosawa, Kenta Kato, Kei Muto, Junichiro Yamaguchi

**Affiliations:** Department of Applied Chemistry, Waseda University 513 Wasedatsurumakicho Shinjuku Tokyo 162-0041 Japan junyamaguchi@waseda.jp; Waseda Institute for Advanced Study, Waseda University 513 Wasedatsurumakicho Shinjuku Tokyo 162-0041 Japan

## Abstract

A deoxygenative transformation of diarylketones leading to multiply arylated alkanes was developed. Diarylketones were reacted with diphenylphosphine oxide resulting in a phospha-Brook rearrangement, followed by palladium-catalyzed cross-couplings or a Friedel–Crafts type alkylation to afford the corresponding multiply arylated alkanes. A variety of diarylketones can be converted to multiply arylated alkanes such as diarylmethanes, tetraarylethanes, and triarylmethanes by reduction, dimerization, and arylation in one pot. Furthermore, a one-pot conversion from arylcarboxylic acids to diarylmethanes and tetraarylethanes, and a synthesis of tetraarylmethane and triphenylethane using sequential coupling reactions are also presented.

## Abstract 2

We succeeded in synthesizing five different types of multiply arylated alkanes from diarylketones in a single step. The key for this method is the generation of diarylphosphinates via a phospha-Brook rearrangement of ketones with phosphine oxide.

## Graphical abstract

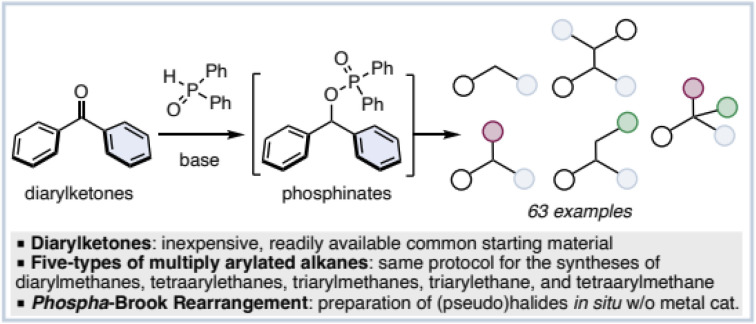

## Introduction

**Fig. 1:**
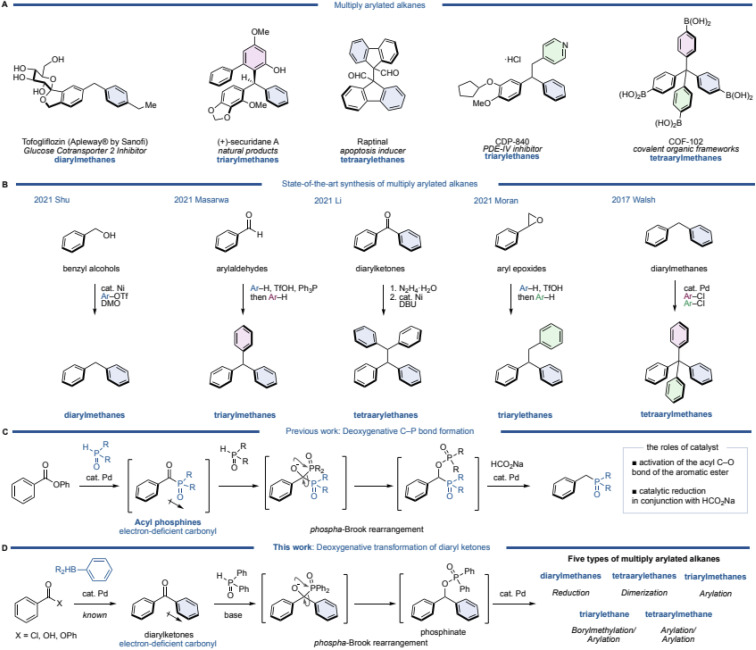
(A) Multiply arylated alkanes in pharmaceuticals, natural products, and organic materials. (B) State-of-the-art synthesis of multiply arylated alkanes. (C) Deoxygenative C–P bond formation of aromatic esters. (D) Deoxygenative transformation of diarylketones.

Multiply arylated alkanes are widely used as pharmaceuticals, natural products, and organic materials (Fig. 1A). A diarylmethane such as tofogliflozin is a representative motif among multiply arylated alkanes.^1^ A triarylmethane such as (+)-securidane A and a tetraarylethane such as Raptinal are also known bioactive products.^2–4^ CDP-840, a phosphodiesterase (PDE)-IV inhibitor,^5^ is classified as a triarylethane, and COF-102, known as a covalent organic framework, has a tetraarylmethane skeleton.^6,7^ Since these are important structures, the synthesis of multiply arylated alkanes has long been investigated.^8–21^ Particularly recently, excellent catalytic and efficient syntheses of these alkanes have been reported using readily available building blocks and state-of-the-art methodologies (Fig. 1B). For example, diarylmethanes are generally synthesized from benzyl electrophiles, but recently, a direct coupling from benzyl alcohol, which is a readily available starting material, has been reported. Shu and coworkers discovered a nickel-catalyzed direct cross-coupling of benzylic alcohols with aryl triflates in the presence of DMO (dimethyl oxalate) to afford the corresponding diarylmethanes.^22^ Masarwa and coworkers reported a unique synthetic method of triarylmethanes from aldehydes:^23^ an aldehyde was mixed with an arene and triphenylphosphine in the presence of TfOH to generate a diarylphosphonium salt, followed by another arene in one pot to furnish the corresponding triarylmethane. This simple operation allowed for a one-pot synthesis of triarylmethanes from aldehydes, but this method is limited to the use of electron-rich arenes. Li's group reported a deoxygenative dimerization from diaryl ketones, inspired by a classical Wolff–Kishner type of diarylmethane synthesis.^24^ This is an excellent method that can synthesize triarylethanes in two steps (2 pots) through the formation of hydrazone as an intermediate. Moran's group then developed a one-pot synthesis of triarylethanes from aryl epoxides.^25^ A ring-opening reaction of the epoxide by an electron-rich arene was followed by a Friedel–Crafts type alkylation of the resulting diarylethanol with another electron-rich arene *via* a phenonium intermediate. Although this method appears to be a classical synthetic method, a variety of electron-rich triarylethanes can be prepared. Walsh and coworkers demonstrated a straightforward synthesis of tetraarylmethanes from diarylmethanes.^26^ After deprotonation of the diarylmethane with a base (KO*t*-Bu), two further arenes can be introduced by palladium-catalyzed C–H arylations with aryl chlorides. A variety of tetraarylmethanes can be synthesized, although an electron-deficient heteroarene (a pyridine moiety) is required for one of the aromatic rings of the diarylmethane. While various syntheses of multiply arylated alkanes have been reported,^27,28^ thus far, there is no unified synthetic protocol from the same starting materials.

Meanwhile, recent attention has been focused on the deoxygenative transformation of aromatic carbonyls, which has been extensively studied by the Li group and others.^29–33^ Regarding this field of research, we have also developed a deoxygenative C–P bond formation of aromatic esters (Fig. 1C).^34^ In this reaction, an aromatic ester reacts with phosphine oxide in the presence of a palladium catalyst to give an acylphosphine oxide species as the intermediate. The intermediate has a highly electron-deficient carbonyl, thus a nucleophilic attack of the phosphine oxide to the carbonyl occurs, followed by a [1,2]-phospha-Brook rearrangement and reduction of the carbon–oxygen bond using a palladium catalyst and sodium formate (HCO_2_Na) as a mild reductant to afford the corresponding deoxygenative products. Based on this mechanism, we envisioned that this reaction could be utilized as a unified synthesis of multiply arylated alkanes from diarylketones (Fig. 1D).

Diarylketones are inexpensive, readily available basic chemicals that can also be generated from aryl carboxylic acids, acid chlorides, and esters in a single step by palladium-catalyzed cross-coupling.^35–38^ In general, diarylketones have an electron-deficient carbonyl, and phosphine oxide is able to attack the carbonyl, which would then undergo a [1,2]-phospha-Brook rearrangement to produce diarylphosphinates.

Since this phosphinate would be a common synthetic intermediate similar to (pseudo)halogenated diarylmethane, we hypothesized that it could be transformed to diarylmethanes by reduction, tetraarylethanes by dimerization, and triarylmethanes by arylation. Furthermore, a homologation of the intermediate by diborylmethane, followed by arylation would afford triarylethanes, and tetraarylmethanes would be synthesized by arylation of triarylmethanes. Herein, we report a deoxygenative transformation of diarylketones with diphenyl phosphine oxide by using a palladium catalyst and HCO_2_Na, leading to five different types of multiply arylated arenes in one pot.

## Results and discussion

### Discovery and screening of optimal conditions for the synthesis of diarylmethanes and tetraarylethanes by deoxygenative transformations

Following our previous work on deoxygenative C–P bond formation,^34^ benzophenone (1) and diphenylphosphine oxide (1.5 equiv.) with 5.0 mol% PdCl_2_, 10 mol% dcype (1,2-bis(dicyclohexylphosphino)ethane), and sodium formate (HCO_2_Na, 1.5 equiv.) as a hydrogen source in 1,2-dimethoxyethane (DME) at 150 °C for 12 h were used as the initial conditions. As a result, these conditions successfully gave the desired diphenylmethane (2A) in 10% yield along with the unexpected tetraphenylethane (3A) in 4% yield. After extensive optimization of the reaction conditions (see the ESI for details†), we identified the optimal conditions for 2A. To our delight, when the conditions were changed to PdCl_2_/PPh_3_ as a catalyst and Cs_2_CO_3_ (2.0 equiv.), HCO_2_Na (2.0 equiv.) in DMSO at 150 °C for 1 h, the yield of 2A significantly improved to 93% and no 3A was observed (Table 1, entry 1).

**Table 1:** Variations from standard conditions^aa^

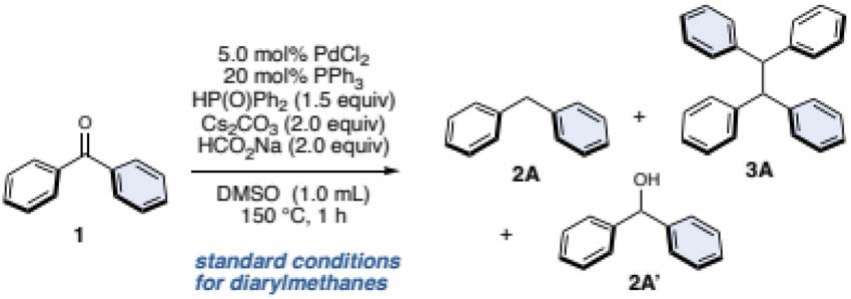					
entry	variations^b^	1/%	2A/%	3A/%	2A′/%
1	None	0	93	0	0
2	w/o PdCl_2_	35	0	0	44
3	w/o PPh_3_	14	56	0	15
4	w/o HP(O)Ph_2_	86	0	0	12
5	w/o Cs_2_CO_3_	27	31	12	28
6	w/o HCO_2_Na	64	13	4	14
7	100 °C	4	83	0	0
8^c^	DME, 12 h	0	13	76	0

a Conditions; 1 (0.20 mmol), PdCl_2_ (5.0 mol%), PPh_3_ (20 mol%), Cs_2_CO_3_ (2.0 equiv.), HCO_2_Na (1.5 equiv.), DMSO (1.0 mL), 150 °C, 1 h. Yields were determined by ^1^H NMR analysis.

b Variations from standard conditions.

c Cs_2_CO_3_ (0.40 equiv.) was used.

Next, control experiments were undertaken. The palladium salt is essential for this reaction, because without it, the reaction furnished the “half-reduced” diphenylmethanol (2A′), and the starting material 1 was also recovered (Table 1, entry 2). Without the ligand, the yield was lower (Table 1, entry 3), and diphenylphosphine oxide was critical (Table 1, entry 4). The reaction can proceed without the base, but the yield of 2A and the selectivity for 2A and 3A was significantly decreased (Table 1, entry 5). The reaction resulted in further decreasing product yields without HCO_2_Na (Table 1, entry 6). Although this reaction is typically performed at 150 °C, it can proceed at 100 °C with only a slight decrease in yield of 2A (Table 1, entry 7). We then examined various conditions to improve the yield of 3A and found that simply changing the solvent to DME, and decreasing the amount of Cs_2_CO_3_ (0.40 equiv.) significantly improves the yield of 3A. Finally, by extending the reaction time to 12 h, we succeeded in preferentially producing 3A in 76% yield (Table 1, entry 8, and see the ESI for details†).

### Substrate scope for diarylmethanes and tetraarylethanes

**Fig. 2:**
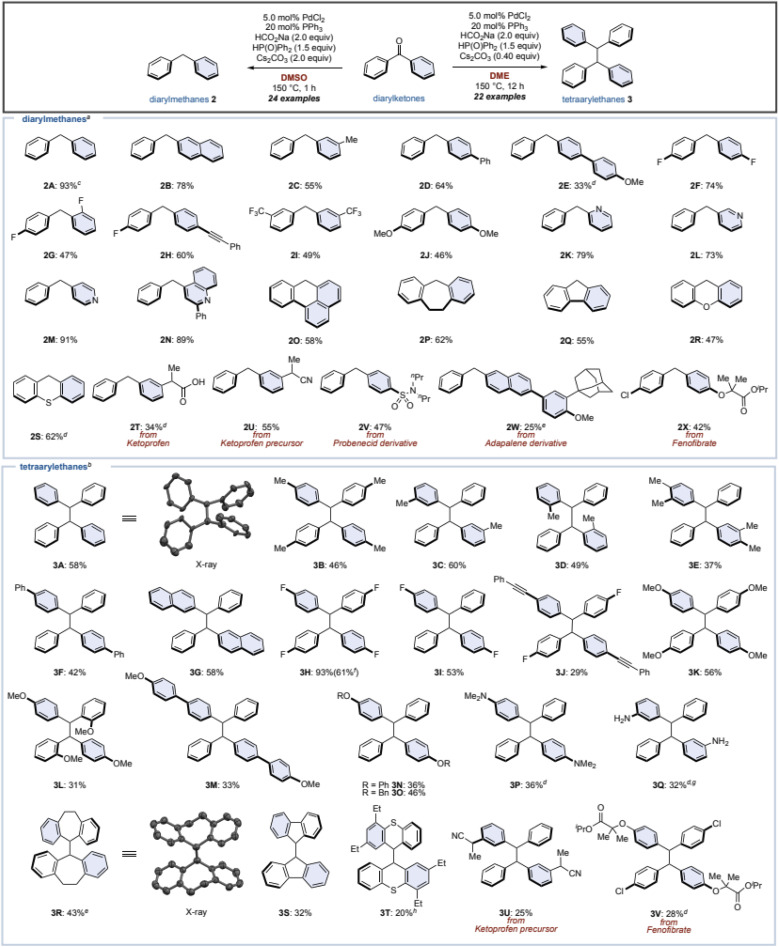
Substrate scope Conditions; ^*a*^1 (0.40 mmol), diphenylphosphine oxide (1.5 equiv.), PdCl_2_ (5.0 mol%), PPh_3_ (20 mol%), HCO_2_Na (2.0 equiv.), Cs_2_CO_3_ (2.0 equiv.), DMSO (1.0 mL), 150 °C, 1 h. ^*b*^1 (0.40 mmol), diphenylphosphine oxide (1.5 equiv.), PdCl_2_ (5.0 mol%), PPh_3_ (20 mol%), HCO_2_Na (2.0 equiv.), Cs_2_CO_3_ (0.40 equiv.), DME (1.0 mL), 150 °C, 12 h. ^*c*^ Yield was determined by ^1^H NMR analysis due to the volatile nature of 2A. ^*d*^ PdCl_2_ (10 mol%), PPh_3_ (40 mol%) were used. ^*e*^ P*n*-Bu_3_ (20 mol%), K_2_CO_3_ (2.0 equiv.), MeCN, 12 h. ^*f*^ 6.0 mmol scale. ^*g*^ Isolated as an acetamide by treating with Ac_2_O. ^*h*^ 20 mol% MePPh_2_, K_2_CO_3_ (2.0 equiv.), MeCN, 12 h.

Under these optimized conditions, the substrate scope of diarylketones was investigated (Fig. 2). Changing one of phenyl groups of the benzophenone (1) to a naphthyl or a *meta*-tolyl group also afforded diarylmethanes 2B and 2C in moderate yields. Biphenyl group worked as well (2D), however, installing a methoxy group at the *para*-position of the biphenyl decreased the yield (2E) even after increasing the amounts of catalysts, since this resulted in a lot of accompanying diarylmethanol (54% yield). The low yield was caused by the failure of the Pd/HCO_2_Na reduction of the phosphinate formed by the phospha-Brook rearrangement due to the electron-donating group. As a result, the phosphinate was hydrolyzed after quenching by water, resulting in the alcohol. Fluorinated diarylketones gave the corresponding diarylmethanes 2F and 2G in moderate yields. Diarylketones with an alkyne, bis-trifluoromethyl groups, or dimethoxy groups were tolerated to furnish the corresponding diarylmethanes 2H–2J. When the phenyl group of 1 was replaced by heteroaromatics such as pyridine or quinoline, the corresponding diarylmethanes 2K–2N were obtained in good to high yields. Even for cyclic ketones, the corresponding diarylmethanes 2O–2S were afforded, regardless of the ring size or the presence of heteroatoms. We also attempted this reaction with ketoprofen as well as its precursor, giving the corresponding diarylmethanes 2T and 2U. Additionally, diarylketones, which can be readily derivatized in one step from carboxylic-acid-containing drugs such as probenecid and adapalene, successfully converted to the corresponding diarylmethanes 2V and 2W, albeit in low yields. Fenofibrate, which is a well-known pharmaceutical, succeeded in giving product 2X in a moderate yield.

Next, we examined the substrate scope for the synthesis of tetraarylethanes 3 using the standard conditions (Table 1, entry 8). Benzophenone (1) gave 1,1,2,2-tetraphenylethane (3A) in 58% isolated yield, and the structure of 3A was assigned by X-ray crystallographic analysis. Mono- or di-tolylmethanones with methyl groups in the *para*-, *meta*-, or *ortho*-positions, as well as xylylmethanone smoothly generated the corresponding tetraarylethanes 3B–3E. Diarylketones bearing biphenyl and naphthyl groups afforded the corresponding tetraarylethanes 3F and 3G in moderate yields. Note that when unsymmetrical diarylketones are used, the resulting tetraarylethanes 3 become a mixture of diastereomers with a ratio of almost 1 : 1. A ketone with bis-4-fluorophenyl groups reacted well to give the desired product 3H in a high yield, and this reaction also proceeds on gram scale (61% yield). In contrast, reactions with a mono-fluorophenyl group gave 3I and 3J in moderate to low yields. Diarylketones with methoxy, alkoxy, dimethylamino, and NH_2_ groups were tolerated to furnish the corresponding tetraarylethanes 3K–3Q. In the case of low yields, the corresponding diarylmethanes and the diarylmethanols were obtained as by-products. Cyclic ketones such as dibenzosuberone did not progress at all under our standard conditions. Therefore, we re-optimized the reaction and found that tributyl phosphine was effective as a ligand to give 3R in 43% yield (the structure of 3R was assigned by X-ray crystallographic analysis). However, the reaction conditions were ineffective with other cyclic ketones (3S and 3T). Ketoprofen precursor and fenofibrate were applicable to this reaction as well (3U and 3V).

### Studies for elucidation of the reaction mechanism

**Fig. 3:**
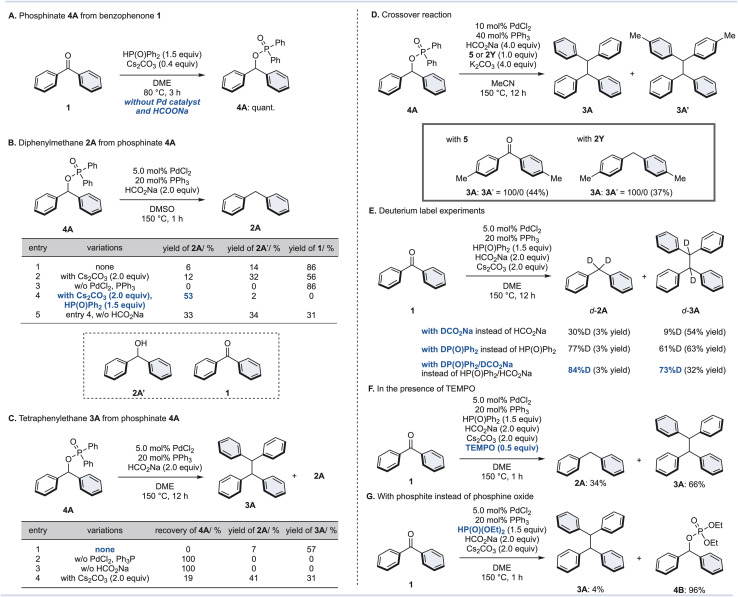
(A) Phosphinate 4A from benzophenone (1). (B) Diphenylmethane (2A) from phosphinate 4A. (C) Tetraphenylethane 3A from phosphinate 4A. (D) Crossover reaction. Numbers in the parentheses are yields of 3A. (E) Deuterium labeling experiments. (F) Standard conditions with TEMPO as a radical scavenger. (G) With phosphite instead of phosphine oxide.

We hypothesize the reaction mechanism as follows: (1) the diarylketone starting material could be converted to a diarylphosphinate *via* a phospha-Brook rearrangement; (2) then, reduction and dimerization by a Pd/HCO_2_Na catalytic system would lead to diarylmethanes and tetraarylethanes. However, their precise mechanisms and requirements for adequate conditions are not known. Therefore, we performed additional control experiments to elucidate the reaction mechanism for the synthesis of diarylmethane 2 and tetraarylethane 3 (Fig. 3). When benzophenone (1) was reacted with diphenylphosphine oxide in the presence of Cs_2_CO_3_ as a base without Pd/HCO_2_Na, phosphinate 4A was obtained quantitatively (Fig. 3A). Although stoichiometric amounts of metal carbonates (Cs_2_CO_3_ or K_2_CO_3_) are not required, the reaction did not proceed at all without the addition of metal carbonates. These results indicate that the base is critical for the formation of phosphinate 4A, but that high temperatures (150 °C), a palladium catalyst, and HCO_2_Na are not required (see the ESI for details†).

Next, we attempted the diphenylmethane (2A) synthesis from phosphinate 4A under our optimal conditions without addition of Cs_2_CO_3_ (Fig. 3B). Surprisingly, the desired diphenylmethane 2A was obtained in only 6% yield, and ketone 1 was obtained as the major product (entry 1). The addition of Cs_2_CO_3_ slightly improved the yield of 2A (12%), but increased the yield of alcohol 2A′ as well, which is an effect of phosphinate 4A being partially hydrolyzed (entry 2). Without the addition of the palladium catalyst, 2A was not produced at all and was completely converted to ketone 1 (entry 3). It was finally realized that the desired diphenylmethane 2A was obtained in 53% yield by adding diphenylphosphine oxide and Cs_2_CO_3_ (entry 4). In the absence of HCO_2_Na (entry 5), the yield of 2A decreased, but the yield increased for alcohol 2A′ and ketone 1. The existing equilibrium between ketone 1 and phosphinate 4A in DMSO suggests that reduction might be proceeding as soon as the phosphinate 4A is produced by the Pd/HCO_2_Na catalytic system.

Additional control experiments for the synthesis of tetraphenylethane 3A from phosphinate 4A were performed (Fig. 3C). Phosphinate 4A in DME in the presence of PdCl_2_/PPh_3_ and HCO_2_Na gave the desired tetraphenylethane 3A in 57% yield, along with 7% diphenylmethane 2A as a byproduct. This result is mostly consistent with the results from ketone 1 (Table 1, entry 8), but the requirements were further elaborated. Without the addition of a palladium catalyst or HCO_2_Na, the reaction hardly proceeded (entries 2 and 3). Furthermore, an excess amount of Cs_2_CO_3_ was found to inhibit the dimerization reaction (entry 4). Hence, the amount of Cs_2_CO_3_ in the dimerization of 1 was kept minimal (0.40 equiv., see Fig. 2).

Furthermore, the following crossover experiment was performed to confirm whether dimerization of the phosphinate 4A or cross-coupling with the diphenylketone (1)/diphenylmethane (2A) occurs (Fig. 3D). To this end, phosphinate 4A was reacted under our standard conditions for dimerization with di*p*-tolyl ketone 5 or diarylmethane 2Y (1.0 equiv. of each), respectively. If a crossover reaction had occurred, 3A′ would have been detected in addition to 3A, however 3A′ was not detected at all. These results suggest that a dimerization reaction of phosphinate 4A is proceeding.

Moreover, deuterium labelling experiments were conducted (Fig. 3E). Under optimal conditions for 2A synthesis, deuterated DCO_2_Na was added instead of HCO_2_Na to afford *d*-2A (30% D) and *d*-3A (9% D). When diphenylphosphine oxide (DP(O)Ph_2_) was used instead of diphenylphosphine oxide, this led to a dramatic increase in the deuterated ratio of *d*-2A (77% D) and *d*-3A (61% D). Finally, both deuterated agents were combined to give 2A (84% D) and 3A (73% D). These results suggest that diphenylphosphine oxide works as a hydrogen source in this reaction.

We also attempted this reductive transformation under a radical scavenger (Fig. 3F): in the presence of TEMPO, the reaction gave both 2A and 3A. This result confirmed that these reactions do not proceed through a radical pathway. Lastly, the same reaction was attempted using diethyl phosphite instead of diphenylphosphine oxide (Fig. 3G). This is because generally the phospha-Brook rearrangement is almost exclusively reported for phosphite esters.^39–42^ When this reaction was performed with diethyl phosphite, the phosphate ester 4B was obtained in 96% yield, but the desired tetraphenylethane 3A was formed in small amounts. Thus, the reaction proved to be effective only with diphenylphosphine oxide. Based on the results of these control experiments, it was concluded that the dimerization reaction also proceeds *via* diarylphosphinate. Although the exact reaction mechanism of dimerization remains unclear, similar dimerization reactions have proceeded when diaryl pseudohalides were used in the presence of Pd, and Ni catalysts,^43–45^ and we believe that dimerization occurs through disproportionation of the oxidative addition complexes of the Pd catalysts.

### Synthesis of triarylmethanes

**Fig. 4:**
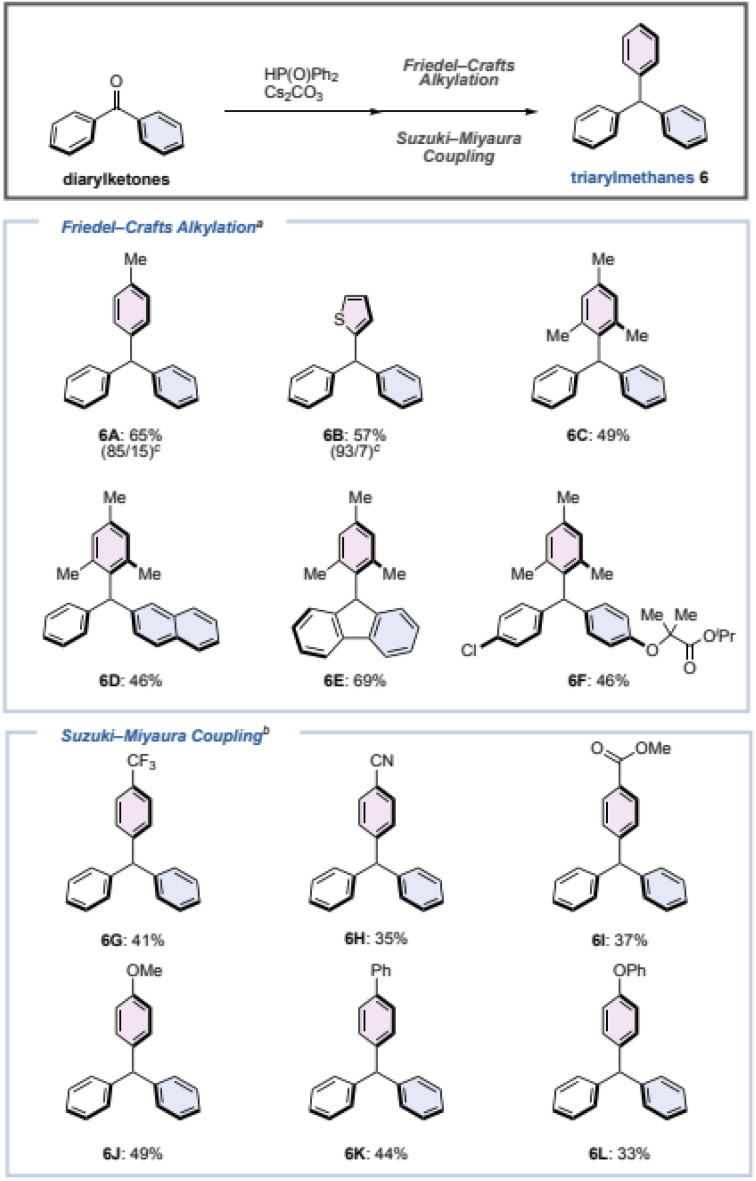
Synthesis of triarylmethanes. Conditions: ^*a*^ diarylketone (0.40 mmol), diphenylphosphine oxide (1.5 equiv.), Cs_2_CO_3_ (0.40 equiv.), DME (2.0 mL), 80 °C, 3 h; then arene (1.0 mL), TfOH (2.0 equiv.), RT, 2 min. ^*b*^ Diarylketone (0.40 mmol), diphenylphosphine oxide (1.2 equiv.), Cs_2_CO_3_ (2.0 equiv.), 1,4-dioxane (2.0 mL), 150 °C, 1 h; then arylboronic acid (1.5 equiv.), Pd(OAc)_2_ (10 mol%), P (*p*-tolyl)_3_ (40 mol%), 150 °C, 1 h. ^*c*^ The ratio of isomers (*para*/*ortho*-position (6A) or C_2_/C_3_ position (6B)) is shown in parentheses.

Next, we considered that the phosphinate is a (pseudo)halide, and that triarylmethane could be synthesized by screening for appropriate conditions. To the best of our knowledge, Friedel–Crafts-type arylations or cross-couplings of phosphite have been reported,^46–48^ but there is no report for phosphinates thus far. As a result of our investigation, we found the following two reaction conditions for the one-pot synthesis of triarylmethane from diarylketones (Fig. 4).

For electron-rich aromatic rings, Friedel–Crafts-type reaction conditions were effective. Benzophenone (1) was reacted with diphenyl phosphine oxide (1.5 equiv.) and Cs_2_CO_3_ (0.40 equiv.) in DME at 80 °C for 3 h to generate phosphinate 4A*in situ*, followed by the addition of toluene, thiophene, or mesitylene with TfOH (2.0 equiv.) at room temperature for 2 min to give the corresponding triarylmethanes 6A–6C in moderate yields. Although small amounts of positional isomers were produced when generating 6A and 6B, triarylmethanes can be synthesized rapidly from benzophenone (1) without the use of metal catalysts. Not only benzophenone (1), but also an unsymmetrical diaryl ketone, a cyclic diaryl ketone, and a diarylketone with substituents such as a chlorine and an alkoxy could be converted to the corresponding triarylmethanes 6D–6F in moderate yields. When an aromatic ring with an electron-withdrawing substituent was used as the substrate, a Suzuki–Miyaura-type coupling reaction was effective. Similarly, after the preparation of phosphinate 4A, the corresponding triarylmethanes were formed simply by adding arylboronic acid (1.5 equiv.), Pd(OAc)_2_ (10 mol%), and P (*p*-tolyl)_3_ (40 mol%) to the reaction vessel and stirring at 150 °C for 1 h. In addition to triarylmethanes 6G–6I with trifluoromethyl, cyano, and ester groups, triarylmethanes 6J–6L with methoxy, phenyl, and phenoxy groups at the *para* position of the aromatic ring could be synthesized.

### Synthetic utility and applications of this method

**Fig. 5:**
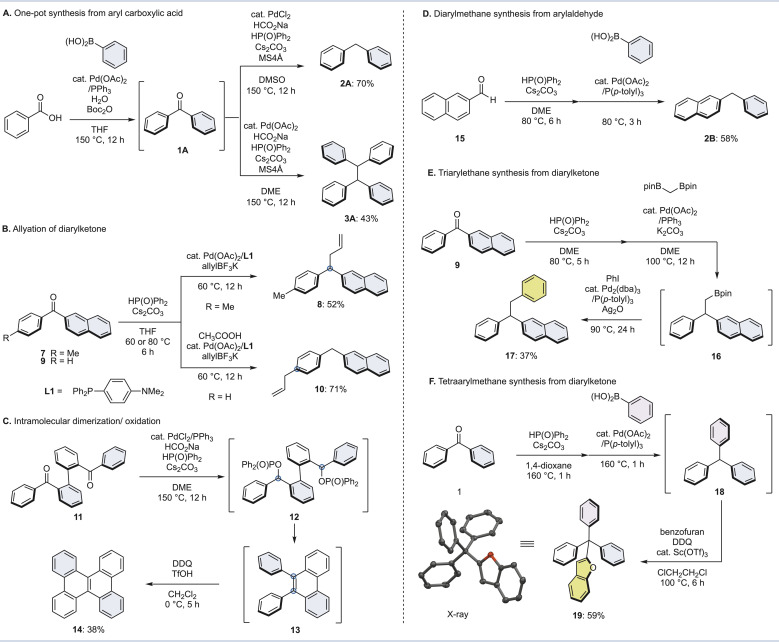
Synthetic utility and applications of deoxygenative transformation of diarylketones. (A) One-pot synthesis from aryl carboxylic acid. (B) Allylation of diarylketone. (C) Intramolecular dimerization/oxidation. (D) Diarylmethane synthesis from arylaldehyde. (E) Triarylethane synthesis from diarylketone. (F) Tetraarylmethane synthesis from diarylketone.

Finally, the synthetic utility and applications of this method were investigated (Fig. 5). As mentioned in the introduction, diaryl ketones can be readily prepared from aromatic carboxylic acids. Therefore, we demonstrated a one-pot diarylmethane/tetraarylethane synthesis from aryl carboxylic acids (Fig. 5A). The reaction of benzoic acid with phenylboronic acid and Boc_2_O in the presence of a palladium catalyst gave benzophenone (1) *via* a mixed acid anhydride.^33^ When 1 was subjected directly to the diverging conditions of Fig. 2 without isolation in one pot, diphenylmethane (2A) and tetraphenylmethane (3A) were successfully synthesized in 70% and 43% yields from benzoic acid, respectively. We also attempted a deoxygenative allylation of diarylketones (Fig. 5B). After phosphinate formation by a phospha-Brook rearrangement of naphthalen-2-yl(*p*-tolyl)methanone (7), allyltrifluoroborate with palladium catalyst/L1 led to benzyl substitution to form allyldiarylmethane 8 in 52% yield. In contrast, the use of naphthalen-2-yl (phenyl)methanone (9), which is a demethyl form of 7, afforded diarylmethane 10, which substituted at the *para*-position of the phenyl group. Diarylmethane 10 was likely formed from a π-benzyl palladium complex by a dearomatizing allylation reaction.^49,50^

Next, an intramolecular dimerization was carried out (Fig. 5C). Applying the reaction conditions from the tetraarylethane synthesis to compound 11, which is essentially two diphenylketones linked together, the diphosphinate intermediate 12 becomes diphenylphenanthrene 13 as the coupling and subsequent auto-oxidation proceeds. Oxidation of 13 with DDQ in the presence of TfOH gave dibenzo [*g*,*p*]chrysene 14 in good yield. This method has the potential to be used in the synthesis of various polycyclic aromatic hydrocarbons (PAHs).

Furthermore, the phospha-Brook rearrangement can be used to make diarylmethane from aromatic aldehydes (Fig. 5D). For example, diphenylphosphine oxide and a base are applied to 2-naphthaldehyde (15) to form the corresponding phosphinate, followed by an sp^3^ Suzuki–Miyaura-type coupling with phenylboronic acid to afford diarylmethane 2B in moderate yield. Since aromatic aldehydes are also inexpensive and readily available, a variety of diarylmethanes may be synthesized in a practical fashion.

Finally, we attempted to synthesize triarylethanes and tetraarylmethanes from diarylketones (Fig. 5E and F). The coupling reaction of 9 with diborylmethane (1.5 equiv.) underwent phospha-Brook rearrangement, followed by palladium catalyst and base to afford diarylethylboronate 16. A one-pot alkyl-Suzuki–Miyaura type coupling with phenylboronic acid was carried out without isolation of 16, and then triarylethanes 17 were successfully produced in 37% yield from diarylketone 9. Furthermore, after subjecting benzophenone (1) to the reaction conditions of Fig. 5 for the synthesis of triarylmethane, the resulting triphenylmethane 18 was treated with benzofuran, DDQ and catalytic Sc(OTf)_3_ to give tetraarylmethane 19.^51^ The structure of tetraarylmethane 19 was determined by X-ray crystallography.

## Conclusions

In summary, we developed a unified synthesis of multiply arylated alkanes from diarylketones. The key for this reaction is the readily generation of diarylphosphinates from diarylketones with diphenylphosphine oxide *via* a phospha-Brook rearrangement. Using this method, we have succeeded in synthesizing five different types of multiply arylated alkanes from diarylketones in a single step or one-pot. Expanding the range of nucleophiles for diarylphosphinates including enantioselective transformations is currently undergoing in our laboratory.^52^

## Data availability

All experimental data is available in the ESI.†

## Author contributions

J. Y. conceived and designed the study. M. B. K. performed the chemical experiments. M. B. K. and K. M. analyzed the data. K. K. performed the X-ray crystallography experiments and analyzed the obtained data. J. Y. wrote the manuscript and all authors discussed the results and commented on the final manuscript.

## Conflicts of interest

There are no conflicts to declare.

## Supplementary Material

SC-013-D2SC03720C-s001

SC-013-D2SC03720C-s002
