# Supplementary material for: Unified synthesis of multiply arylated alkanes by catalytic deoxygenative transformation of diarylketones
Source: Chem Sci. 2022 Aug 22;13(36):10743–51. doi: 10.1039/d2sc03720c (PMC9491083; doi:10.1039/d2sc03720c)

Electronic Supplementary Material (ESI) for Chemical Science.
This journal is © The Royal Society of Chemistry 2022

Supplementary Information

Unified Synthesis of Multiply Arylated Alkanes by Catalytic Deoxygenative Transformation of Diarylketones

Miki B. Kurosawa,[a] Kenta Kato,[a] Kei Muto,[b] and Junichiro Yamaguchi*[a]

a Department of Applied Chemistry, Waseda University, 513 Wasedatsurumakicho, Shinjuku, Tokyo 162-0041 Japan.

b Waseda Institute for Advanced Study, Waseda University, 513 Wasedatsurumakicho, Shinjuku, Tokyo 162-0041 Japan.

E-mail: junyamaguchi@waseda.jp

Table of Contents

1. General S2–S3
2. Pd-Catalyzed Deoxygenative Synthesis of Diarylmethanes from Diarylketones S4–S9
3. Pd-Catalyzed Deoxygenative Synthesis of Tetraarylethanes from Diarylketones S10–S18
4. Pd-Catalyzed Deoxygenative Synthesis of Triarylmethanes from Diarylketones S19–S24
5. One-Pot Synthesis of Diphenylmethane (**2A**) S25
6. One-Pot Synthesis of 1,1,2,2-Tetraphenylethane (**3A**) S25–S26
7. One-Pot Synthesis of 2-(1-(*p*-Tolyl)but-3-en-1-yl)naphthalene (**8**) S26
8. One-Pot Synthesis of 2-(4-Allylbenzyl)naphthalene (**10**) S27
9. Intramolecular Dimerization/ Oxidation S27–S28
10. 2-Benzylnaphthalene (**2B**) Synthesis from Arylaldehyde S28
11. 2-(1,2-Diphenylethyl)naphthalene (**16**) Synthesis from Diarylketone S29
12. 2-Tritylbenzofuran (**18**) Synthesis from Benzophenone (**1**) S30
13. Effect of Parameters S31
14. Mechanistic Study S32–S35
15. X-ray Crystal Structure Analysis S36–S41
16. References S42–S42
17. ^{1}H, ^{13}C, ^{19}F and ^{31}P NMR Spectra S44–S148

1. General

Unless otherwise noted, all reactants or reagents including dry solvents were obtained from commercial suppliers and used as received. Cs_2CO_3 was gifted by Iwatani Corporation. PPh_3 was purchased from KANTO Chemical. Diphenylphosphine oxide, HCO_2Na, benzophenone (**1**), naphthalen-2-yl(phenyl)methanone (**9**), phenyl(*m*-tolyl)methanone, [1,1'-biphenyl]-4-yl(phenyl)methanone, bis(4-fluorophenyl)methanone, (2-fluorophenyl)(4-fluorophenyl)methanone, bis(3-(trifluoromethyl)phenyl)methanone, phenyl(pyridin-2-yl)methanone, phenyl(pyridin-3-yl)methanone, phenyl(pyridin-4-yl)methanone, 7*H*-benzo[*de*]anthracen-7-one, 10,11-dihydro-5*H*-dibenzo[*a*,*d*][7]annulen-5-one, 9*H*-fluoren-9-one, 9*H*-xanthen-9-one, 9*H*-thioxanthen-9-one, 2-(3-benzoylphenyl)propanoic acid, 2-(3-benzoylphenyl)propanenitrile, isopropyl 2-(4-(4-chlorobenzoyl)phenoxy)-2-methylpropanoate, di-*p*-tolylmethanone (**5**), phenyl(*o*-tolyl)methanone, (3,4-dimethylphenyl)(phenyl)methanone, (4-fluorophenyl)(phenyl)methanone, (2-methoxyphenyl)(4-methoxyphenyl)methanone, (4-phenoxyphenyl)(phenyl)methanone, (4-(benzyloxy)phenyl)(phenyl)methanone, (4-(dimethylamino)phenyl)(phenyl)methanone, (3-aminophenyl)(phenyl)methanone, phenyl(thiophen-2-yl)methanone, and 2,4-diethyl-9*H*-thioxanthen-9-one were purchased from Tokyo Chemical Industry (TCI). DCO_2Na (>99% D), $NaBD_4$ (98% D), and (4-fluorophenyl)(4-(phenylethynyl)phenyl)methanone were purchased from Sigma-Aldrich. $PdCl_2$ and bis(4-methoxyphenyl)methanone were purchased from FUJIFILM Wako Pure Chemical Corporation. (4'-Methoxy-[1,1'-biphenyl]-4-yl)(phenyl)methanone,[1] phenyl(2-phenylquinolin-4-yl)methanone,[2] 4-benzoyl-*N*,*N*-dipropylbenzenesulfonamide,[3] (6-(3-(adamantan-1-yl)-4-methoxyphenyl)naphthalen-2-yl)(phenyl)methanone,[4] naphthalen-2-yl(*p*-tolyl)methanone (**7**),[5] and [1,1'-biphenyl]-2,2'-diylbis(phenylmethanone) (**11**)[6] were synthesized according to procedures and the spectra matched with those of compounds reported in the literature. Unless otherwise noted, all reactions were performed with dry solvents under an atmosphere of N_2 in dried glassware using standard vacuum-line techniques. All deoxygenative reactions of diarylketones were performed in 20-mL glass vessel tubes equipped with J. Young® O-ring tap and heated (IKA Plate RCT Digital) in an oil bath or a 9-well aluminum reaction block (IKA H 135.103 Block 9 × 16 ml) unless otherwise noted. All work-up and purification procedures were carried out with reagent-grade solvents under air unless otherwise noted.

Analytical thin-layer chromatography (TLC) was performed using Silica-gel 70 TLC Plate-Wako (0.25 mm). The developed chromatogram was analyzed by UV lamp (254 nm). Flash column chromatography was performed with Biotage Isolera® equipped with Biotage Sfär Cartridge Silica D columns. Preparative thin-layer chromatography (PTLC) was performed using Wakogel B5-F silica coated plates (0.75 mm) prepared in our laboratory. Preparative recycling gel permeation chromatography (GPC) was performed with a JAI LaboACE LC-5060 instrument equipped with JAIGEL-2HR columns using $CHCl_3$ as an eluent. High-resolution mass spectra (HRMS) were

conducted on Thermo Fisher Scientific ExactivePlus Orbitrap (ESI and DART). Nuclear magnetic resonance (NMR) spectra were recorded on a JEOL JNM-ECS-400 (^{1}H 400 MHz, ^{13}C 101 MHz), JEOL JNM-ECZ-400 (^{1}H 400 MHz, ^{13}C 101 MHz, ^{31}P 162 MHz, ^{19}F 376 MHz), or JEOL JNM-ECZ600R/S1 (^{13}C 151 MHz). Chemical shifts for ^{1}H NMR are expressed in parts per million (ppm) relative to tetramethylsilane (δ 0.00 ppm) and CHD_2SOCD_3 (δ 2.50 ppm) in DMSO-d_6. Chemical shifts for ^{13}C NMR are expressed in ppm relative to $CDCl_3$ (δ 77.0 ppm) and DMSO-d_6 (δ 39.5 ppm). Chemical shifts for ^{31}P NMR are expressed in ppm relative to H_3PO_4 (δ 0.00 ppm) as an external standard. Chemical shifts for ^{19}F NMR are expressed in ppm relative to fluorobenzene (δ –113.15 ppm) as an internal standard.

Data are reported as follows: chemical shift, multiplicity (s = singlet, d = doublet, dd = doublet of doublets, t = triplet, td = triplet of doublets, q = quartet, m = multiplet, brs = broad singlet), coupling constant (Hz), and integration.

2. Pd-Catalyzed Deoxygenative Synthesis of Diarylmethanes from Diarylketones

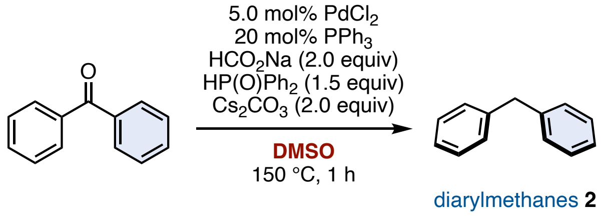

General Procedure

A 20-mL glass vessel equipped with J. Young® O-ring tap containing a magnetic stirring bar and Cs_2CO_3 (260.7 mg, 0.80 mmol, 2.0 equiv) was dried with a heat-gun *in vacuo* and filled with N_2 gas after cooling to room temperature. To this were added diarylketones (0.40 mmol, 1.0 equiv), $PdCl_2$ (3.6 mg, 0.020 mmol, 5.0 mol%), PPh_3 (21.0 mg, 0.080 mmol, 20 mol%), HCO_2Na (54.4 mg, 0.80 mmol, 2.0 equiv), and diphenylphosphine oxide (121.3 mg, 0.60 mmol, 1.5 equiv). The vessel was placed under vacuum and refilled N_2 gas three times. To this vessel was added DMSO (2.0 mL). The vessel was sealed with an O-ring tap and then heated at 150 °C for 1 h in a 9-well reaction block with stirring. After cooling the reaction mixture to room temperature, the mixture was added brine and extracted three times with hexane/EtOAc. The combined organic layer was dried over Na_2SO_4, filtrated, and then concentrated *in vacuo*. The residue was purified by PTLC to afford the corresponding product **2**.

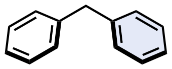

Diphenylmethane (2A)

Because **2A** was volatile, the yield of **2A** was determined as 93% by ^{1}H NMR analysis of crude mixture by using CH_2Br_2 as an internal standard. Diphenylmethane (**2A**) is commercially available from TCI (D0896).

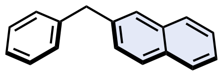

2-Benzylnaphthalene (2B)

Purification by PTLC (hexane/EtOAc = 4:1) afforded **2B** as a brown liquid (68.2 mg, 78% yield). The spectra were matched with those of the commercial reagent from Sigma-Aldrich (T154032).

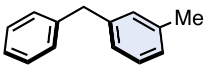

1-Benzyl-3-methylbenzene (2C)

Purification by PTLC (hexane/EtOAc = 4:1) afforded **2C** as a colorless liquid (40.3 mg, 55% yield). The spectra were matched with those of the commercial reagent from FUJIFILM Wako Pure Chemical Corporation (OR311219).

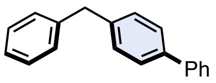

4-Benzyl-1,1'-biphenyl (2D)

Purification by PTLC (hexane/EtOAc = 4:1) afforded **2D** as a white solid (62.6 mg, 64% yield). The spectra were matched with those of the commercial reagent from TCI (B1519).

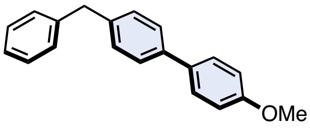

4-Benzyl-4’-methoxy-1,1’-biphenyl (2E)[7]

The reaction was conducted by using 10 mol% of $PdCl_2$ and 40 mol% of PPh_3. Purification by PTLC (hexane/EtOAc = 6:1) afforded **2E** as a white solid (36.3mg, 33% yield). ^{1}H NMR (400 MHz, $CDCl_3$) δ 7.50 (d, J = 8.0 Hz, 2H), 7.47 (d, J = 8.0 Hz, 2H), 7.30 (t, J = 8.0 Hz, 2H), 7.27–7.18 (m, 5H), 6.95 (d, J = 8.0 Hz, 2H), 4.01 (s, 2H), 3.83 (s, 3H); ^{13}C NMR (101 MHz, $CDCl_3$) δ 159.0, 141.1, 139.6, 138.6, 133.5, 129.3, 128.9, 128.5, 128.0, 126.8, 126.1, 114.1, 55.3, 41.5. The spectra matched with those of this compound reported in the literature.[7]

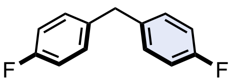

Bis(4-fluorophenyl)methane (2F)

Purification by PTLC (hexane/EtOAc = 4:1) afforded **2F** as a brown liquid (60.0 mg, 74% yield). The spectra were matched with those of the commercial reagent from TCI (D1925).

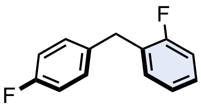

1-Fluoro-2-(4-fluorobenzyl)benzene (2G)

The reaction was conducted in 0.20 mmol scale. Purification by PTLC (hexane/$CHCl_3$ = 4:1) afforded **2G** as a colorless liquid (38.6 mg, 47% yield). ^{1}H NMR (400 MHz, $CDCl_3$) δ 7.23–7.09 (m, 4H), 7.08–7.00 (m, 2H), 7.00–6.92 (m, 2H), 3.96 (s, 2H); ^{13}C NMR (101 MHz, $CDCl_3$) δ 161.5 (d, $J_{C–F}$ = 245.3 Hz), 160.9 (d, $J_{C–F}$ = 246.6 Hz), 135.5 (d, $J_{C–F}$ = 3.7 Hz), 130.9 (d, $J_{C–F}$ = 4.7 Hz), 130.1 (d, $J_{C–F}$ = 8.0 Hz), 128.1 (d, $J_{C–F}$ = 8.0 Hz), 127.9 (d, $J_{C–F}$ = 22.8 Hz), 124.1 (d, $J_{C–F}$ = 3.7 Hz), 115.4 (d, $J_{C–F}$ = 22.0 Hz), 115.2 (d, $J_{C–F}$ = 21.4 Hz), 34.0 (d, $J_{C–F}$ = 3.2 Hz); ^{19}F NMR (376 MHz, $CDCl_3$) δ –117.2, –118.0; HRMS (DART) m/z calcd for $C_{13}H_9F_2$ [M–H]$^-$: 203.0667 found 203.0666.

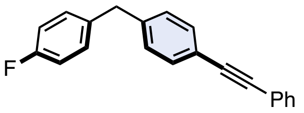

1-Fluoro-4-(4-(phenylethynyl)benzyl)benzene (2H)

Purification by PTLC (hexane/CH_2Cl_2 = 9:1) afforded **2H** as a white solid (68.3 mg, 60% yield). ^{1}H NMR (400 MHz, $CDCl_3$) δ 7.56–7.48 (m, 2H), 7.48–7.40 (m, 2H), 7.39–7.27 (m, 3H), 7.17–7.08 (m, 4H), 7.01–6.91 (m, 2H), 3.94 (s, 2H); ^{13}C NMR (101 MHz, $CDCl_3$) δ 161.5 (d, $J_{C–F}$ = 245.4 Hz), 141.2, 136.2 (d, $J_{C–F}$ = 3.2 Hz), 131.8, 131.5, 130.3 (d, $J_{C–F}$ = 8.1 Hz), 128.9, 128.3, 128.2, 123.3, 121.1, 115.3 (d, $J_{C–F}$ = 21.4 Hz), 89.3, 89.2, 40.9; ^{19}F NMR (376 MHz, $CDCl_3$) δ –117.1; HRMS (DART) *m*/*z* calcd for $C_{21}H_{19}NF$ $[M+NH_4]^+$: 304.1496 found 304.1495.

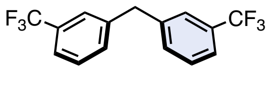

Bis(3-(trifluoromethyl)phenyl)methane (2I)

Purification by PTLC (hexane/EtOAc = 4:1) afforded **2I** as a yellow liquid (59.2 mg, 49% yield). The spectra were matched with those of the commercial reagent from FUJIFILM Wako Pure Chemical Corporation (006389).

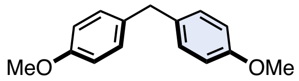

Bis(4-methoxyphenyl)methane (2J)

Purification by PTLC (hexane/EtOAc = 4:1) afforded **2J** as a colorless oil (42.3 mg, 46% yield). The spectra were matched with those of the commercial reagent from Sigma-Aldrich (AMBH97F060D7).

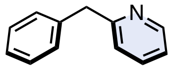

2-Benzylpyridine (2K)

Purification by PTLC (hexane/EtOAc = 4:1) afforded **2K** as a yellow liquid (53.7 mg, 79% yield). The spectra were matched with those of the commercial reagent from TCI (B0436).

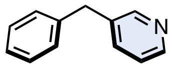

3-Benzylpyridine (2L)

Purification by PTLC (hexane/EtOAc = 4:1) afforded **2L** as a colorless liquid (49.7 mg, 73% yield). The spectra were matched with those of the commercial reagent from TCI (B1553).

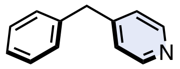

4-Benzylpyridine (2M)

Purification by PTLC (hexane/EtOAc = 4:1) afforded **2M** as a yellow liquid (61.7 mg, 91% yield). The spectra were matched with those of the commercial reagent from TCI (B0437).

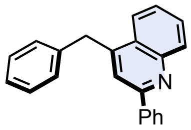

4-Benzyl-2-phenylquinoline (2N)[8]

The reaction was conducted for 12 h. Purification by PTLC (hexane/$CHCl_3$/CH_2Cl_2 = 2:1:1) afforded **2N** as a colorless liquid (105.0 mg, 89%). ^{1}H NMR (400 MHz, $CDCl_3$) δ 8.19 (d, J = 8.4 Hz, 1H), 8.09 (d, J = 8.4 Hz, 2H), 7.96 (d, J = 8.4 Hz, 1H), 7.65 (t, J = 7.2 Hz, 1H), 7.60 (s, 1H), 7.52–7.36 (m, 4H), 7.27 (t, J = 8.0 Hz, 2H), 7.23–7.15 (m, 3H), 4.42 (s, 2H); ^{13}C NMR (101 MHz, $CDCl_3$) δ 157.0, 148.5, 146.9, 139.6, 138.7, 130.4, 129.3, 129.2, 128.8, 128.7, 128.6, 127.5, 126.53, 126.50, 126.2, 123.7, 119.8, 38.4. The spectra matched with those of this compound reported in the literature.[8]

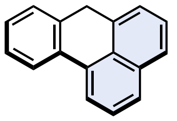

7*H*-Benzo[*de*]anthracene (2O)[9]

Purification by PTLC (hexane/$CHCl_3$/CH_2Cl_2 = 8:1:1) afforded **2O** as a white solid (50.3 mg, 58% yield). ^{1}H NMR (400 MHz, $CDCl_3$) δ 8.03 (d, J = 7.6 Hz, 1H), 8.00 (d, J = 7.6 Hz, 1H), 7.72 (d, J = 7.6 Hz, 1H), 7.66 (d, J = 7.6 Hz, 1H), 7.48 (t, J = 7.6 Hz, 1H), 7.43 (t, J = 7.6 Hz, 1H), 7.39–7.35 (m, 1H), 7.35–7.30 (m, 1H), 7.30–7.26 (m, 2H), 4.58 (s, 2H); ^{13}C NMR (101 MHz, $CDCl_3$) δ 134.3, 134.0, 133.3, 132.4, 131.1, 128.93, 128.85, 127.7, 127.6, 126.8, 126.2, 125.9, 125.5, 124.5, 123.3, 118.6, 34.4. The spectra matched with those of this compound reported in the literature.[9]

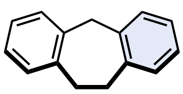

10,11-Dihydro-5*H*-dibenzo[*a,d*][7]annulene (2P)

The reaction was conducted by using 3.0 equiv of diphenylphosphine oxide for 12 h. Purification by PTLC (hexane/EtOAc = 4:1) afforded **2P** as a white solid (48.1 mg, 62% yield). The spectra were matched with those of the commercial reagent from Sigma-Aldrich (D104957).

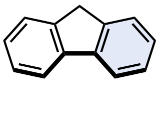

9*H*-Fluorene (2Q)

Purification by PTLC (hexane/EtOAc = 4:1) afforded **2Q** as a white solid (36.5 mg, 55% yield). The spectra were matched with those of the commercial reagent from TCI (F0017).

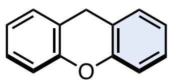

9*H*-Xanthene (2R)

The reaction was conducted by using 3.0 equiv of diphenylphosphine oxide for 12 h. Purification by PTLC (hexane/EtOAc = 4:1) afforded **2R** as a white solid (34.5 mg, 47% yield). The spectra were matched with those of the commercial reagent from TCI (X0003).

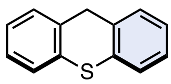

9*H*-Thioxanthene (2S)

The reaction was conducted by using 3.0 equiv of diphenylphosphine oxide for 12 h. Purification by PTLC (hexane/EtOAc = 4:1) afforded **2S** as a white solid (48.9 mg, 62% yield). The spectra were matched with those of the commercial reagent from FUJIFILM Wako Pure Chemical Corporation (327-27301).

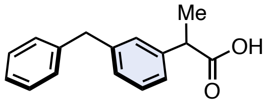

2-(3-Benzylphenyl)propanoic acid (2T)

The reaction was conducted by using 10 mol% of $PdCl_2$, 40 mol% of PPh_3, 0.40 equiv of Cs_2CO_3 and DME at 150 °C for 12 h. Purification by PTLC ($CHCl_3$) afforded **2T** as a colorless liquid (32.6 mg, 34% yield). ^{1}H NMR (400 MHz, $CDCl_3$) δ 7.31–7.26 (m, 2H), 7.25–7.14 (m, 6H), 7.08 (d, *J* = 7.2 Hz, 1H), 3.98 (s, 2H), 3.71 (q, *J* = 7.2 Hz, 1H), 1.50 (d, *J* = 7.2 Hz, 3H); ^{13}C NMR (101 MHz, $CDCl_3$) δ 178.6, 141.6, 140.8, 140.0, 128.9, 128.8, 128.5, 128.3, 128.0, 126.1, 125.2, 45.0, 41.8, 18.2; HRMS (ESI) *m*/*z* calcd for $C_{16}H_{16}O_2Na$ $[M+Na]^+$: 263.1042 found 263.1042.

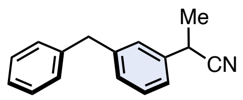

2-(3-Benzylphenyl)propanenitrile (2U)

Purification by PTLC (hexane/EtOAc = 4:1) afforded **2U** as a yellow liquid (48.7 mg, 55% yield). ^{1}H NMR (400 MHz, $CDCl_3$) δ 7.34–7.26 (m, 3H), 7.24–7.16 (m, 5H), 7.14 (d, *J* = 8.0 Hz, 1H), 3.99 (s, 2H), 3.85 (q, *J* = 7.6 Hz, 1H), 1.62 (d, *J* = 7.6 Hz, 3H); ^{13}C NMR (101 MHz, $CDCl_3$) δ 142.1, 140.4,

137.1, 129.2, 128.8, 128.54, 128.46, 127.2, 126.2, 124.3, 121.5, 41.7, 31.0, 21.3; HRMS (DART) *m*/*z* calcd for $C_{16}H_{14}N$ $[M–H]^-$: 220.1121 found 220.1119.

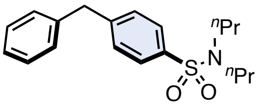

4-Benzyl-*N*,*N*-dipropylbenzenesulfonamide (2V)

Purification by PTLC (hexane/$CHCl_3$ = 9:1) afforded **2V** as a white solid (62.7 mg, 47% yield). ^1H NMR (400 MHz, $CDCl_3$) δ 7.71 (d, *J* = 7.6 Hz, 2H), 7.37–7.27 (m, 4H), 7.26–7.21 (m, 1H), 7.17 (d, *J* = 7.6 Hz, 2H), 4.04 (s, 2H), 3.05 (t, *J* = 7.6 Hz, 4H), 1.62–1.48 (m, 4H), 0.86 (t, *J* = 7.6 Hz, 6H); ^{13}C NMR (101 MHz, $CDCl_3$) δ 145.8, 139.7, 137.8, 129.4, 128.9, 128.6, 127.3, 126.5, 50.1, 41.6, 22.1, 11.1; HRMS (DART) *m*/*z* calcd for $C_{19}H_{26}NO_2S$ $[M+H]^+$: 332.1679 found 332.1676.

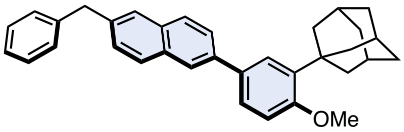

1-(5-(6-Benzylnaphthalen-2-yl)-2-methoxyphenyl)adamantane (2W)

The reaction was conducted by 0.20 mmol scale. Purification by PTLC (hexane/EtOAc = 6:1) afforded **2W** as a white solid (23.3 mg, 25% yield). ^1H NMR (400 MHz, $CDCl_3$) δ 7.93 (s, 1H), 7.79 (dd, *J* = 8.4, 2.0 Hz, 2H), 7.69 (dd, *J* = 8.4, 2.0 Hz, 1H), 7.63 (s, 1H), 7.57 (d, *J* = 2.4 Hz, 1H), 7.50 (dd, *J* = 8.4, 2.4 Hz, 1H), 7.34–7.26 (m, 3H), 7.26–7.16 (m, 3H), 6.97 (d, *J* = 8.4 Hz, 1H), 4.14 (s, 2H), 3.88 (s, 3H), 2.18 (s, 6H), 2.09 (s, 3H), 1.79 (s, 6H); ^{13}C NMR (101 MHz, $CDCl_3$) δ 158.5, 141.0, 138.8, 138.5, 138.3, 133.2, 132.4, 129.0, 128.5, 128.2, 128.0, 127.9, 126.8, 126.1, 125.8, 125.5, 124.8, 112.0, 55.1, 42.1, 40.6, 37.1, 29.1 (three peaks are missing due to overlapping); HRMS (ESI) *m*/*z* calcd for $C_{34}H_{35}O$ $[M+H]^+$: 459.2682 found 459.2681.

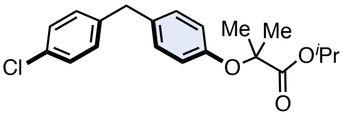

Isopropyl 2-(4-(4-chlorobenzyl)phenoxy)-2-methylpropanoate (2X)

Purification by PTLC (hexane/EtOAc = 4:1) afforded **2X** as a colorless liquid (58.3 mg, 42% yield). ^1H NMR (400 MHz, $CDCl_3$) δ 7.23 (d, *J* = 8.4 Hz, 2H), 7.08 (d, *J* = 8.4 Hz, 2H), 7.00 (d, *J* = 8.4 Hz, 2H), 6.77 (d, *J* = 8.4 Hz, 2H), 5.12–5.02 (m, 1H), 3.87 (s, 2H), 1.56 (s, 6H), 1.21 (d, *J* = 6.0 Hz, 6H); ^{13}C NMR (101 MHz, $CDCl_3$) δ 173.7, 154.0, 139.8, 134.0, 131.8, 130.2, 129.4, 128.5, 119.2, 79.1, 68.9, 40.3, 25.3, 21.5; HRMS (ESI) *m*/*z* calcd for $C_{20}H_{22}ClO_3$ $[M–H]^+$: 345.1252 found 345.1249.

3. Pd-Catalyzed Deoxygenative Synthesis of Tetraarylethanes from Diarylketones

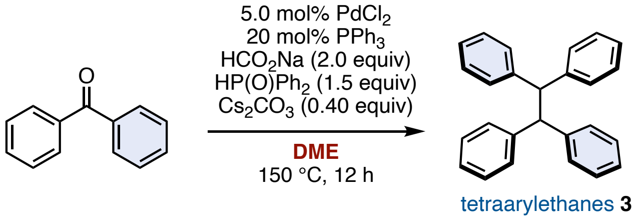

General Procedure

A 20-mL glass vessel equipped with J. Young® O-ring tap containing a magnetic stirring bar and Cs_2CO_3 (52.1 mg, 0.16 mmol, 0.40 equiv) was dried with a heat-gun *in vacuo* and filled with N_2 gas after cooling to room temperature. To this were added diarylketones **1** (0.40 mmol, 1.0 equiv), $PdCl_2$ (3.6 mg, 0.020 mmol, 5.0 mol%), PPh_3 (21.0 mg, 0.080 mmol, 20 mol%), HCO_2Na (54.4 mg, 0.80 mmol, 2.0 equiv), and diphenylphosphine oxide (121.3 mg, 0.60 mmol, 1.5 equiv). The vessel was placed under vacuum and refilled N_2 gas three times. To this vessel was added DME (2.0 mL). The vessel was sealed with an O-ring tap and then heated at 150 °C for 12 h in a 9-well reaction block with stirring. After cooling the reaction mixture to room temperature, the mixture was passed through a short silica-gel pad with EtOAc as an eluent. The filtrate was concentrated *in vacuo*. The residue was purified by PTLC or GPC to afford the corresponding product **3**.

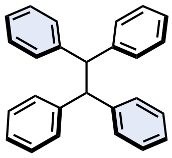

1,1,2,2-Tetraphenylethane (3A)[10]

Purification by PTLC (hexane/CH_2Cl_2 = 5:1) afforded **3A** as a white solid (38.6 mg, 58% yield). ^{1}H NMR (400 MHz, $CDCl_3$) δ 7.16 (d, J = 7.6 Hz, 8H), 7.10 (t, J = 7.6 Hz, 8H), 7.01 (t, J = 7.6 Hz, 4H), 4.77 (s, 2H); ^{13}C NMR (101 MHz, $CDCl_3$) δ 143.4, 128.5, 128.1, 125.8, 56.3. The spectra matched with those of this compound reported in the literature.[10]

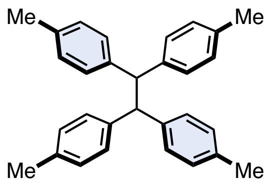

1,1,2,2-Tetra-*p*-tolylethane (3B)[11]

The reaction was conducted by using 2.0 equiv of Cs_2CO_3 for 3 h. Purification by PTLC ($CHCl_3$) afforded **3B** as a white solid (35.6 mg, 46% yield). ^{1}H NMR (400 MHz, $CDCl_3$) δ 7.04 (d, J = 8.0 Hz, 8H), 6.89 (d, J = 8.0 Hz, 8H), 4.68 (s, 2H), 2.17 (s, 12H); ^{13}C NMR (101 MHz, $CDCl_3$) δ 141.1, 134.9, 128.8, 128.2, 55.3, 20.9. The spectra matched with those of this compound reported in the literature.[11]

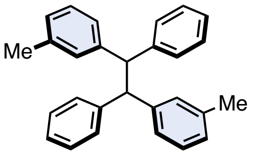

1,2-Diphenyl-1,2-di-*m*-tolylethane (3C)

The reaction was conducted by using 10 mol% of $PdCl_2$, 40 mol% of P(*m*-tolyl)$_3$, 2.0 equiv of Rb_2CO_3 and MeCN at 110 °C. Purification by PTLC (hexane/EtOAc = 4:1) and then GPC to afford **3C** as a white solid (43.4 mg, 60% yield). The product was characterized by the mixture of diastereomers (1:1). ^{1}H NMR (400 MHz, $CDCl_3$) δ 7.19–7.13 (m, 4H), 7.12–7.05 (m, 4H), 7.02–6.92 (m, 8H), 6.84–6.78 (m, 2H), 4.71 (s, 2H), 2.18 (s, 3H), 2.17 (s, 3H); ^{13}C NMR (101 MHz, $CDCl_3$) δ 143.7, 143.6, 143.4, 143.3, 137.5, 137.4, 129.5, 128.5, 128.0, 127.9, 126.6, 125.7, 125.38, 125.35, 56.2, 21.4; HRMS (DART) *m*/*z* calcd for $C_{28}H_{30}N$ $[M+NH_4]^+$: 380.2373 found 380.2372.

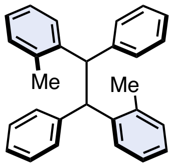

1,2-Diphenyl-1,2-di-*o*-tolylethane (3D)

The reaction was conducted by using 10 mol% of $PdCl_2$, 40 mol% of P(*m*-tolyl)$_3$, 2.0 equiv of Rb_2CO_3 and MeCN at 110 °C. Crude ^{1}H NMR analysis showed **major-3D**:**minor-3D** = 53:47; ^{1}H NMR peaks at 5.02 ppm (s, 2H) and 4.81 ppm (s, 1.74H) were used. Purification by PTLC (hexane/EtOAc = 4:1) afforded **3D** as a white solid (35.5 mg, 49% yield, as a mixture of diastereomers; major/minor = 53:47).

For major isomer: ^{1}H NMR (400 MHz, $CDCl_3$) δ 7.22 (d, J = 8.0 Hz, 2H), 7.10–6.91 (m, 14H), 6.88 (d, J = 8.0 Hz, 2H), 5.02 (s, 2H), 2.14 (s, 6H); For minor isomer: ^{1}H NMR (400 MHz, $CDCl_3$) δ 7.38 (d, J = 8.0 Hz, 2H), 7.10–6.91 (m, 14H), 6.88 (d, J = 8.0 Hz, 2H), 4.81 (s, 2H), 2.28 (s, 6H). ^{13}C NMR of diastereomer mixture (101 MHz, $CDCl_3$) δ 142.8, 142.3, 141.7, 141.0, 136.2, 135.9, 130.4, 130.2, 129.3, 128.9, 128.2, 127.8, 127.7, 127.0, 125.81, 125.75, 125.7, 52.7, 51.1, 20.0, 19.9; HRMS (DART) *m*/*z* calcd for $C_{28}H_{30}N$ $[M+NH_4]^+$: 380.2373 found 380.2372.

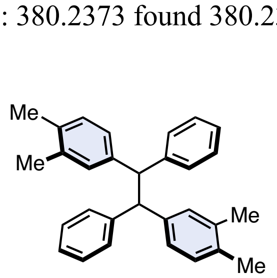

1,2-Bis(3,4-dimethylphenyl)-1,2-diphenylethane (3E)

The reaction was conducted by using 10 mol% of $PdCl_2$, 40 mol% of P(*m*-tolyl)$_3$, 2.0 equiv of Rb_2CO_3 and MeCN at 110 °C. Purification by PTLC (hexane/EtOAc = 4:1) and then GPC to afford **3C**

as a beige solid (29.2 mg, 37% yield). The product was characterized by the mixture of diastereomers. ^{1}H NMR (400 MHz, $CDCl_3$) δ 7.23–7.15 (m, 2H), 7.15–7.02 (m, 6H), 7.02–6.93 (m, 4H), 6.93–6.81 (m, 4H), 4.68 (s, 2H), 2.20–2.06 (m, 12H); ^{13}C NMR (101 MHz, $CDCl_3$) δ 144.2, 144.1, 141.0, 140.9, 136.0, 133.7, 130.0, 129.9, 129.33, 129.28, 128.4, 128.03, 127.99, 125.6, 125.53, 125.48, 55.8, 19.81, 19.77, 19.3; HRMS (DART) *m/z* calcd for $C_{30}H_{34}N$ $[M+NH_4]^+$: 408.2686 found 408.2682.

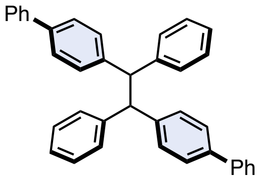

1,2-Di([1,1'-biphenyl]-4-yl)-1,2-diphenylethane (3F)

The reaction was conducted for 3 h. Purification by PTLC (hexane/EtOAc = 6:1) afforded **3F** as a white solid (41.3 mg, 42% yield). The product was characterized by the mixture of diastereomers. ^{1}H NMR (400 MHz, $CDCl_3$) δ 7.49 (d, J = 8.0 Hz, 4H), 7.40–7.33 (m, 8H), 7.30–7.18 (m, 10H), 7.17–7.10 (m, 4H), 7.04 (t, J = 8.0 Hz, 2H), 4.85 (s, 2H); ^{13}C NMR (101 MHz, $CDCl_3$) δ 143.41, 143.36, 142.6, 142.5, 140.7, 138.54, 138.49, 128.9, 128.6, 128.5, 128.3, 128.2, 127.0, 126.9, 126.8, 126.0, 125.9, 56.0; HRMS (DART) *m/z* calcd for $C_{38}H_{34}N$ $[M+NH_4]^+$: 504.2686 found 504.2682.

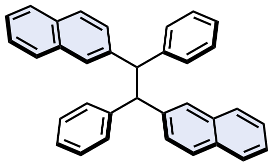

1,2-Di(naphthalen-2-yl)-1,2-diphenylethane (3G)

The reaction was conducted by using 0.40 equiv of K_2CO_3. Crude ^{1}H NMR analysis showed **major-3G**:**minor-3G** = 51:49; ^{1}H NMR peaks at 5.09 ppm (s, 2H) and 5.08 ppm (s, 1.95H) were used. Purification by PTLC (hexane/EtOAc = 6:1) afforded **3G** as a cream solid (50.6 mg, 58% yield, as a mixture of diastereomers; major/minor = 51:49).

For major isomer: ^{1}H NMR (400 MHz, $CDCl_3$) δ 7.71–7.56 (m, 8H), 7.44–7.20 (m, 10H), 7.14–6.92 (m, 6H), 5.09 (s, 2H); For minor isomer: ^{1}H NMR (400 MHz, $CDCl_3$) δ 7.71–7.56 (m, 8H), 7.44–7.26 (m, 7H), 7.25–7.20 (m, 3H), 7.14–6.92 (m, 6H), 5.08 (s, 2H); ^{13}C NMR (101 MHz, $CDCl_3$) of diastereomer mixture δ 143.4, 143.2, 141.0, 140.8, 133.38, 133.35, 131.9, 128.63, 128.56, 128.22, 128.16, 127.9, 127.8, 127.69, 127.66, 127.5, 127.4, 127.1, 127.0, 125.9, 125.7, 125.6, 125.24, 125.21, 56.2; HRMS (DART) *m/z* calcd for $C_{34}H_{30}N$ $[M+NH_4]^+$: 452.2373 found 452.2369.

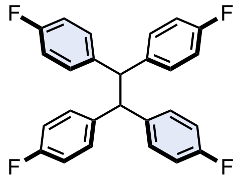

1,1,2,2-Tetrakis(4-fluorophenyl)ethane (3H)[10]

Purification by PTLC (hexane/EtOAc = 6:1) afforded **3H** as a white solid (75.8 mg, 93% yield, 0.40 mmol scale). Purification by Isolera® (hexane/EtOAc = 9:1 to 3:2) afforded **3H** as a white solid (747 mg, 61% yield, 6.0 mmol scale). ^{1}H NMR (400 MHz, $CDCl_3$) δ 7.08–7.01 (m, 8H), 6.87–6.79 (m, 8H), 4.62 (s, 2H); ^{13}C NMR (101 MHz, $CDCl_3$) δ 161.1 (d, $J_{C–F}$ = 247.2 Hz), 138.5 (d, $J_{C–F}$ = 2.9 Hz), 129.7 (d, $J_{C–F}$ = 7.8 Hz), 115.2 (d, $J_{C–F}$ = 21.3 Hz), 55.2; ^{19}F NMR (376 MHz, $CDCl_3$) δ –116.6. The spectra matched with those of this compound reported in the literature.[10]

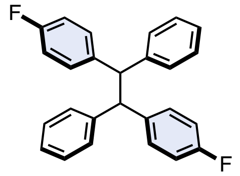

1,2-Bis(4-fluorophenyl)-1,2-diphenylethane (3I)

The reaction was conducted for 1 h. Purification by PTLC (hexane/$CHCl_3$/CH_2Cl_2 = 4:1:1) afforded **3I** as a white solid (39.2 mg, 53% yield). The product was characterized by the mixture of diastereomers. ^{1}H NMR (400 MHz, $CDCl_3$) δ 7.20–6.97 (m, 14H), 6.86–6.72 (m, 4H), 4.70 (s, 2H); ^{13}C NMR (101 MHz, $CDCl_3$) δ 161.03 (d, $J_{C–F}$ = 246.3 Hz), 161.01 (d, $J_{C–F}$ = 245.1 Hz), 143.0, 142.9, 139.1 (d, $J_{C–F}$ = 3.2 Hz), 139.0 (d, $J_{C–F}$ = 3.3 Hz), 129.81 (d, $J_{C–F}$ = 7.7 Hz), 129.78 (d, $J_{C–F}$ = 7.8 Hz), 128.4, 128.3, 126.12, 126.07, 115.1 (d, $J_{C–F}$ = 21.0 Hz), 115.0 (d, $J_{C–F}$ = 21.3 Hz), 55.7; ^{19}F NMR (376 MHz, $CDCl_3$) δ –117.1, –117.2; HRMS (DART) *m/z* calcd for $C_{26}H_{24}NF_2$ $[M+NH_4]^+$: 388.1871 found 388.1863.

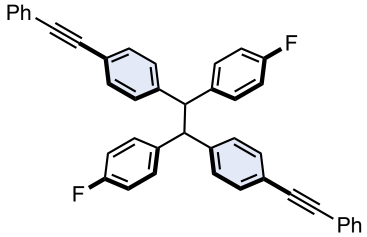

1,2-Bis(4-fluorophenyl)-1,2-bis(4-(phenylethynyl)phenyl)ethane (3J)

The reaction was conducted by 2.0 equiv of Cs_2CO_3. Purification by PTLC (hexane/EtOAc = 4:1) afforded **3J** as a yellow solid (33.2 mg, 29% yield). The product was characterized by the mixture of diastereomers. ^{1}H NMR (400 MHz, $CDCl_3$) δ 7.51–7.43 (m, 4H), 7.39–7.27 (m, 10H), 7.14–7.02 (m, 8H), 6.87–6.77 (m, 4H), 4.69 (s, 2H); ^{13}C NMR (101 MHz, $CDCl_3$) δ 161.2 (d, $J_{C–F}$ = 246.4 Hz), 143.1, 143.0, 138.3 (d, $J_{C–F}$ = 3.2 Hz), 138.2 (d, $J_{C–F}$ = 3.0 Hz), 131.7, 131.5, 129.8 (d, $J_{C–F}$ = 8.1 Hz), 128.4, 128.3, 128.21, 128.18, 123.22, 123.19, 121.12, 121.09, 115.3 (d, $J_{C–F}$ = 21.3 Hz), 115.23 (d, $J_{C–F}$ = 21.4

Hz), 89.3, 89.1, 55.5; ^{19}F NMR (376 MHz, $CDCl_3$) δ –116.5; HRMS (DART) *m*/*z* calcd for $C_{42}H_{29}F_2$ $[M+H]^+$: 571.2232 found 571.2227.

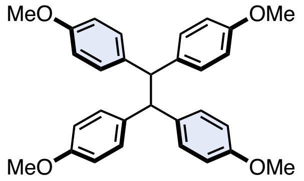

1,1,2,2-Tetrakis(4-methoxyphenyl)ethane (3K)[11]

The reaction was conducted by using 2.0 equiv of K_2CO_3 and MeCN. Purification by PTLC (hexane/EtOAc = 6:1) afforded **3K** as a white solid (50.9 mg, 56% yield). ^{1}H NMR (400 MHz, $CDCl_3$) δ 7.02 (d, *J* = 8.8 Hz, 8H), 6.65 (d, *J* = 8.8 Hz, 8H), 4.57 (s, 2H), 3.68 (s, 12H); ^{13}C NMR (101 MHz, $CDCl_3$) δ 157.9, 133.7, 129.7, 113.8, 55.2, 40.1.

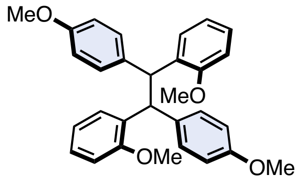

1,2-Bis(2-methoxyphenyl)-1,2-bis(4-methoxyphenyl)ethane (3L)[12]

The reaction was conducted by using 2.0 equiv of K_2CO_3 and MeCN. Crude ^{1}H NMR analysis showed **major-3L**:**minor-3L** = 55:45; ^{1}H NMR peaks at 5.23 ppm (s, 2H) and 5.33 ppm (s, 1.61H) were used. Purification by PTLC ($CHCl_3$) afforded **3L** as a white solid (28.0 mg, 31% yield, as a mixture of diastereomers; major/minor = 55:45).

For major isomer: ^{1}H NMR (400 MHz, $CDCl_3$) δ 7.28 (dd, *J* = 7.6, 1.6 Hz, 2H), 7.13–7.06 (m, 4H), 7.01–6.93 (m, 2H), 6.73 (td, *J* = 7.6, 1.6 Hz, 2H), 6.68–6.57 (m, 6H), 5.23 (s, 2H), 3.72 (s, 6H), 3.68 (s, 6H); For minor isomer: ^{1}H NMR (400 MHz, $CDCl_3$) δ 7.34 (dd, *J* = 7.6, 1.6 Hz, 2H), 7.13–7.06 (m, 4H), 7.01–6.93 (m, 2H), 6.79 (td, *J* = 7.6, 1.6 Hz, 2H), 6.68–6.57 (m, 6H), 5.33 (s, 2H), 3.68 (s, 6H), 3.66 (s, 6H); ^{13}C NMR of diastereomer mixture (101 MHz, $CDCl_3$) δ 157.2, 157.1, 156.62, 156.56, 136.0, 135.9, 133.0, 132.8, 129.7, 129.5, 128.4, 127.9, 126.51, 126.45, 120.4, 120.2, 113.1, 112.9, 110.6, 110.4, 55.5, 54.99, 54.98. The spectra matched with those of this compound reported in the literature.[12]

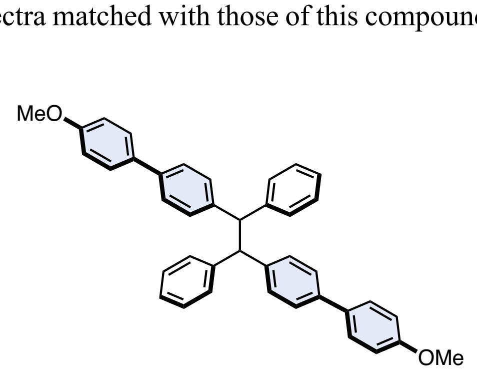

1,2-Bis(4'-methoxy-[1,1'-biphenyl]-4-yl)-1,2-diphenylethane (3M)

Purification by PTLC (hexane/$CHCl_3$ = 1:9) afforded **3M** as a white solid (35.6 mg, 33% yield). The product was characterized by the mixture of diastereomers. ^{1}H NMR (400 MHz, $CDCl_3$) δ 7.45–7.38 (m, 4H), 7.35–7.28 (m, 4H), 7.28–7.17 (m, 8H), 7.17–7.07 (m, 4H), 7.07–6.98 (m, 2H), 6.93–6.85 (m, 4H), 4.84 (s, 2H), 3.80 (s, 6H); ^{13}C NMR (101 MHz, $CDCl_3$) δ 158.9, 143.6, 141.9, 138.1, 133.3, 128.8, 128.5, 128.23, 128.16, 127.8, 126.42, 126.35, 125.9, 114.0, 55.9, 55.3; HRMS (DART) *m*/*z* calcd for $C_{40}H_{38}O_2N$ $[M+NH_4]^+$: 564.2897 found 564.2892.

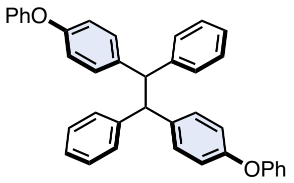

1,2-Bis(4-phenoxyphenyl)-1,2-diphenylethane (3N)

The reaction was conducted by using 10 mol% of $PdCl_2$, 40 mol% of P(*m*-tolyl)$_3$, 2.0 equiv of Rb_2CO_3 and MeCN at 110 °C. Purification by PTLC (hexane/$CHCl_3$ = 9:1) afforded **3N** as a white solid (36.9 mg, 36% yield). The product was characterized by the mixture of diastereomers. ^{1}H NMR (400 MHz, $CDCl_3$) δ 7.39–7.22 (m, 4H), 7.22–7.00 (m, 16H), 7.03–6.98 (m, 4H), 6.82–6.70 (m, 4H), 4.71 (s, 2H); ^{13}C NMR (101 MHz, $CDCl_3$) δ 157.4, 155.0, 154.9, 143.4, 143.2, 138.7, 138.5, 129.8, 129.7, 129.60, 129.55, 128.51, 128.45, 128.2, 126.0, 122.89, 122.87, 118.8, 118.47, 118.45, 55.9; HRMS (DART) *m*/*z* calcd for $C_{38}H_{34}O_2N$ $[M+NH_4]^+$: 536.2584 found 536.2584.

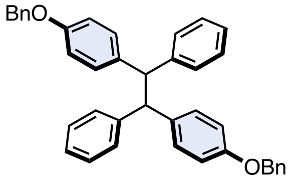

1,2-Bis(4-(benzyloxy)phenyl)-1,2-diphenylethane (3O)

The reaction was conducted by using 2.0 equiv of Cs_2CO_3. Purification by PTLC (hexane/EtOAc = 4:1) and then GPC afforded **3O** as a white solid (50.3 mg, 46% yield, as a mixture of diastereomers; major/minor = 50:50). The product was characterized by the mixture of diastereomers. ^{1}H NMR (400 MHz, $CDCl_3$) δ 7.46–7.26 (m, 10H), 7.22–6.96 (m, 14H), 6.80–6.66 (m, 4H), 4.92 (s, 2H), 4.90 (s, 2H), 4.67 (s, 2H); ^{13}C NMR (101 MHz, $CDCl_3$) δ 156.79, 156.78, 143.89, 143.85, 137.1, 136.10, 136.07, 129.42, 129.39, 128.5, 128.4, 128.12, 128.08, 127.9, 127.5, 125.7, 114.5, 114.4, 69.9, 69.8, 55.7; HRMS (ESI) *m*/*z* calcd for $C_{40}H_{38}O_2N$ $[M+NH_4]^+$: 564.2897 found 564.2896.

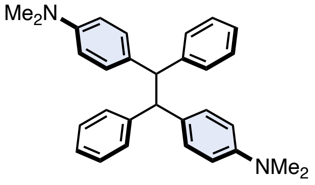

4,4'-(1,2-Diphenylethane-1,2-diyl)bis(*N*,*N*-dimethylaniline) (3P)

The reaction was conducted by using 2.0 equiv of Cs_2CO_3, 10 mol% of $PdCl_2$ and 40 mol% of PPh_2. Purification by PTLC ($CHCl_3$/MeOH 19:1, and then hexane/EtOAc = 4:1) and then GPC afforded **3P** as a white solid (30.3 mg, 36% yield). The product was characterized by the mixture of diastereomers. ^{1}H NMR (400 MHz, $CDCl_3$) δ 7.18–7.13 (m, 4H), 7.11–7.07 (m, 4H), 7.00–6.97 (m, 6H), 6.50–6.46 (m, 4H), 4.63 (s, 2H), 2.80 (s, 12H); ^{13}C NMR (101 MHz, $CDCl_3$) δ 148.5, 144.8, 132.1, 129.1, 128.5, 128.0, 125.3, 112.5, 55.4, 40.6; HRMS (ESI) *m*/*z* calcd for $C_{30}H_{33}N_2$ $[M+H]^+$: 421.2638 found 421.2636.

Note: The product contains some impurities that were unable to remove using GPC.

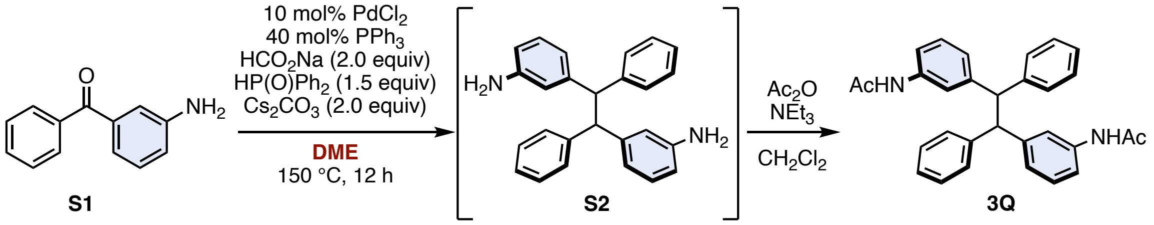

3,3'-(1,2-Diphenylethane-1,2-diyl)dianiline (3Q)

The reaction using **S1** was conducted by using 2.0 equiv of Cs_2CO_3, 10 mol% of $PdCl_2$ and 40 mol% of PPh_2 for 12 h. Crude mixture of **S2** was dissolved in CH_2Cl_2 (1.75 mL). To this solution, NEt_3 (166 µL, 1.2 mmol, 3.0 equiv) and then Ac_2O (79 µL, 0.84 mmol, 2.1 equiv) were slowly added at 0 °C. After stirring the mixture for 3 h at room temperature, the reaction mixture was added saturated $Na_2S_2O_3$ aq. The mixture was extracted three times with CH_2Cl_2. The combined organic layer was washed with brine, dried over $MgSO_4$, filtrated, and concentrated *in vacuo*. The residue was purified by PTLC ($CHCl_3$/MeOH = 19:1) to afford a cream solid. The obtained solid was washed with Et_2O to afford **3Q** as a cream solid (29.1 mg, 32% yield). ^{1}H NMR (400 MHz, DMSO-d_6) δ 9.77 (s, 2H), 7.61–7.51 (m, 2H), 7.40–7.28 (m, 4H), 7.28–7.20 (m, 2H), 7.20–7.13 (m, 2H), 7.13–6.98 (m, 6H), 6.98–6.87 (m, 2H), 4.97 (s, 2H), 3.38 (s, 6H); ^{13}C NMR (101 MHz, DMSO-d_6) δ 168.2, 144.6, 144.1, 139.1, 128.4, 128.2, 128.1, 125.7, 122.8, 119.0, 116.7, 54.6, 24.0; HRMS (ESI) *m*/*z* calcd for $C_{30}H_{29}N_2O_2$ $[M+H]^+$: 449.2226 found 449.2224.

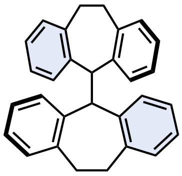

10,10',11,11'-Tetrahydro-5*H*,5'*H*-5,5'-bidibenzo[*a*,*d*][7]annulene (3R)[13]

The reaction was conducted by using 20 mol% of P^nBu_3, 2.0 equiv of K_2CO_3 and MeCN. Purification by PTLC (hexane/EtOAc = 4:1) and then GPC afforded **3R** as a white solid (33.6 mg, 43% yield). The product was characterized by the mixture of isomers. ^{1}H NMR (400 MHz, $CDCl_3$) δ 7.06 (d,

J = 7.6 Hz, 4H), 6.96 (t, J = 7.6 Hz, 4H), 6.69 (t, J = 7.6 Hz, 4H), 6.53 (d, J = 7.6 Hz, 4H), 4.78 (s, 2H), 3.81–3.69 (m, 4H), 3.09–2.97 (m, 4H); ^{13}C NMR (101 MHz, $CDCl_3$) δ 139.4, 139.2, 131.9, 130.1, 126.4, 125.1, 60.7, 33.9. The spectra matched with those of this compound reported in the literature.[13]

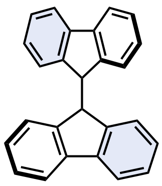

9*H*,9'*H*-9,9'-Bifluorene (3S)[10]

The reaction was conducted in 0.20 mmol scale by using 0.40 equiv of K_2CO_3. Purification by PTLC (hexane/EtOAc = 6:1) afforded **3S** as a yellow solid (10.6 mg, 32% yield). ^{1}H NMR (400 MHz, $CDCl_3$) δ 7.65 (d, J = 7.2 Hz, 4H), 7.35–7.27 (m, 4H), 7.09 (t, J = 7.2 Hz, 4H), 7.01–6.90 (m, 4H), 4.84 (s, 2H); ^{13}C NMR (101 MHz, $CDCl_3$) δ 144.6, 141.6, 127.4, 126.8, 124.2, 119.8, 49.9. The spectra matched with those of this compound reported in the literature.[10]

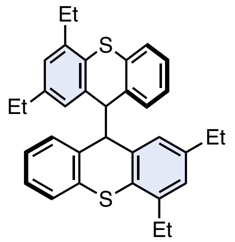

2,2',4,4'-Tetraethyl-9*H*,9'*H*-9,9'-bithioxanthene (3T)

The reaction was conducted by using 20 mol% of $MePPh_2$, 2.0 equiv of K_2CO_3 and MeCN. Crude ^{1}H NMR analysis showed **major-3T**:**minor-3T** = 58:42; ^{1}H NMR peaks at 4.53 ppm (s, 2H) and 4.50 ppm (s, 1.44H) were used. Purification by PTLC (hexane) afforded **3T** as a white solid (20.6 mg, 20% yield, as a mixture of diastereomers; major/minor = 58:42).

For major isomer: ^{1}H NMR (400 MHz, $CDCl_3$) δ 7.46 (d, J = 8.0 Hz, 2H), 7.12–7.02 (m, 2H), 6.84–6.74 (m, 4H), 6.32 (d, J = 8.0 Hz, 2H), 6.08–6.04 (m, 2H), 4.53 (s, 2H), 2.96–2.73 (m, 4H), 2.34–2.20 (m, 4H), 1.33 (t, J = 7.6 Hz, 6H), 0.91 (t, J = 7.6 Hz, 6H); For minor isomer: ^{1}H NMR (400 MHz, $CDCl_3$) δ 7.44 (d, J = 8.0 Hz, 2H), 7.12–7.02 (m, 2H), 6.84–6.74 (m, 4H), 6.29 (d, J = 8.0 Hz, 2H), 6.08–6.04 (m, 2H), 4.50 (s, 2H), 2.96–2.73 (m, 4H), 2.34–2.20 (m, 4H), 1.33 (t, J = 7.6 Hz, 6H), 0.91 (t, J = 7.6 Hz, 6H); ^{13}C NMR of diastereomer mixture (101 MHz, $CDCl_3$) δ 141.6, 141.5, 140.10, 140.05, 136.6, 136.5, 135.9, 132.5, 132.3, 130.0, 129.9, 127.64, 127.57, 127.3, 126.4, 126.2, 126.1, 125.94, 125.89, 125.4, 48.5, 48.3, 28.13, 28.05, 27.2, 15.8, 15.5, 15.0, 14.7; HRMS (DART) m/z calcd for $C_{34}H_{38}S_2N$ $[M+NH_4]^+$: 524.2440 found 524.2446.

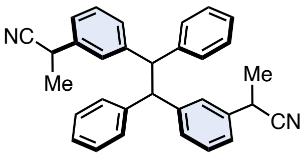

2,2'-((1,2-Diphenylethane-1,2-diyl)bis(4,1-phenylene))dipropanenitrile (3U)

The reaction was conducted by using 2.0 equiv of K_2CO_3 and MeCN. Purification by PTLC ($CHCl_3$) afforded **3U** as a white solid (21.7 mg, 25% yield). The product was characterized by the mixture of isomers. ^{1}H NMR (400 MHz, $CDCl_3$) δ 7.23–7.10 (m, 12H), 7.10–7.03 (m, 4H), 7.03–6.96 (m, 2H), 4.77–4.72 (m, 2H), 3.77–3.66 (m, 2H), 1.46–1.39 (m, 6H); ^{13}C NMR (101 MHz, $CDCl_3$) δ 144.50, 144.47, 144.4, 144.2, 144.14, 144.09, 142.6, 142.52, 142.50, 142.46, 142.32, 142.27, 142.25, 142.2, 136.84, 136.80, 136.7, 129.1, 129.0, 128.5, 128.4, 128.3, 128.2, 128.1, 128.0, 127.10, 127.07, 127.01, 126.99, 126.97, 126.9, 126.2, 124.4, 124.29, 124.26, 121.51, 121.49, 56.4, 56.3, 31.1, 31.0, 29.7, 21.44, 21.39, 21.3; HRMS (DART) *m*/*z* calcd for $C_{32}H_{29}N_2$ $[M+H]^+$: 441.2325 found 441.2321.

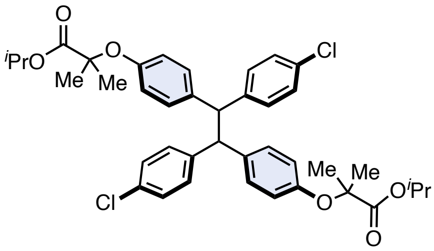

Diisopropyl 2,2'-(((1,2-bis(4-chlorophenyl)ethane-1,2-diyl)bis(4,1-phenylene))bis(oxy))bis(2-methylpropanoate) (3V)

The reaction was conducted by using 10 mol% of $PdCl_2$, 40 mol% of PPh_3 and 0.40 equiv of K_2CO_3. Purification by PTLC ($CHCl_3$) afforded **3V** as a beige solid (38.1 mg, 28% yield). The product was characterized by the mixture of diastereomers. ^{1}H NMR (400 MHz, $CDCl_3$) δ 7.09–6.95 (m, 8H), 6.95–6.88 (m, 4H), 6.65–6.54 (m, 4H), 5.04–4.94 (m, 2H), 4.52 (s, 2H), 1.503 (s, 6H), 1.497 (s, 6H), 1.10 (t, J = 6.4 Hz, 12H); ^{13}C NMR (101 MHz, $CDCl_3$) δ 173.7, 153.8, 141.9, 135.9, 131.5, 129.7, 128.9, 128.2, 118.5, 78.9, 68.8, 55.2, 25.3, 25.2, 21.41, 21.39.; HRMS (ESI) *m*/*z* calcd for $C_{40}H_{48}O_6NCl_2$ $[M+NH_4]^+$: 708.2853 found 708.2847.

4. Pd-Catalyzed Deoxygenative Synthesis of Triarylmethanes from Diarylketones

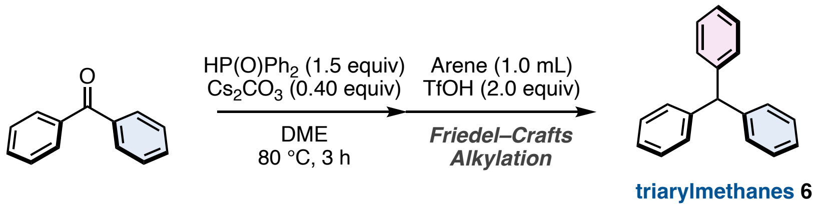

Friedel–Crafts Alkylation

A 20-mL glass vessel equipped with J. Young® O-ring tap containing a magnetic stirring bar and Cs_2CO_3 (52.1 mg, 0.16 mmol, 0.40 equiv) was dried with a heat-gun *in vacuo* and filled with N_2 gas after cooling to room temperature. To this were added diarylketones (0.40 mmol, 1.0 equiv) and diphenylphosphine oxide (121.3 mg, 0.60 mmol, 1.5 equiv). The vessel was placed under vacuum and refilled N_2 gas three times. To this vessel was added DME (2.0 mL). The vessel was sealed with an O-ring tap and then heated at 80 °C for 3 h in a 9-well reaction block with stirring. After cooling the reaction mixture to room temperature, the mixture was concentrated *in vacuo*. To the residue were added arene (1.0 mL) and TfOH (0.80 mmol, 2.0 equiv) at 0 °C. After stirring the mixture for several minutes with monitoring reaction progress with TLC, the reaction was quenched with saturated $NaHCO_3$ aq. The mixture was extracted three times with EtOAc. The combined organic layer was dried over Na_2SO_4, filtrated, and concentrated *in vacuo*. The residue was purified by PTLC to afford **6**.

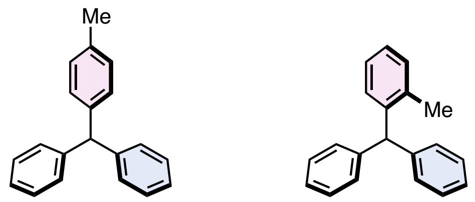

***p*-6A** ***o*-6A**

(*p*-Tolylmethylene)dibenzene (*p*-6A)[14]

Crude ^{1}H NMR analysis showed ***p*-6A**:***o*-6A** = 85:15; ^{1}H NMR peaks at 5.68 ppm (s, 0.17H) and 5.52 ppm (s, 1H) were used. Purification by PTLC (hexane/EtOAc = 4:1) afforded **6A** as a colorless liquid (67.6 mg, 65% yield, as a mixture of structural isomer; ***p*-6A**:***o*-6A** = 85:15).

For ***p*-6A**: ^{1}H NMR (400 MHz, $CDCl_3$) δ 7.32–7.22 (m, 4H), 7.22–7.14 (m, 2H), 7.14–7.03 (m, 6H), 7.03–6.96 (m, 2H), 5.50 (s, 1H), 2.30 (s, 3H). ^{13}C NMR (101 MHz, $CDCl_3$) δ 144.1, 140.9, 135.8, 129.4, 129.3, 129.0, 128.2, 126.2, 56.4, 21.0. The spectra matched with those of this compound reported in the literature.[14]

For ***o*-6A**[14]: ^{1}H NMR (400 MHz, $CDCl_3$) δ 7.32–7.03 (m, 13H), 6.85–6.79 (m, 1H), 5.68 (s, 1H), 2.22 (s, 3H).

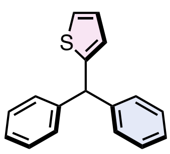

2-6B

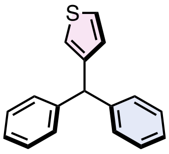

3-6B

2-Benzhydrylthiophene (2-6B)[15]

The reaction was conducted by using thiophene (0.50 mL). Crude ^{1}H NMR analysis showed **2-6B**:**3-6B** = 93:7; ^{1}H NMR peaks at 5.69 ppm (s, 1H) and 5.52 ppm (s, 0.07H) were used. Purification by PTLC (hexane/$CHCl_3$ = 9:1) afforded **6B** as a white solid (57.1 mg, 57% yield, as a mixture of structural isomer; **2-6B**/**3-6B** = 93:7).

For **2-6B**: ^{1}H NMR (400 MHz, $CDCl_3$) δ 7.40–7.27 (m, 4H), 7.25–7.19 (m, 7H), 6.97–6.92 (m, 1H), 6.72–6.67 (m, 1H), 5.69 (s, 1H); ^{13}C NMR (101 MHz, $CDCl_3$) δ 147.9, 143.8, 128.8, 128.4, 126.7, 126.6, 126.4, 124.5, 52.1. The spectra matched with those of this compound reported in the literature.[15]

For **3-6B**[14]: ^{1}H NMR (400 MHz, $CDCl_3$) δ 7.41–7.13 (m, 11H), 6.90–6.86 (m, 1H), 6.75–6.73 (m, 1H), 5.52 (s, 1H).

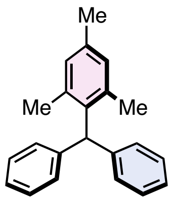

(Mesitylmethylene)dibenzene (6C)[16]

Purification by PTLC (hexane/EtOAc = 6:1) afforded **6C** as a colorless liquid (55.9 mg, 49% yield). ^{1}H NMR (400 MHz, $CDCl_3$) δ 7.30–7.22 (m, 4H), 7.22–7.16 (m, 2H), 7.13–7.07 (m, 4H), 6.86 (s, 2H), 6.00 (s, 1H), 2.29 (s, 3H), 2.01 (s, 6H); ^{13}C NMR (101 MHz, $CDCl_3$) δ 142.5, 137.6, 137.0, 136.0, 130.1, 129.3, 128.1, 125.9, 51.0, 22.0, 20.8. The spectra matched with those of this compound reported in the literature.[16]

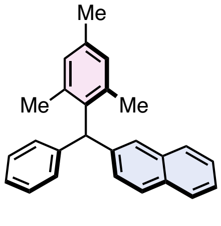

2-(Mesityl(phenyl)methyl)naphthalene (6D)

The reaction was conducted by using mesitylene (0.50 mL). Purification by PTLC (hexane/$CHCl_3$ = 9:1) afforded **6D** as a beige solid (62.0 mg, 46% yield). ^{1}H NMR (400 MHz, $CDCl_3$) δ 7.83–7.77 (m, 1H), 7.74 (d, J = 8.0 Hz, 1H), 7.69–7.64 (m, 1H), 7.45–7.38 (m, 3H), 7.32–7.18 (m, 5H), 7.13 (d, J = 8.0 Hz, 2H), 6.14 (s, 1H), 2.30 (s, 3H), 2.03 (s, 6H); ^{13}C NMR (101 MHz, $CDCl_3$) δ 142.4, 140.2, 137.7,

136.8, 136.1, 133.4, 132.0, 130.2, 129.5, 128.23, 128.19, 127.9, 127.6, 127.5, 127.4, 126.0, 125.8, 125.4, 51.3, 22.0, 20.8; HRMS (DART) *m*/*z* calcd for $C_{26}H_{28}N$ $[M+NH_4]^+$: 354.2216 found 354.2213.

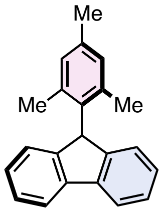

9-Mesityl-9*H*-fluorene (6E)

The reaction was conducted by using mesitylene (0.50 mL). Purification by PTLC (hexane/EtOAc = 4:1) afforded **6E** as a yellow solid (78.3 mg, 69% yield). ^{1}H NMR (400 MHz, $CDCl_3$) δ 7.83 (d, J = 7.6 Hz, 2H), 7.38 (t, J = 7.6 Hz, 2H), 7.25–7.19 (m, 3H), 7.02 (s, 1H), 6.65 (s, 1H), 5.48 (s, 1H), 2.67 (s, 3H), 2.28 (s, 3H), 1.08 (s, 3H); ^{13}C NMR (101 MHz, $CDCl_3$) δ 147.2, 140.9, 137.8, 137.7, 136.2, 133.8, 130.5, 128.8, 127.1, 126.8, 124.1, 120.0, 49.7, 21.7, 20.8, 18.6; HRMS (DART) *m*/*z* calcd for $C_{22}H_{24}N$ $[M+NH_4]^+$: 302.1903 found 302.1901.

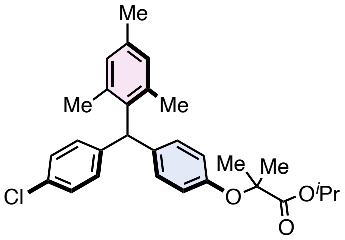

Isopropyl 2-(4-((4-chlorophenyl)(mesityl)methyl)phenoxy)-2-methylpropanoate (6F)

Purification by PTLC (hexane/EtOAc = 6:1) afforded **6F** as a colorless liquid (85.5 mg, 46% yield). ^{1}H NMR (400 MHz, $CDCl_3$) δ 7.21 (d, J = 8.8 Hz, 2H), 7.00 (d, J = 8.8 Hz, 2H), 6.91 (d, J = 8.8 Hz, 2H), 6.83 (s, 2H), 6.75 (d, J = 8.8 Hz, 2H), 5.86 (s, 1H), 5.12–5.01 (m, 1H), 2.27 (s, 3H), 1.96 (s, 6H), 1.57 (s, 6H), 1.21 (d, J = 6.4 Hz, 6H); ^{13}C NMR (101 MHz, $CDCl_3$) δ 173.7, 153.7, 141.3, 137.3, 136.7, 136.1, 135.3, 131.6, 130.5, 130.2, 129.7, 128.2, 118.8, 79.0, 68.8, 49.6, 25.4, 25.3, 21.9, 21.5, 20.7; HRMS (ESI) *m*/*z* calcd for $C_{29}H_{33}ClO_3Na$ $[M+Na]^+$: 487.2010 found 487.2011.

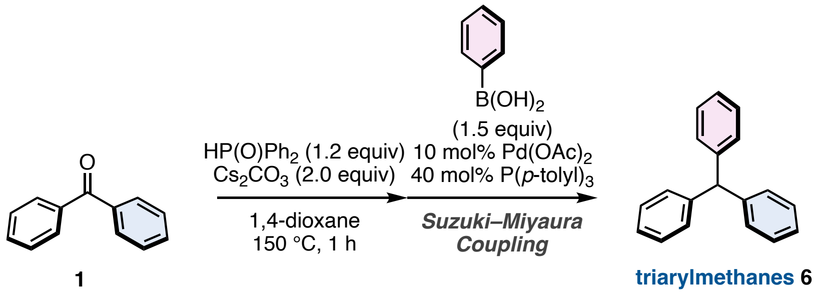

Suzuki–Miyaura Coupling

A 20-mL glass vessel equipped with J. Young® O-ring tap containing a magnetic stirring bar and Cs_2CO_3 (260.7 mg, 0.80 mmol, 2.0 equiv) was dried with a heat-gun *in vacuo* and filled with N_2 gas after cooling to room temperature. To this were added benzophenone (**1**: 72.9 mg, 0.40 mmol, 1.0 equiv) and diphenylphosphine oxide (97.1 mg, 4.8 mmol, 1.2 equiv). The vessel was placed under vacuum and refilled N_2 gas three times. To this vessel was added 1,4-dioxane (2.0 mL). The vessel was sealed with an O-ring tap and then heated at 150 °C for 1 h in a 9-well reaction block with stirring. After cooling the reaction mixture to room temperature, to this mixture were added $Pd(OAc)_2$ (9.0 mg, 0.040 mmol, 10 mol%), P(*p*-tolyl)$_3$ (48.7 mg, 0.16 mmol, 40 mol%) and arylboronic acid (6.0 mmol, 1.5 equiv) under a stream of N_2 gas. The vessel was sealed with an O-ring tap and then heated at 150 °C for 1 h in a 9-well reaction block with stirring. After cooling the reaction mixture to room temperature, the mixture was passed through a short silica-gel pad with EtOAc as an eluent. The filtrate was concentrated *in vacuo*. The residue was purified by PTLC or GPC to afford **6**.

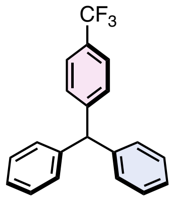

((4-(Trifluoromethyl)phenyl)methylene)dibenzene (6G)[14]

Purification by PTLC (hexane/EtOAc = 4:1) and then GPC to afford **6G** as a white solid (50.8 mg, 41% yield). ^{1}H NMR (400 MHz, $CDCl_3$) δ 7.53 (d, J = 8.0 Hz, 2H), 7.34–7.26 (m, 4H), 7.26–7.18 (m, 4H), 7.13–7.05 (m, 4H), 5.59 (s, 1H); ^{13}C NMR (151 MHz, $CDCl_3$) δ 148.0, 142.9, 129.8, 129.4, 128.6 (q, J_{C-F} = 32.3 Hz), 128.5, 126.7, 125.2 (q, J_{C-F} = 3.9 Hz), 124.3 (q, J_{C-F} = 272 Hz), 56.6; ^{19}F NMR (376 MHz, $CDCl_3$) δ –62.5. The spectra matched with those of this compound reported in the literature.[14]

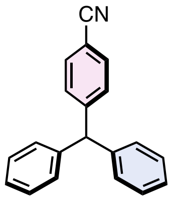

4-Benzhydrylbenzonitrile (6H)[17]

Purification by PTLC (hexane/Et_2O = 10:1) and then GPC to afford **6H** as a colorless liquid (37.8 mg, 35% yield). ^{1}H NMR (400 MHz, $CDCl_3$) δ 7.57 (d, J = 8.0 Hz, 2H), 7.31 (t, J = 8.0 Hz, 4H), 7.27–7.20 (m, 4H), 7.07 (d, J = 8.0 Hz, 4H), 5.58 (s, 1H); ^{13}C NMR (101 MHz, $CDCl_3$) δ 149.5, 142.4, 132.1, 130.2, 129.3, 128.6, 126.8, 118.9, 110.2, 56.8. The spectra matched with those of this compound reported in the literature.[17]

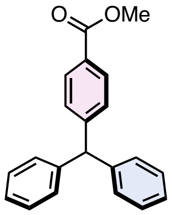

Methyl 4-benzhydrylbenzoate (6I)

Purification by PTLC (hexane/EtOAc = 4:1) and then GPC to afford **6I** as a white solid (44.5 mg, 37% yield). ^{1}H NMR (400 MHz, $CDCl_3$) δ 7.99–7.93 (m, 2H), 7.32–7.25 (m, 4H), 7.25–7.17 (m, 4H), 7.14–7.07 (m, 4H), 5.59 (s, 1H), 3.89 (s, 3H); ^{13}C NMR (101 MHz, $CDCl_3$) δ 167.0, 149.2, 143.0, 129.6, 129.5, 129.4, 128.4, 128.3, 126.6, 56.8, 52.0; HRMS (ESI) *m*/*z* calcd for $C_{21}H_{19}O_2$ $[M+H]^+$: 303.1380 found 303.1379.

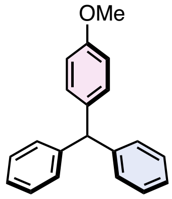

((4-Methoxyphenyl)methylene)dibenzene (6J)[14]

Purification by PTLC (hexane/EtOAc = 4:1) and then GPC to afford **6J** as a colorless liquid (53.8 mg, 49% yield). ^{1}H NMR (400 MHz, $CDCl_3$) δ 7.34–7.23 (m, 4H), 7.23–7.16 (m, 2H), 7.11 (d, J = 7.6 Hz, 4H), 7.06–6.99 (m, 2H), 6.85–6.79 (m, 2H), 5.50 (s, 1H), 3.76 (s, 3H); ^{13}C NMR (101 MHz, $CDCl_3$) δ 158.0, 144.2, 136.1, 130.3, 129.4, 128.2, 126.2, 113.6, 56.0, 55.2. The spectra matched with those of this compound reported in the literature.[14]

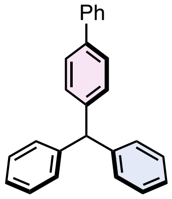

4-Benzhydryl-1,1'-biphenyl (6K)[18]

Purification by PTLC (hexane/EtOAc = 4:1) and then GPC to afford **6K** as a white solid (56.1 mg, 44% yield). ^{1}H NMR (400 MHz, $CDCl_3$) δ 7.60–7.53 (m, 2H), 7.53–7.47 (m, 2H), 7.41 (t, J = 8.0 Hz, 2H), 7.34–7.26 (m, 5H), 7.24–7.18 (m, 3H), 7.18–7.13 (m, 5H), 5.58 (s, 1H); ^{13}C NMR (101 MHz, $CDCl_3$) δ 143.8, 143.0, 140.8, 139.1, 129.8, 129.4, 128.7, 128.3, 127.1, 127.0, 126.4, 56.5 (one peak is missing due to overlapping). The spectra matched with those of this compound reported in the literature.[18]

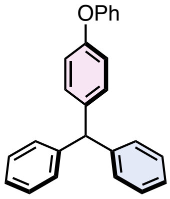

((4-Phenoxyphenyl)methylene)dibenzene (6L)

Purification by PTLC (hexane/$CHCl_3$/MeOH = 4:1:1) and then GPC to afford **6L** as a white solid (44.2 mg, 33% yield). ^1H NMR (400 MHz, $CDCl_3$) δ 7.35–7.16 (m, 8H), 7.16–7.09 (m, 4H), 7.09–7.02 (m, 3H), 7.03–6.95 (m, 2H), 6.95–6.86 (m, 2H), 5.53 (s, 1H); ^{13}C NMR (101 MHz, $CDCl_3$) δ 157.2, 155.6, 143.9, 138.7, 130.6, 129.7, 129.4, 128.3, 126.3, 123.2, 118.8, 118.6, 56.1; HRMS (DART) *m*/*z* calcd for $C_{25}H_{24}ON$ $[M+NH_4]^+$: 354.1852 found 354.1851.

5. One-Pot Synthesis of Diphenylmethane (2A)

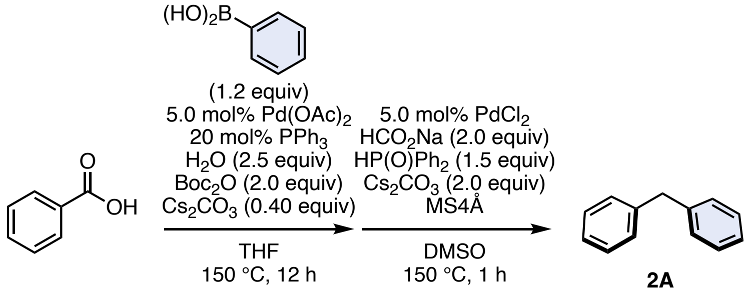

2A

A 20-mL glass vessel equipped with J. Young® O-ring tap containing a magnetic stirring bar and Cs_2CO_3 (52.1 mg, 0.16 mmol, 0.40 equiv) was dried with a heat-gun *in vacuo* and filled with N_2 gas after cooling to room temperature. To this were added benzoic acid (0.40 mmol, 1.0 equiv), $Pd(OAc)_2$ (4.5 mg, 0.020 mmol, 5.0 mol%), PPh_3 (21.0 mg, 0.080 mmol, 20 mol%), and phenylboronic acid (58.5 mg, 0.48 mmol, 1.2 equiv). The vessel was placed under vacuum and refilled N_2 gas three times. To this vessel were added Boc_2O (183 µL, 0.80 mmol, 2.0 equiv), water (18 µL, 1.0 mmol, 2.5 equiv), and THF (1.6 mL). The vessel was sealed with an O-ring tap and then heated at 150 °C for 12 h in a 9-well reaction block with stirring. After cooling the reaction mixture to room temperature, the mixture was concentrated *in vacuo*. The vessel was filled with N_2 gas. To the same vessel were added $PdCl_2$ (3.6 mg, 0.020 mmol, 5.0 mol%), HCO_2Na (54.4 mg, 0.80 mmol, 2.0 equiv), diphenylphosphine oxide (121.3 mg, 0.60 mmol, 1.5 equiv), dried Cs_2CO_3 (260.7 mg, 0.80 mmol, 2.0 equiv), and MS4Å (100 mg). The vessel was placed under vacuum and refilled N_2 gas three times. To this vessel was added DMSO (2.0 mL). The vessel was sealed with an O-ring tap and then heated at 150 °C for 1 h in a 9-well reaction block with stirring. After cooling the reaction mixture to room temperature, the mixture was added brine and then extracted three times with hexane/EtOAc. The combined organic layer was dried over Na_2SO_4, filtrated, and then concentrated *in vacuo*. The yield of **2A** was estimated as 70% according to the ^1H NMR analysis of crude mixture by using CH_2Br_2 as an internal standard.

6. One-Pot Synthesis of 1,1,2,2-Tetraphenylethane (3A)

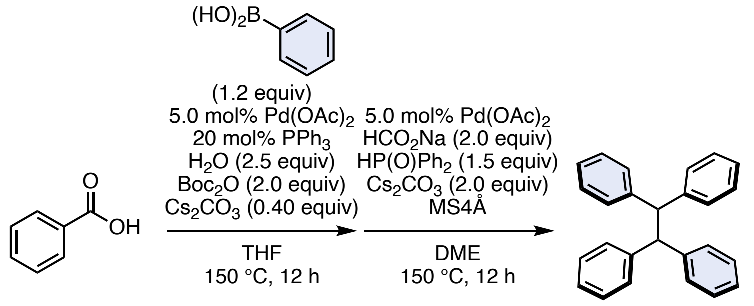

3A

A 20-mL glass vessel equipped with J. Young® O-ring tap containing a magnetic stirring bar and Cs_2CO_3 (52.1 mg, 0.16 mmol, 0.40 equiv) was dried with a heat-gun *in vacuo* and filled with N_2 gas after cooling to room temperature. To this were added benzoic acid (0.40 mmol, 1.0 equiv), $Pd(OAc)_2$ (4.5 mg, 0.020 mmol, 5.0 mol%), PPh_3 (21.0 mg, 0.080 mmol, 20 mol%), and phenylboronic acid (58.5

mg, 0.48 mmol, 1.2 equiv). The vessel was placed under vacuum and refilled N_2 gas three times. To this vessel were added Boc_2O (183 µL, 0.80 mmol, 2.0 equiv), water (18 µL, 1.0 mmol, 2.5 equiv), and THF (1.6 mL). The vessel was sealed with an O-ring tap and then heated at 150 °C for 12 h in a 9-well reaction block with stirring. After cooling the reaction mixture to room temperature, the mixture was concentrated *in vacuo*. The vessel was filled with N_2 gas. To the same vessel were added $Pd(OAc)_2$ (4.5 mg, 0.020 mmol, 5.0 mol%), HCO_2Na (54.4 mg, 0.80 mmol, 2.0 equiv), diphenylphosphine oxide (121.3 mg, 0.60 mmol, 1.5 equiv), dried Cs_2CO_3 (260.7 mg, 0.80 mmol, 2.0 equiv), and MS4Å (100 mg). The vessel was placed under vacuum and refilled N_2 gas three times. To this vessel was added DME (2.0 mL). The vessel was sealed with an O-ring tap and then heated at 150 °C for 12 h in a 9-well reaction block with stirring. After cooling the reaction mixture to room temperature, the mixture was passed through a short silica-gel pad with EtOAc as an eluent. The filtrate was concentrated *in vacuo*. The residue was purified by PTLC (hexane/EtOAc = 4:1) to afford 1,1,2,2-tetraphenylethane (**3A**) as a white solid (28.5 mg, 43% yield).

7. One-Pot Synthesis of 2-(1-(*p*-Tolyl)but-3-en-1-yl)naphthalene (8)[19]

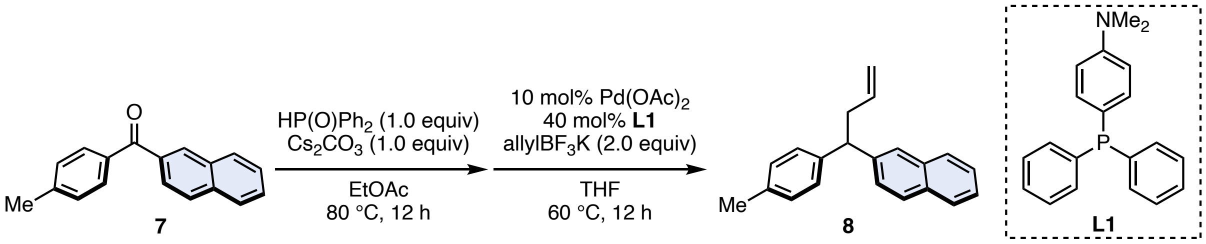

An 8-mL glass tube equipped with a screw cap containing a magnetic stirring bar and Cs_2CO_3 (65.2 mg, 0.20 mmol, 1.0 equiv) was dried with a heat-gun *in vacuo* and filled N_2 after cooling to room temperature. To this tube were added naphthalen-2-yl(*p*-tolyl)methanone (**7**: 49.3 mg, 0.20 mmol, 1.0 equiv) and diphenylphosphine oxide (40.4 mg, 0.20 mmol, 1.0 equiv). The tube was placed under vacuum and refilled N_2 gas three times. To this tube was added EtOAc (1.0 mL). The vessel was sealed with a screw cap and then heated at 80 °C for 12 h in a 16-well reaction block with stirring. After cooling the reaction mixture to room temperature, the mixture was refilled N_2 gas. To the same vessel were added $Pd(OAc)_2$ (4.5 mg, 0.020 mmol, 10 mol%), **L1** (24.4 mg, 0.080 mmol, 40 mol%), potassium allyltrifluoroborate (59.2 mg, 0.40 mmol, 2.0 equiv), and THF (1.0 mL) under a stream of N_2 gas. The vessel was sealed with a screw cap and then heated at 60 °C for 12 h in a 16-well reaction block with stirring. After cooling the reaction mixture to room temperature, the mixture was passed through a short silica-gel pad with EtOAc as an eluent. The filtrate was concentrated *in vacuo*. The residue was purified by PTLC (hexane/EtOAc = 9:1) to afford 2-(1-(*p*-tolyl)but-3-en-1-yl)naphthalene (**8**) as a colorless liquid (28.2 mg, 52% yield). ^{1}H NMR (400 MHz, $CDCl_3$) δ 7.81–7.75 (m, 2H), 7.73 (d, J = 8.4 Hz, 1H), 7.69 (s, 1H), 7.47–7.36 (m, 2H), 7.33 (d, J = 8.4 Hz, 1H), 7.17 (d, J = 8.0 Hz, 2H), 7.09 (d, J = 8.0 Hz, 2H), 5.83–5.67 (m, 1H), 5.11–4.91 (m, 2H), 4.14 (t, J = 7.6 Hz, 1H), 2.97–2.81 (m, 2H), 2.30 (s, 3H); ^{13}C NMR (101 MHz, $CDCl_3$) δ 142.2, 141.4, 136.9, 135.7, 133.5, 132.1, 129.1, 128.0, 127.9, 127.7,

127.5, 126.8, 125.9, 125.3, 116.3, 50.8, 39.8, 21.0 (one peak is missing due to overlapping). The spectra matched with those of this compound reported in the literature.[19]

8. One-Pot Synthesis of 2-(4-Allylbenzyl)naphthalene (10)

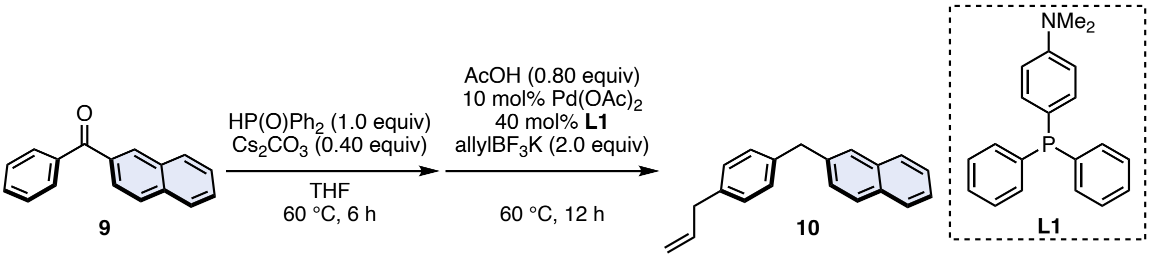

An 8-mL glass tube equipped with a screw cap containing a magnetic stirring bar and Cs_2CO_3 (26.1 mg, 0.080 mmol, 0.40 equiv) was dried with a heat-gun *in vacuo* and filled N_2 after cooling to room temperature. To this tube were added naphthalen-2-yl(phenyl)methanone (**9**: 46.5 mg, 0.20 mmol, 1.0 equiv) and diphenylphosphine oxide (40.4 mg, 0.20 mmol, 1.0 equiv). The tube was placed under vacuum and refilled N_2 gas three times. To this tube was added THF (2.0 mL). The vessel was sealed with a screw cap and then heated at 60 °C for 6 h in a 16-well reaction block with stirring. After cooling the reaction mixture to room temperature, the mixture was refilled N_2 gas. To the same vessel were added acetic acid (AcOH: 9.2 μL, 0.16 mmol, 0.80 equiv), $Pd(OAc)_2$ (4.5 mg, 0.020 mmol, 10 mol%), **L1** (24.4 mg, 0.080 mmol, 40 mol%), and potassium allyltrifluoroborate (59.2 mg, 0.40 mmol, 2.0 equiv) under a stream of N_2 gas. The vessel was sealed with a screw cap and then heated at 60 °C for 12 h in a 16-well reaction block with stirring. After cooling the reaction mixture to room temperature, the reaction was quenched with H_2O. The mixture was extracted three times with Et_2O. The combined organic layer was dried over Na_2SO_4, filtrated, and concentrated *in vacuo*. The residue was purified by PTLC (hexane) to afford 2-(4-allylbenzyl)naphthalene (**10**) as a white solid (36.9 mg, 71% yield). ^{1}H NMR (400 MHz, $CDCl_3$) δ 7.80–7.71 (m, 3H), 7.62 (s, 1H), 7.47–7.36 (m, 2H), 7.33–7.25 (m, 1H), 7.15 (d, J = 8.0 Hz, 2H), 7.10 (d, J = 8.0 Hz, 2H), 6.01–5.87 (m, 1H), 5.11–4.99 (m, 2H), 4.10 (s, 2H), 3.35 (d, J = 6.4 Hz, 2H); ^{13}C NMR (101 MHz, $CDCl_3$) δ 138.73, 138.69, 137.9, 137.5, 133.6, 132.0, 129.0, 128.7, 128.0, 127.62, 127.59, 127.5, 127.0, 125.9, 125.3, 115.7, 41.7, 39.8; HRMS (DART) m/z calcd for $C_{20}H_{22}N$ $[M+NH_4]^+$: 276.1747 found 276.1745.

9. Intramolecular Dimerization/ Oxidation

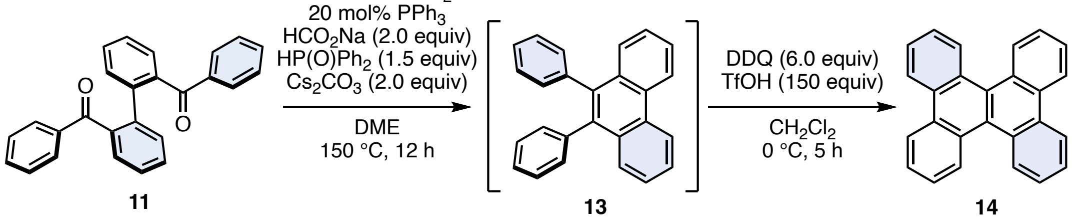

A 20-mL glass vessel equipped with J. Young® O-ring tap containing a magnetic stirring bar and Cs_2CO_3 (130.3 mg, 0.40 mmol, 2.0 equiv) was dried with a heat-gun *in vacuo* and filled with N_2 gas after cooling to room temperature. To this were added [1,1'-biphenyl]-2,2'-diylbis(phenylmethanone) (**11**: 0.20 mmol, 1.0 equiv), $PdCl_2$ (1.8 mg, 0.010 mmol, 5.0 mol%), PPh_3 (10.5 mg, 0.040 mmol, 20 mol%), HCO_2Na (27.2 mg, 0.40 mmol, 2.0 equiv), and diphenylphosphine oxide (60.7 mg, 0.30 mmol, 1.5 equiv). The vessel was placed under vacuum and refilled N_2 gas three times. To this vessel was added DME (1.0 mL). The vessel was sealed with an O-ring tap and then heated at 150 °C for 12 h in a 9-well reaction block with stirring. After cooling the reaction mixture to room temperature, the mixture was concentrated *in vacuo*. To the same vessel were added 2,3-dichloro-5,6-dicyano-1,4-benzoquinone (DDQ: 136.2 mg, 0.60 mmol, 6.0 equiv), CH_2Cl_2 (10 mL), and then TfOH (2.7 mL, 30 mmol, 150 equiv) at 0 °C. After stirring the mixture at 0 °C for 5 h, the reaction was quenched with $NaHCO_3$ aq. The mixture was extracted three times with CH_2Cl_2. The combined organic layer was washed with water and brine, dried over $MgSO_4$, filtrated, and concentrated *in vacuo*. The residue was purified by PTLC (hexane/$CHCl_3$ = 9:1) to afford dibenzo[*g*,*p*]chrysene (**14**) as a white solid (25.1 mg, 38% yield). The spectra of **14** were matched with those of the commercial reagent from TCI (D3736).

10. 2-Benzylnaphthalene (2B) Synthesis from Arylaldehyde

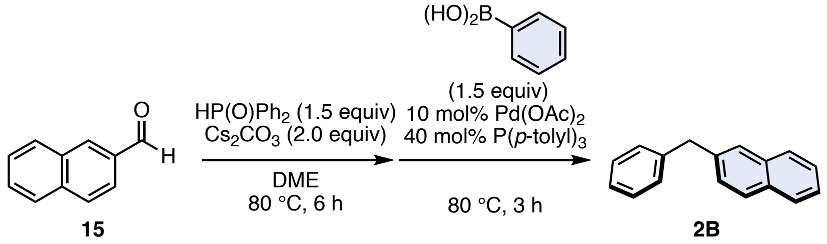

A 20-mL glass vessel equipped with J. Young® O-ring tap containing a magnetic stirring bar and Cs_2CO_3 (260.7 mg, 0.80 mmol, 2.0 equiv) was dried with a heat-gun *in vacuo* and filled with N_2 gas after cooling to room temperature. To this were added 2-naphthaldehyde (**15**: 62.5 mg, 0.40 mmol, 1.0 equiv) and diphenylphosphine oxide (121.3 mg, 0.60 mmol, 1.5 equiv). The vessel was placed under vacuum and refilled N_2 gas three times. To this vessel was added DME (2.0 mL). The vessel was sealed with an O-ring tap and then heated at 80 °C for 6 h in a 9-well reaction block with stirring. After cooling the reaction mixture to room temperature, the mixture was refilled N_2 gas. To the same vessel were added phenylboronic acid (73.2 mg, 0.60 mmol, 1.5 equiv), $Pd(OAc)_2$ (9.0 mg, 0.040 mmol, 10 mol%) and P(*p*-tolyl)$_3$ (48.7 mg, 0.16 mmol, 40 mol%) under a stream of N_2 gas. The vessel was sealed with an O-ring tap and then heated at 80 °C for 3 h in a 9-well reaction block with stirring. After cooling the reaction mixture to room temperature, the mixture was passed through a short silica-gel pad with EtOAc as an eluent. The filtrate was concentrated *in vacuo*. The residue was purified by PTLC (hexane/EtOAc = 9:1) to afford 2-benzylnaphthalene (**2B**) as a colorless liquid (50.9 mg, 58% yield).

11. 2-(1,2-Diphenylethyl)naphthalene (16) Synthesis from Diarylketone[20]

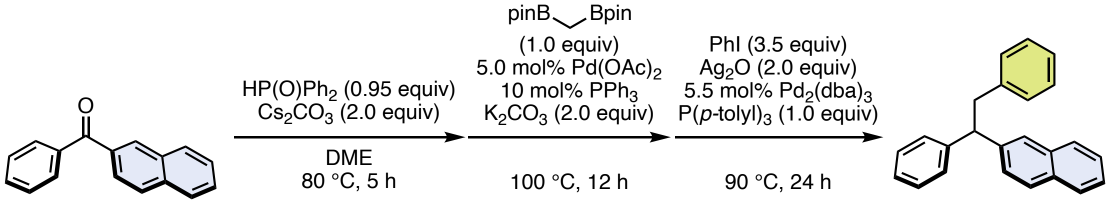

A 20-mL glass vessel equipped with J. Young® O-ring tap containing a magnetic stirring bar and Cs_2CO_3 (130.3 mg, 0.40 mmol, 2.0 equiv) was dried with a heat-gun *in vacuo* and filled with N_2 gas after cooling to room temperature. To this was added naphthalen-2-yl(phenyl)methanone (**9**: 46.5 mg, 0.20 mmol, 1.0 equiv) and diphenylphosphine oxide (38.4 mg, 0.19 mmol, 0.95 equiv). The vessel was placed under vacuum and refilled N_2 gas three times. To this vessel was added DME (2.0 mL). The vessel was sealed with an O-ring tap and then heated at 80 °C for 5 h in a 9-well reaction block with stirring. After cooling the reaction mixture to room temperature, the vessel was refilled N_2 gas. To the same vessel were added bis(4,4,5,5-tetramethyl-1,3,2-dioxaborolan-2-yl)methane (53.6 mg, 0.20 mmol, 1.0 equiv), $Pd(OAc)_2$ (2.3 mg, 0.010 mmol, 5.0 mol%), PPh_3 (5.3 mg, 0.020 mmol, 10 mol%), and dried K_2CO_3 (55.3 mg, 0.40 mmol, 2.0 equiv) under a stream of N_2 gas. The vessel was sealed with an O-ring tap and then heated at 100 °C for 12 h in a 9-well reaction block with stirring. After cooling the reaction mixture to room temperature, the vessel was refilled N_2 gas. To the same vessel were added $Pd_2(dba)_3{\cdot}CHCl_3$ (11.4 mg, 0.011 mmol, 5.5 mol%), P(*p*-tolyl)$_3$ (60.9 mg, 0.20 mmol, 1.0 equiv), Ag_2O (92.7 mg, 0.40 mmol, 2.0 equiv), and iodobenzene (78 μL, 0.70 mmol, 3.5 equiv). The vessel was sealed with an O-ring tap and then heated at 90 °C for 24 h in a 9-well reaction block with stirring. After cooling the reaction mixture to room temperature, the mixture was passed through a short silica-gel pad with EtOAc as an eluent. The filtrate was concentrated *in vacuo*. The residue was purified by PTLC (hexane/EtOAc = 6:1) to afford 2-(1,2-diphenylethyl)naphthalene (**16**) as a white solid (22.9 mg, 37% yield). ^{1}H NMR (400 MHz, $CDCl_3$) δ 7.79–7.73 (m, 2H), 7.72 (d, *J* = 8.4 Hz, 1H), 7.66 (s, 1H), 7.46–7.38 (m, 2H), 7.33 (dd, *J* = 8.4, 2.0 Hz, 1H), 7.25–7.21 (m, 4H), 7.20–7.07 (m, 4H), 7.06–6.99 (m, 2H), 4.40 (t, *J* = 7.6 Hz, 1H), 3.54–3.39 (m, 2H); ^{13}C NMR (101 MHz, $CDCl_3$) δ 144.3, 141.9, 140.2, 133.4, 132.1, 129.1, 128.4, 128.2, 128.1, 128.0, 127.7, 127.5, 126.9, 126.2, 126.1, 125.90, 125.89, 125.4, 53.1, 41.9. The spectra matched with those of this compound reported in the literature.[20]

12. 2-Tritylbenzofuran (18) Synthesis from Benzophenone (1)

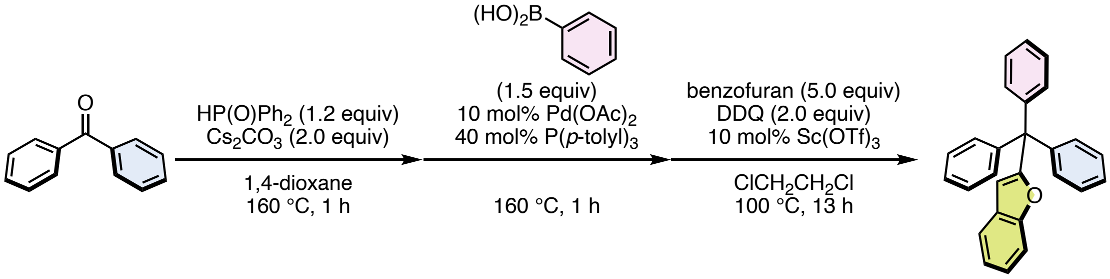

1 **18**

A 20-mL glass vessel equipped with J. Young® O-ring tap containing a magnetic stirring bar and Cs_2CO_3 (260.6 mg, 0.80 mmol, 2.0 equiv) was dried with a heat-gun *in vacuo* and filled with N_2 gas after cooling to room temperature. To this were added benzophenone (**1**: 72.8 mg, 0.40 mmol, 1.0 equiv) and diphenylphosphine oxide (97.1 mg, 0.48 mmol, 1.2 equiv). The vessel was placed under vacuum and refilled N_2 gas three times. To this vessel was added 1,4-dioxane (2.0 mL). The vessel was sealed with an O-ring tap and then heated at 160 °C for 1 h in a 9-well reaction block with stirring. After cooling the reaction mixture to room temperature, the vessel was refilled N_2 gas. To the same vessel were added $Pd(OAc)_2$ (9.0 mg, 0.040 mmol, 10 mol%), $P(p\text{-tolyl})_3$ (48.7 mg, 0.16 mmol, 40 mol%), and phenylboronic acid (73.2 mg, 0.60 mmol, 1.5 equiv) under a stream of N_2 gas. The vessel was sealed with an O-ring tap and then heated at 160 °C for 1 h in a 9-well reaction block with stirring. After cooling the reaction mixture to room temperature, the mixture was passed through a pad of Celite® with EtOAc as an eluent. The filtrate was concentrated *in vacuo*. The tetraarylmethane synthesis follows Nambo's procedure. To an 8-mL glass tube equipped with a screw cap containing a magnetic stirring bar were added the obtained mixture and $Sc(OTf)_3$ (19.7 mg, 0.040 mmol, 10 mol%). The tube was placed under vacuum and refilled N_2 gas three times. To this mixture were added $ClCH_2CH_2Cl$ (1.2 mL), benzofuran (215 µL, 2.0 mmol, 5.0 equiv), and 4,5-dichloro-3,6-dioxocyclohexa-1,4-diene-1,2-dicarbonitrile (DDQ: 181.6 mg, 0.80 mmol, 2.0 equiv). The tube was sealed with a screw cap and then heated at 100 °C for 13 h in a 16-well aluminum reaction block with stirring After cooling the reaction mixture to room temperature, the mixture was passed through a pad of Celite® with EtOAc as an eluent. The filtrate was concentrated *in vacuo*. The residue was purified by PTLC (hexane/EtOAc = 9:1) to afford 2-tritylbenzofuran (**18**) as a white solid (85.6 mg, 59% yield). ^{1}H NMR (400 MHz, $CDCl_3$) δ 7.48 (d, J = 8.0 Hz, 1H), 7.43 (d, J = 8.0 Hz, 1H), 7.33–7.25 (m, 9H), 7.23–7.20 (m, 1H), 7.20–7.13 (m, 7H), 6.47 (s, 1H); ^{13}C NMR (101 MHz, $CDCl_3$) 162.3, 155.1, 144.6, 130.4, 128.0, 127.8, 126.8, 123.9, 122.7, 120.9, 111.4, 108.4, 61.4. The spectra matched with those of this compound reported in the literature.[21]

13. Effect of Parameters

13-1. Variations from Standard Conditions

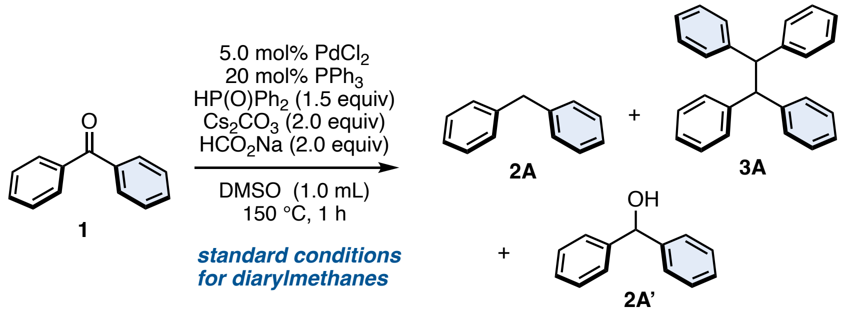

entry	variations from 'standard' conditions	recovery of **1**/ %	yield of **2A**/ %	yield of **3A**/ %	yield of **2A'**/ %
1	10 mol% dcype, w/o Cs_2CO_3, DME, 12 h	0	10	4	0
2	none	0	93	0	0
3	w/o $PdCl_2$	35	0	0	44
4	w/o PPh_3	14	56	0	15
5	w/o $HP(O)Ph_2$	86	0	0	12
6	w/o Cs_2CO_3	27	31	12	28
7	w/o HCO_2Na	64	13	4	14
8	100 °C	4	83	0	0
9	Cs_2CO_3 (0.40 equiv), DME	35	36	0	4
10	20 mol% $AsPh_3$, Cs_2CO_3 (0.40 equiv), DME	4	19	35	13
11	20 mol% XPhos, Cs_2CO_3 (0.40 equiv), DME	0	19	31	0
12	$HP(O)Ph_2$ (1.0 equiv), Cs_2CO_3 (0.40 equiv), DME	20	18	51	0
13	$HP(O)Ph_2$ (2.0 equiv), Cs_2CO_3 (0.40 equiv), DME	0	40	44	2
14	HCO_2Na (0.50 equiv), Cs_2CO_3 (0.40 equiv), DME	42	4	17	0
15	HCO_2Cs (2.0 equiv), Cs_2CO_3 (0.40 equiv), DME	0	53	46	0
16	Cs_2CO_3 (0.40 equiv), DME, 12 h	0	13	76	0
17	K_2CO_3 (0.40 equiv), DME, 12 h	5	18	54	23
18	CsF (0.40 equiv), DME, 12 h	24	11	28	28
19	5.0 mol% $Pd(OAc)_2$, Cs_2CO_3 (0.40 equiv), DME, 12 h	0	6	67	27
20	5.0 mol% $Pd(PPh_3)_4$, w/o PPh_3, Cs_2CO_3 (0.40 equiv), DME, 12 h	0	18	69	13
21	5.0 mol% $[Pd(allyl)Cl]_2$, Cs_2CO_3 (0.40 equiv), DME, 12 h	5	8	26	65
22	Cs_2CO_3 (0.40 equiv), MeCN, 12 h	0	43	50	7
23	Cs_2CO_3 (0.40 equiv), toluene, 12 h	0	34	60	0
24	Cs_2CO_3 (0.40 equiv), DMF, 12 h	2	47	31	19
25	Cs_2CO_3 (0.40 equiv), ^{t}AmylOH, 12 h	0	24	36	19

Recoveries and yields were determined by ^{1}H NMR using CH_2Br_2 as an internal standard.

13-2. Variations from Standard Conditions of Phosphinate

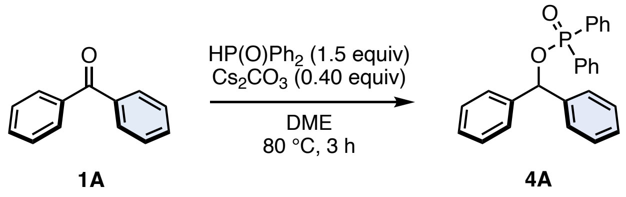

entry	variations from 'standard' conditions	recovery of **1A**/ %	yield of **4A**/ %
1		0	**quant.**
2	Cs_2CO_3 (2.0 equiv), EtOAc	0	87
3	DMSO	0	93
4	toluene	85	15
5	5.0 mol% $Pd(OAc)_2$	10	89
6	20 mol% PPh_3	0	91
7	5.0 mol% $Pd(OAc)_2$, 20 mol% PPh_3	28	72
8	HCO_2Na (2.0 equiv), 150 °C	0	80
9	H_2O (2.5 equiv)	60	35

Recoveries and yields were determined by ^{1}H NMR using CH_2Br_2 as an internal standard.

14. Mechanistic Study

14-1. Phosphinate 4A from Benzophenone 1

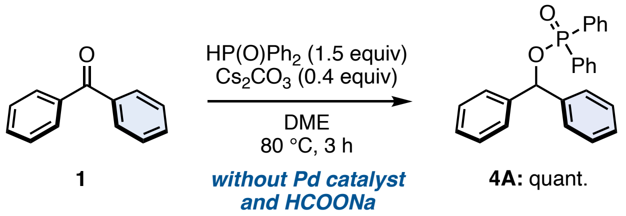

A 20-mL glass vessel equipped with J. Young® O-ring tap containing a magnetic stirring bar and Cs_2CO_3 (26.3 mg, 0.080 mmol, 0.40 equiv) was dried with a heat-gun *in vacuo* and filled with N_2 gas after cooling to room temperature. To this were added benzophenone (**1**: 36.0 mg, 0.20 mmol, 1.0 equiv) and diphenylphosphine oxide (60.7 mg, 0.30 mmol, 1.5 equiv). The vessel was placed under vacuum and refilled N_2 gas three times. To this vessel was added DME (1.0 mL). The vessel was sealed with an O-ring tap and then heated at 80 °C for 3 h in a 9-well reaction block with stirring. After cooling the reaction mixture to room temperature, the mixture was passed through a pad of Celite® with EtOAc as an eluent. The filtrate was concentrated *in vacuo*. The yield of benzhydryl diphenylphosphinate (**4A**) was determined by crude ^{1}H NMR analysis as >99% using CH_2Br_2 as an internal standard. The spectra of **4A** matched with those of this compound reported in the literature.[22]

14-2. Crossover Reaction

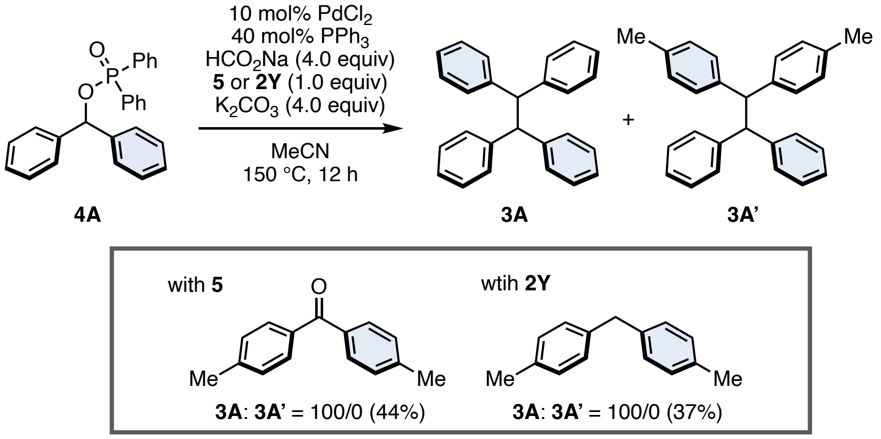

A 20-mL glass vessel equipped with J. Young® O-ring tap containing a magnetic stirring bar and K_2CO_3 (110.6 mg, 0.80 mmol, 4.0 equiv) was dried with a heat-gun *in vacuo* and filled with N_2 gas after cooling to room temperature. To this were added benzhydryl diphenylphosphinate (**4A**: 76.5 mg, 0.20 mmol, 1.0 equiv), $PdCl_2$ (3.1 mg, 0.020 mmol, 10 mol%), PPh_3 (21.6 mg, 0.080 mmol, 40 mol%), HCO_2Na (54.4 mg, 0.80 mmol, 4.0 equiv), and **5** (42.7 mg, 0.20 mmol, 1.0 equiv) or **2Y** (32.5 mg, 0.20 mmol, 1.0 equiv). The vessel was placed under vacuum and refilled N_2 gas three times. To this vessel was added MeCN (1.0 mL). The vessel was sealed with an O-ring tap and then heated at 150 °C for 12 h in a 9-well reaction block with stirring. After cooling the reaction mixture to room temperature, the mixture was passed through a short silica-gel pad with EtOAc as an eluent. The filtrate was concentrated

in vacuo. The yields of **3A** and **3A'** were determined by crude ^{1}H NMR analysis (using CH_2Br_2 as an internal standard) as 44% and 0%, respectively, when **5** was added. In the case of **2Y**, the yields of **3A** and **3A'** were determined as 37% and 0%, respectively.

14-3. Deuterium Label Experiments

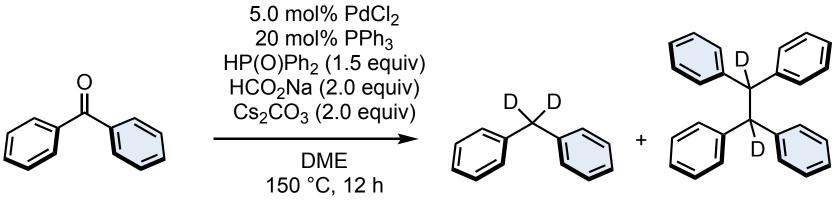

1 *d*-**2A** *d*-**3A**

	d-2A	*d*-3A
**with DCO_2Na** instead of HCO_2Na	25%D (22% yield)	7%D (57% yield)
**with $DP(O)Ph_2$** instead of $HP(O)Ph_2$	78%D (18% yield)	62%D (63% yield)
**with $DP(O)Ph_2/DCO_2Na$** instead of $HP(O)Ph_2/HCO_2Na$	**97%D** (15% yield)	**74%D** (66% yield)

Following the General Procedure for the synthesis of tetraarylethanes from diarylketones (page S10), the deoxygenative reaction of **1** was conducted with following modification.

1) When using DCO_2Na instead of HCO_2Na, *d*-**2A** (1.8 mg, 3% yield, 30%D) and *d*-**3A** (36.4 mg, 54% yield, 9%D) were isolated.

2) When using $DP(O)Ph_2$ instead of $HP(O)Ph_2$, *d*-**2A** (2.3 mg, 3% yield, 77%D) and *d*-**3A** (41.9 mg, 62% yield, 61%D) were isolated.

3) When using DCO_2Na and $DP(O)Ph_2$ instead of HCO_2Na and $HP(O)Ph_2$, *d*-**2A** (1.8 mg, 3% yield, 84%D) and *d*-**3A** (22.0 mg, 32% yield, 73%D) were isolated.

Reaction of Deuterated Compound S3

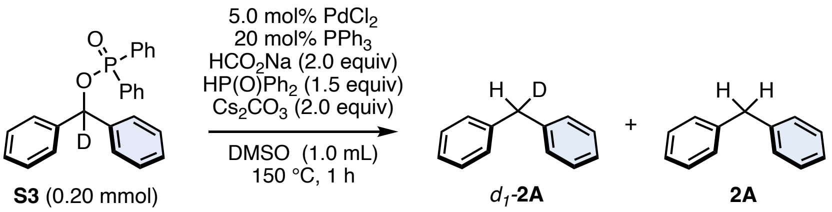

S3 (0.20 mmol) d_1-**2A** **2A**

d_1-**2A**+**2A** (71(54)%)
d_1-**2A**:**2A** = 85:15

14-4. Deoxygenative Reaction of 1 in the Presence of TEMPO.

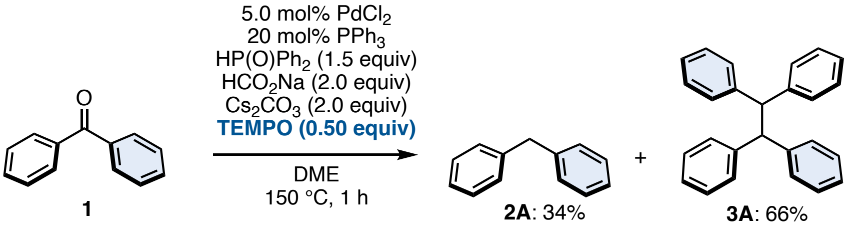

1 **2A**: 34% **3A**: 66%

A 20-mL glass vessel equipped with J. Young® O-ring tap containing a magnetic stirring bar and Cs_2CO_3 (26.1 mg, 0.080 mmol, 0.40 equiv) was dried with a heat-gun *in vacuo* and filled with N_2 gas after cooling to room temperature. To this were added benzophenone (**1**: 36.4 mg, 0.20 mmol, 1.0 equiv), $PdCl_2$ (1.8 mg, 0.010 mmol, 5.0 mol%), PPh_3 (10.5 mg, 0.040 mmol, 20 mol%), HCO_2Na (27.2 mg, 0.40 mmol, 2.0 equiv), diphenylphosphine oxide (60.7 mg, 0.30 mmol, 1.5 equiv), and 2,2,6,6-tetramethylpiperidine 1-oxyl (TEMPO: 15.6 mg, 0.10 mmol, 0.50 equiv). The vessel was placed under vacuum and refilled N_2 gas three times. To this vessel was added DME (1.0 mL). The vessel was sealed with an O-ring tap and then heated at 150 °C for 1 h in a 9-well reaction block with stirring. After cooling the reaction mixture to room temperature, the mixture was passed through a short silica-gel pad with EtOAc as an eluent. The filtrate was concentrated *in vacuo*. The yields of **2A** and **3A** were determined by crude ^{1}H NMR analysis as 34% and 66%, respectively, using CH_2Br_2 as an internal standard.

14-5. Deoxygenative Reaction of 1 with Phosphite Instead of Phosphine Oxide.

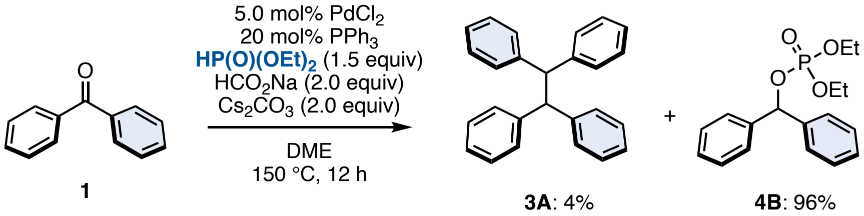

A 20-mL glass vessel equipped with J. Young® O-ring tap containing a magnetic stirring bar and Cs_2CO_3 (26.1 mg, 0.080 mmol, 0.40 equiv) was dried with a heat-gun *in vacuo* and filled with N_2 gas after cooling to room temperature. To this were added benzophenone (**1**: 36.4 mg, 0.20 mmol, 1.0 equiv), $PdCl_2$ (1.8 mg, 0.010 mmol, 5.0 mol%), PPh_3 (10.5 mg, 0.040 mmol, 20 mol%), HCO_2Na (27.2 mg, 0.40 mmol, 2.0 equiv), and diethyl phosphonate (39 μL, 0.30 mmol, 1.5 equiv). The vessel was placed under vacuum and refilled N_2 gas three times. To this vessel was added DME (1.0 mL). The vessel was sealed with an O-ring tap and then heated at 150 °C for 1 h in a 9-well reaction block with stirring. After cooling the reaction mixture to room temperature, the mixture was passed through a short silica-gel pad with EtOAc as an eluent. The filtrate was concentrated *in vacuo*. The yields of **3A** was determined by crude ^{1}H NMR analysis as 4% using CH_2Br_2 as an internal standard. In this case, we observed the generation of benzhydryl diethyl phosphate (**4B**, 96% yield determined by ^{1}H NMR).[23]

14-6. Preparation of Diphenylmethyl-*d* Diphenylphosphinate (S3)

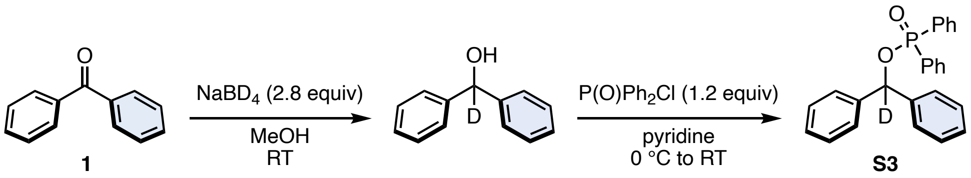

To a solution of benzophenone (**1**: 182.2 mg, 1.0 mmol, 1.0 equiv) in methanol (2.5 mL) was added sodium tetrahydroborate-d_4 (117.2 mg, 2.8 mmol, 2.8 equiv). This mixture was stirred for 3 h at room temperature. The mixture was added water and then extracted three times with CH_2Cl_2. The combined organic layer was dried over Na_2SO_4, filtrated, and then concentrated *in vacuo*. To the resulted mixture were added pyridine (0.50 mL) and diphenylphosphinic chloride (223.6 µL, 1.2 mmol, 1.2 equiv) at 0 °C. After stirring the mixture for 2 h at room temperature, the mixture was added 1M HCl aq. and extracted three times with EtOAc. The combined organic layer was dried over Na_2SO_4, filtrated, and then concentrated *in vacuo*. The residue was purified by recrystallization (CH_2Cl_2/hexane) to afford diphenylmethyl-*d* diphenylphosphinate (**S3**) as a colorless solid (131.1 mg, 34% yield, 98% D). ^{1}H NMR (400 MHz, $CDCl_3$) δ 7.79–7.63 (m, 4H), 7.49–7.41 (m, 2H), 7.39–7.18 (m, 14H), 6.49 (d, J = 10 Hz, 0.02H); ^{13}C NMR (101 MHz, $CDCl_3$) δ 140.6 (d, $J_{C–P}$ = 4.3 Hz), 132.0 (d, $J_{C–P}$ = 2.8 Hz), 131.73 (d, $J_{C–P}$ = 10.4 Hz), 131.66 (d, $J_{C–P}$ = 137.4 Hz), 128.3, 128.2, 127.8, 127.2 (the peak of O*C*HPh$_2$ was overlapped with $CDCl_3$); ^{31}P NMR (162 MHz, $CDCl_3$) δ 31.7; HRMS (ESI) *m*/*z* calcd for $C_{25}H_{20}DO_2NaP$ $[M+Na]^+$: 408.1234 found 408.1233.

15. X-ray Crystal Structure Analysis

15-1. 3A

Recrystallization from $CHCl_3$/hexane solution (vapor diffusion) gave crystals of **3A** suitable for X-ray analysis. A suitable crystal was mounted with Paratone oil on a MiTeGen MicroMounts and transferred to the 3-axis Eulerian Goniometer of a Rigaku R-AXIS RAPID II system with Ultrax 18 kW rotating anode X-ray generator using graphite-monochromated Cu-Kα radiation and imaging plate area detector. Cell parameters were determined and refined, and raw frame data were integrated using RAPID-AUTO (RIGAKU, 1998). The structures were solved by direct methods with (SHELXT)[24] and refined by full-matrix least-squares techniques against F^2 (SHELXL-2018/3)[25] by using Olex2 software package.[26] The intensities were corrected for Lorentz and polarization effects. The non-hydrogen atoms were refined anisotropically. Hydrogen atoms were placed using AFIX instructions.

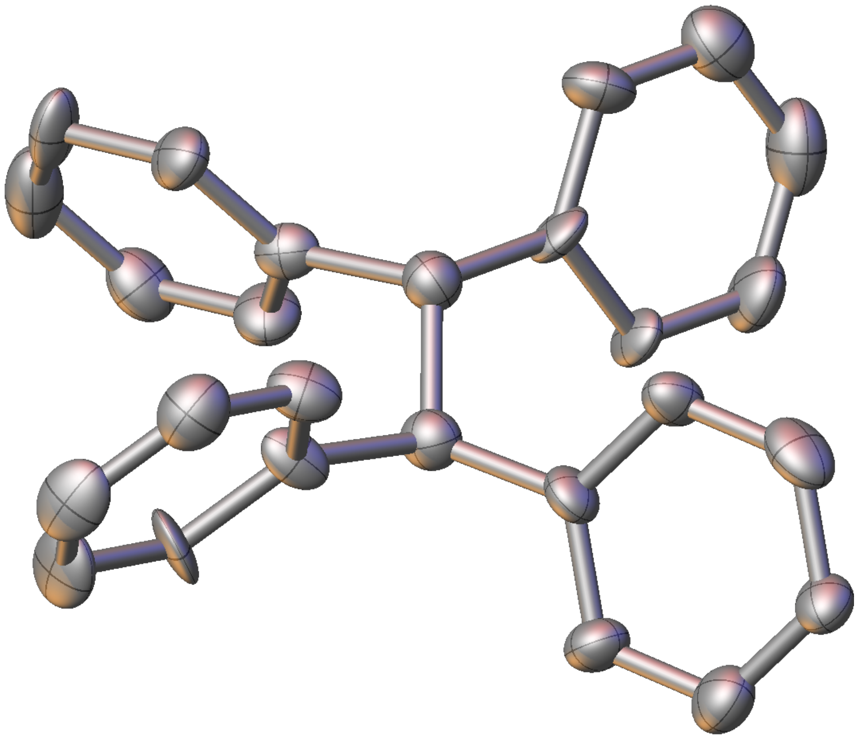

Figure 1. ORTEP drawing of one symmetry unique complete conformation of **3A** with 50% thermal ellipsoid. All hydrogen atoms and the disordered molecule are omitted for clarity.

Table 1 Crystal data and structure refinement for **3A**.

**Compound**	**3A**
CCDC Number	2121107
Empirical formula	$C_{26}H_{22}$
Formula weight	334.43
T / K	173(2)
Crystal system	monoclinic
Space group	$C2/c$
a / Å	17.661(3)
b / Å	5.8999(12)
c / Å	17.598(3)
α / °	90
β / °	91.211(7)
γ / °	90
V / Å^3	1833.4(6)
Z	4
D_{calc}, / g cm^{-3}	1.212
μ / mm^{-1}	0.513
F(000)	712.0
Crystal size / mm	0.1 × 0.1 × 0.1
λ / Å	1.5418
2θ range / °	10.018 to 136.368
Reflections collected	9136
Indep reflns/R_{int}	1672/0.0393
Params	187
GOF on F^2	1.124
R_1, wR_2 [I>2σ(I)]	0.0541, 0.1341
R_1, wR_2 [all data]	0.0707, 0.1497
Max./Mini. Peak / e Å^{-3}	0.16/–0.22

15-2. 3R

Recrystallization from acetone/methanol solution (vapor diffusion) gave crystals of **3R** suitable for X-ray analysis. A suitable crystal was mounted with Paratone oil on a MiTeGen MicroMounts and transferred to the 3-axis Eulerian Goniometer of a Rigaku R-AXIS RAPID II system with Ultrax 18 kW rotating anode X-ray generator using graphite-monochromated Cu-Kα radiation and imaging plate area detector. Cell parameters were determined and refined, and raw frame data were integrated using RAPID-AUTO (RIGAKU, 1998). The structures were solved by direct methods with (SHELXT)[24] and refined by full-matrix least-squares techniques against F^2 (SHELXL-2018/3)[25] by using Olex2 software package.[26] The intensities were corrected for Lorentz and polarization effects. The non-hydrogen atoms were refined anisotropically. Hydrogen atoms were placed using AFIX instructions.

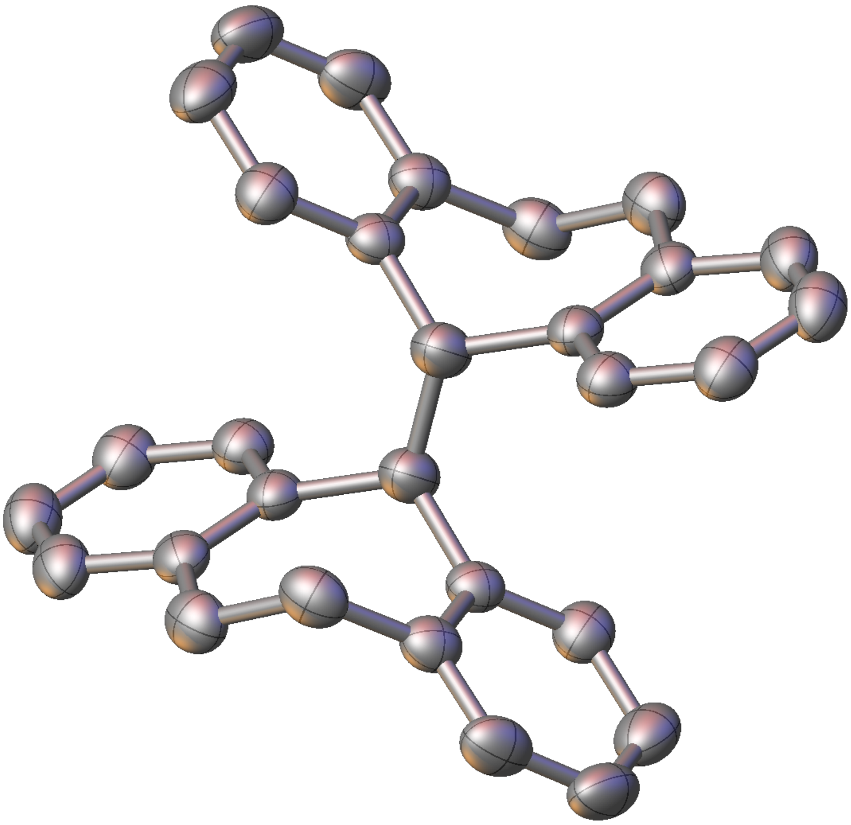

Figure 2. ORTEP drawing of **3R** with 50% thermal ellipsoid. All hydrogen atoms are omitted for clarity. Compound **3R** crystallizes with two independent molecules in the unit cell (only one molecule is shown in Figure 2).

Table 2 Crystal data and structure refinement for **3R**.

**Compound**	**3R**
CCDC Number	2121108
Empirical formula	$C_{30}H_{26}$
Formula weight	386.51
T / K	173(2)
Crystal system	triclinic
Space group	P–1
a / Å	9.9103(12)
b / Å	10.3946(13)
c / Å	10.4347(13)
α / °	98.955(7)
β / °	91.413(6)
γ / °	103.689(7)
V / Å^3	1029.5(2)
Z	2
D_{calc}, / g cm^{-3}	1.247
μ / mm^{-1}	0.527
F(000)	412.0
Crystal size / mm	0.2 × 0.2 × 0.1
λ / Å	1.5418
2θ range / °	8.596 to 136.42
Reflections collected	11946
Indep reflns/R_{int}	3689/0.0480
Params	271
GOF on F^2	0.948
R_1, wR_2 [I>2σ(I)]	0.0485, 0.1124
R_1, wR_2 [all data]	0.0816, 0.1241
Max./Mini. Peak / e Å^{-3}	0.13/–0.22

15-3. 18

Recrystallization from toluene/hexane solution (vapor diffusion) gave crystals of **18** suitable for X-ray analysis. A suitable crystal was mounted with Immersion oil viscosity 1,250 cSt (lit.) (SIGMA–ALDRICH) on a MiTeGen MicroMounts and transferred to Rigaku XtaLAB Synergy-S diffractometer equipped with a HyPix-6000HE Hybrid Photon Counting detector and dual Mo and Cu microfocus sealed tube. Cell parameters were determined and refined, and raw frame data were integrated using CrysAlis^{Pro} (Rigaku Oxford Diffraction, 2021).[27] The structures were solved by direct methods with (SHELXT)[24] and refined by full-matrix least-squares techniques against F^2 (SHELXL-2018/3)[25] by using Olex2 software package.[26] The intensities were corrected for Lorentz and polarization effects. The non-hydrogen atoms were refined anisotropically. Hydrogen atoms were placed using AFIX instructions.

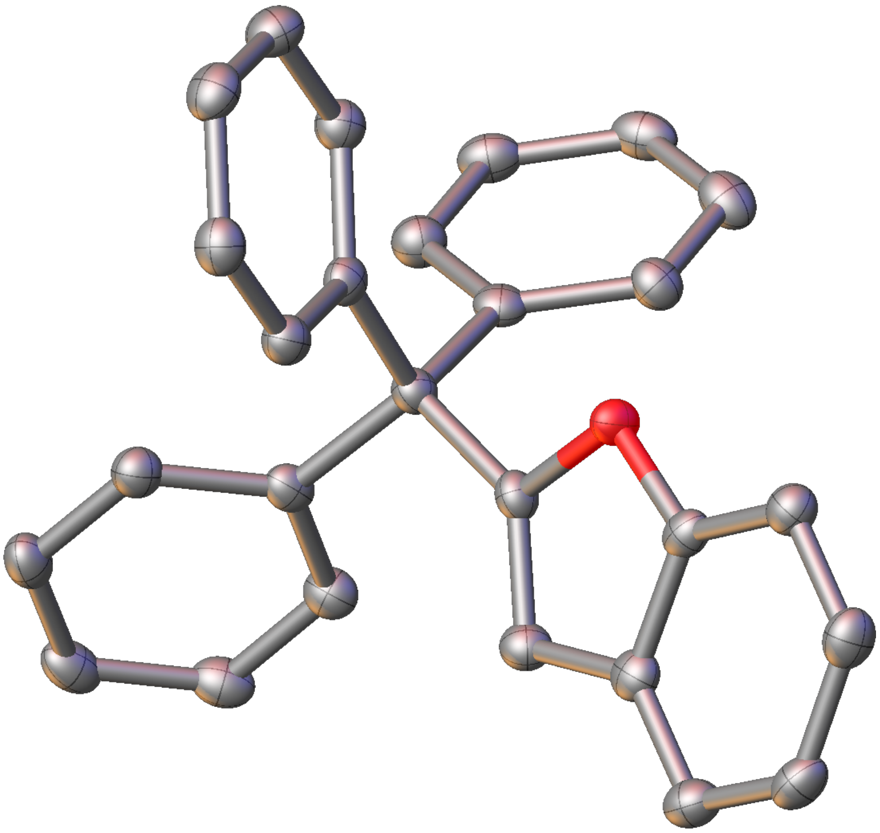

Figure 3. ORTEP drawing of **18** with 50% thermal ellipsoid. All hydrogen atoms are omitted for clarity.

Table 3 Crystal data and structure refinement for **18**.

**Compound**	**18**
CCDC Number	2159413
Empirical formula	$C_{27}H_{20}O$
Formula weight	360.43
T / K	100(10)
Crystal system	monoclinic
Space group	$P2_1$
a / Å	7.33910(10)
b / Å	10.8689(2)
c / Å	11.7668(2)
α / °	90
β / °	96.0850(10)
γ / °	90
V / Å^3	933.33(3)
Z	2
D_{calc}, / g cm^{-3}	1.283
μ / mm^{-1}	0.588
F(000)	380.0
Crystal size / mm	0.28 × 0.09 × 0.05
λ / Å	1.54184
2θ range / °	7.556 to 150.166
Reflections collected	11335
Indep reflns/R_{int}	3526/0.0401
Params	253
GOF on F^2	1.043
R_1, wR_2 [I>2σ(I)]	0.0315, 0.0761
R_1, wR_2 [all data]	0.0331, 0.0773
Max./Mini. Peak / e Å^{-3}	0.14/–0.17

16. References

[1] Seo, T.; Ishiyama, T.; Kubota, K.; Ito, H. Solid-State Suzuki–Miyaura Cross-Coupling Reactions: Olefin-Accelerated C–C Coupling Using Mechanochemistry. *Chem. Sci.* **2019**, *10*, 8202–8210.

[2] Gao, G.-L.; Niu, Y.-N.; Yan, Z.-Y.; Wang, H.-L.; Wang, G.-W.; Shaukat, A.; Liang, Y.-M. Unexpected Domino Reaction via Pd-Catalyzed Sonogashira Coupling of Benzimidoyl Chlorides with 1,6-Enynes and Cyclization to Synthesize Quinoline Derivatives. *J. Org. Chem.* **2010**, *75*, 1305–1308.

[3] Idris, M. A.; Lee, S. Palladium-Catalyzed Amide N–C Hiyama Cross-Coupling: Synthesis of Ketones. *Org. Lett.* **2020**, *22*, 9190–9195.

[4] Mai, S.; Li, W.; Li, X.; Zhao, Y.; Song, Q. Palladium-Catalyzed Suzuki–Miyaura Coupling of Thioureas or Thioamides. *Nat. Chem.* **2019**, *10*, 5709–5720.

[5] Liu, J.; Zhou, X.; Rao, H.; Xiao, F.; Li, C.-J., Deng, G.-J. Direct Synthesis of Aryl Ketones by Palladium-Catalyzed Desulfinative Addition of Sodium Sulfinates to Nitriles. *Chem. Eur. J.* **2011**, *17*, 7996–7999.

[6] Zhang, C.; Rao, Y. Weak Coordination Promoted Regioselective Oxidative Coupling Reaction for 2,2′-Difunctional Biaryl Synthesis in Hexafluoro-2-propanol. *Org. Lett.* **2015**, *17*, 4456–4459.

[7] Kim, C.-B.; Jo, H.; Ahn, B.-K.; Kim, C. K.; Park, K. Nickel N-Heterocyclic Carbene Catalyst for Cross-Coupling of Neopentyl Arenesulfonates with Methyl and Primary Alkyl Grignard Reagents. *J. Org. Chem.* **2009**, *74*, 9566–9569.

[8] Huang, C.-Y.; Li, J.; Liu, W.; Li, C.-J. Diacetyl as a “Traceless” Visible Light Photosensitizer in Metal-Free Cross-Dehydrogenative Coupling Reactions. *Chem. Sci.* **2019**, *10*, 5018–5024.

[9] Xue, C.; Wang, L.; Han, J. Palladium-Catalyzed Site-Selective Benzocyclization of Naphthoic Acids with Diaryliodonium Salts: Efficient Access to Benzanthrones. *J. Org. Chem.* **2020**, *85*, 15406–15414.

[10] Sumiyama, K.; Toriumi, N.; Iwasawa, N. Use of Isopropyl Alcohol as a Reductant for Catalytic Dehydoxylative Dimerization of Benzylic Alcohols Utilizing Ti–O Bond Photohomolysis. *Eur. J. Org. Chem.* **2021**, 2474–2478.

[11] Wakui, H.; Kawasaki, S.; Satoh, T.; Miura, M.; Nomura, M. Palladium-Catalyzed Reaction of 2-Hydroxy-2-methylpropiophenone with Aryl Bromides: A Unique Multiple Arylation via Successive C–C and C–H Bond Cleavages. *J. Am. Chem. Soc.* **2004**, *126,* 8658–8659.

[12] Chung, M.-K.; Qi, G.; Stryker, J. M. Synthesis of Sterically Hindered Ortho-Substituted Tetraphenylethenes. Electronic Effects in the McMurry Olefination Reaction. *Org. Lett.* **2006**, *8*, 1491–1494.

[13] Agranat, I.; Cohen, S.; Isaksson, R.; Sandstroem, J.; Suissa, M. R. Static and Dynamic Stereochemistry of a Chiral, Doubly Bridged 9,10-Diphenylanthracene from a Stereospecific Polycyclic Aromatic Dicarbonyl Coupling. *J. Org. Chem.* **1990***, 55,* 4943–4950.

[14] Nambo, M.; Crudden, C. M. Modular Synthesis of Triarylmethanes Through Palladium-Catalyzed

Sequential Arylation of Methyl Phenyl Sulfone. *Angew. Chem., Int. Ed.* **2014**, *53*, 742–746.
[15] Saha, T.; Kumar, M. S. L.; Bera, S.; Karkara, B. B.; Panda, G. Efficient Access to Triarylmethanes Through Decarboxylation. *RSC Adv.* **2017**, *7,* 6966–6971.
[16] Sato, Y.; Aoyama, T.; Takido, T.; Kodomari, M. Direct Alkylation of Aromatics Using Alcohols in the Presence of $NaHSO_4/SiO_2$. *Tetrahedron* **2012**, *68*, 7077–7081.
[17] Prakash, G. K. S.; Panja, C.; Shakhmin, A.; Shah, E.; Mathew, T.; Olah, G. A. BF_3-H_2O Catalyzed Hydroxyalkylation of Aromatics with Aromatic Aldehydes and Dicarboxaldehydes: Efficient Synthesis of Triarylmethanes, Diarylmethylbenzaldehydes, and Anthracene Derivatives. *J. Org. Chem.* **2009**, *74*, 8659–8668.
[18] Zhang, Z.; Wang, H.; Qiu, N.; Kong, Y.; Zeng, W.; Zhang, Y.; Zhao, J. Synthesis of Triarylmethanes via Palladium-Catalyzed Suzuki Coupling of Trimethylammonium Salts and Arylboronic Acids. *J. Org. Chem*. **2018**, *83*, 8710–8715.
[19] Peng, B.; Feng, X.; Zhang, X.; Ji, L.; Bao, M. Regioselective Control Using a Catalyst Switch in the Reaction of Diarylmethyl Chlorides with Allyltributylstannane. *Tetrahedron* **2010**, *66*, 6013–6018.
[20] Anthony, D.; Lin, Q.; Baudet, J.; Diao, T. Nickel-Catalyzed Asymmetric Reductive Diarylation of Vinylarenes. *Angew. Chem., Int. Ed.* **2019**, *58*, 3198–3202.
[21] Nambo, M.; Yim, J. C.-H.; Fowler, K. G.; Crudden, C. M. Synthesis of Tetraarylmethanes by the Triflic Acid-Promoted Formal Cross-Dehydrogenative Coupling of Triarylmethanes with Arenes. *Synlett* **2017**, *28*, 2936–2940.
[22] Qian, Y.; Dai, Q.; Li, Z.; Liu, Y.; Zhang, J. *O*-Phosphination of Aldehydes/Ketones Toward Phosphoric Esters: Experimental and Mechanistic Studies. *Org. Lett.* **2020**, *22*, 4742–4748.
[23] Dai, Q.; Liu, L.; Zhang, J. Palladium/Xiao-Phos-Catalyzed Kinetic Resolution of *sec*-Phosphine Oxides by *P*-Benzylation. *Angew. Chem., Int. Ed.* **2021**, *60*, 27247–27252.
[24] Sheldrick, G. M. *SHELXT* – Integrated Space-Group and Crystal-Structure Determination. *Acta Cryst.* **2015**, *A71*, 3–8.
[25] Sheldrick, G. M. Crystal Structure Refinement with *SHELXL*. *Acta Cryst.* **2015**, *C71,* 3–8.
[26] Dolomanov, O. V.; Bourhis, L. J.; Gildea, R. J.; Howard, J. A. K.; Puschmann, H. *OLEX2*: A Complete Structure Solution, Refinement and Analysis Program. *J. Appl. Crystallogr.* **2009**, *42,* 339–341.
[27] Matsumoto, T.; Yamano, Y.; Sato, T.; Ferrara, J. D.; White, F. J.; Meyer, M. "What is this?" A Structure Analysis Tool for Rapid and Automated Solution of Small Molecule Structures. *J. Chem. Crystallogr.* **2021***, 51,* 438–450.

17. ^{1}H , ^{13}C, ^{19}F and ^{31}P NMR Spectra

^{1}H NMR of **2E** (400 MHz, $CDCl_3$)

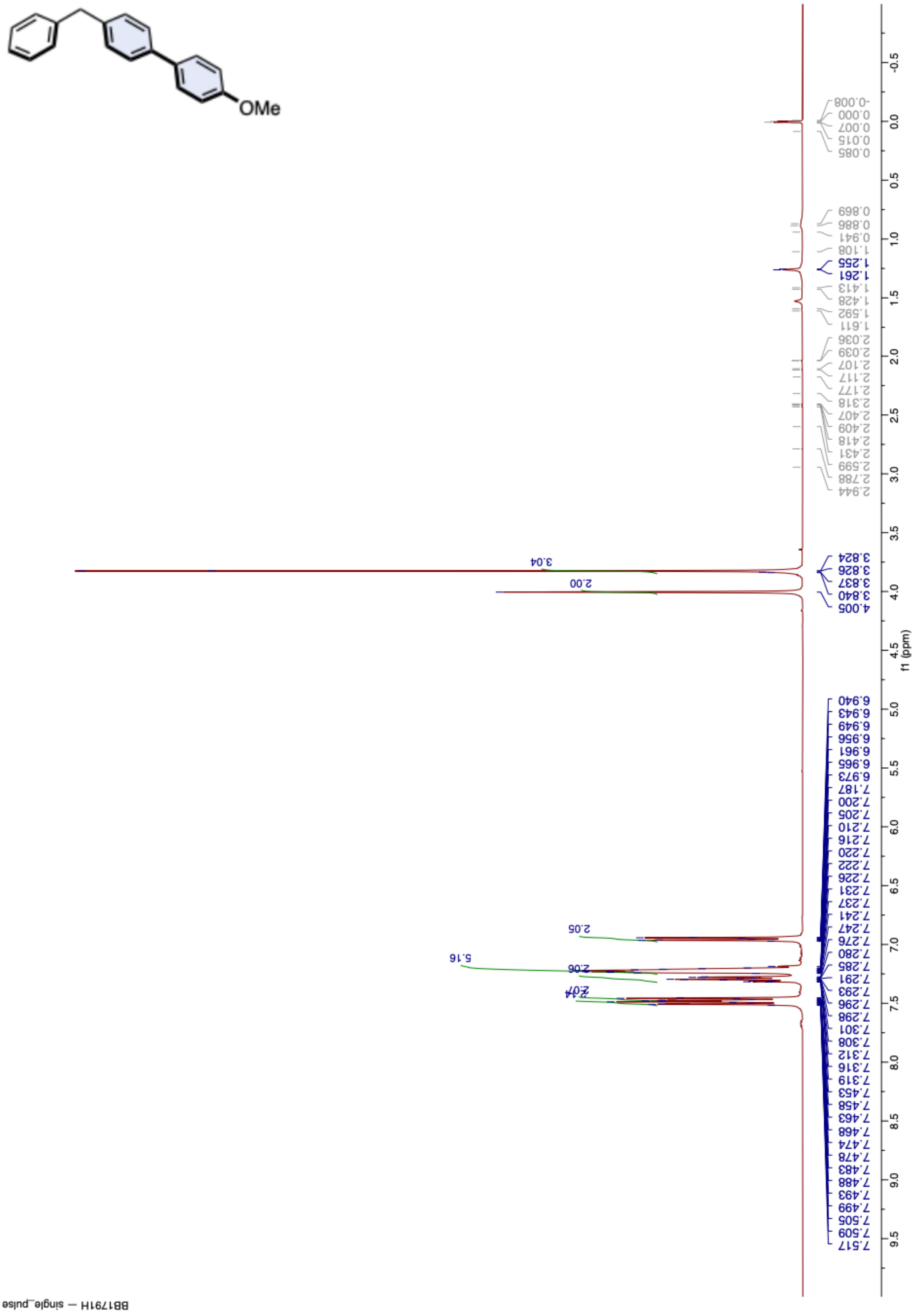

^{13}C NMR of **2E** (101 MHz, $CDCl_3$)

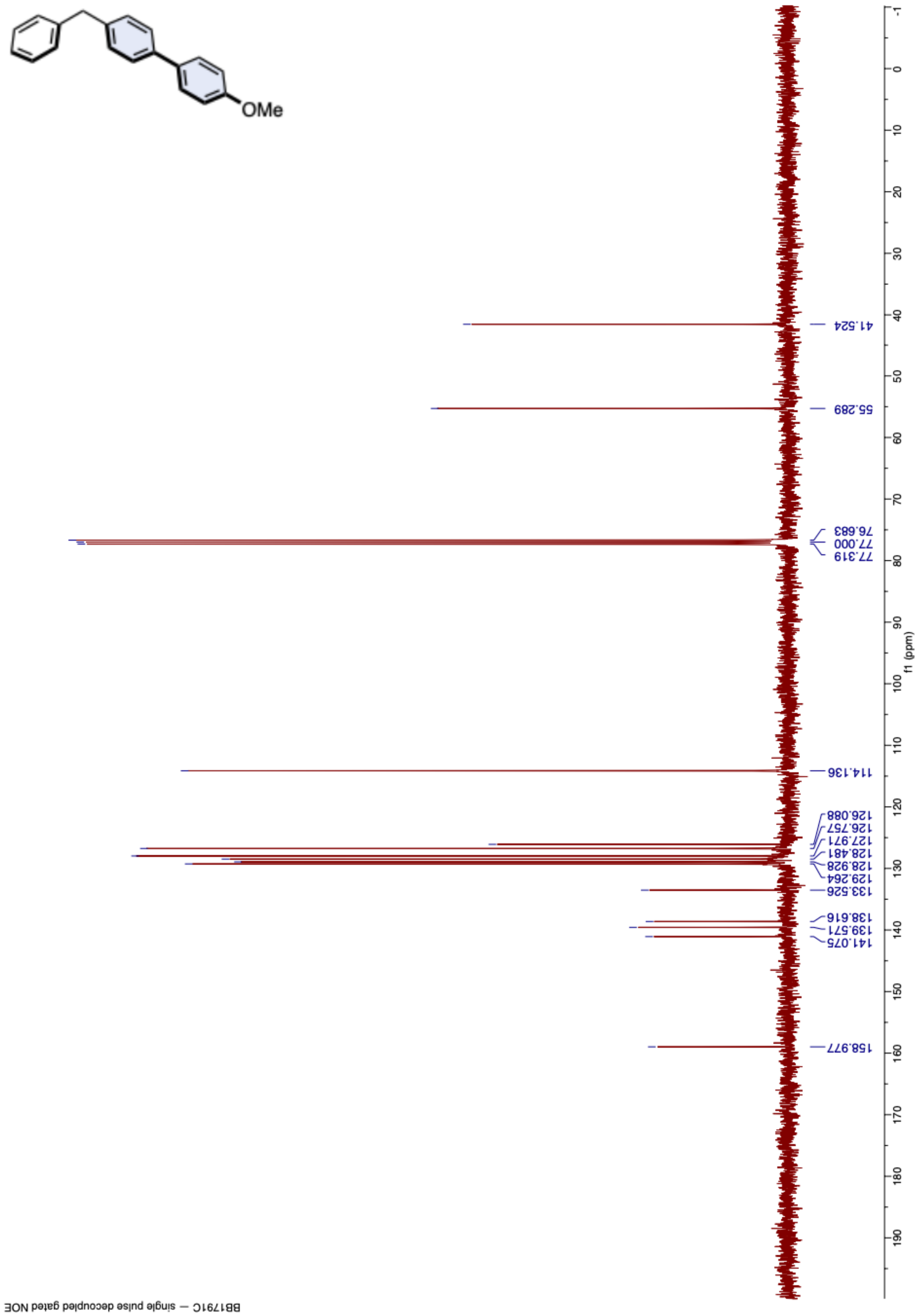

^{1}H NMR of **2G** (400 MHz, $CDCl_3$)

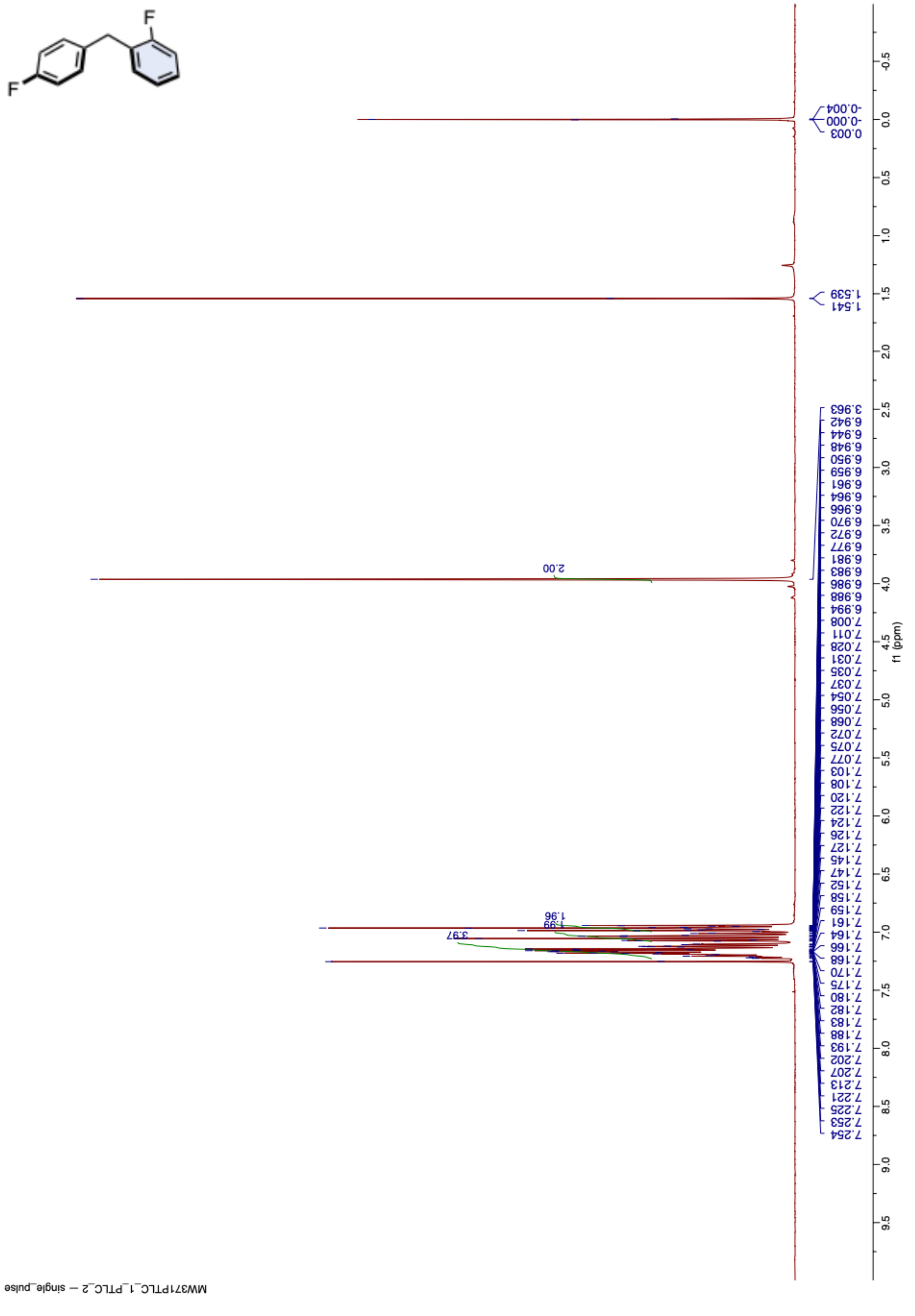

^{13}C NMR of **2G** (101 MHz, $CDCl_3$)

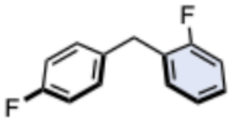

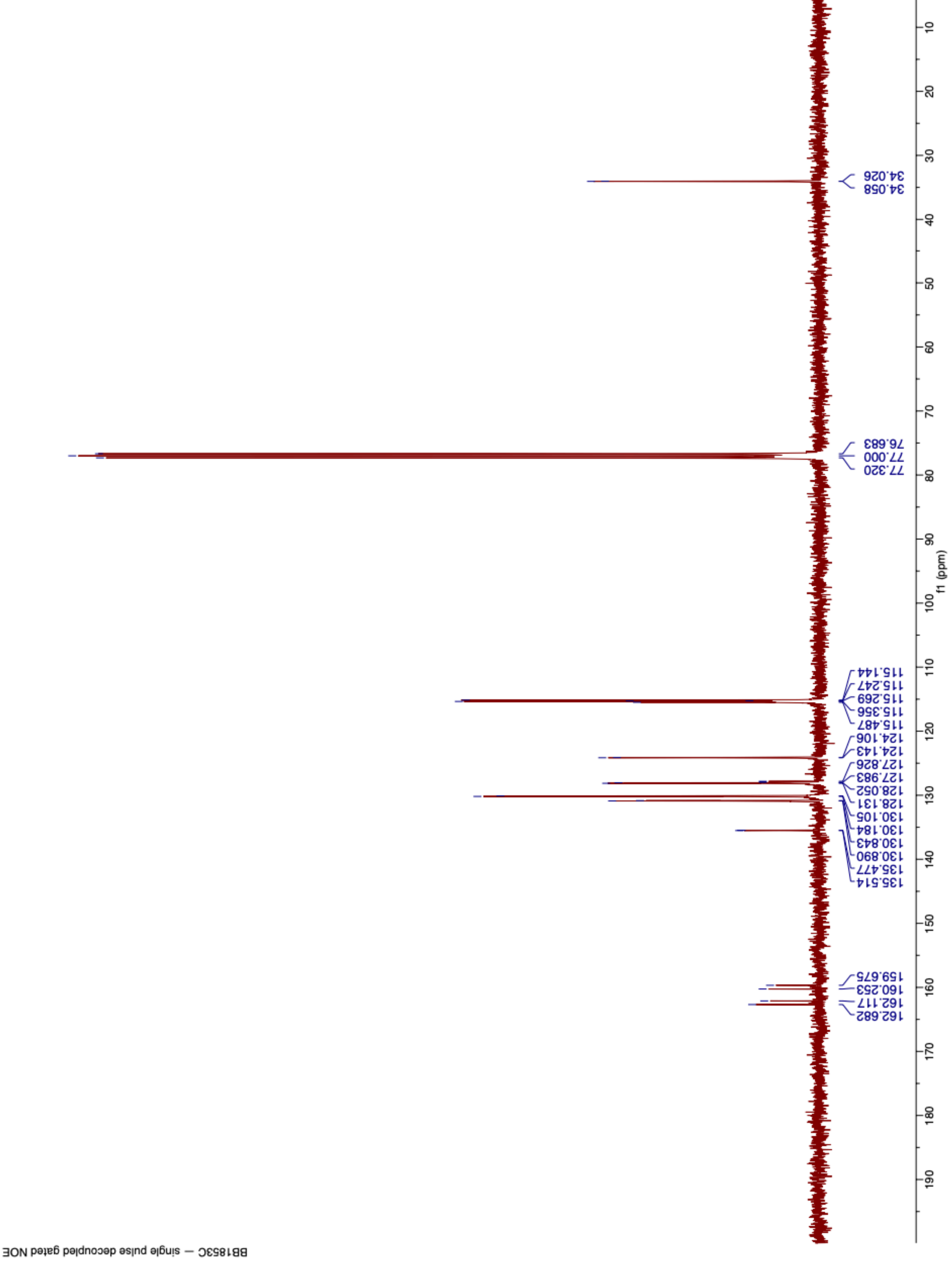

^{19}F NMR of **2G** (376 MHz, $CDCl_3$)

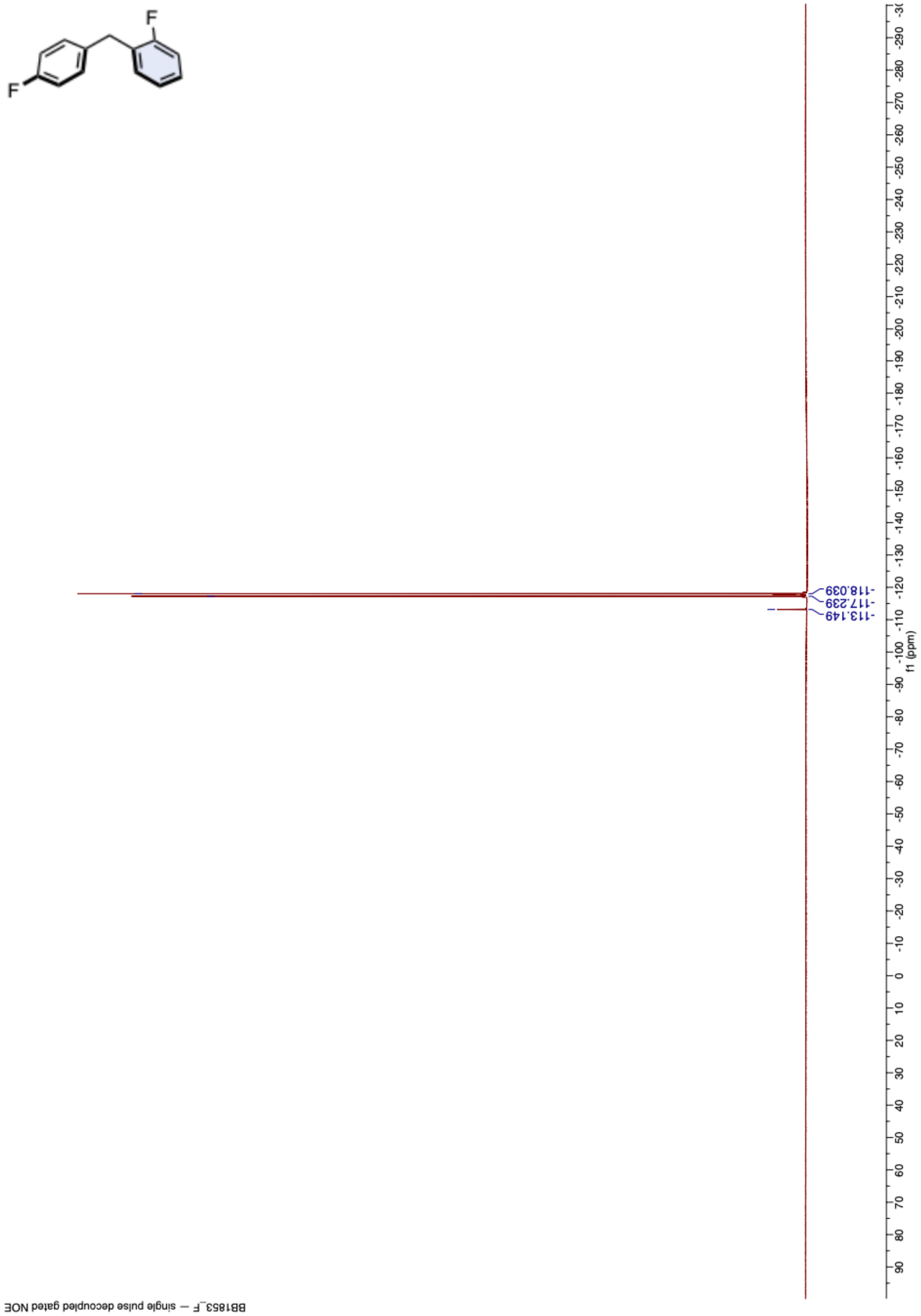

^{1}H NMR of **2H** (400 MHz, $CDCl_3$)

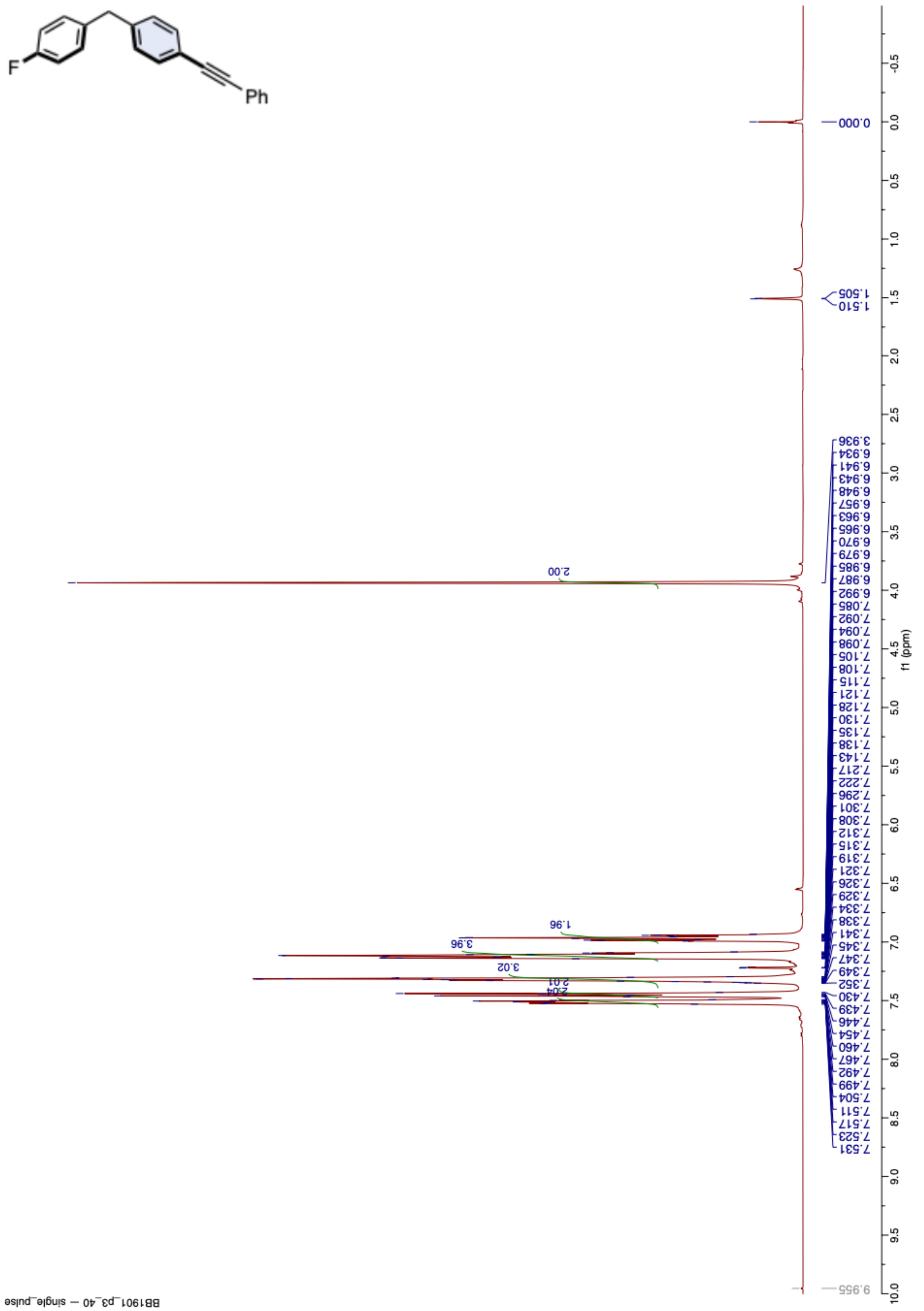

^{13}C NMR of **2H** (101 MHz, $CDCl_3$)

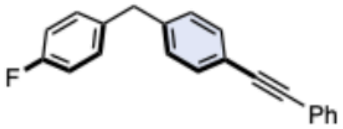

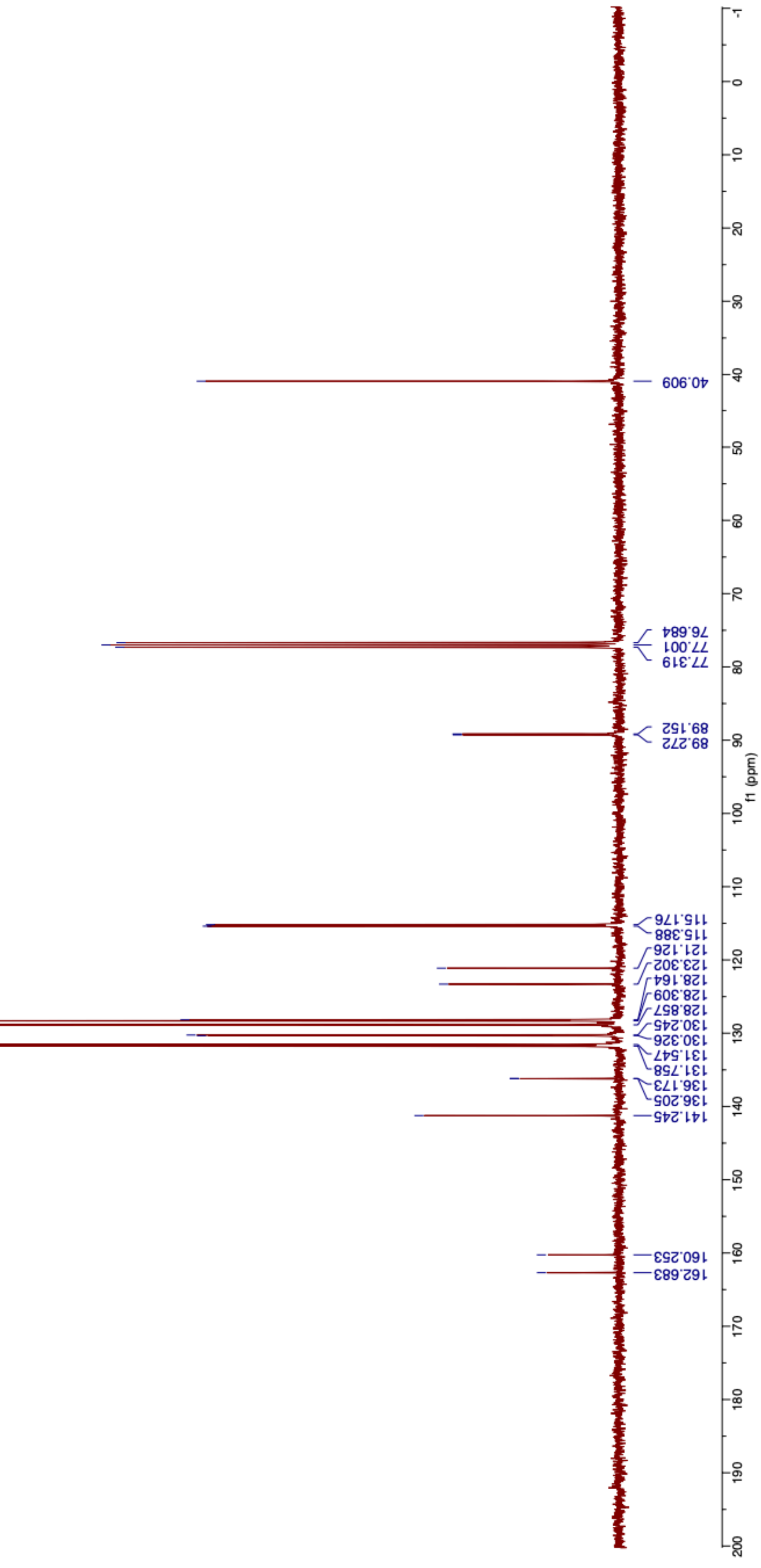

^{19}F NMR of **2H** (376 MHz, $CDCl_3$)

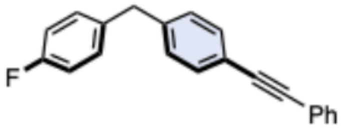

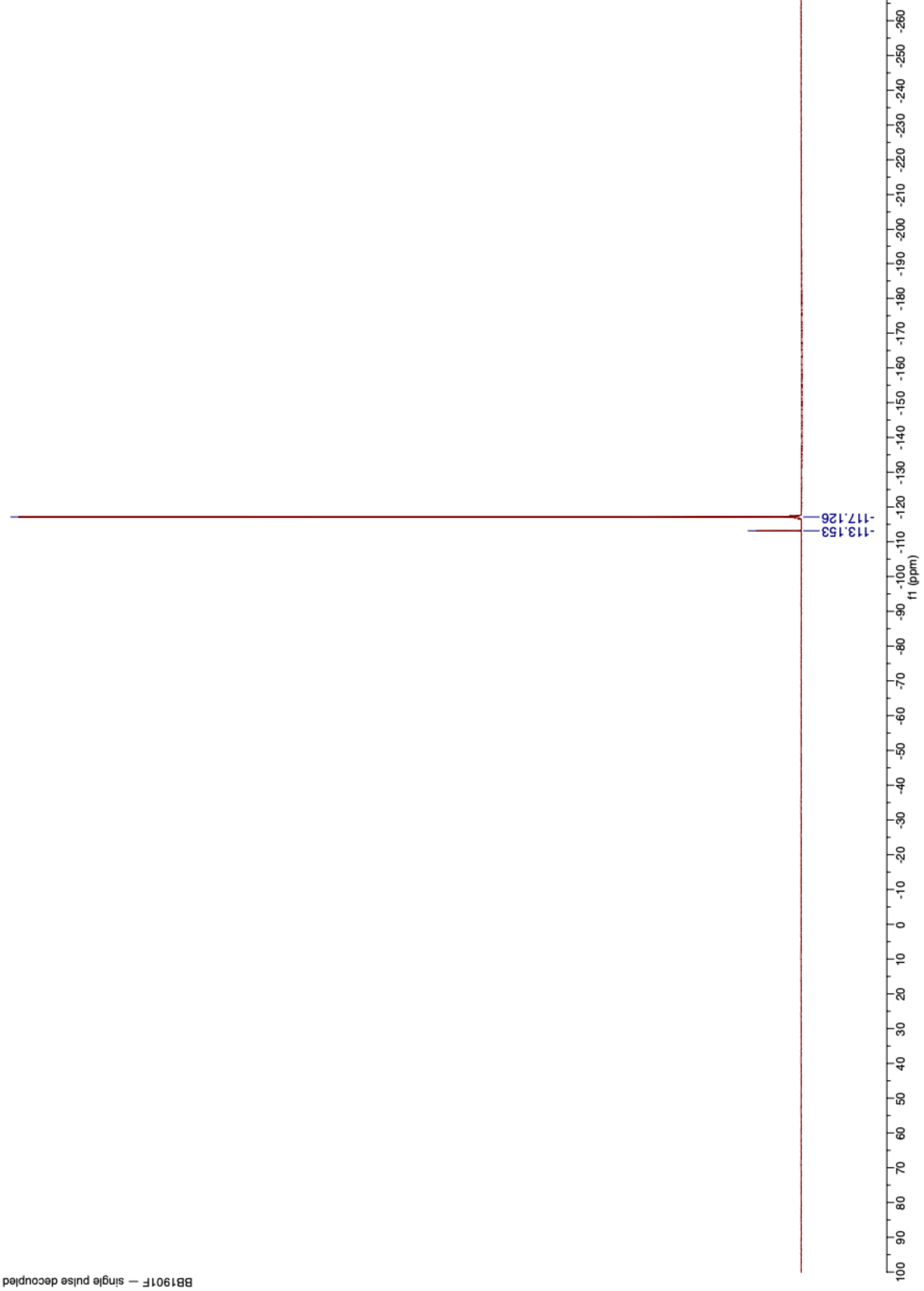

^{1}H NMR of **2N** (400 MHz, $CDCl_3$)

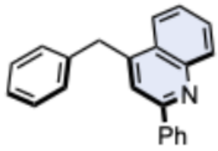

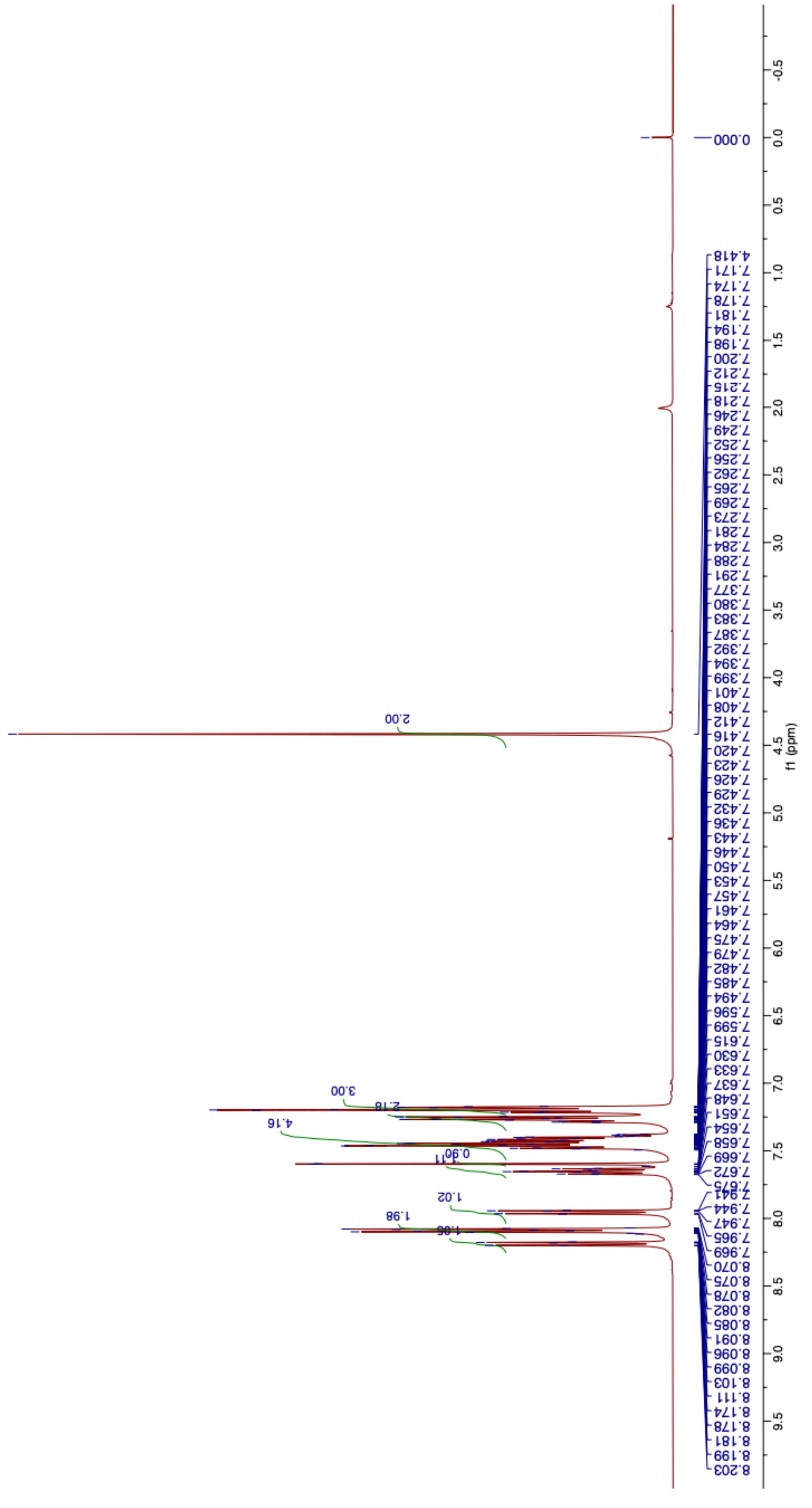

^{13}C NMR of **2N** (101 MHz, $CDCl_3$)

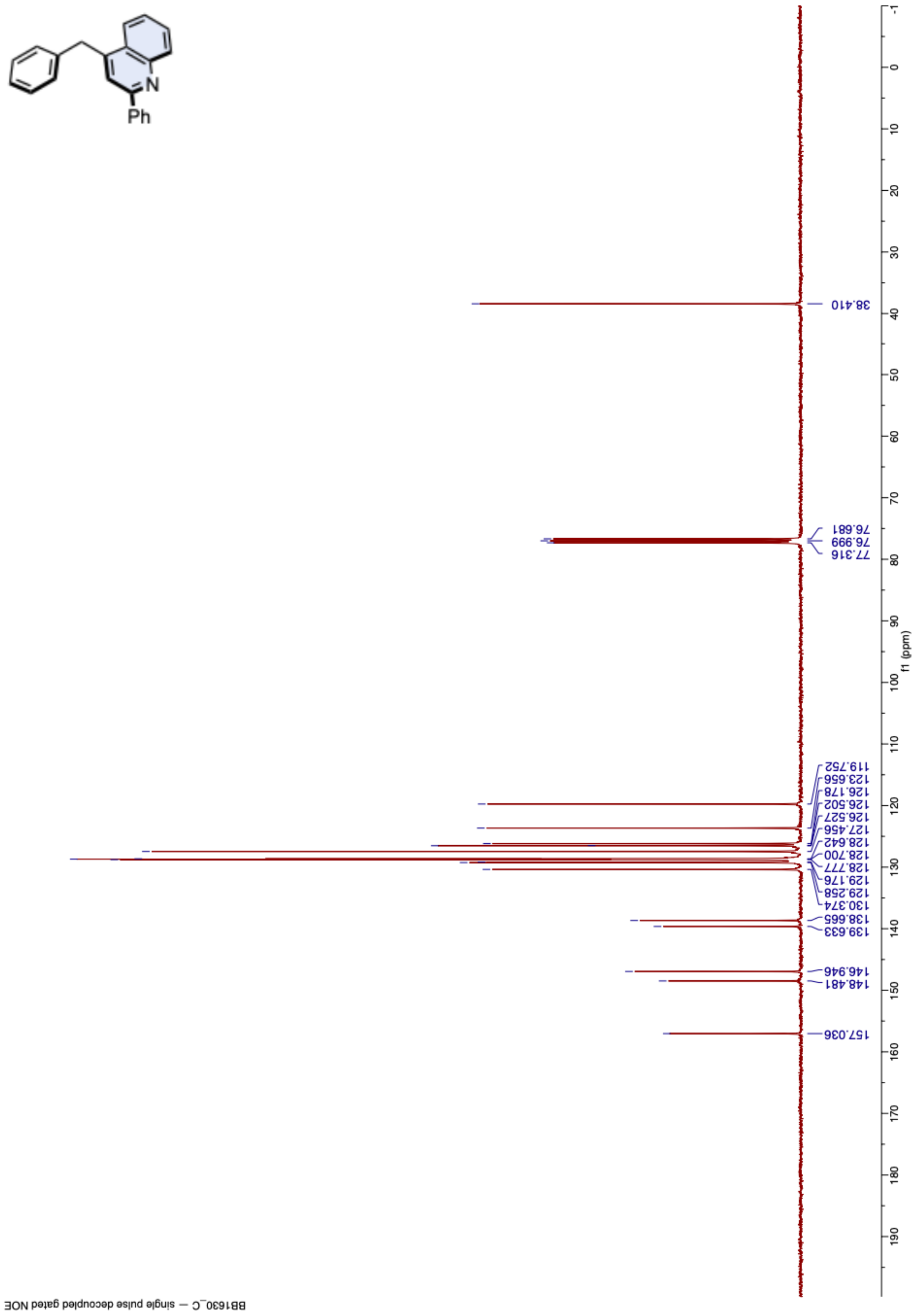

^{1}H NMR of **2O** (400 MHz, $CDCl_3$)

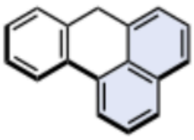

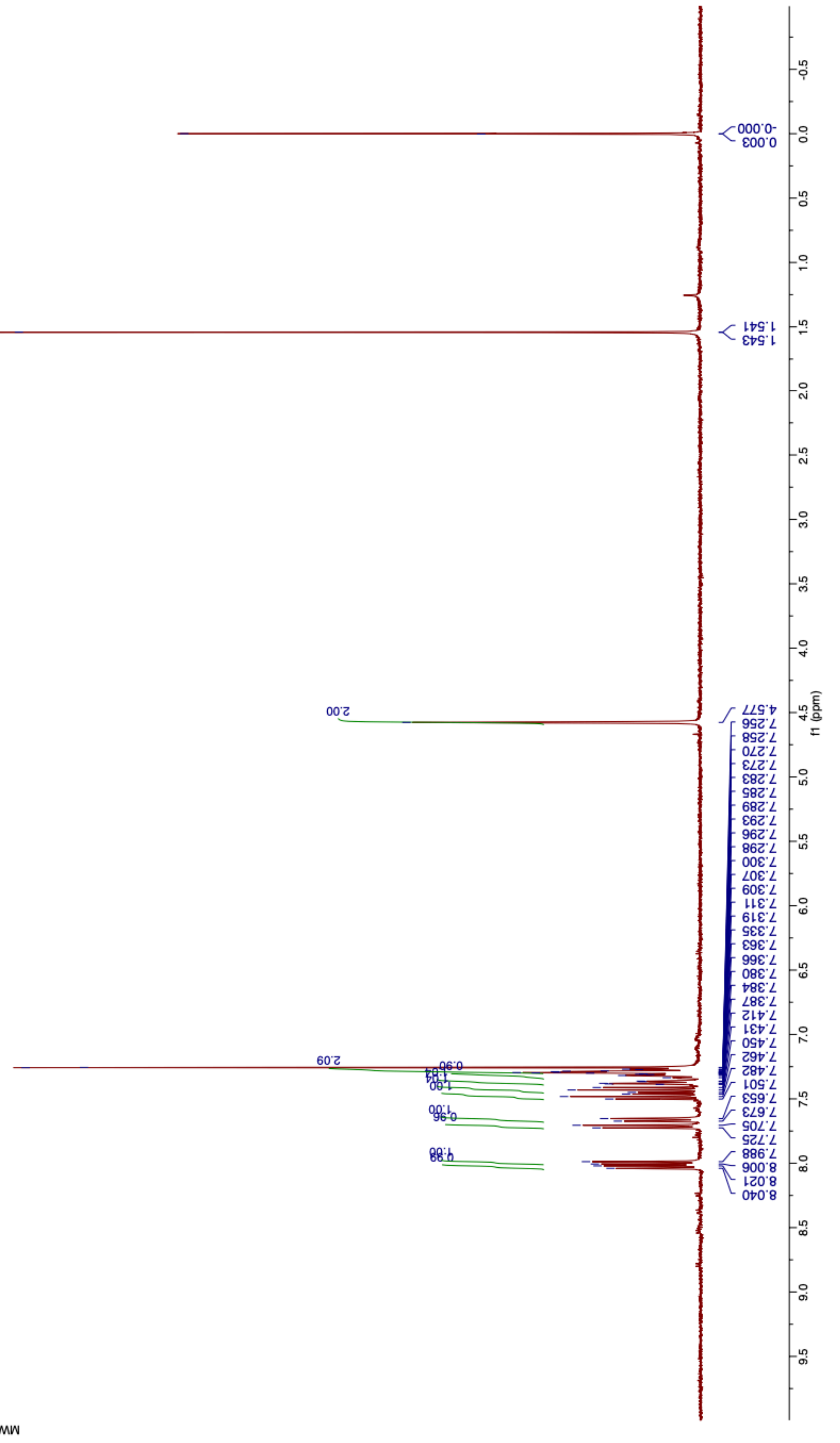

^{13}C NMR of **2O** (101 MHz, $CDCl_3$)

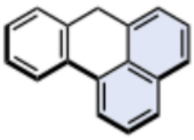

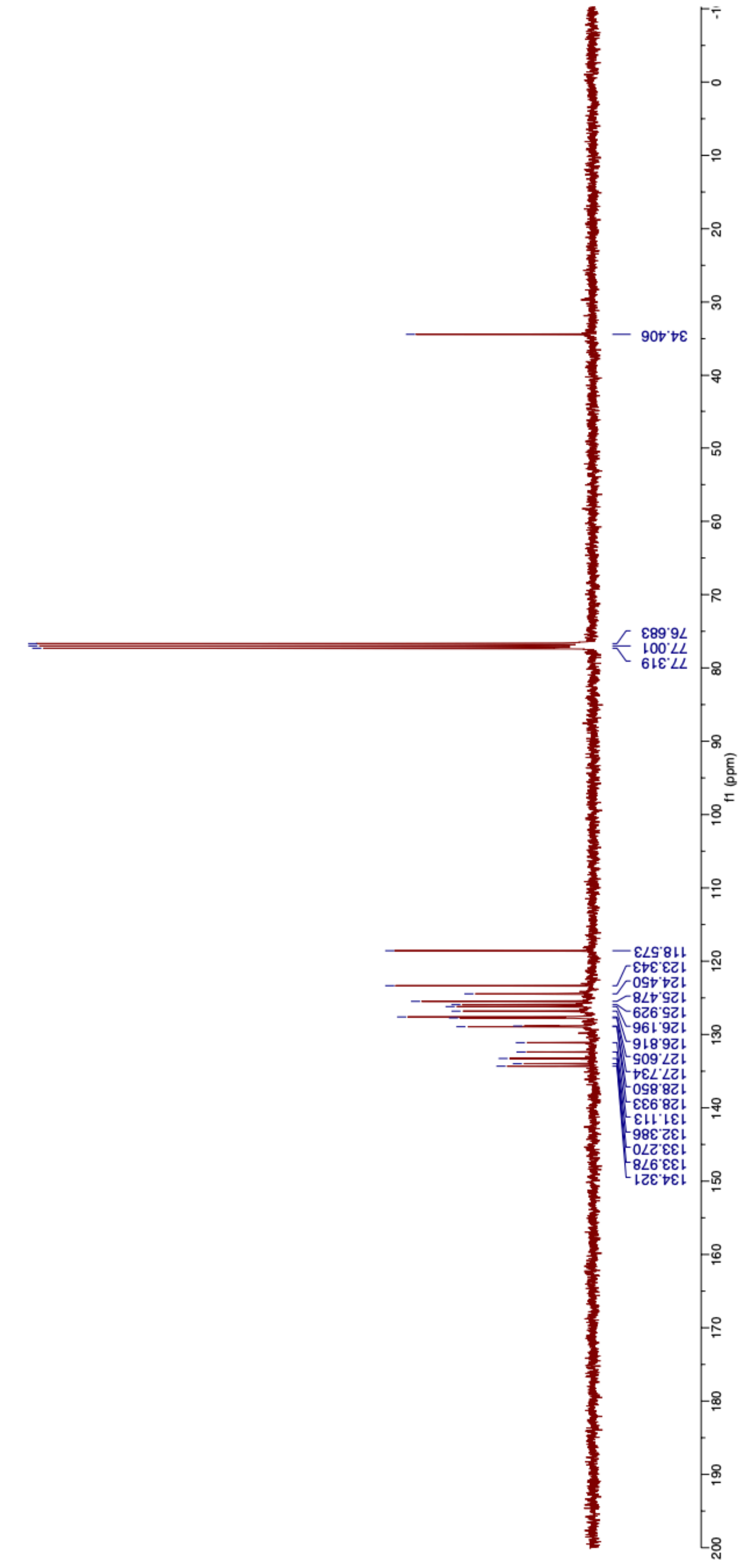

^{1}H NMR of **2T** (400 MHz, $CDCl_3$)

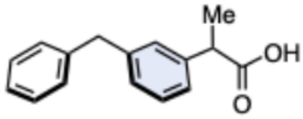

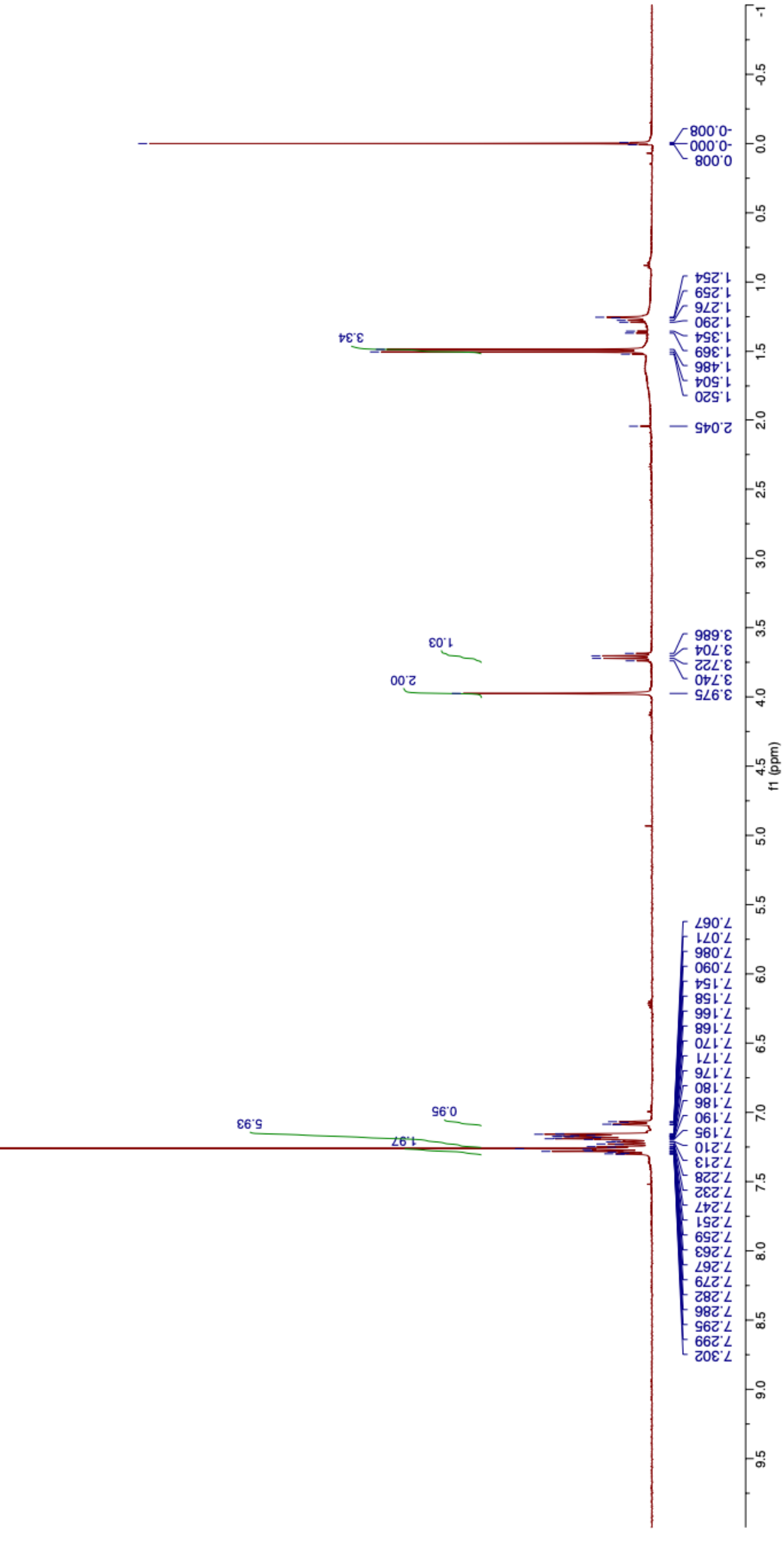

^{13}C NMR of **2T** (101 MHz, $CDCl_3$)

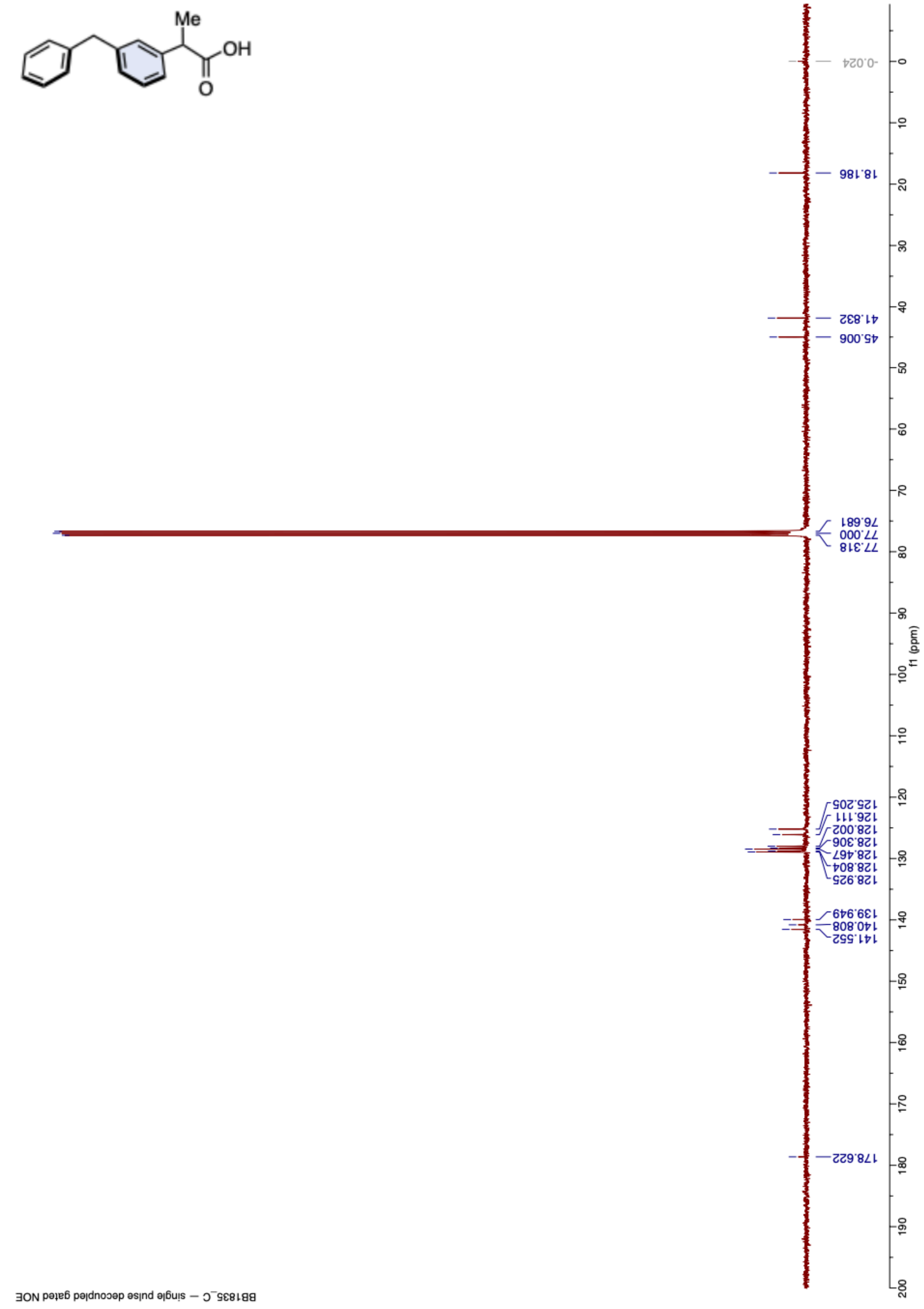

^{1}H NMR of **2U** (400 MHz, $CDCl_3$)

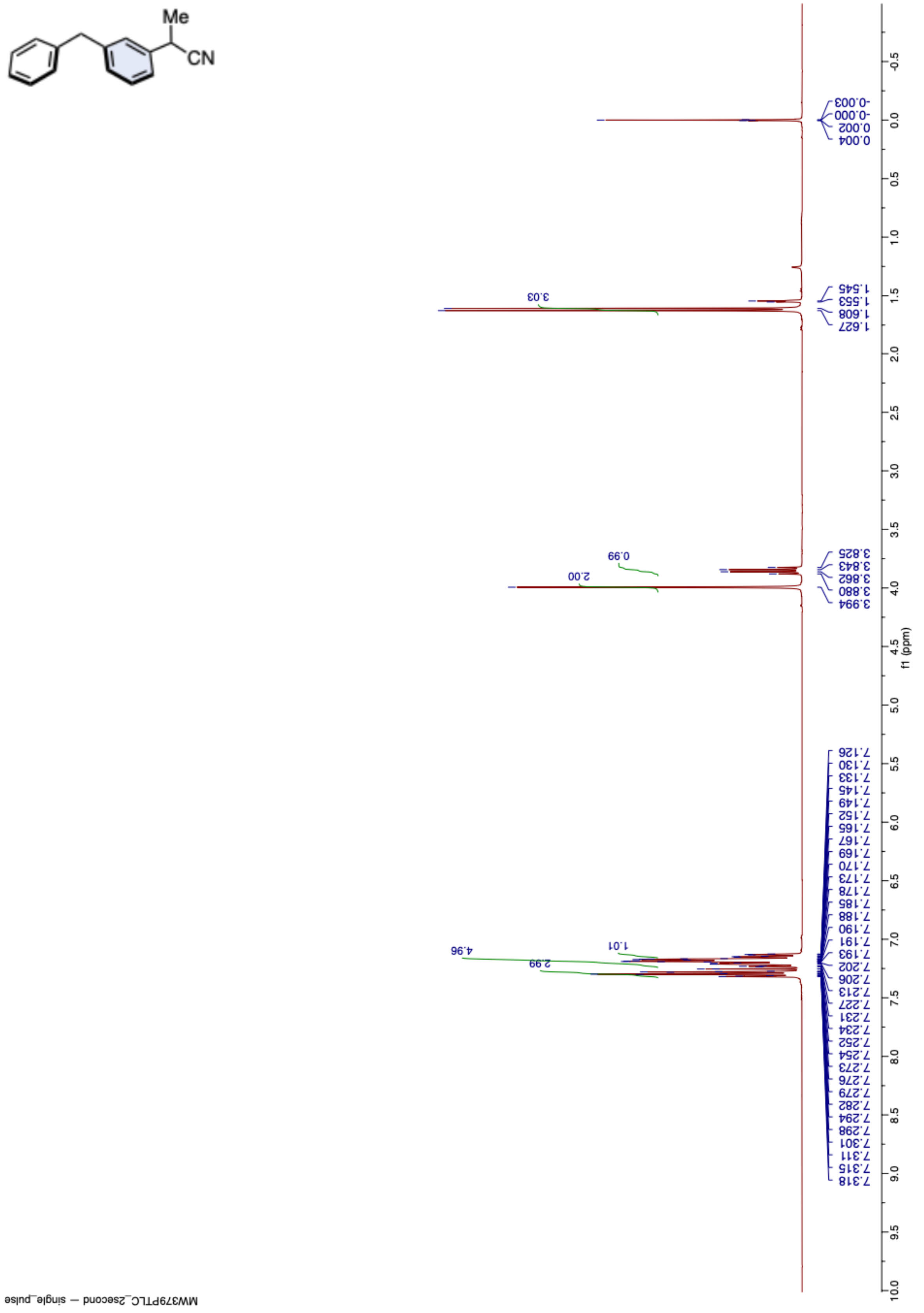

^{13}C NMR of **2U** (101 MHz, $CDCl_3$)

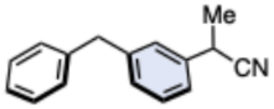

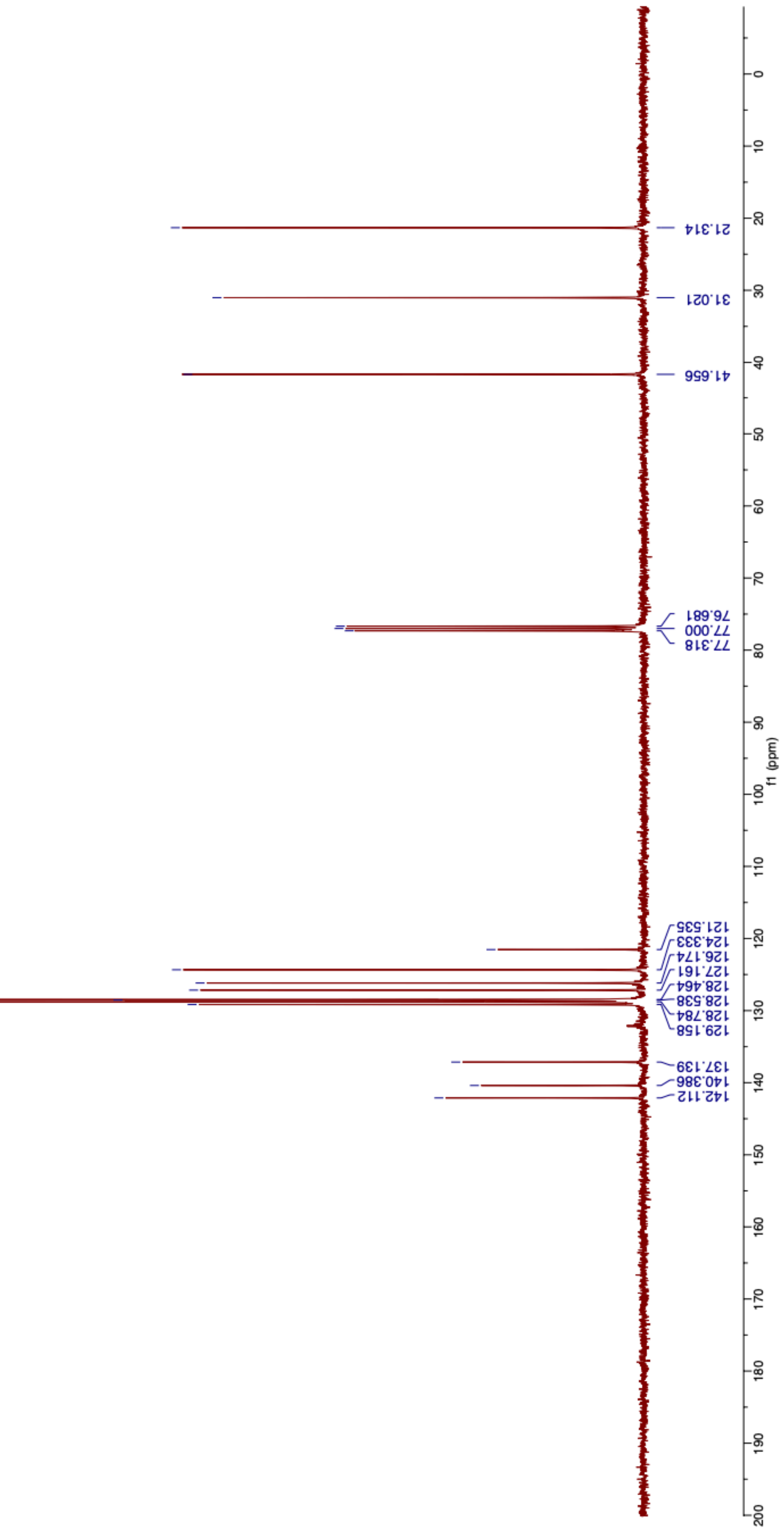

^{1}H NMR of **2V** (400 MHz, $CDCl_3$)

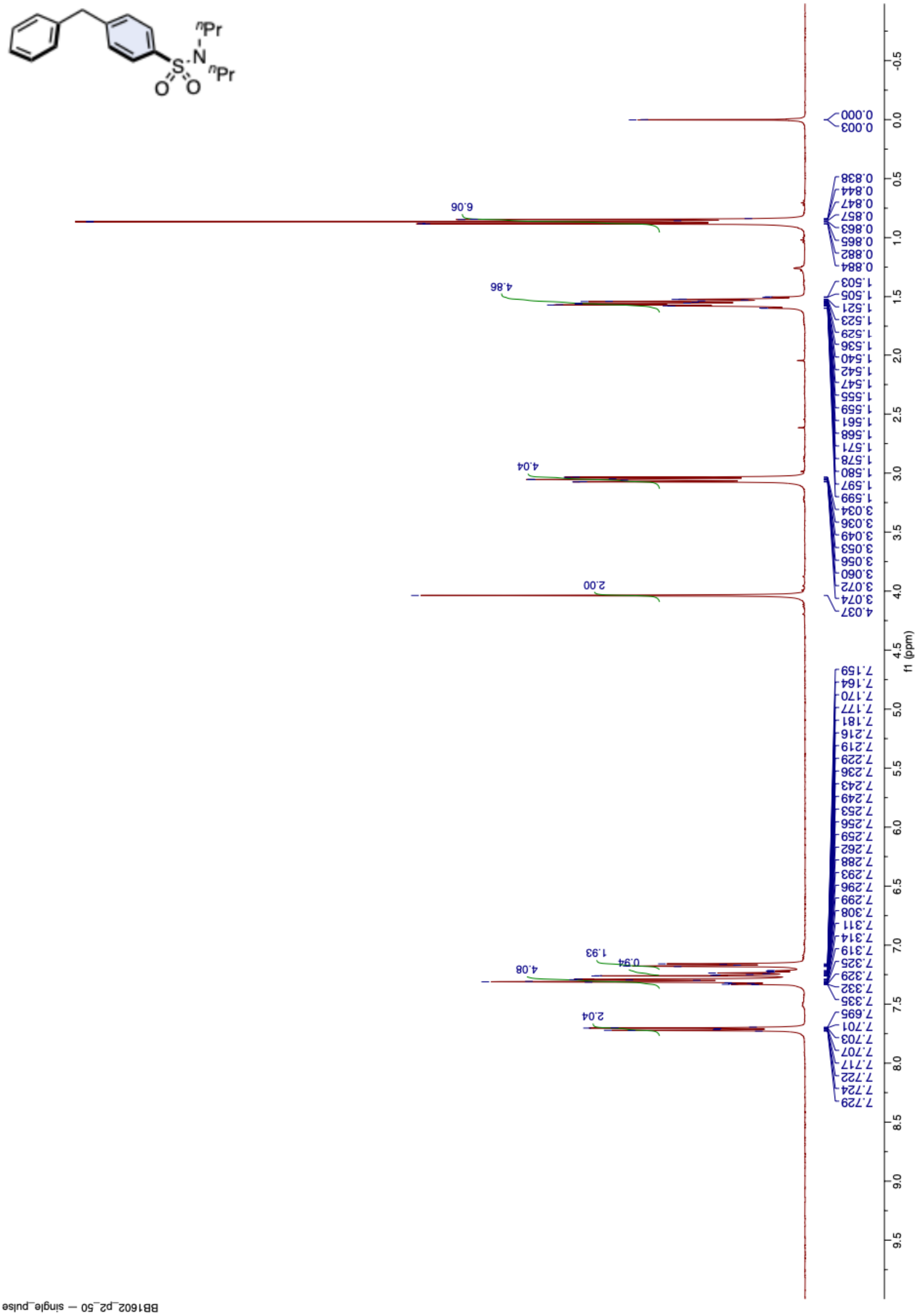

^{13}C NMR of **2V** (101 MHz, $CDCl_3$)

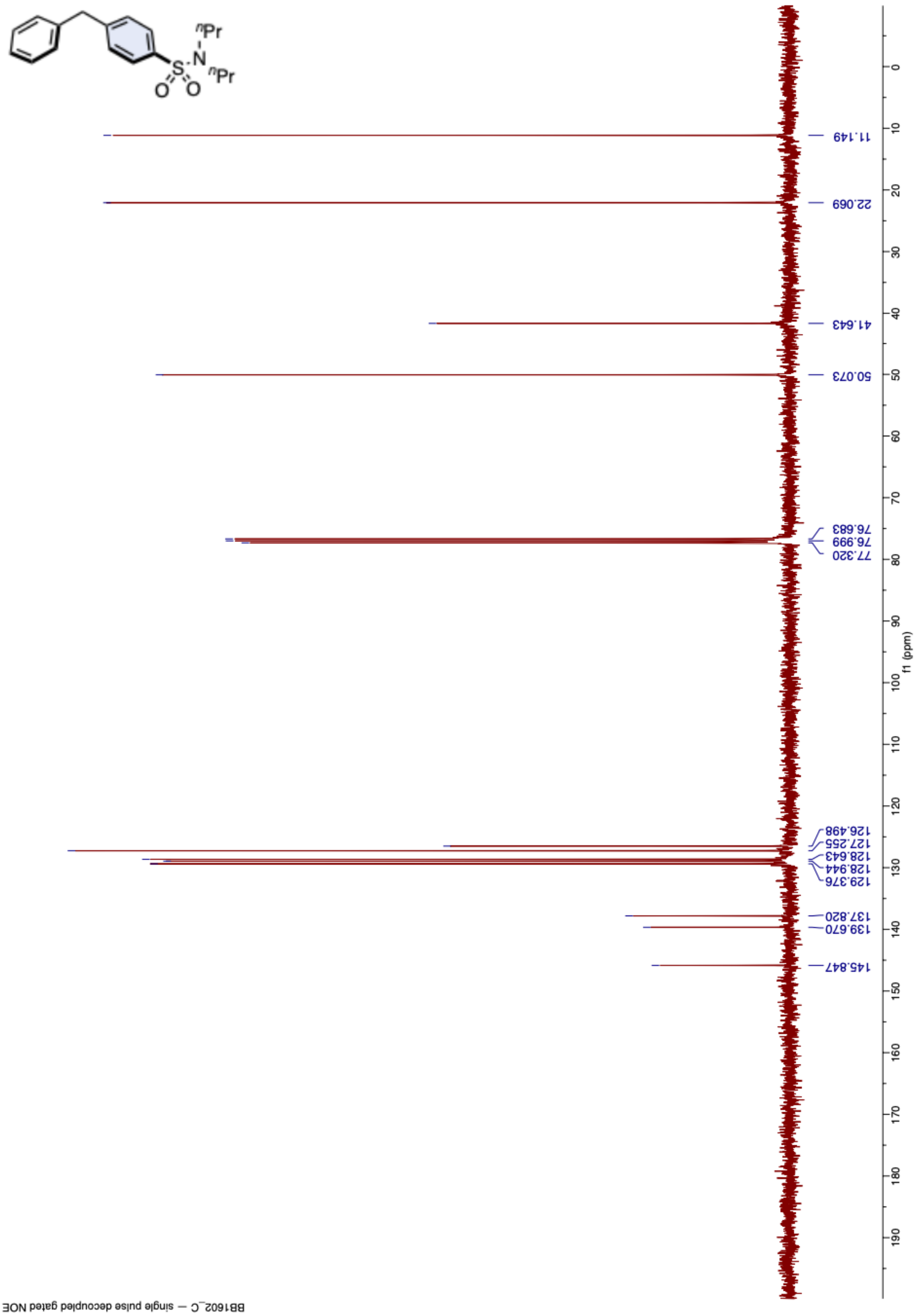

^{1}H NMR of **2W** (400 MHz, $CDCl_3$)

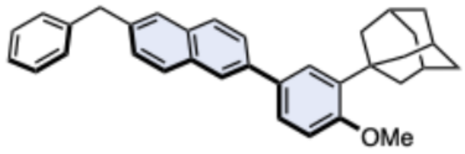

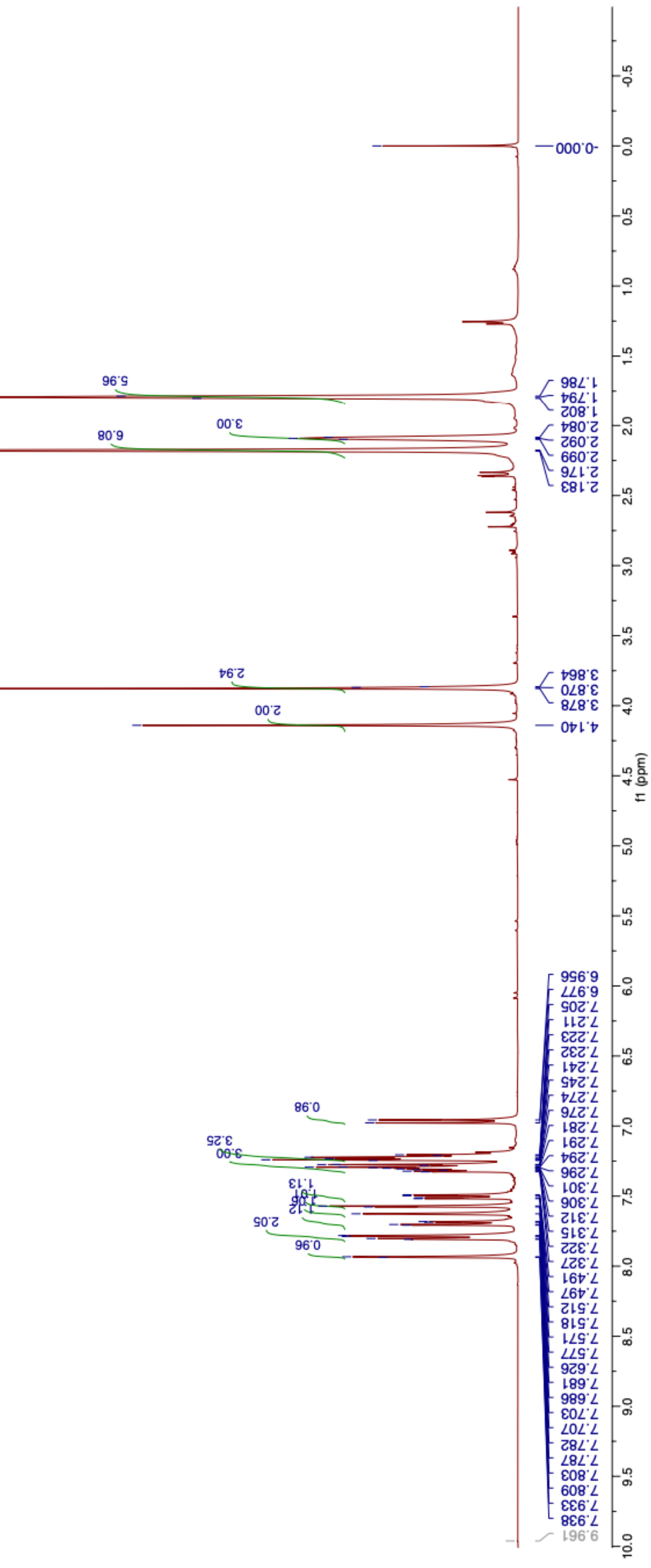

^{13}C NMR of **2W** (101 MHz, $CDCl_3$)

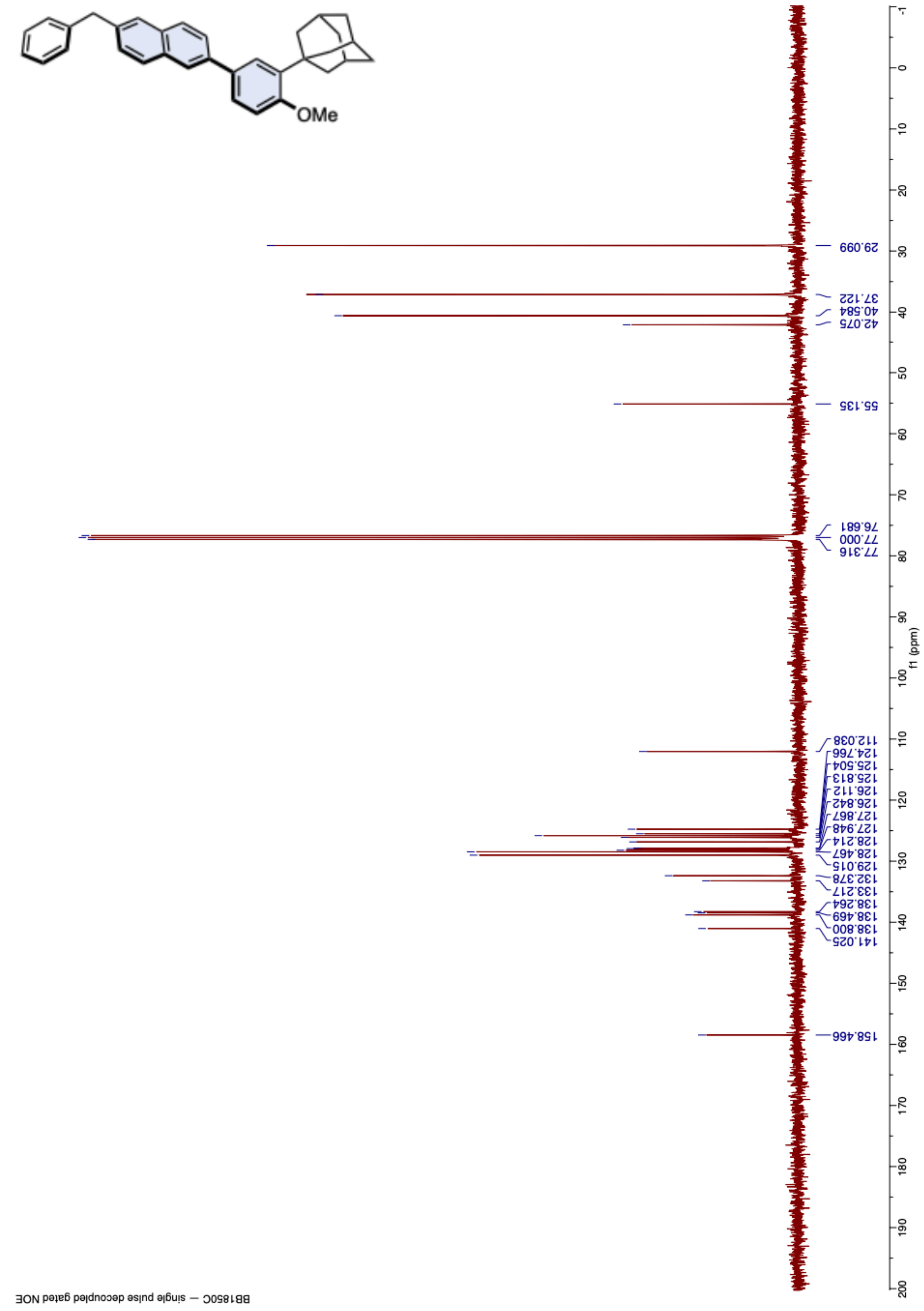

^{1}H NMR of **2X** (400 MHz, $CDCl_3$)

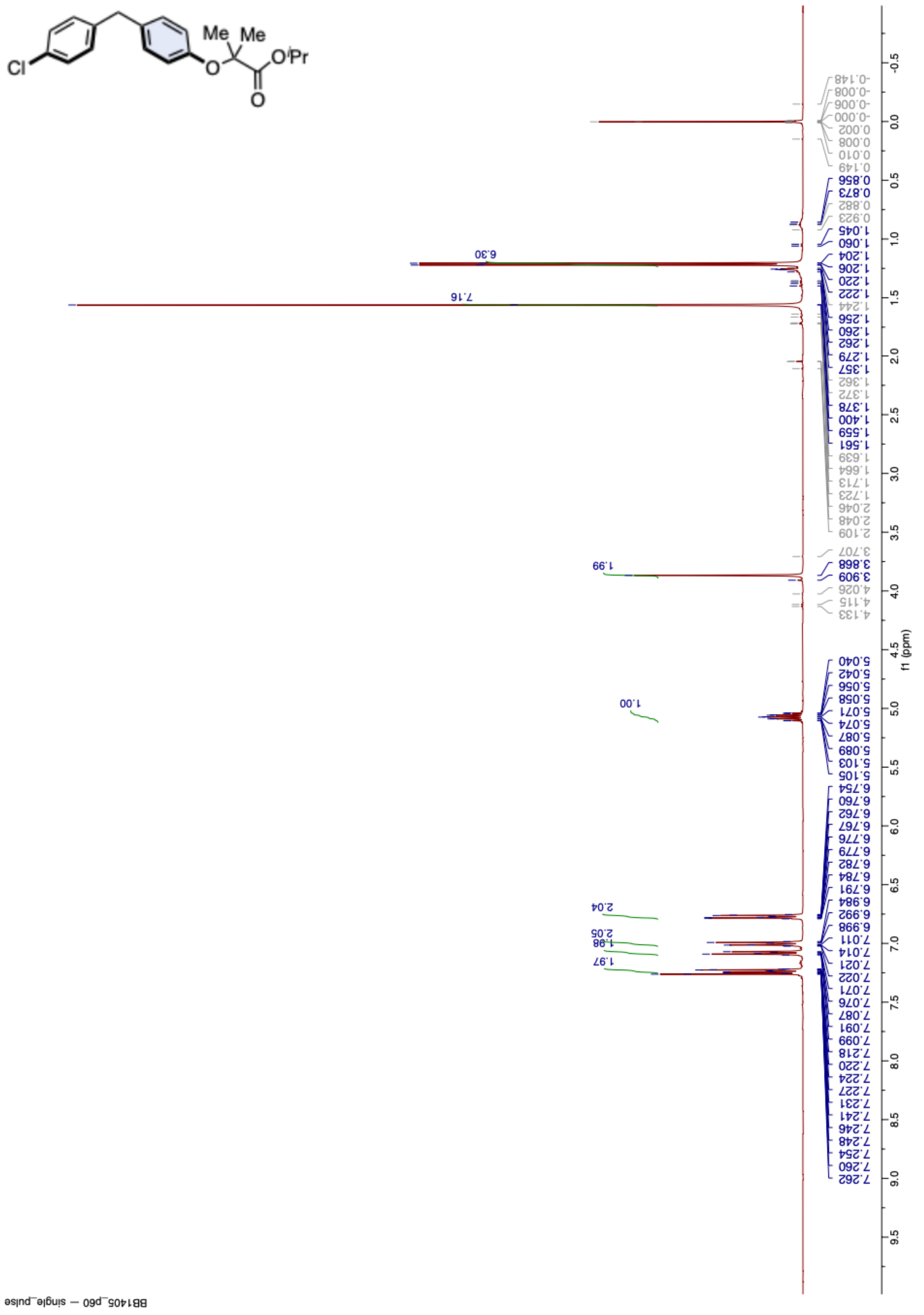

^{13}C NMR of **2X** (101 MHz, $CDCl_3$)

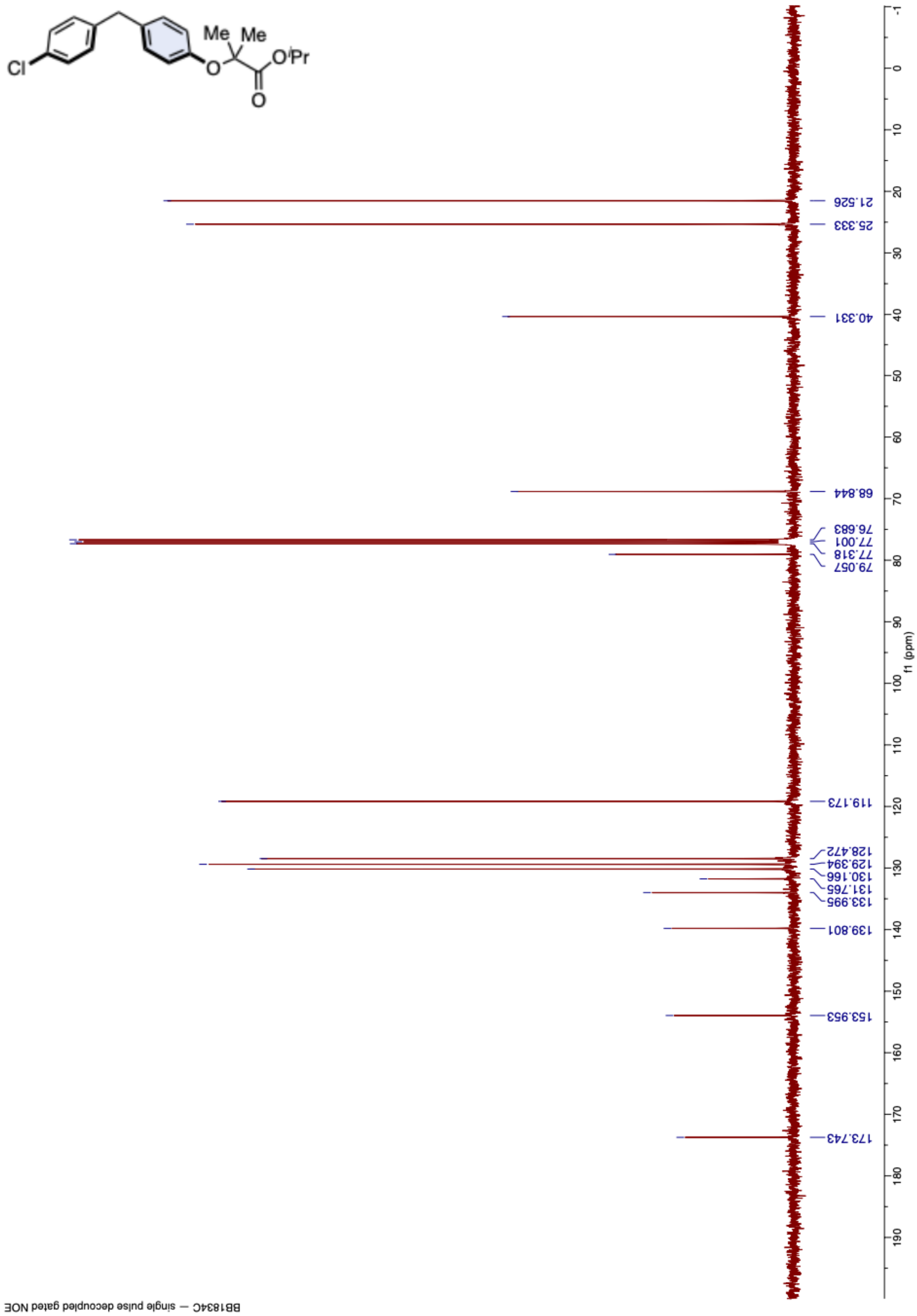

^{1}H NMR of **3A** (400 MHz, $CDCl_3$)

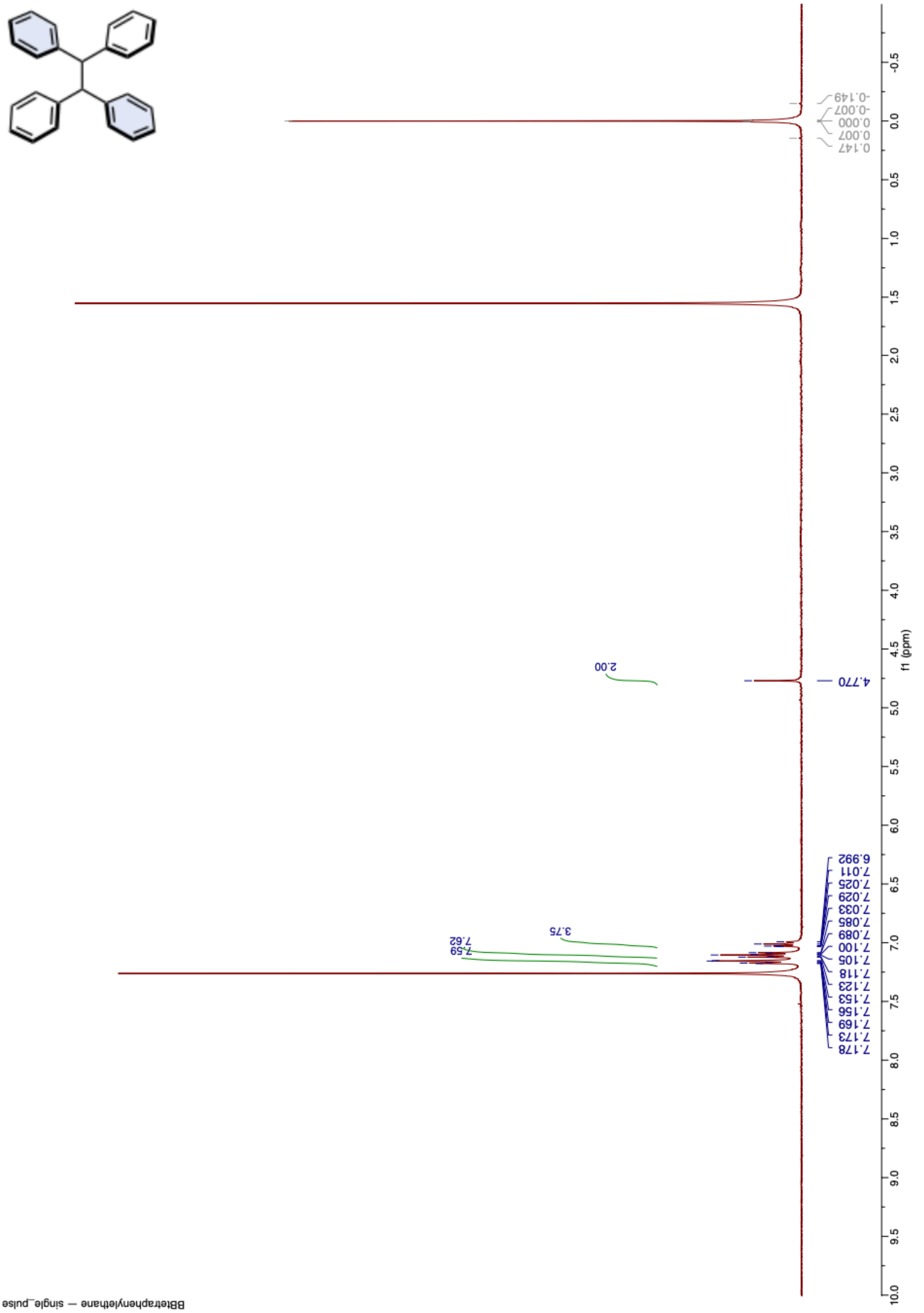

^{13}C NMR of **3A** (101 MHz, $CDCl_3$)

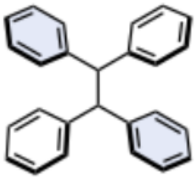

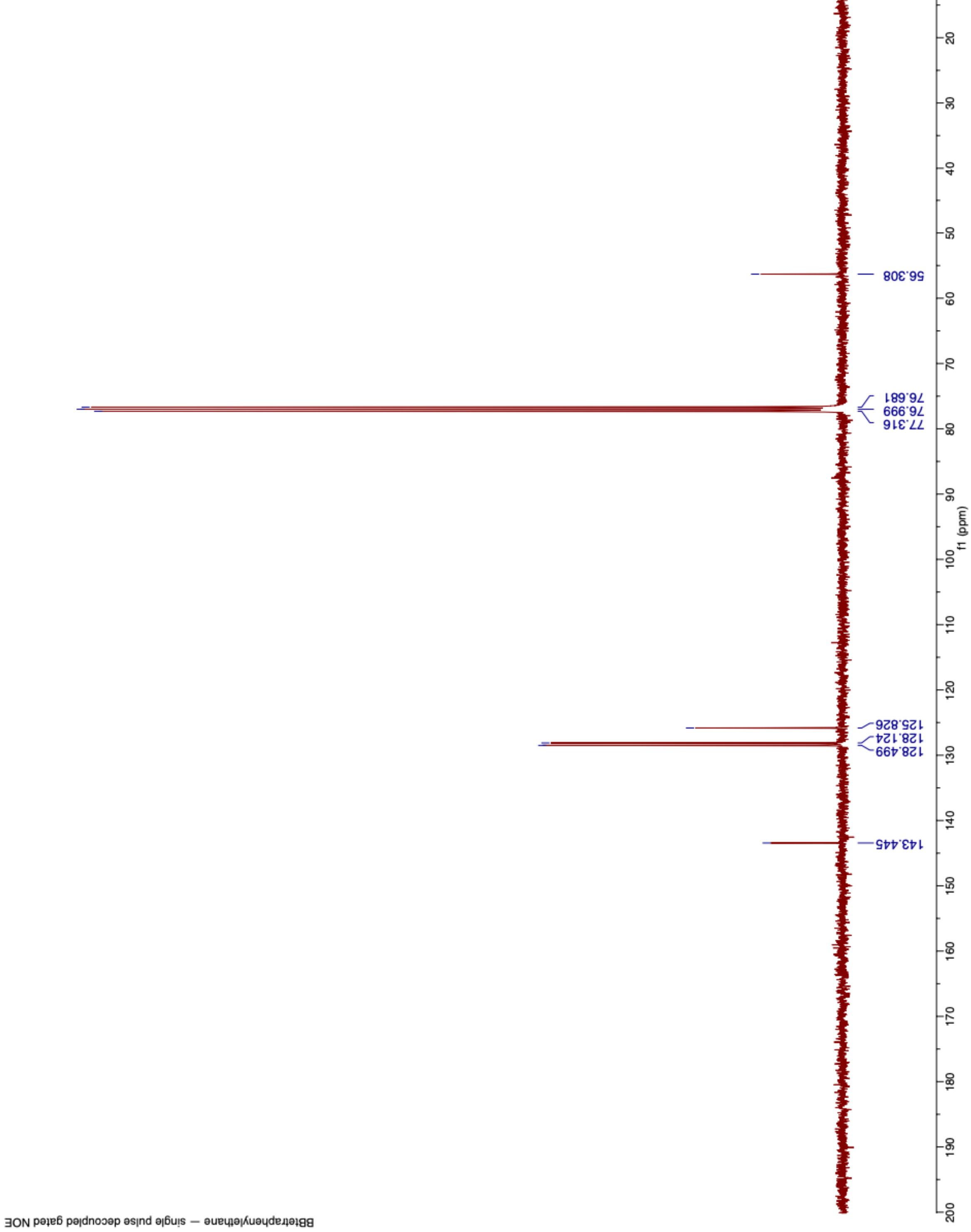

^{1}H NMR of **3B** (400 MHz, $CDCl_3$)

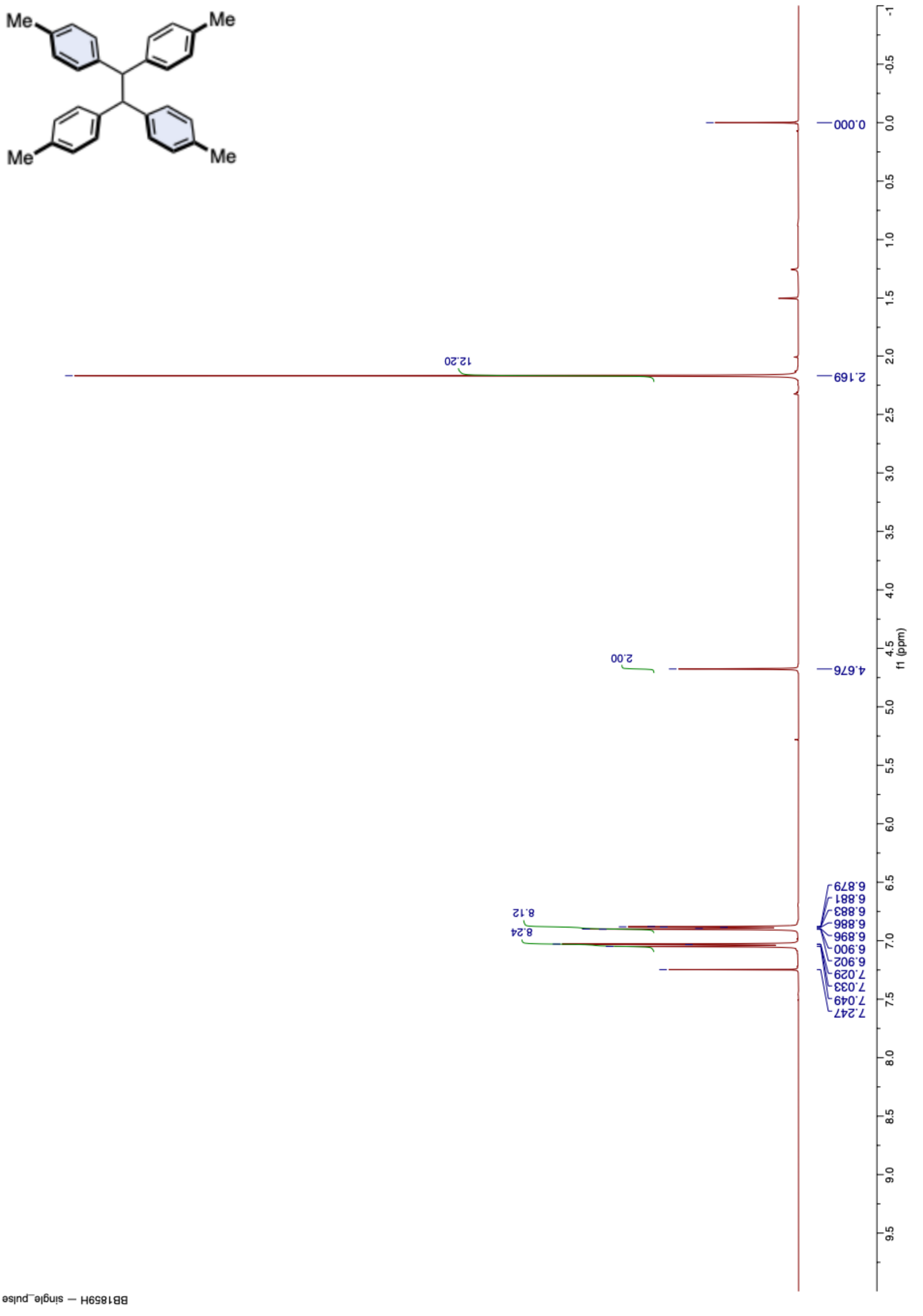

^{13}C NMR of **3B** (101 MHz, $CDCl_3$)

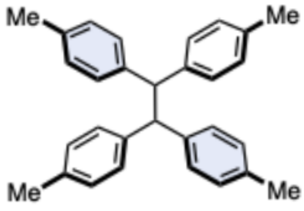

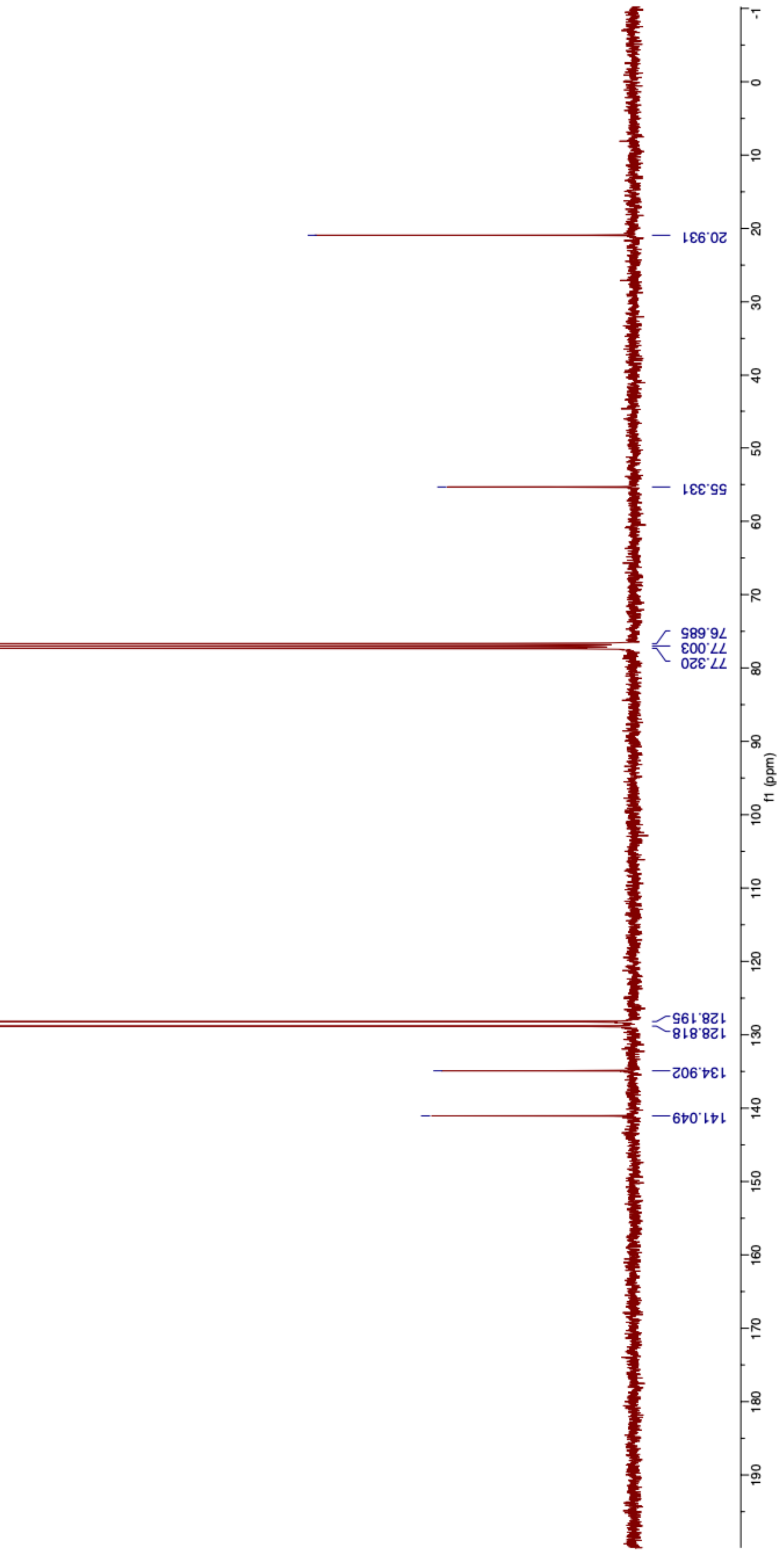

^{1}H NMR of **3C** (400 MHz, $CDCl_3$)

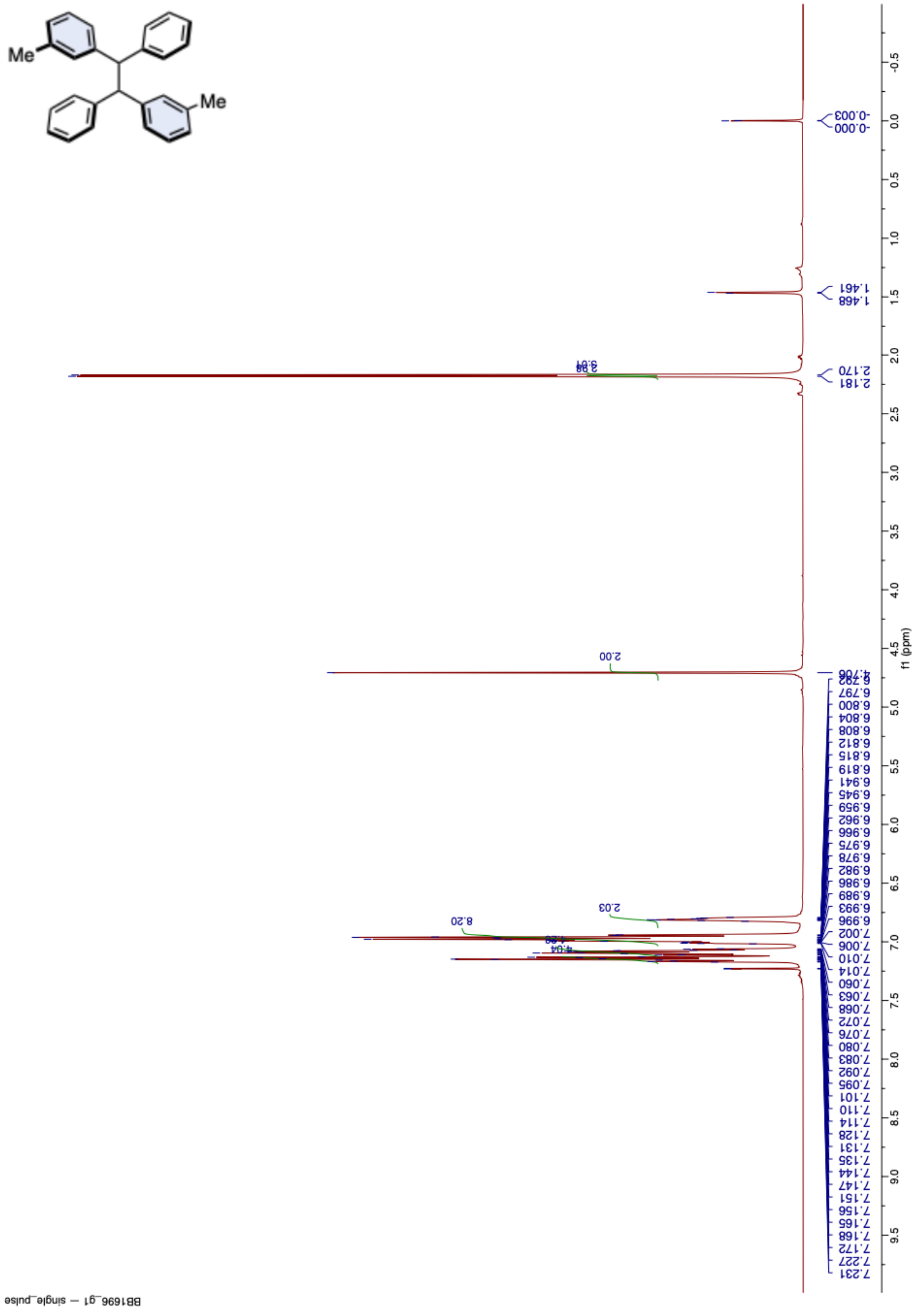

^{13}C NMR of **3C** (101 MHz, $CDCl_3$)

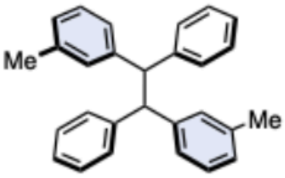

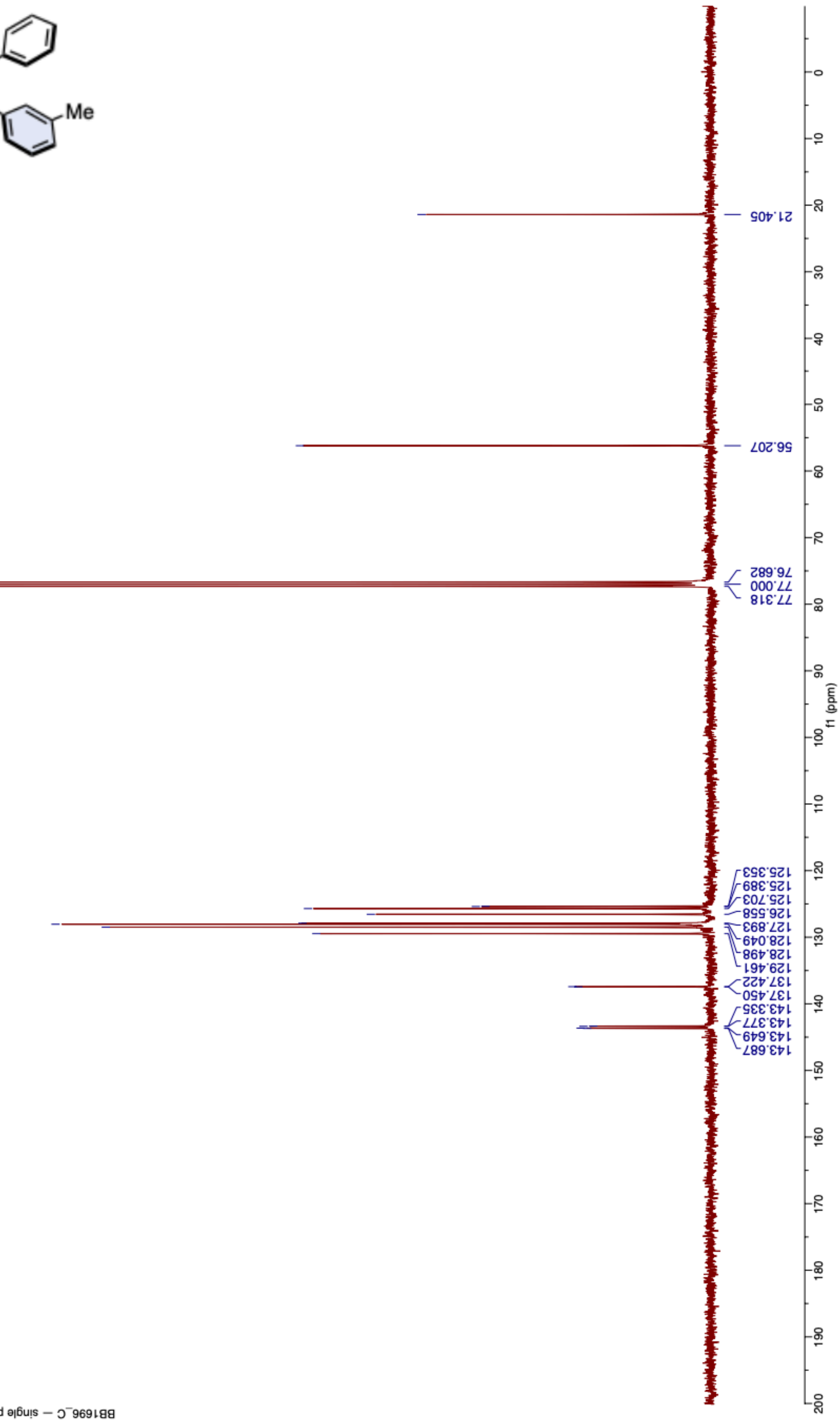

^{1}H NMR of **3D** (400 MHz, $CDCl_3$)

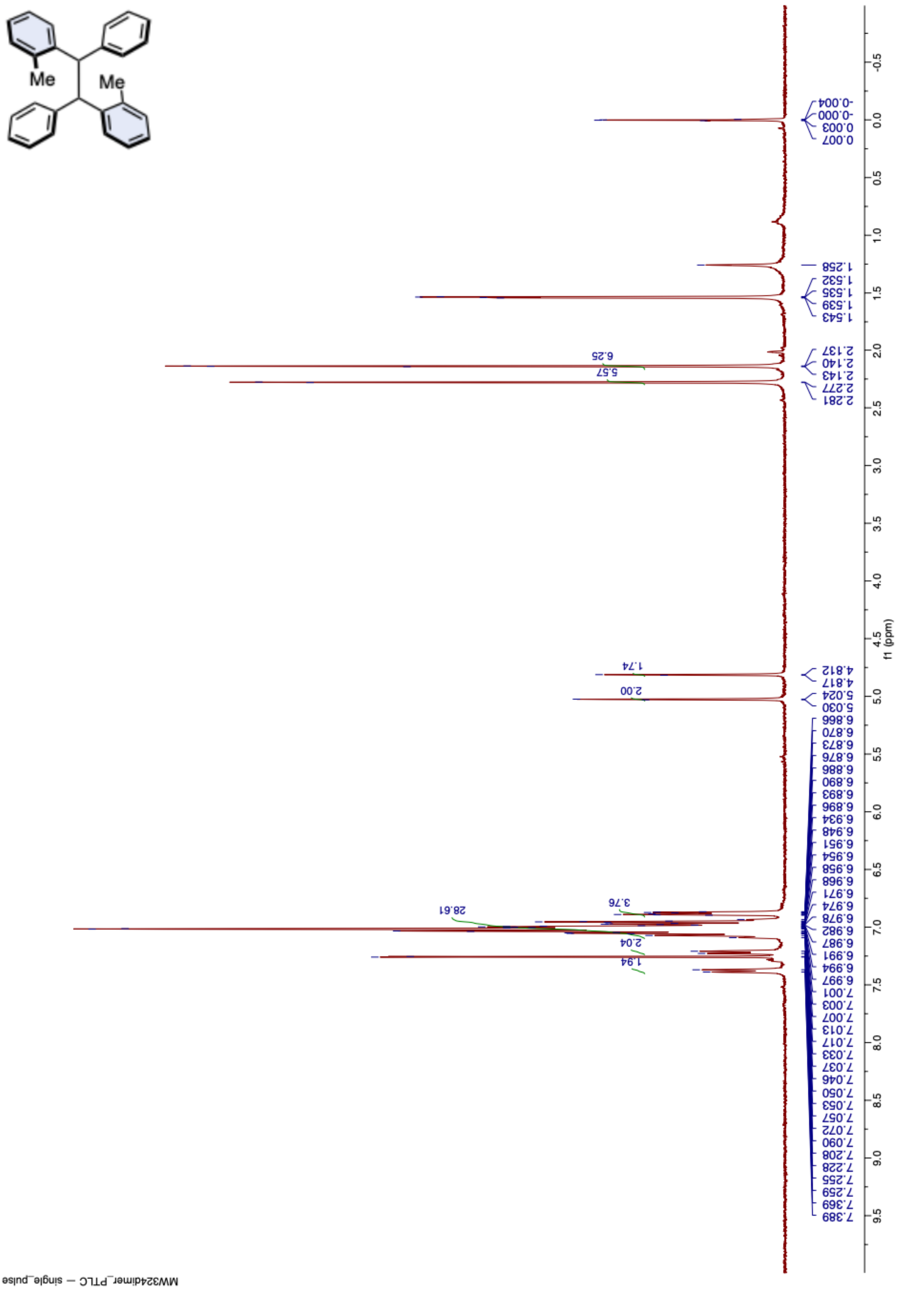

^{13}C NMR of **3D** (101 MHz, $CDCl_3$)

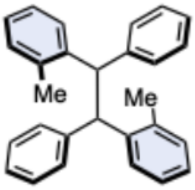

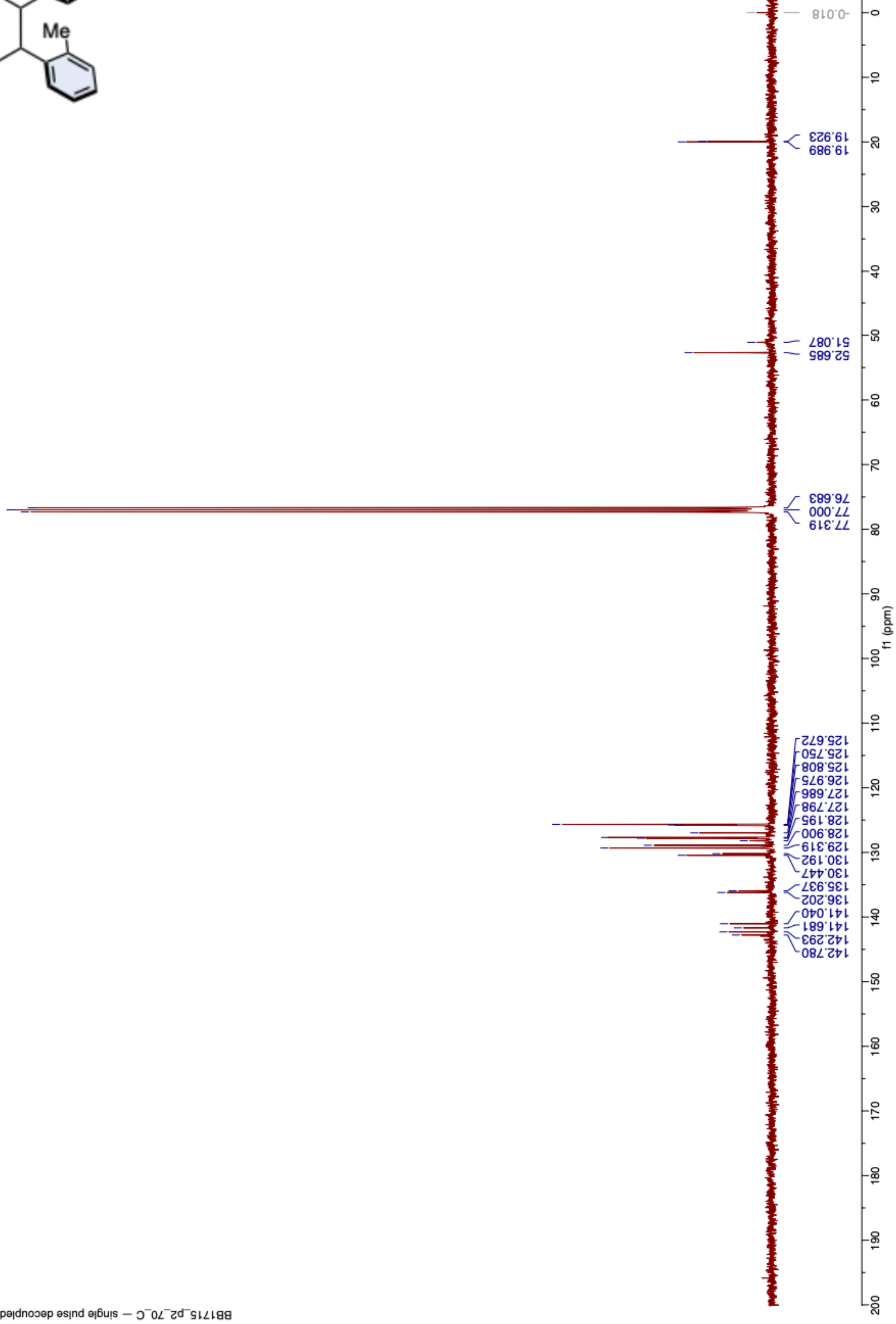

^{1}H NMR of **3E** (400 MHz, $CDCl_3$)

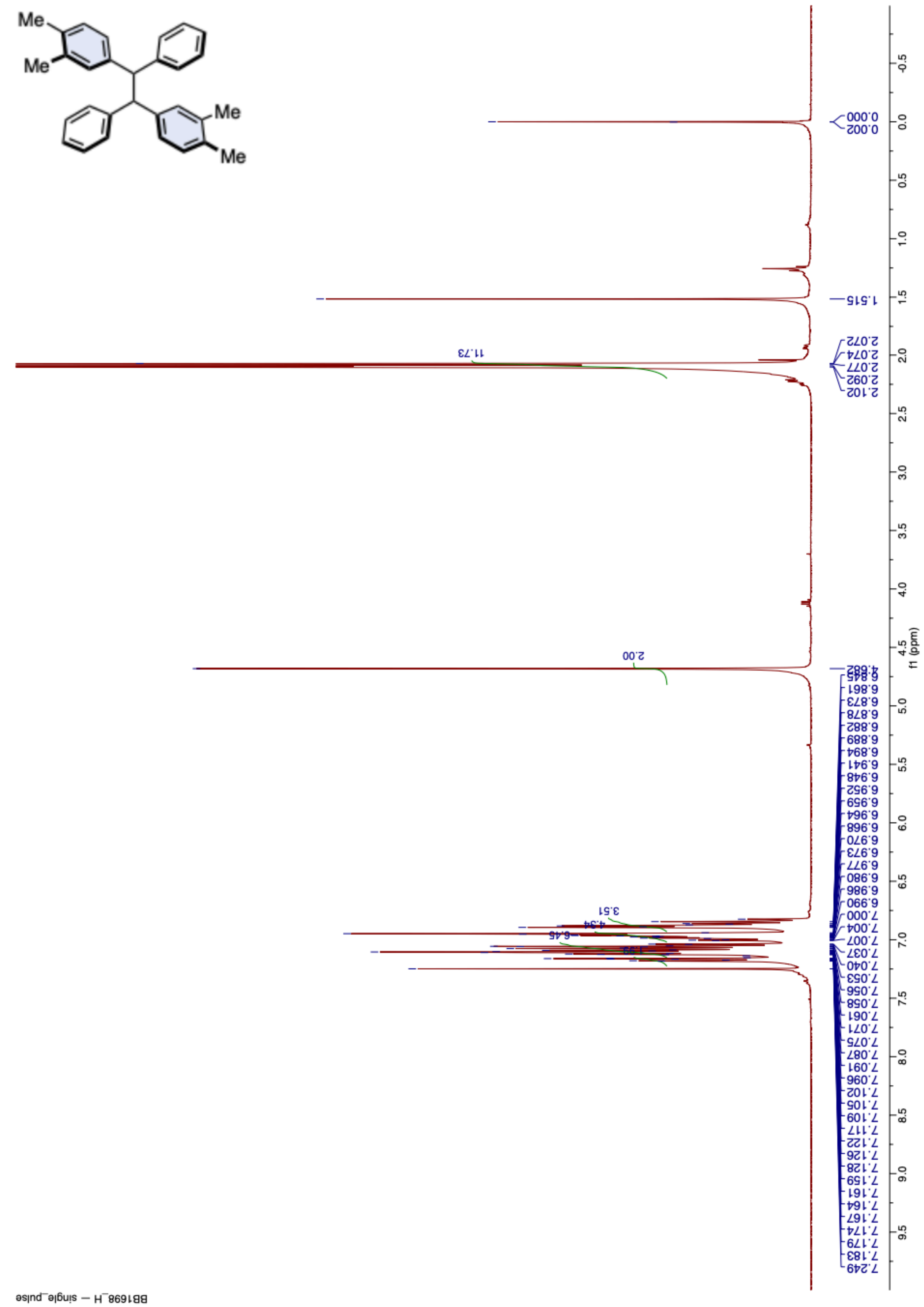

^{13}C NMR of **3E** (101 MHz, $CDCl_3$)

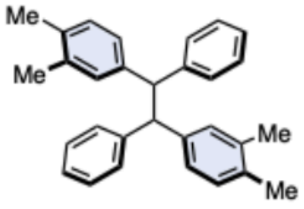

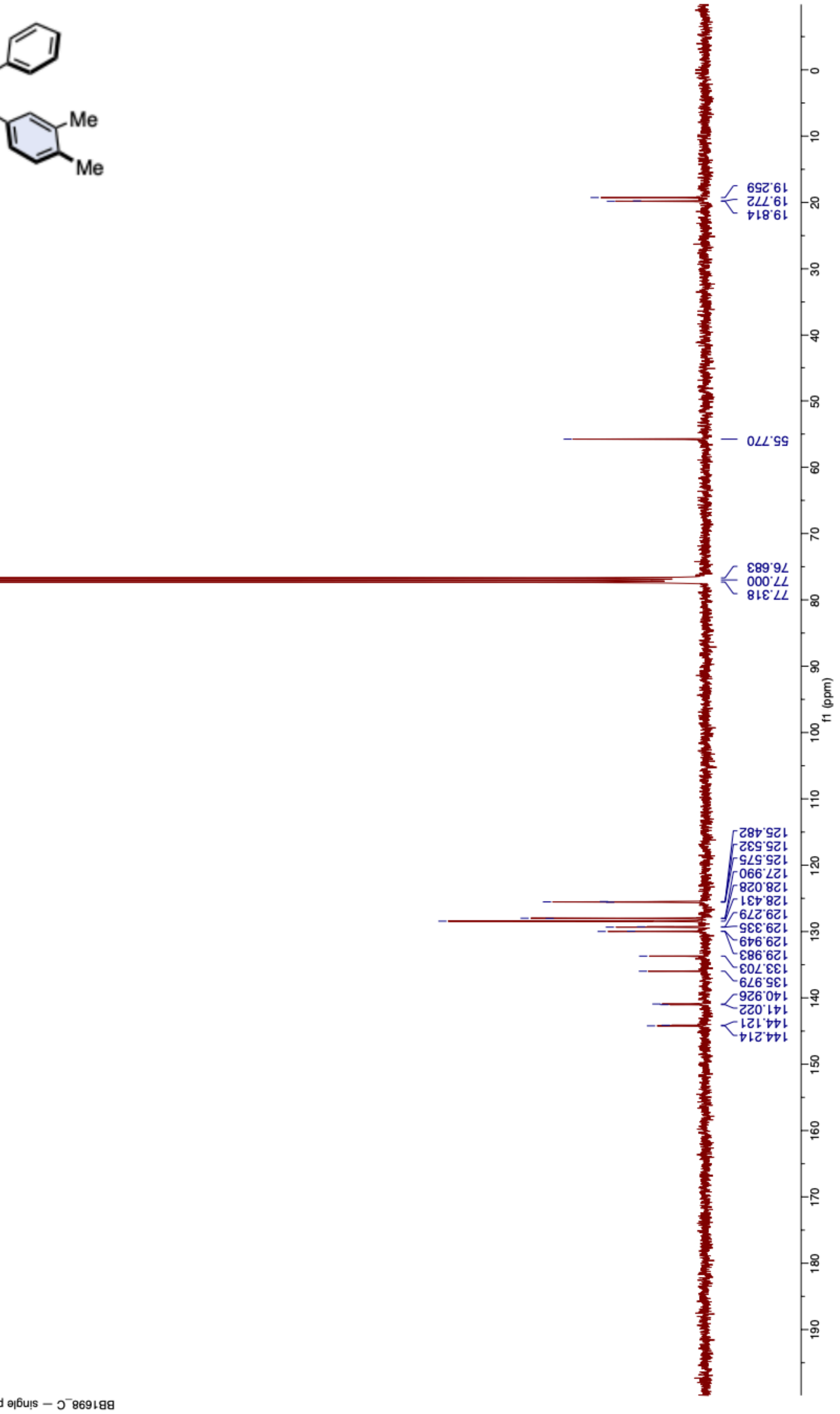

^{1}H NMR of **3F** (400 MHz, $CDCl_3$)

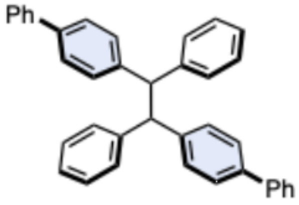

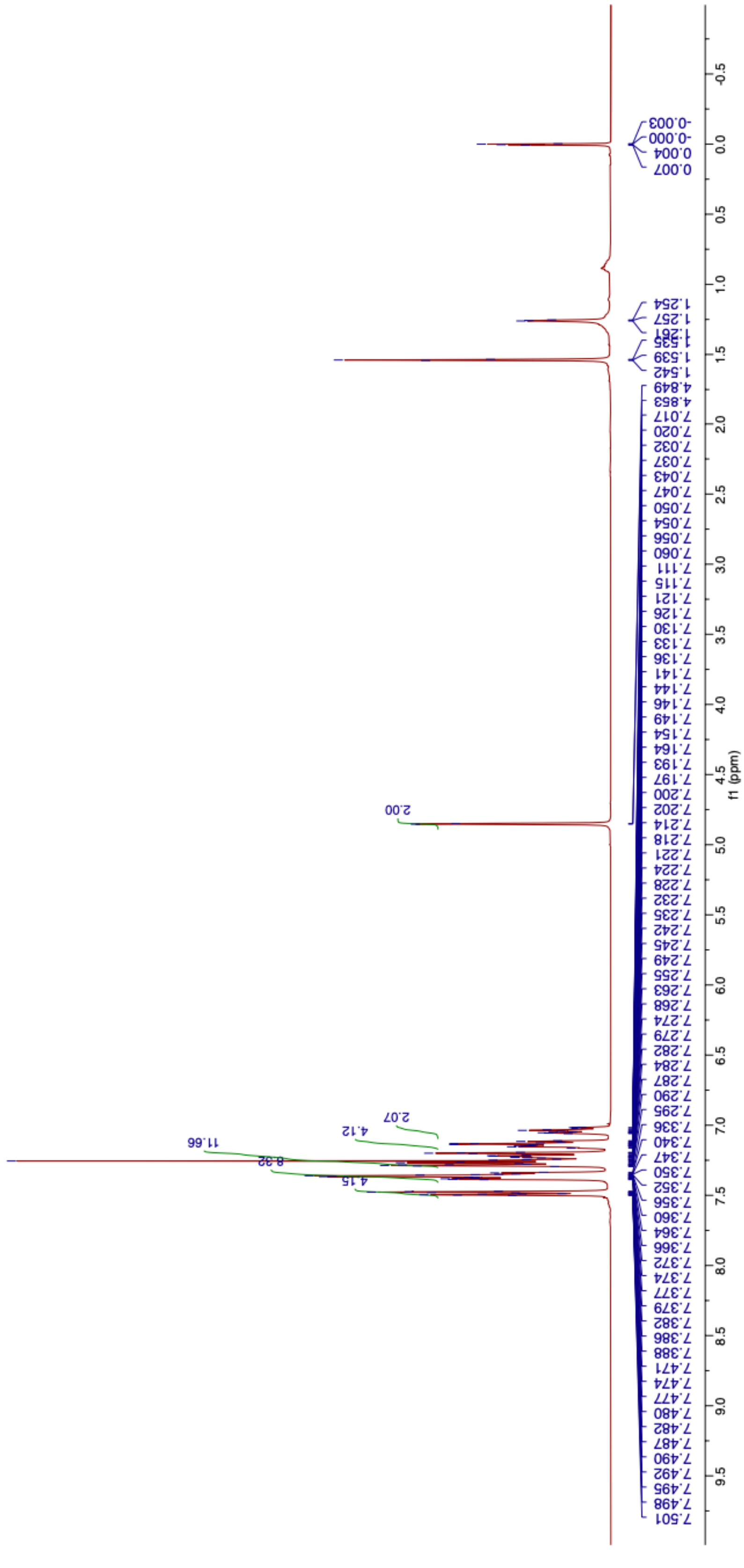

^{13}C NMR of **3F** (101 MHz, $CDCl_3$)

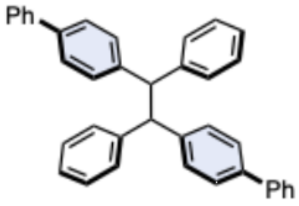

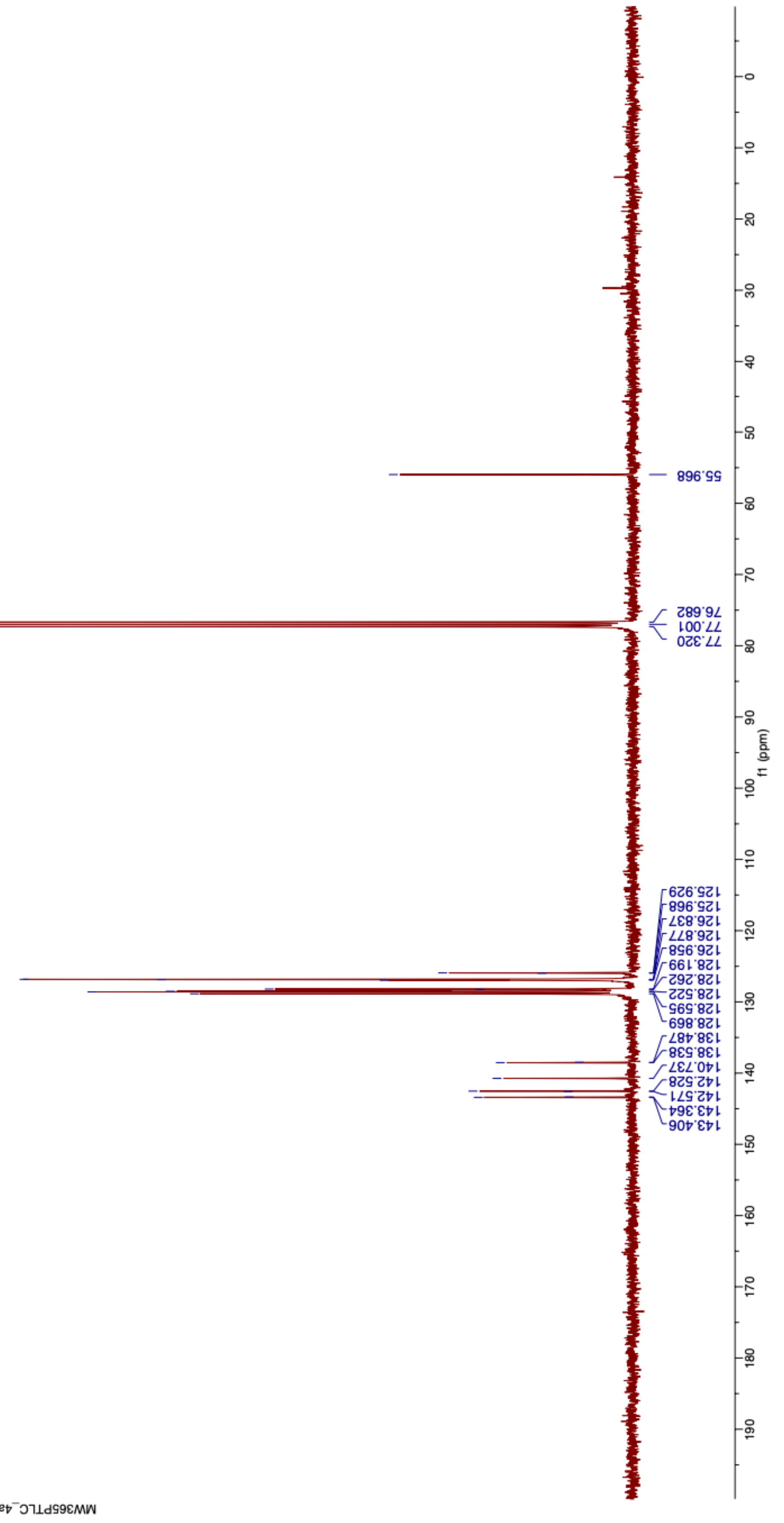

^{1}H NMR of **3G** (400 MHz, $CDCl_3$)

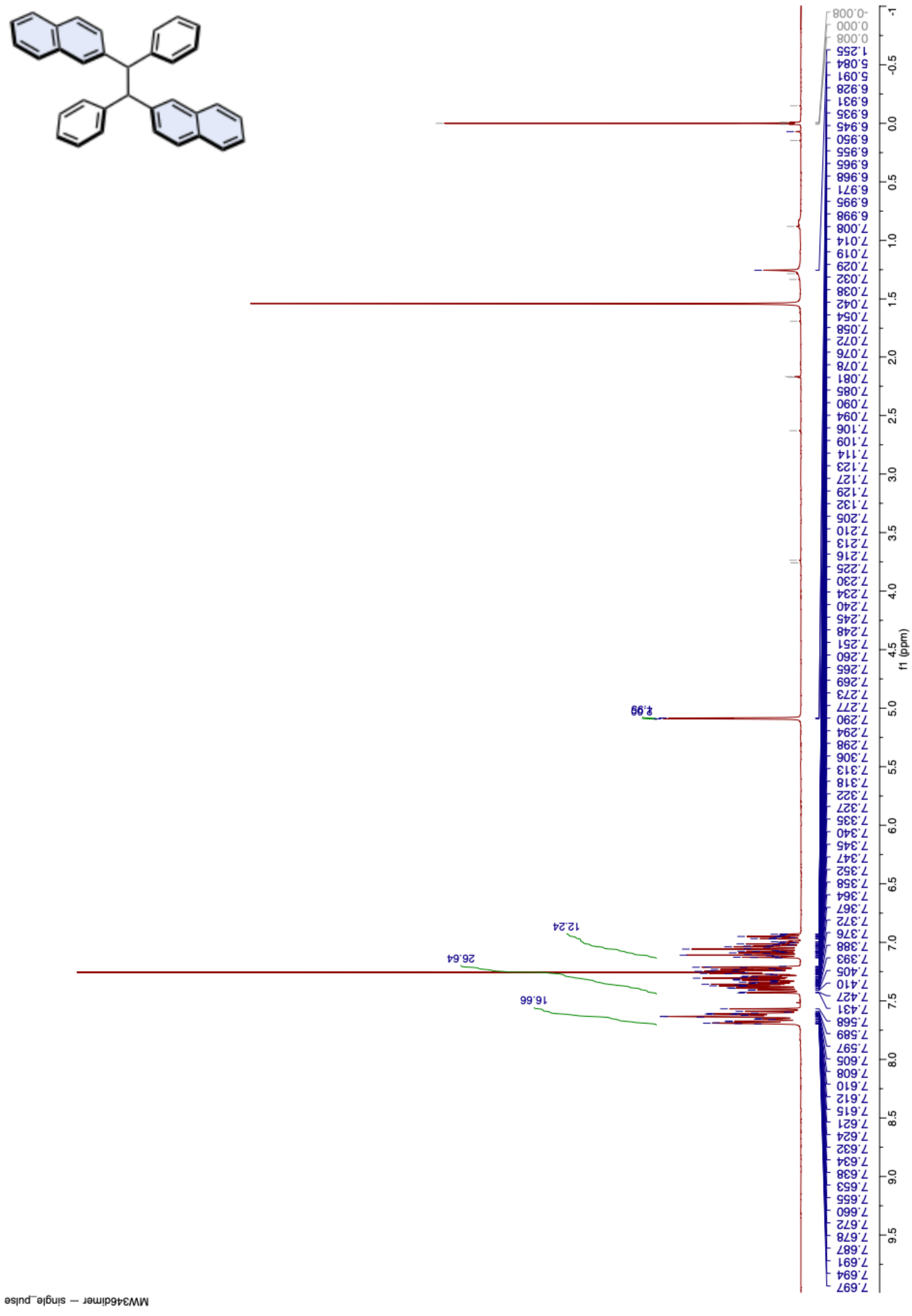

^{13}C NMR of **3G** (101 MHz, $CDCl_3$)

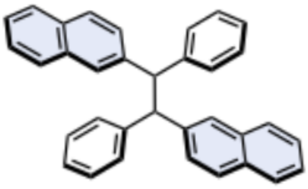

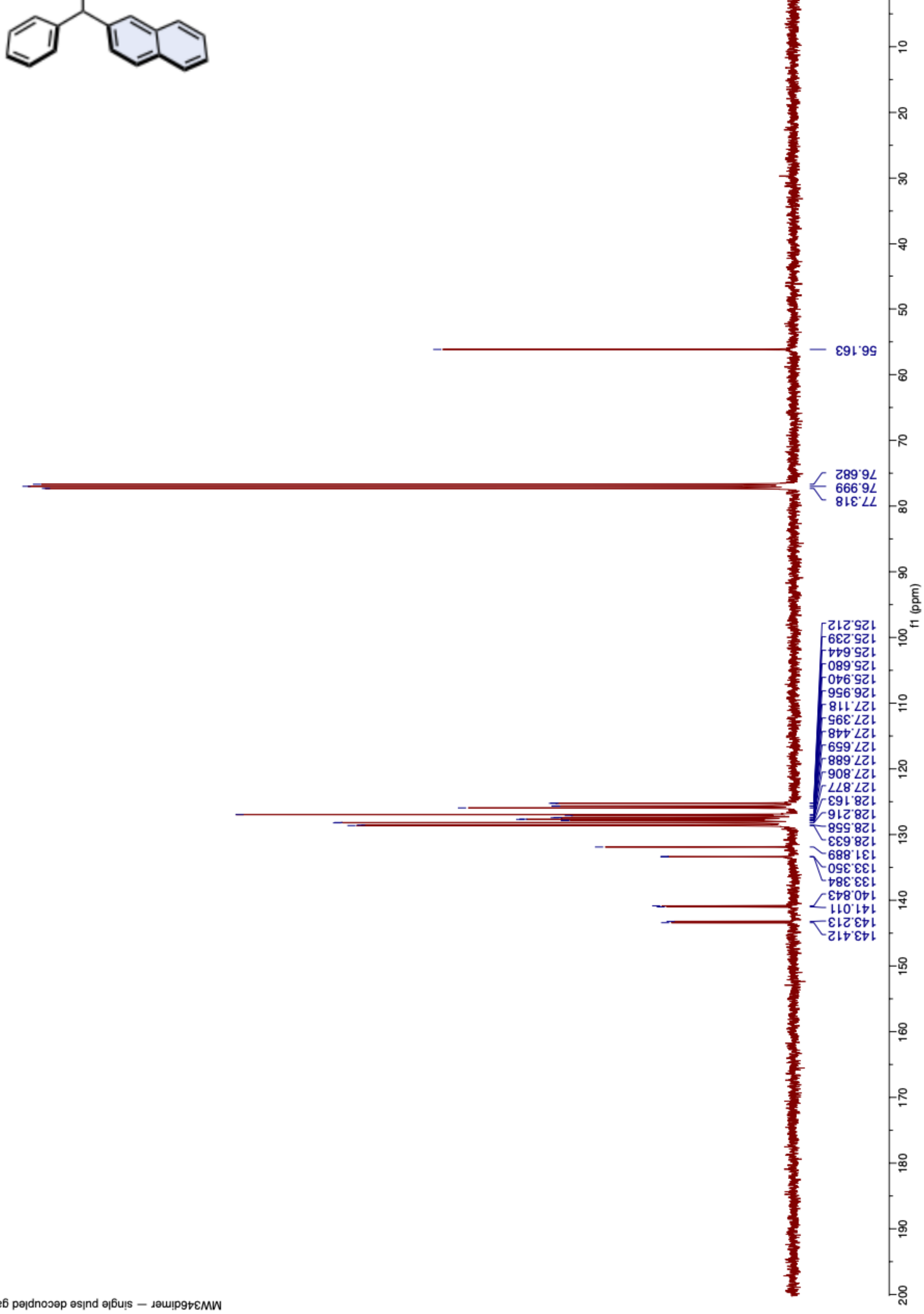

^{1}H NMR of **3H** (400 MHz, $CDCl_3$)

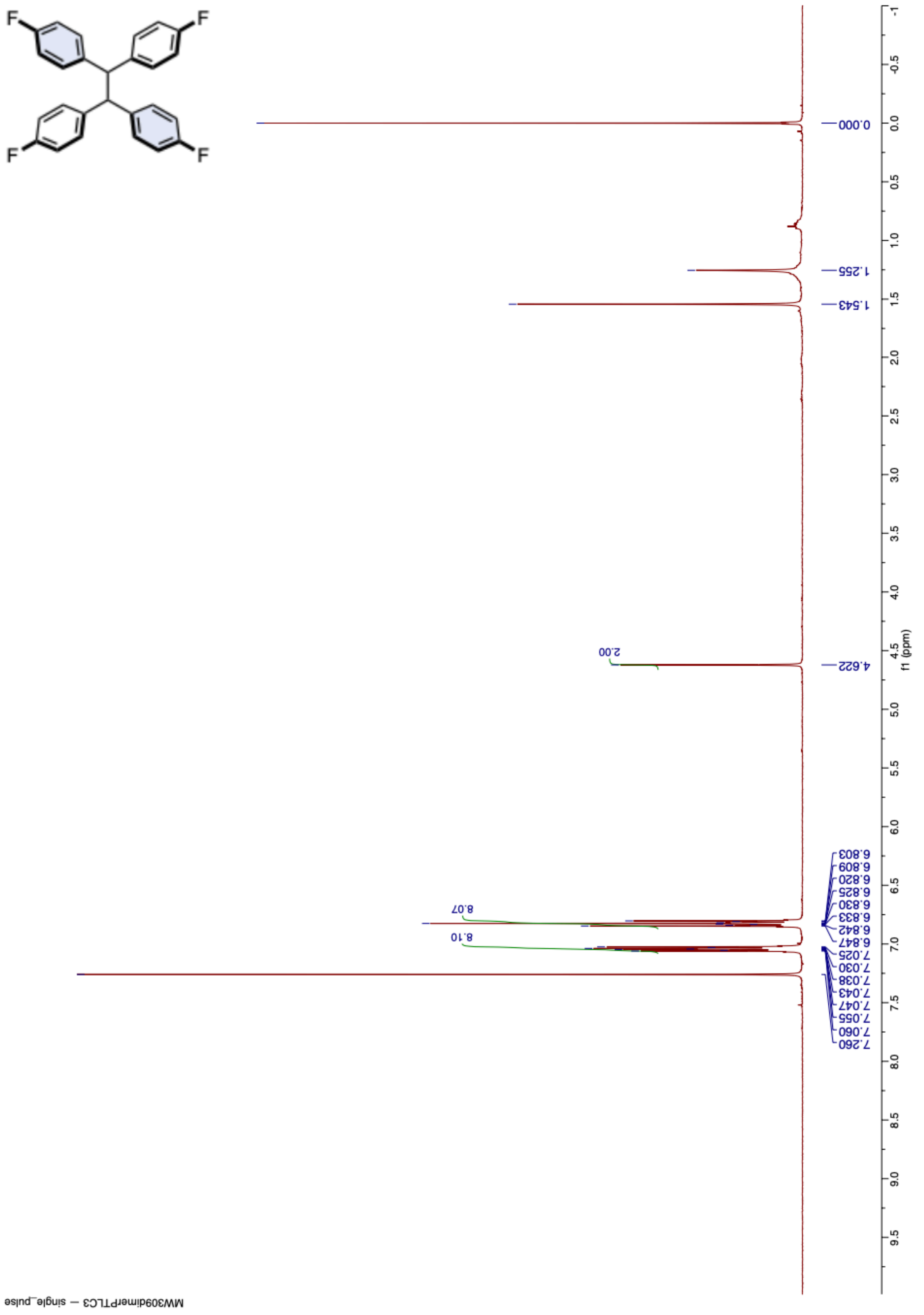

^{13}C NMR of **3H** (101 MHz, $CDCl_3$)

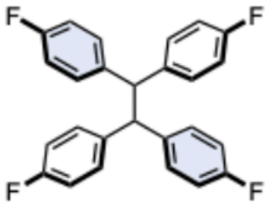

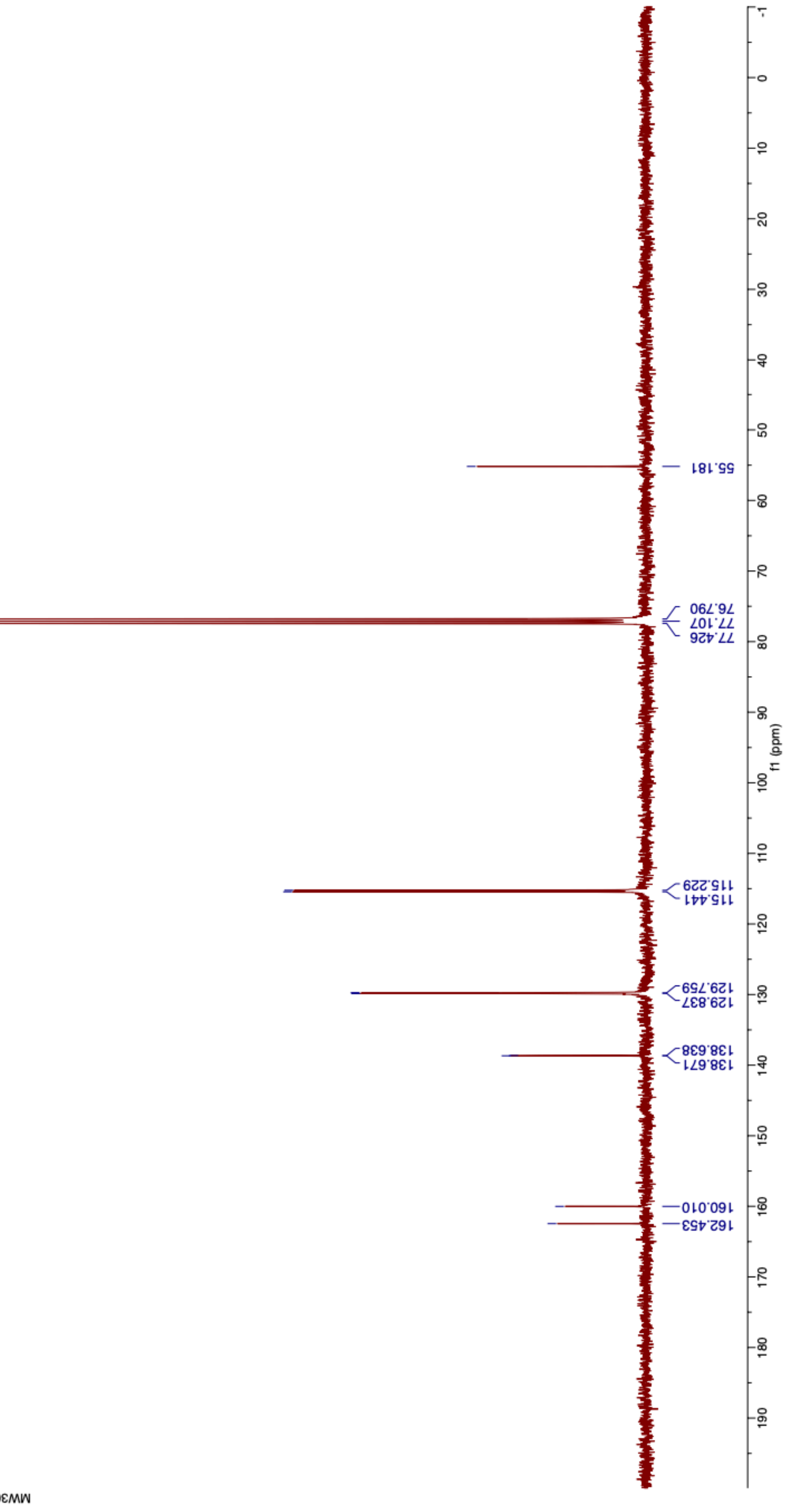

^{19}F NMR of **3H** (376 MHz, $CDCl_3$)

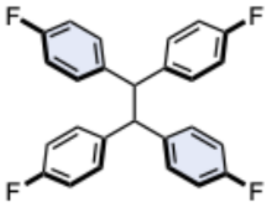

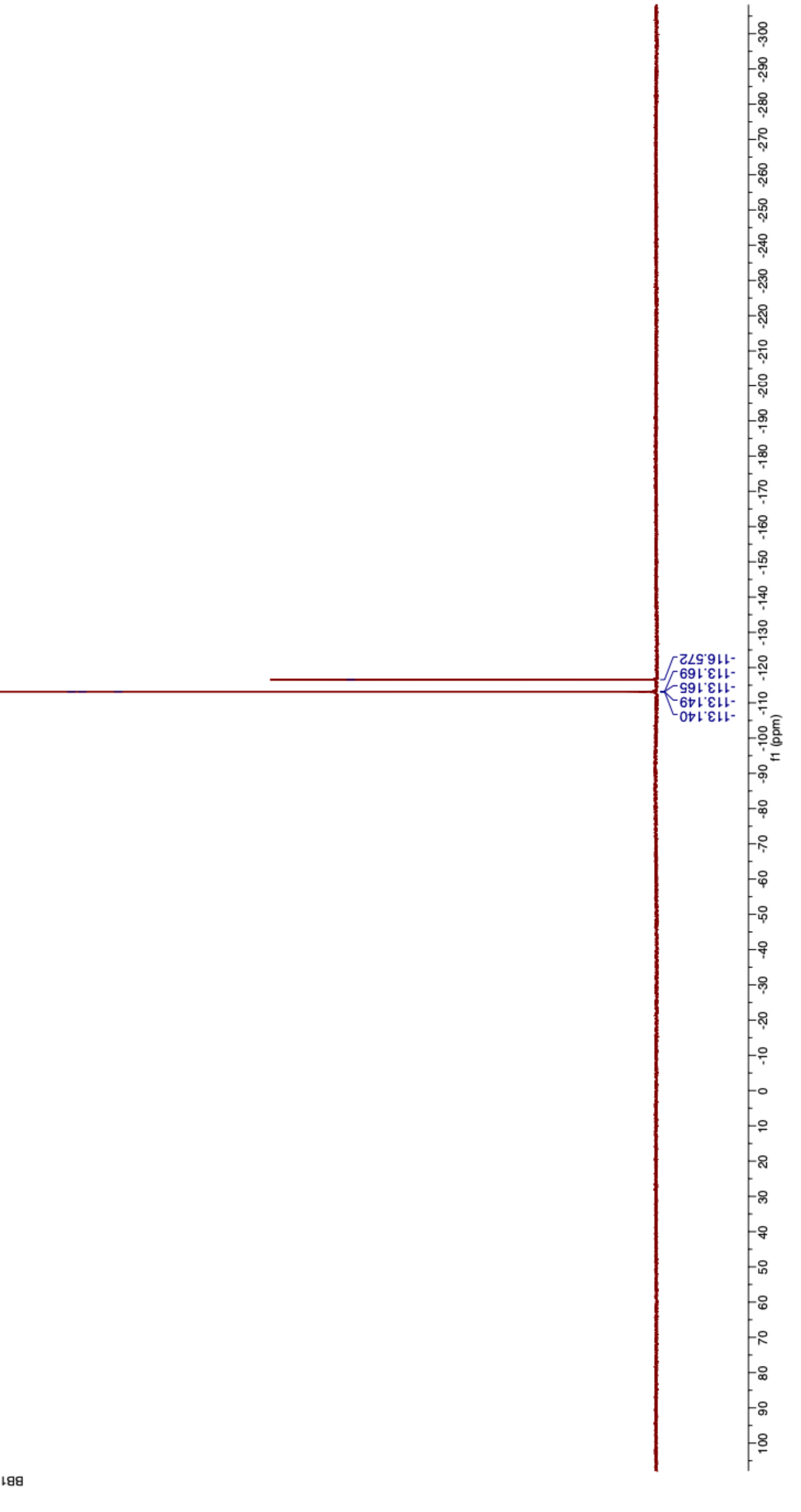

^{1}H NMR of **3l** (400 MHz, $CDCl_3$)

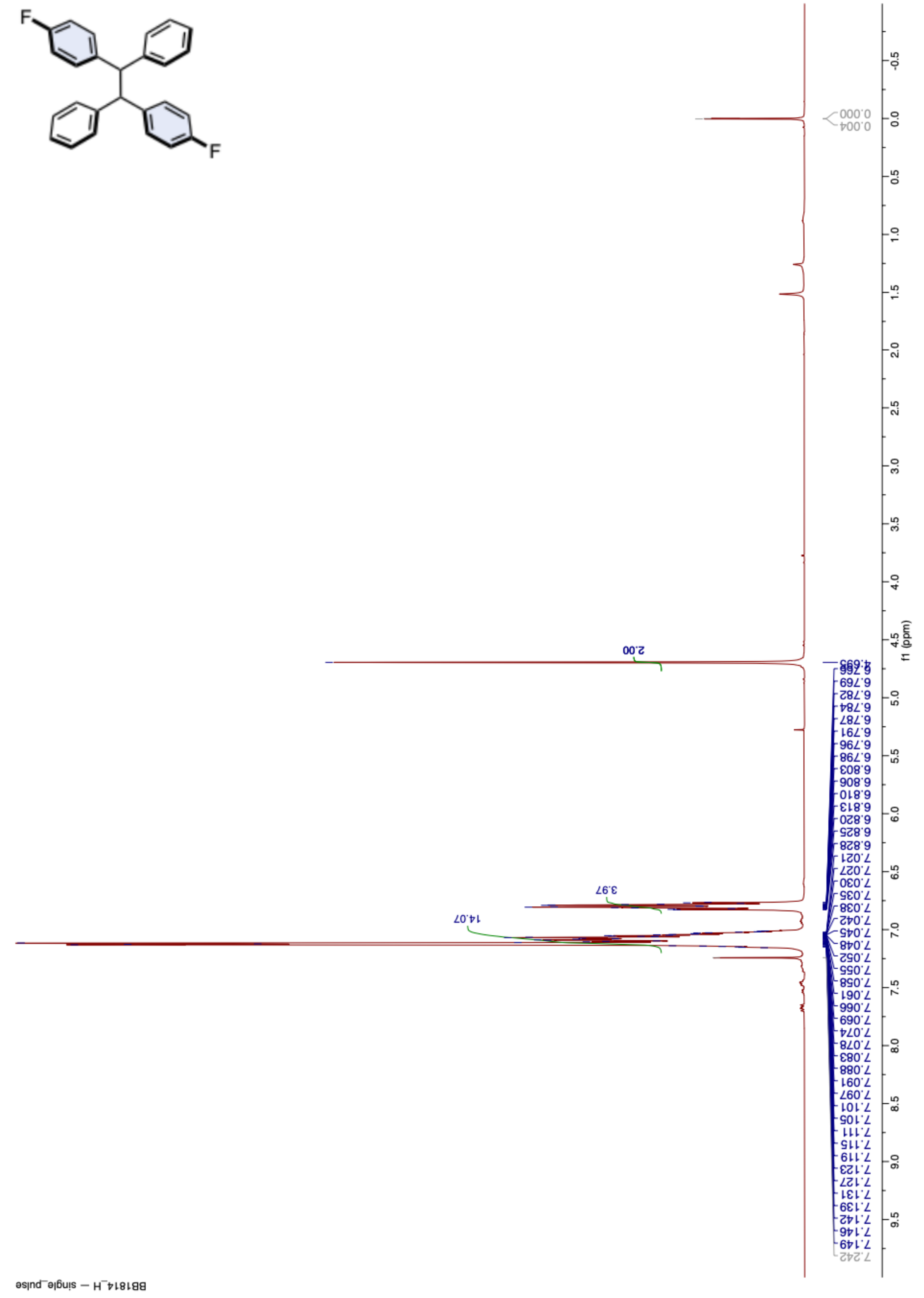

^{13}C NMR of **3l** (101 MHz, $CDCl_3$)

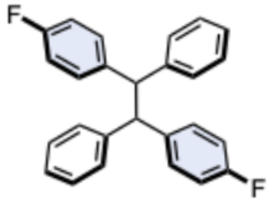

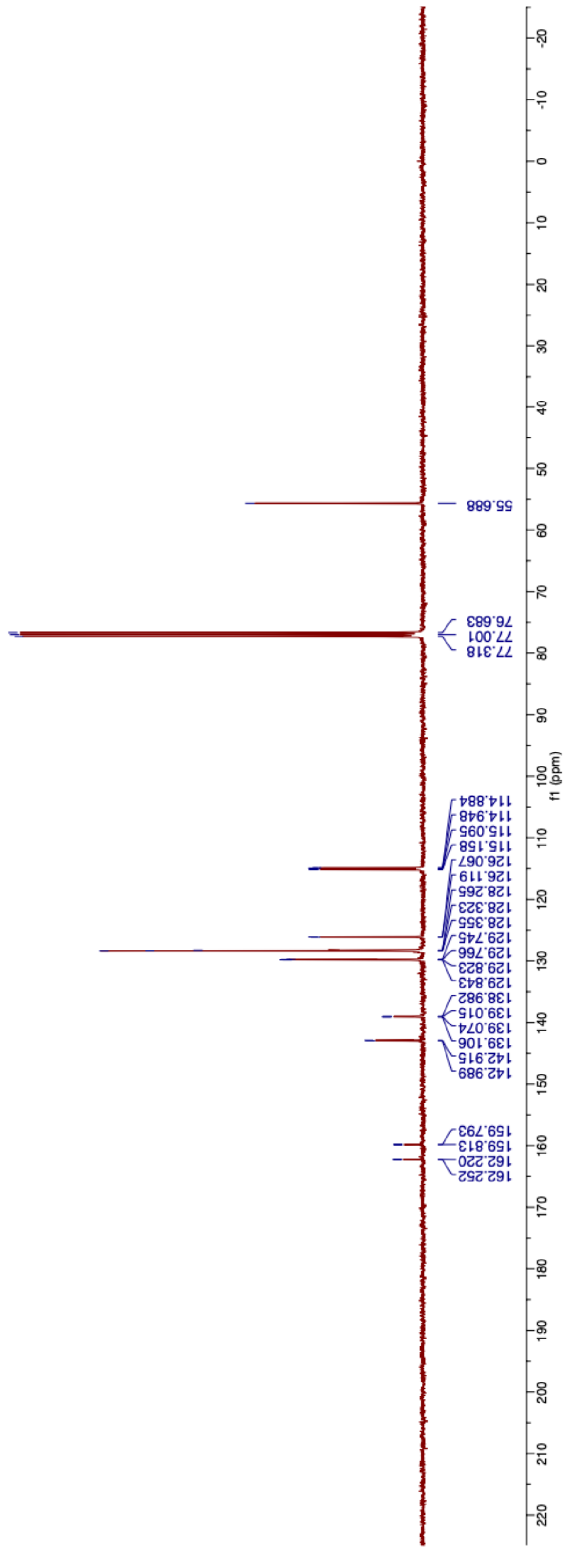

^{19}F NMR of **3l** (376 MHz, $CDCl_3$)

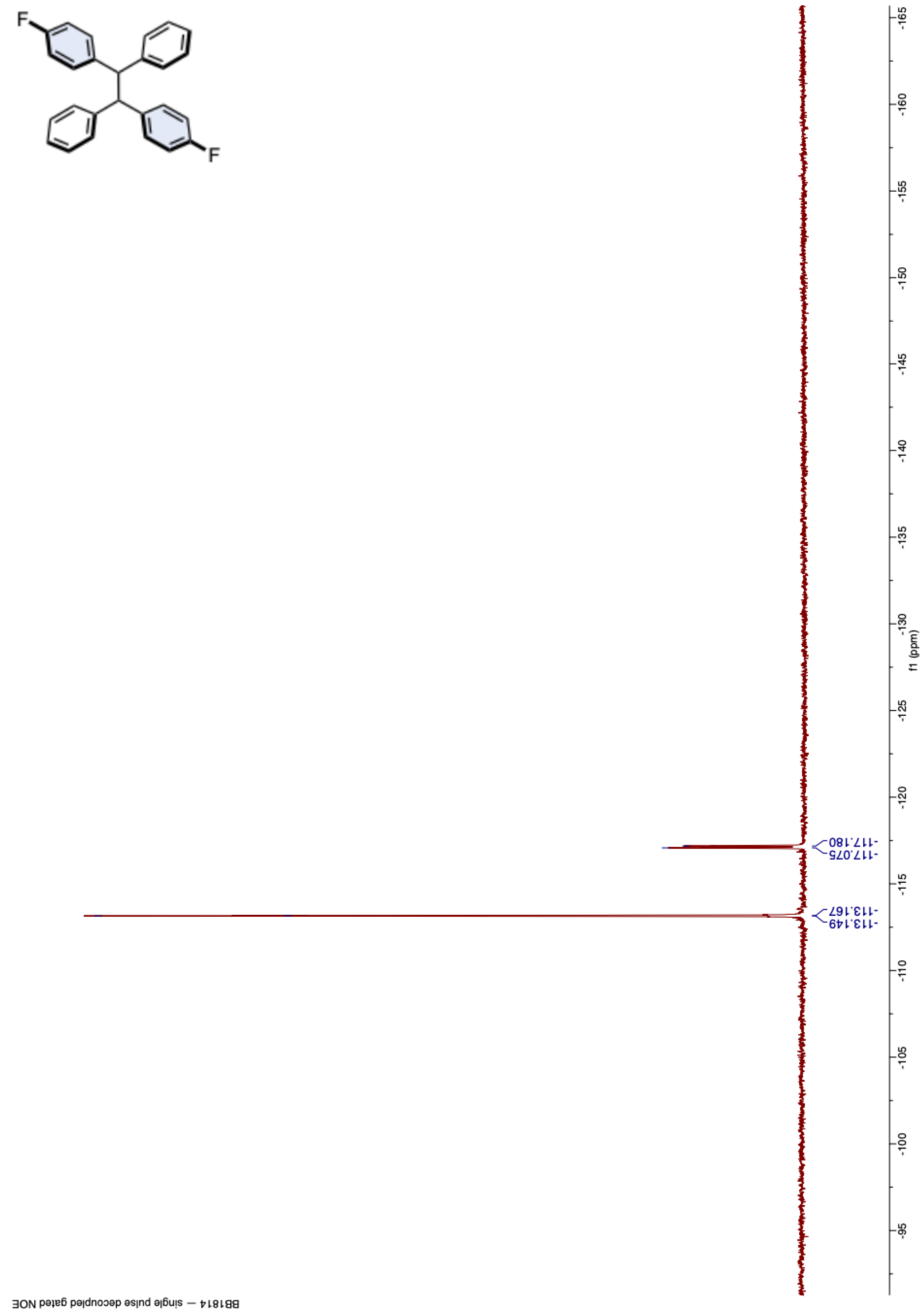

^{1}H NMR of **3J** (400 MHz, $CDCl_3$)

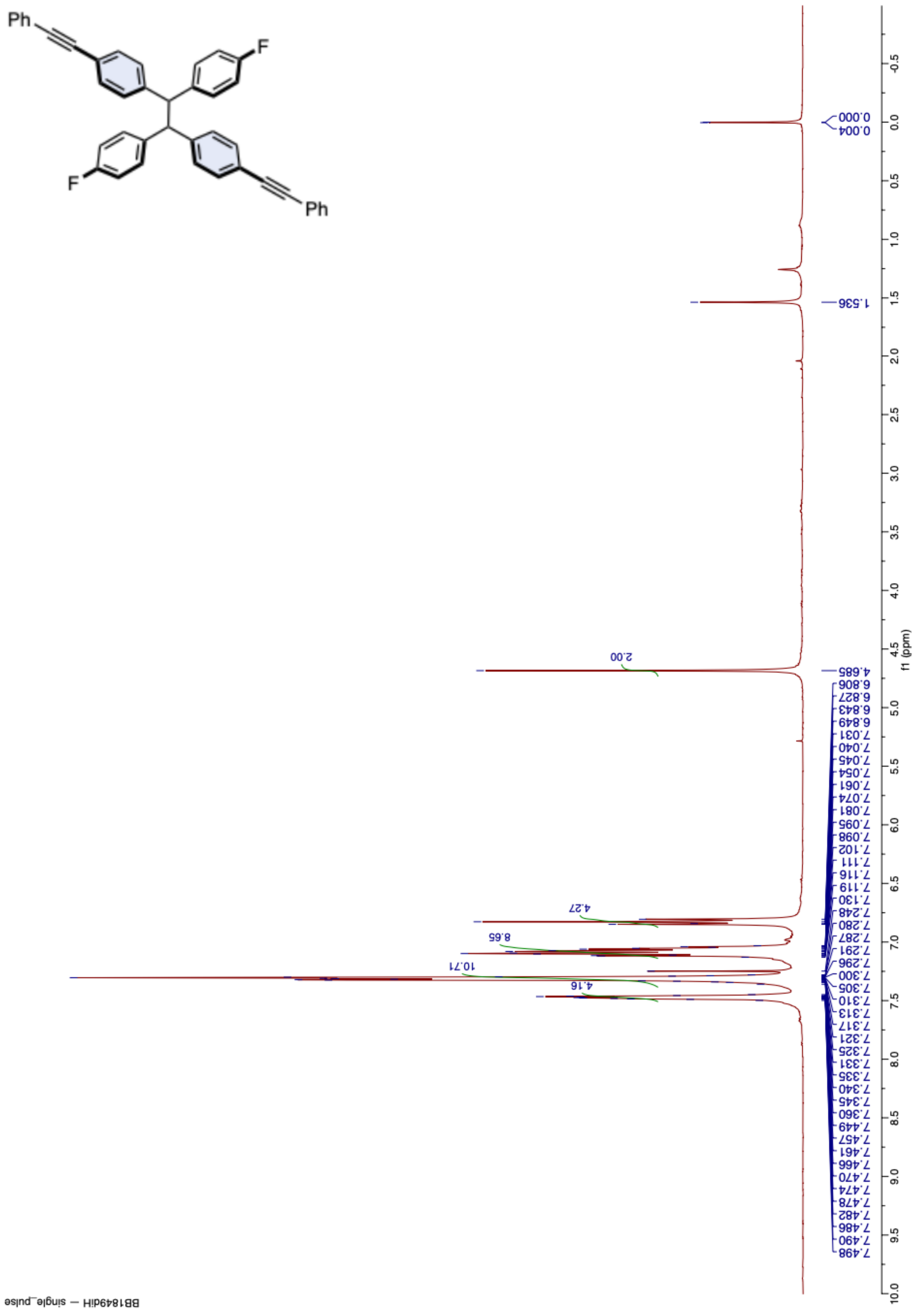

^{13}C NMR of **3J** (101 MHz, $CDCl_3$)

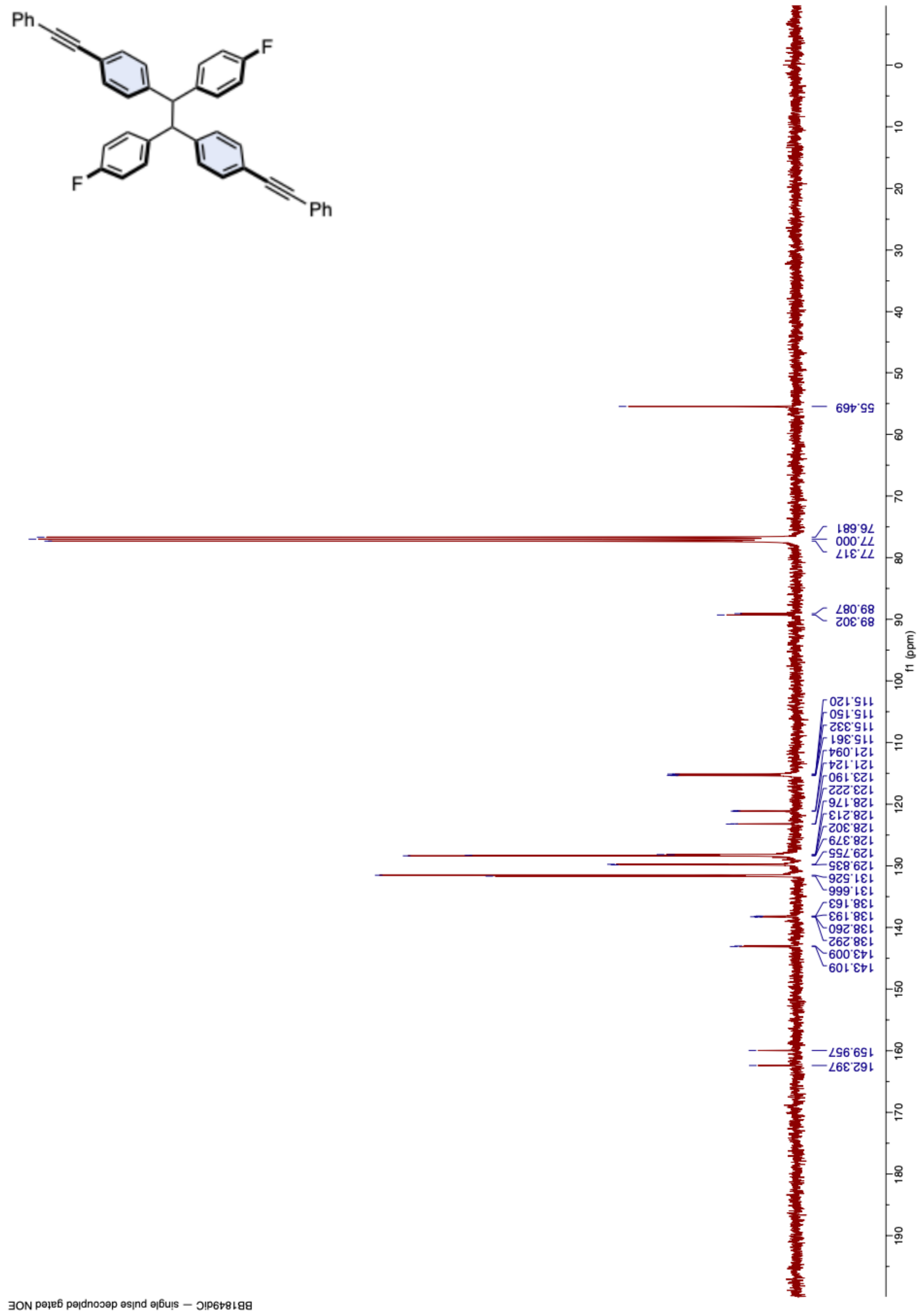

^{19}F NMR of **3J** (376 MHz, $CDCl_3$)

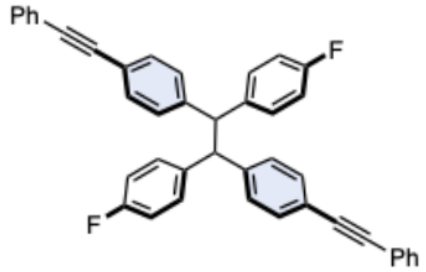

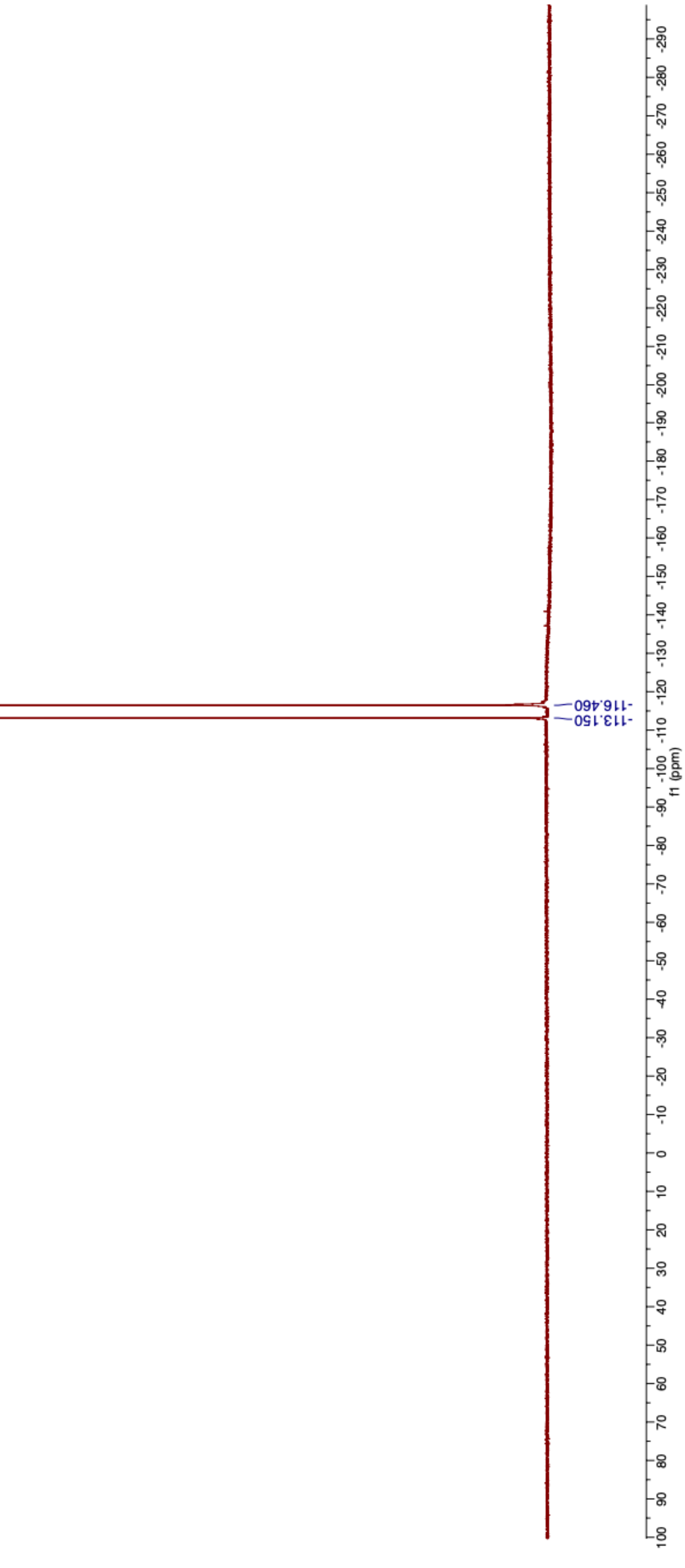

^{1}H NMR of **3K** (400 MHz, $CDCl_3$)

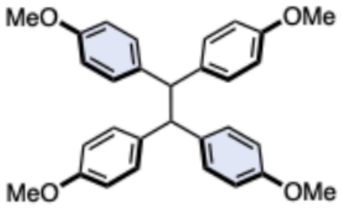

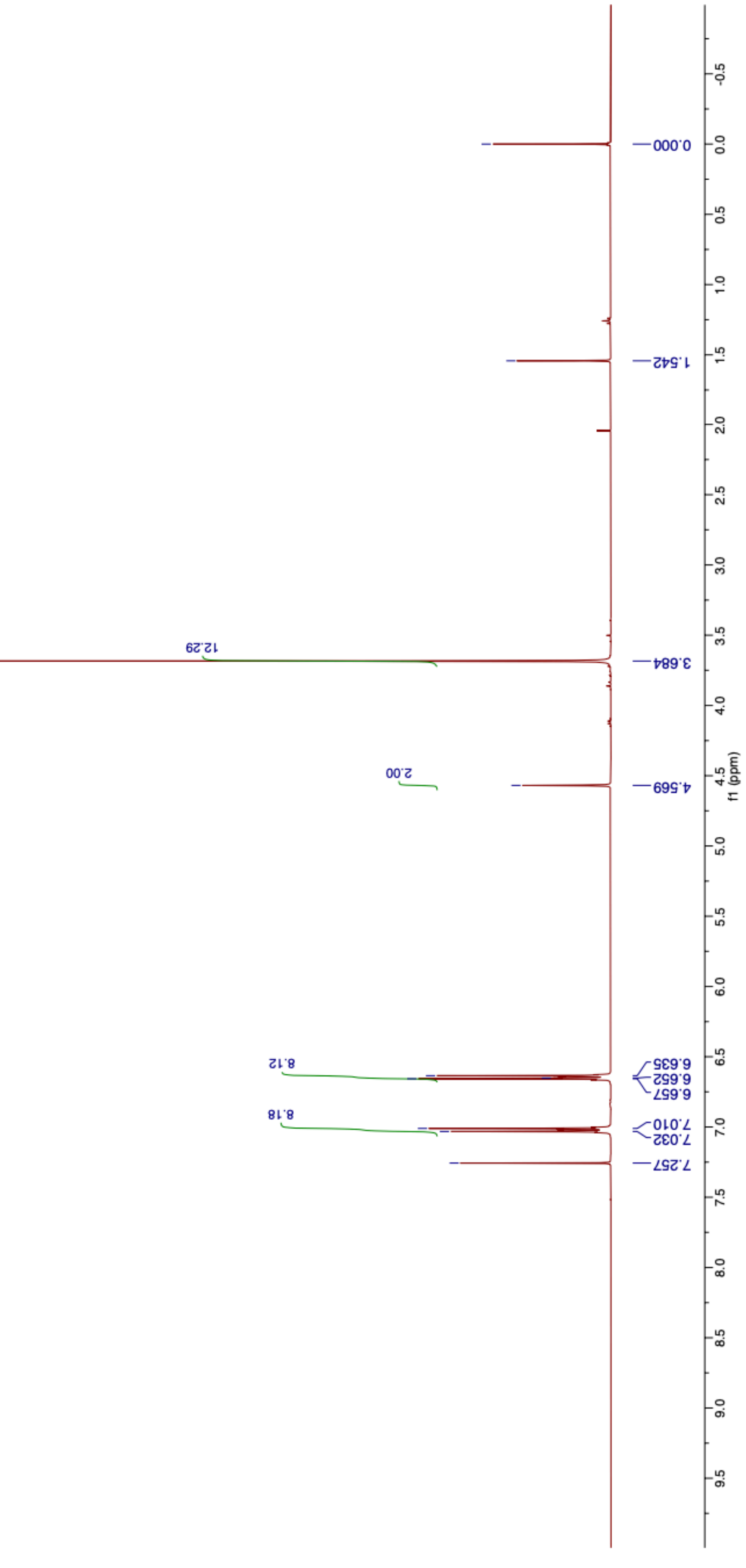

^{13}C NMR of **3K** (101 MHz, $CDCl_3$)

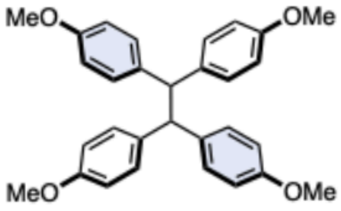

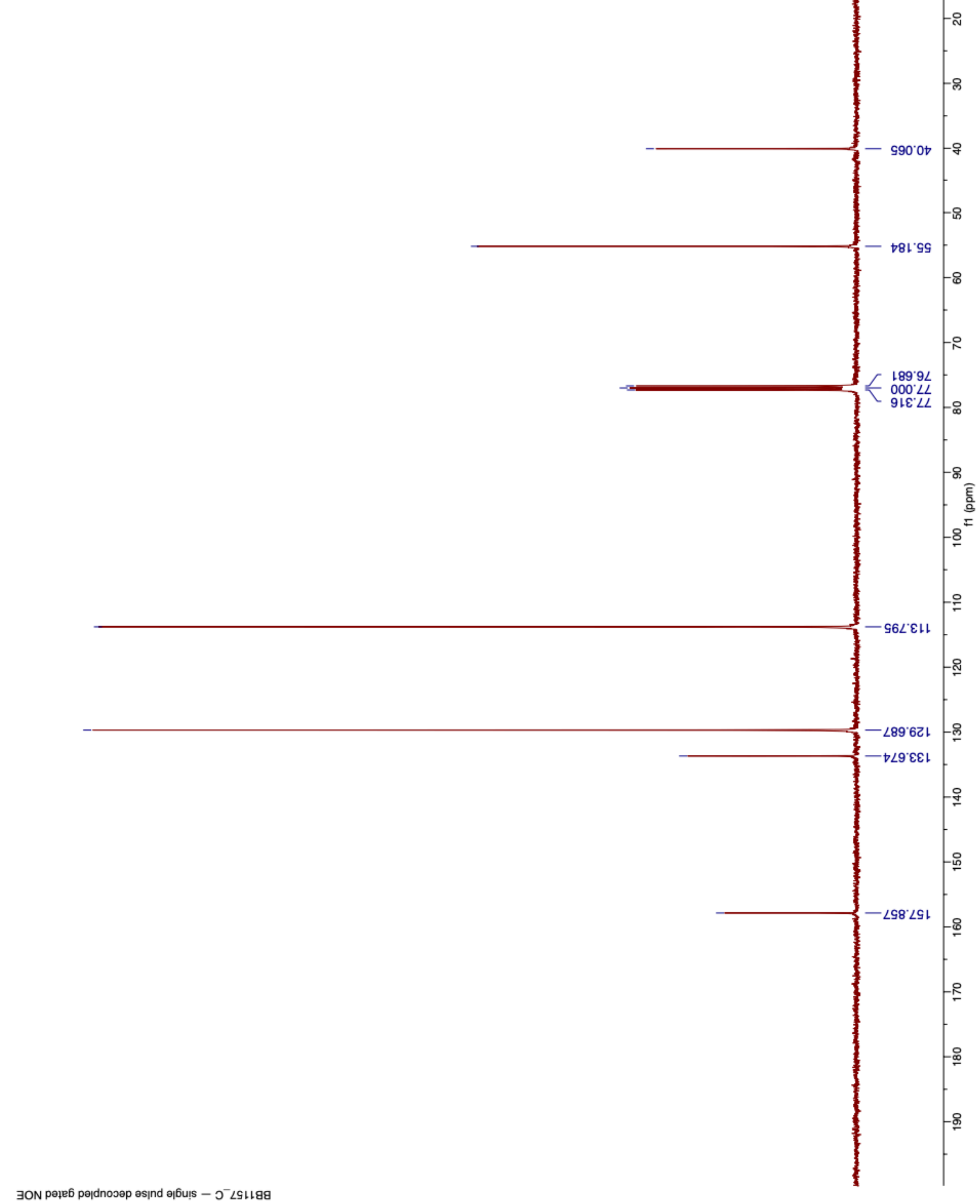

^{1}H NMR of **3L** (400 MHz, $CDCl_3$)

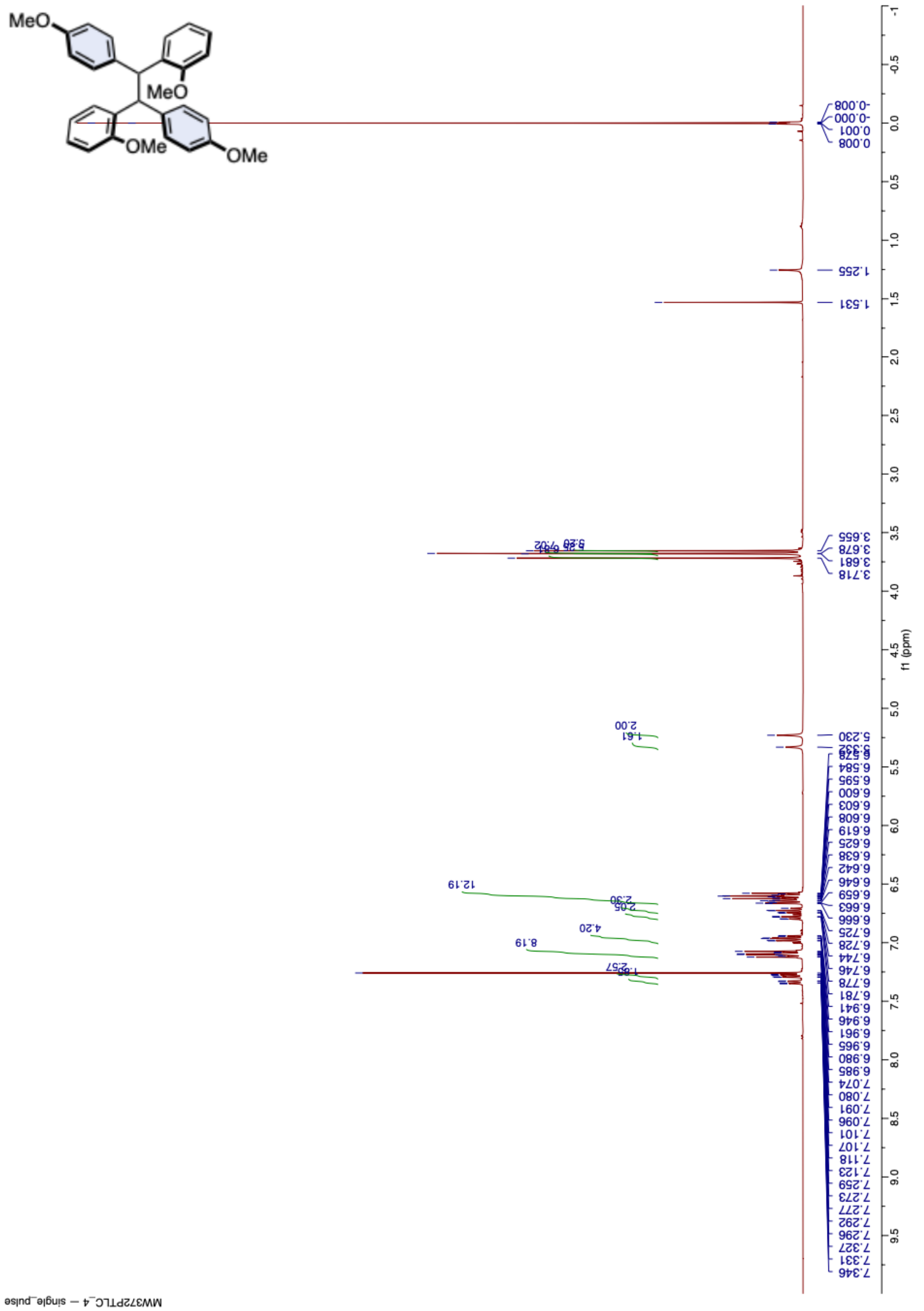

^{13}C NMR of **3L** (101 MHz, $CDCl_3$)

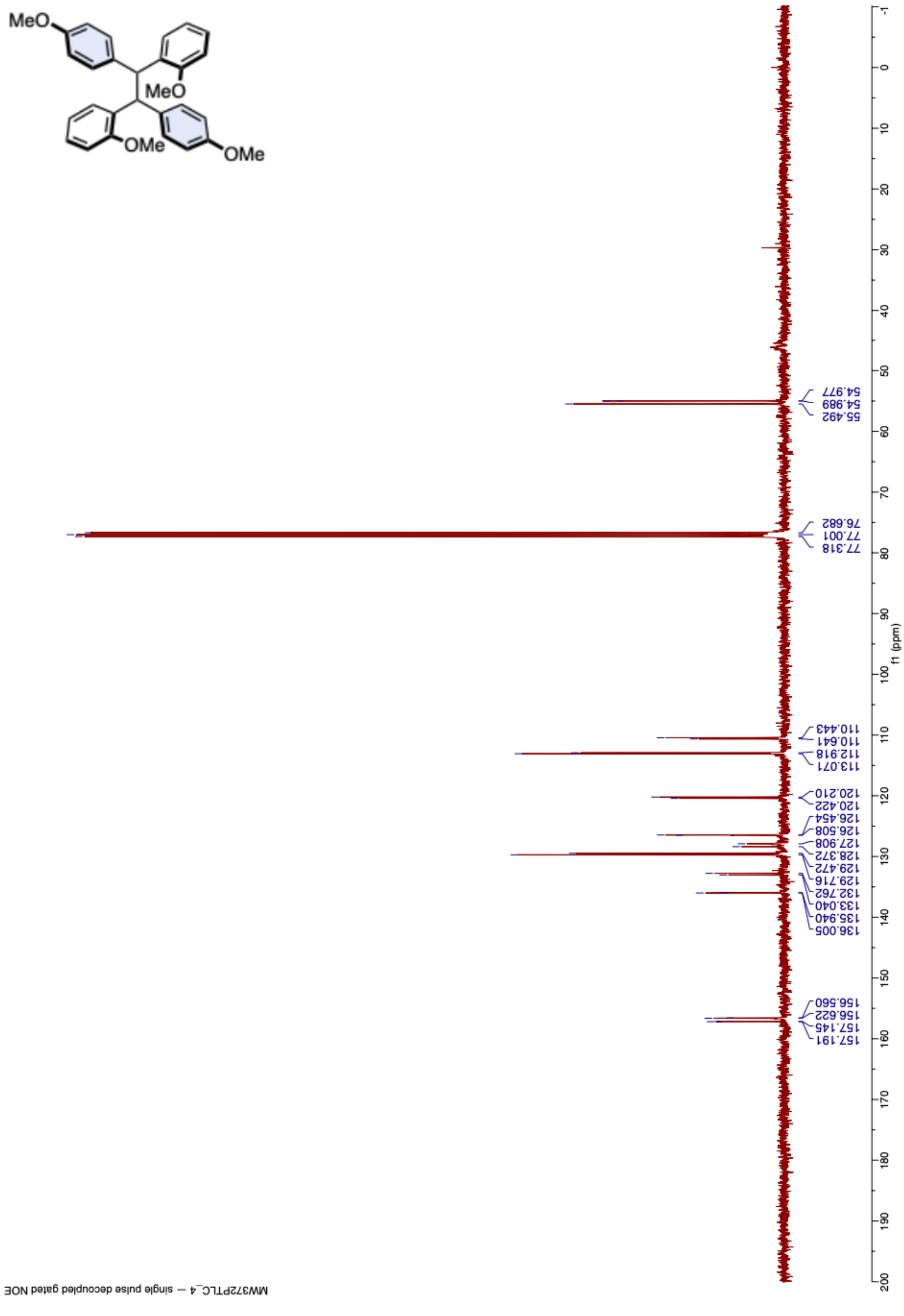

^{1}H NMR of **3M** (400 MHz, $CDCl_3$)

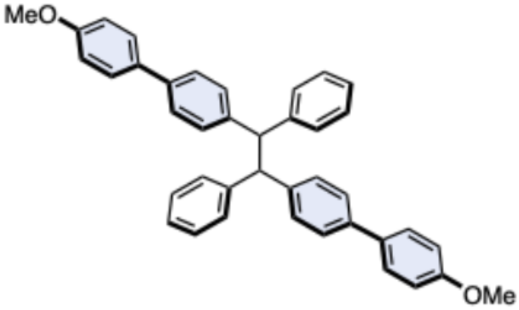

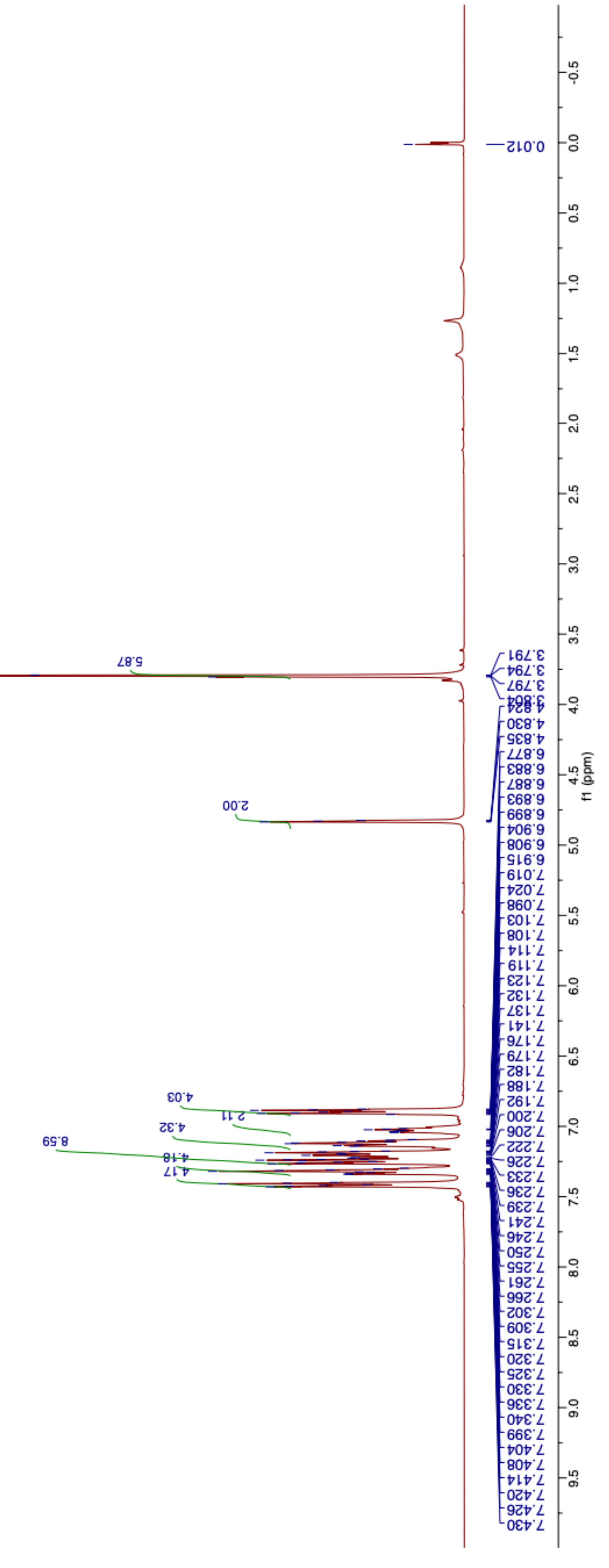

^{13}C NMR of **3M** (101 MHz, $CDCl_3$)

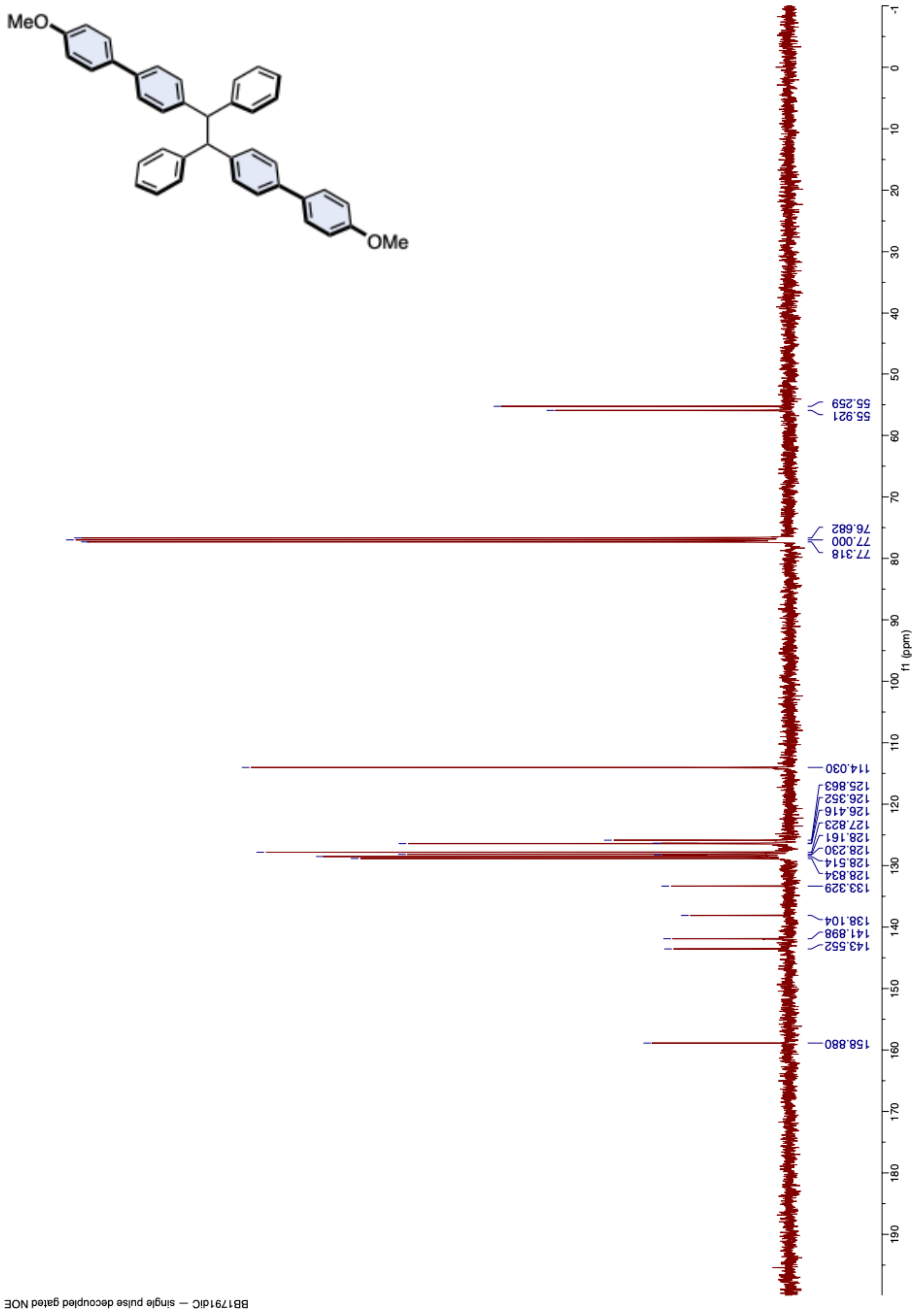

^{1}H NMR of **3N** (400 MHz, $CDCl_3$)

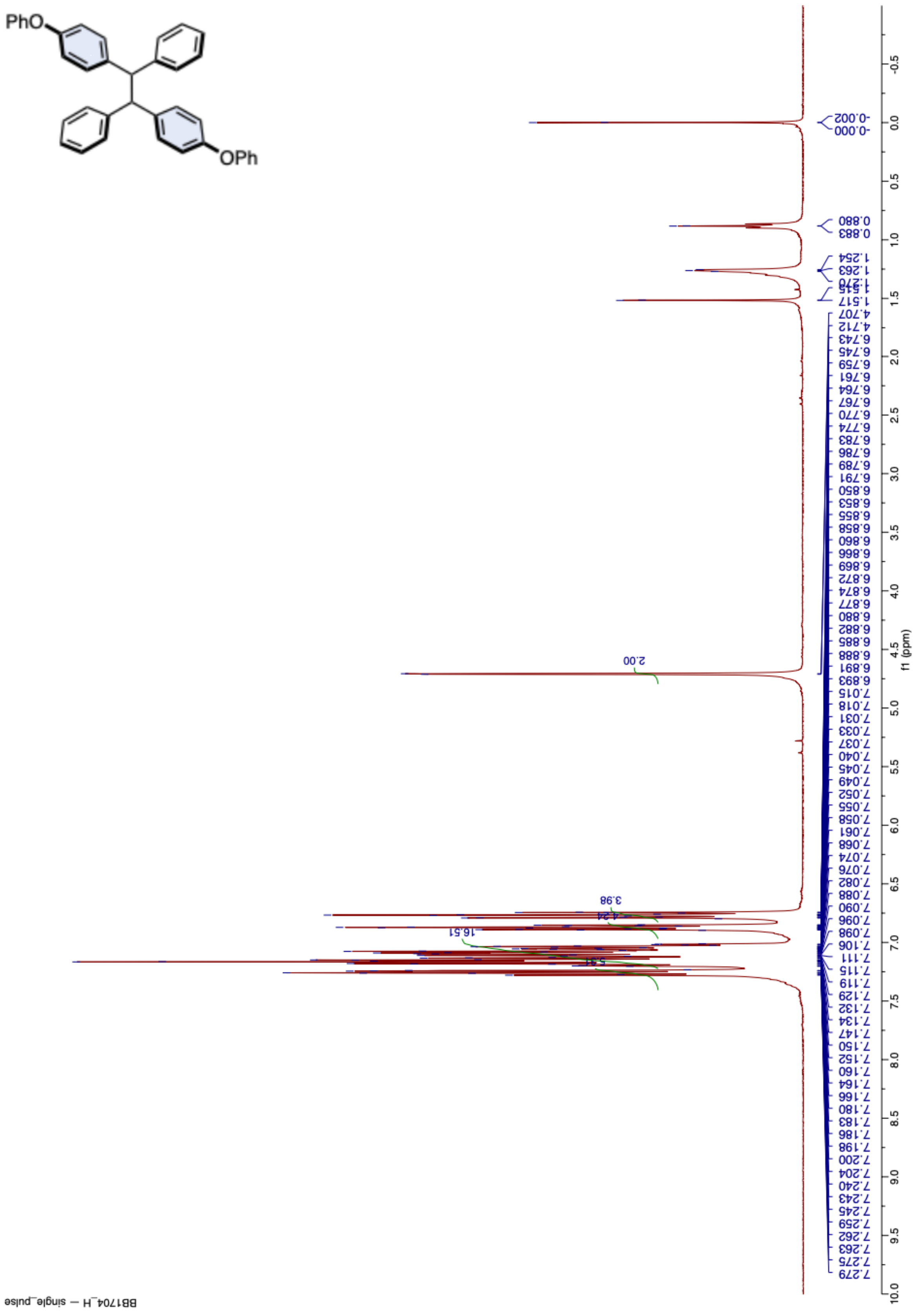

^{13}C NMR of **3N** (101 MHz, $CDCl_3$)

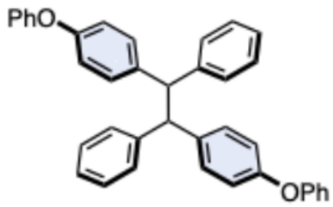

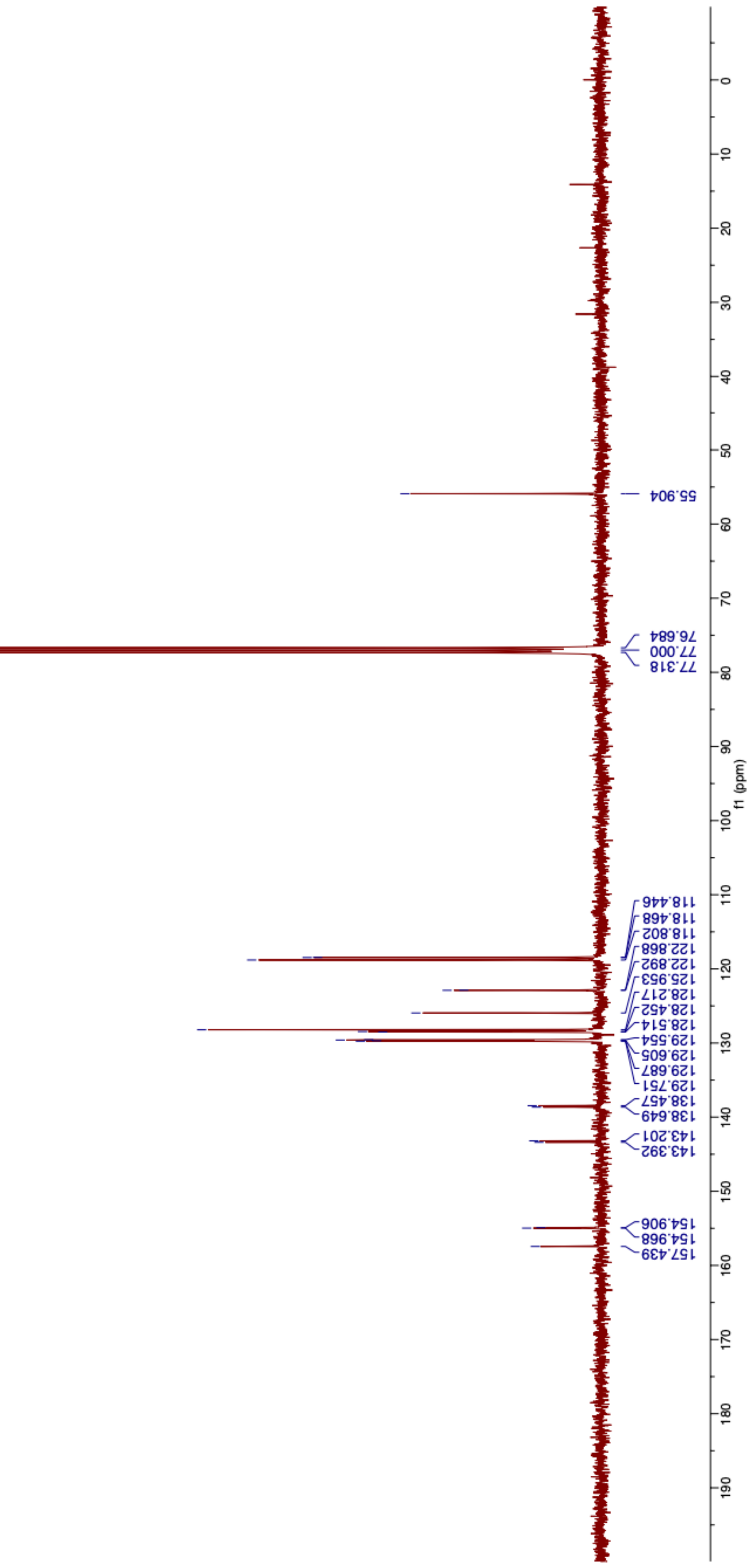

^{1}H NMR of **3O** (400 MHz, $CDCl_3$)

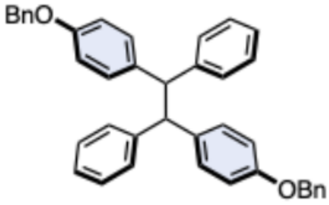

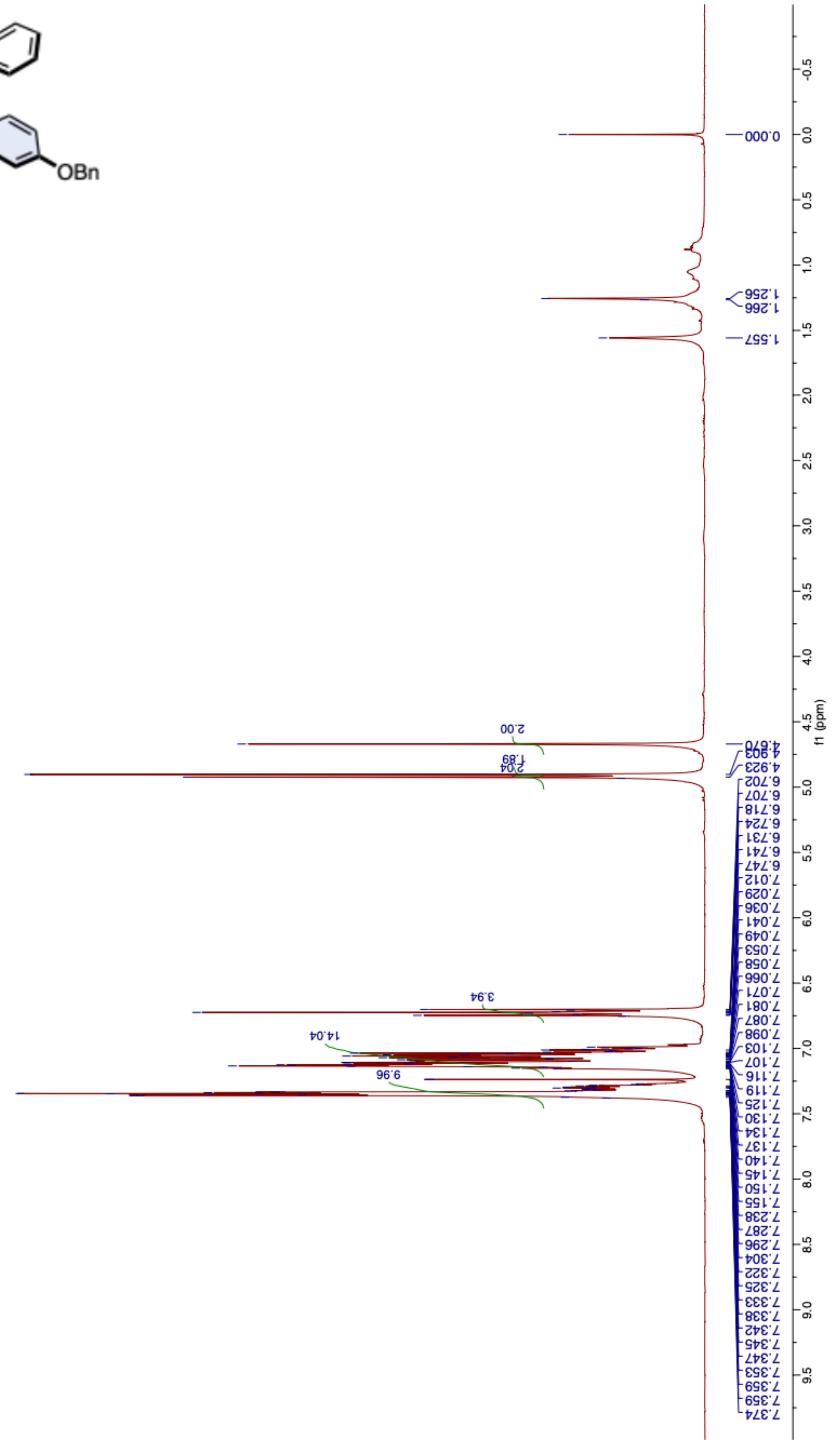

^{13}C NMR of **3O** (101 MHz, $CDCl_3$)

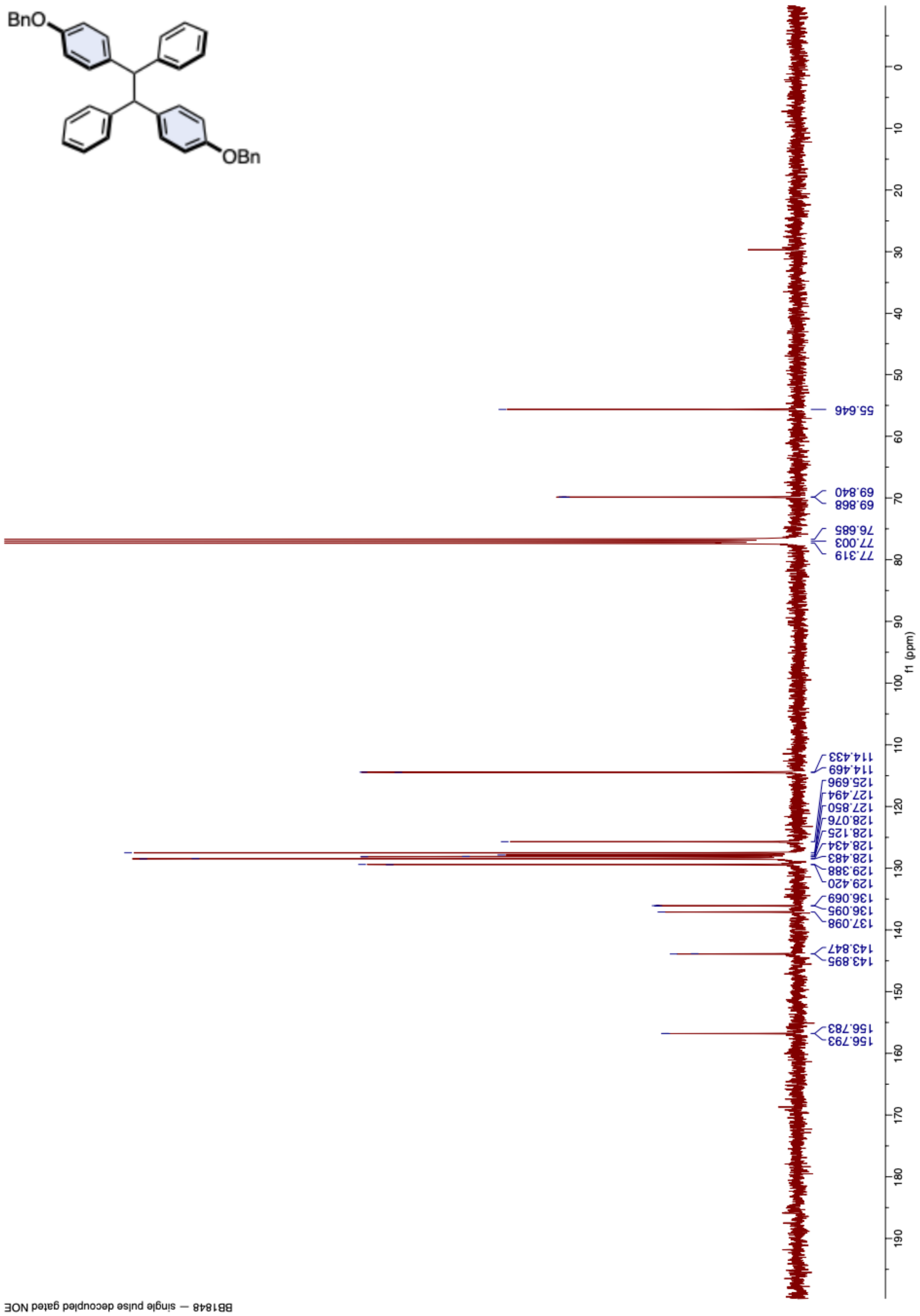

^{1}H NMR of **3P** (400 MHz, $CDCl_3$)

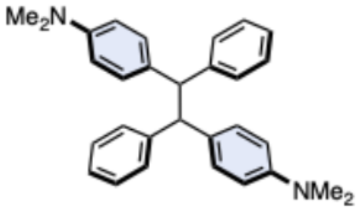

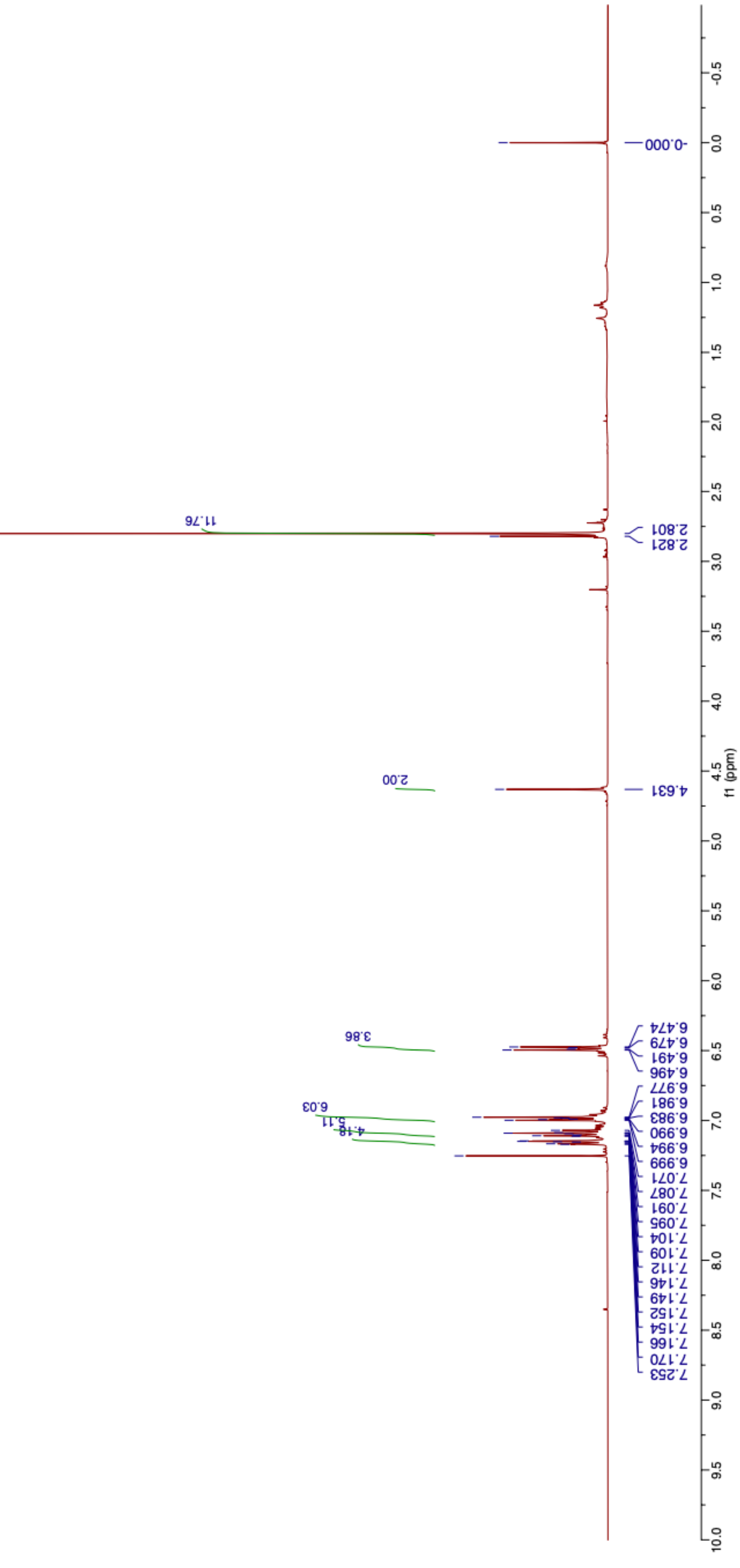

^{13}C NMR of **3P** (101 MHz, $CDCl_3$)

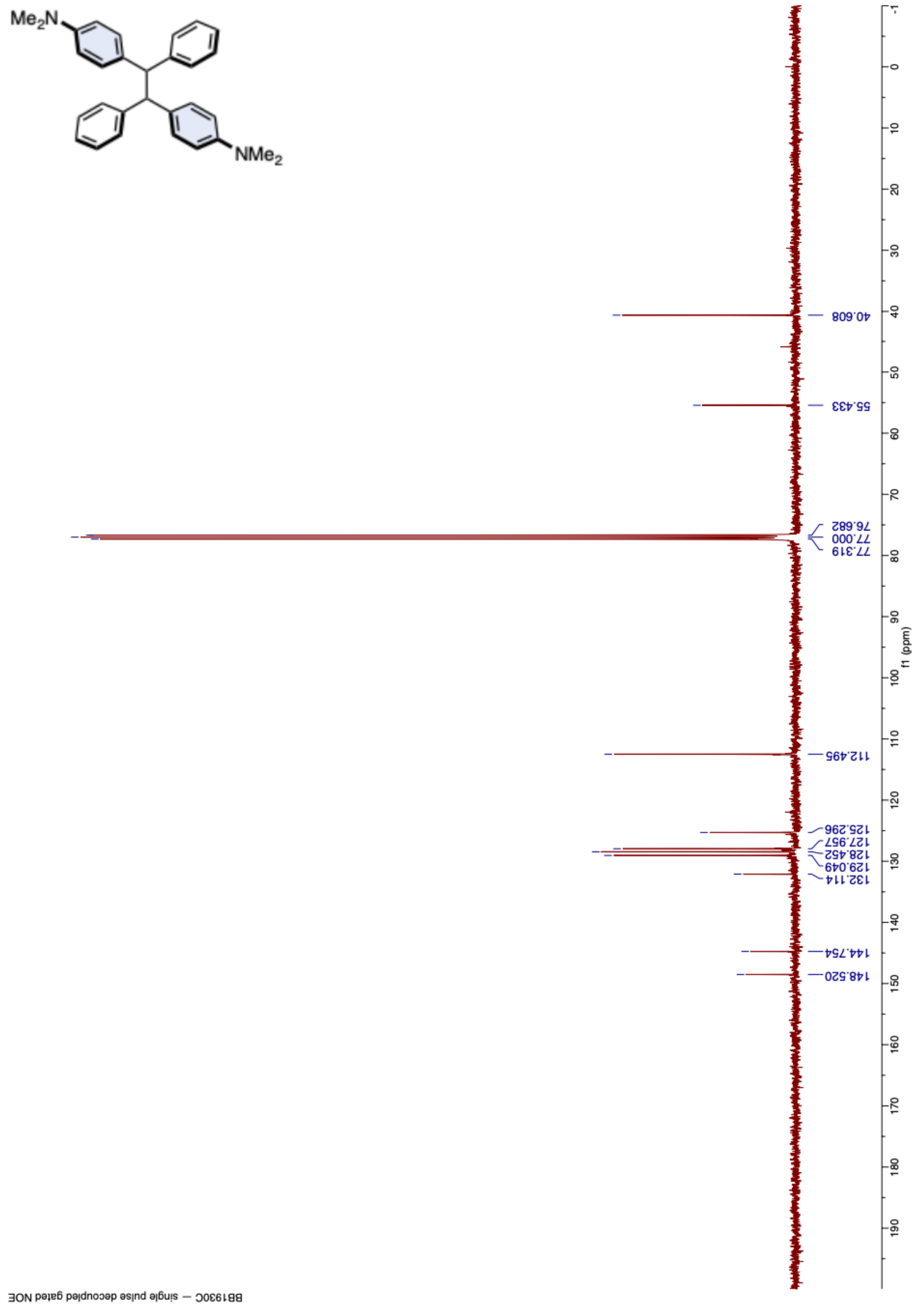

^{1}H NMR of **3Q** (400 MHz, DMSO-d_6)

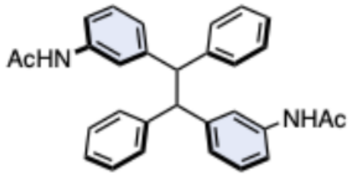

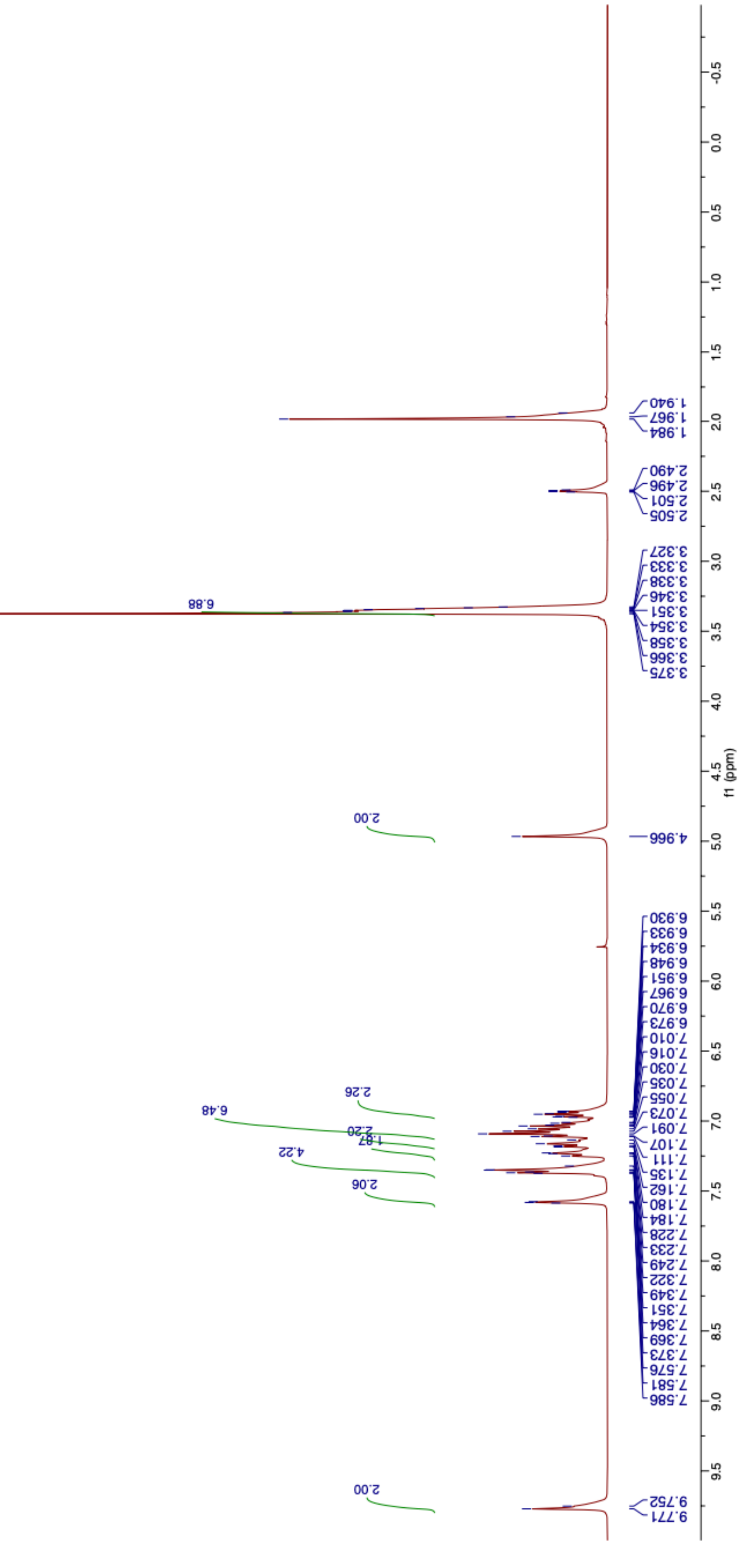

^{13}C NMR of **3Q** (101 MHz, DMSO-d_6)

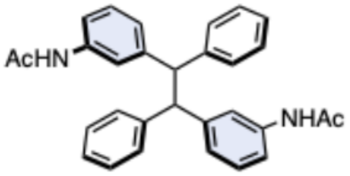

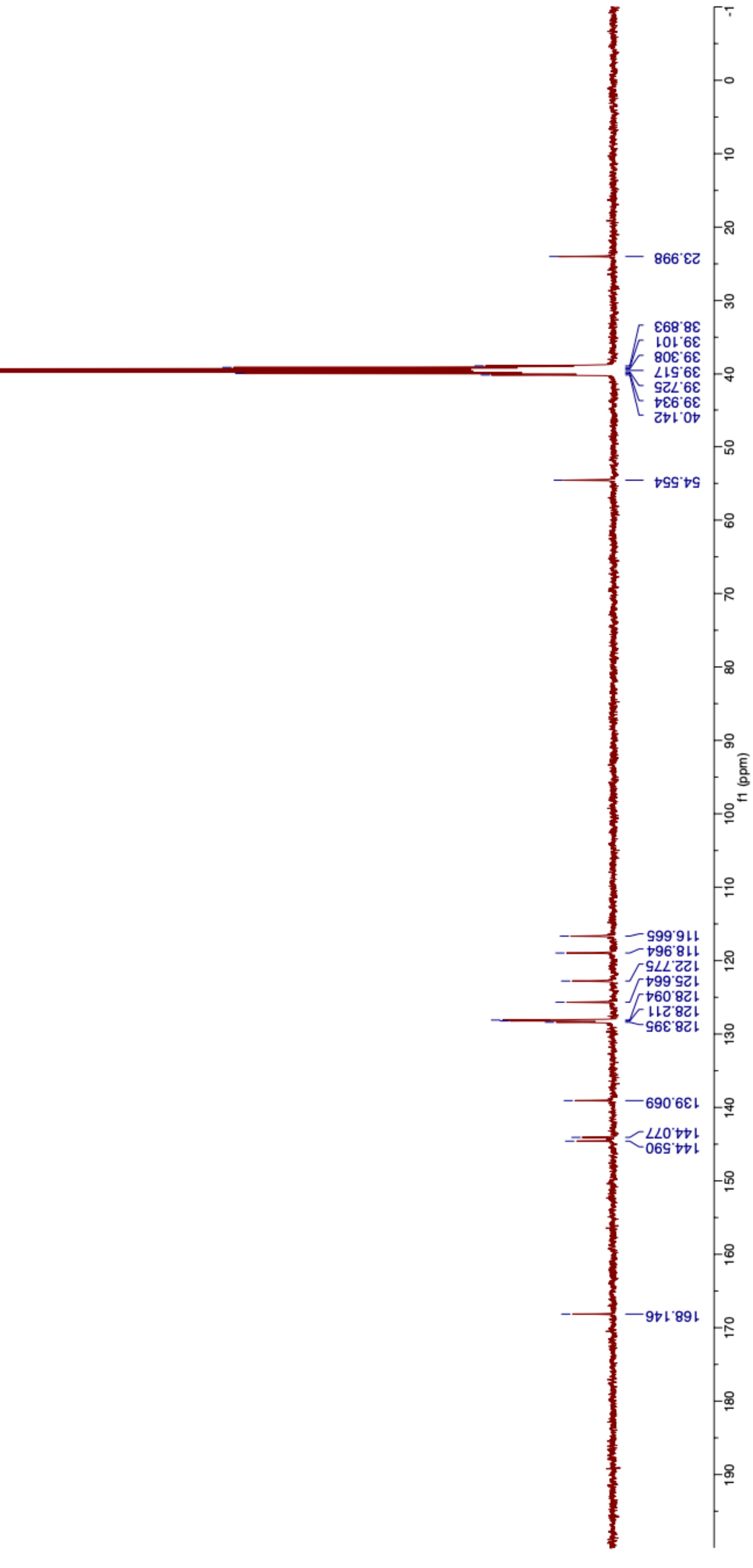

^{1}H NMR of **3R** (400 MHz, $CDCl_3$)

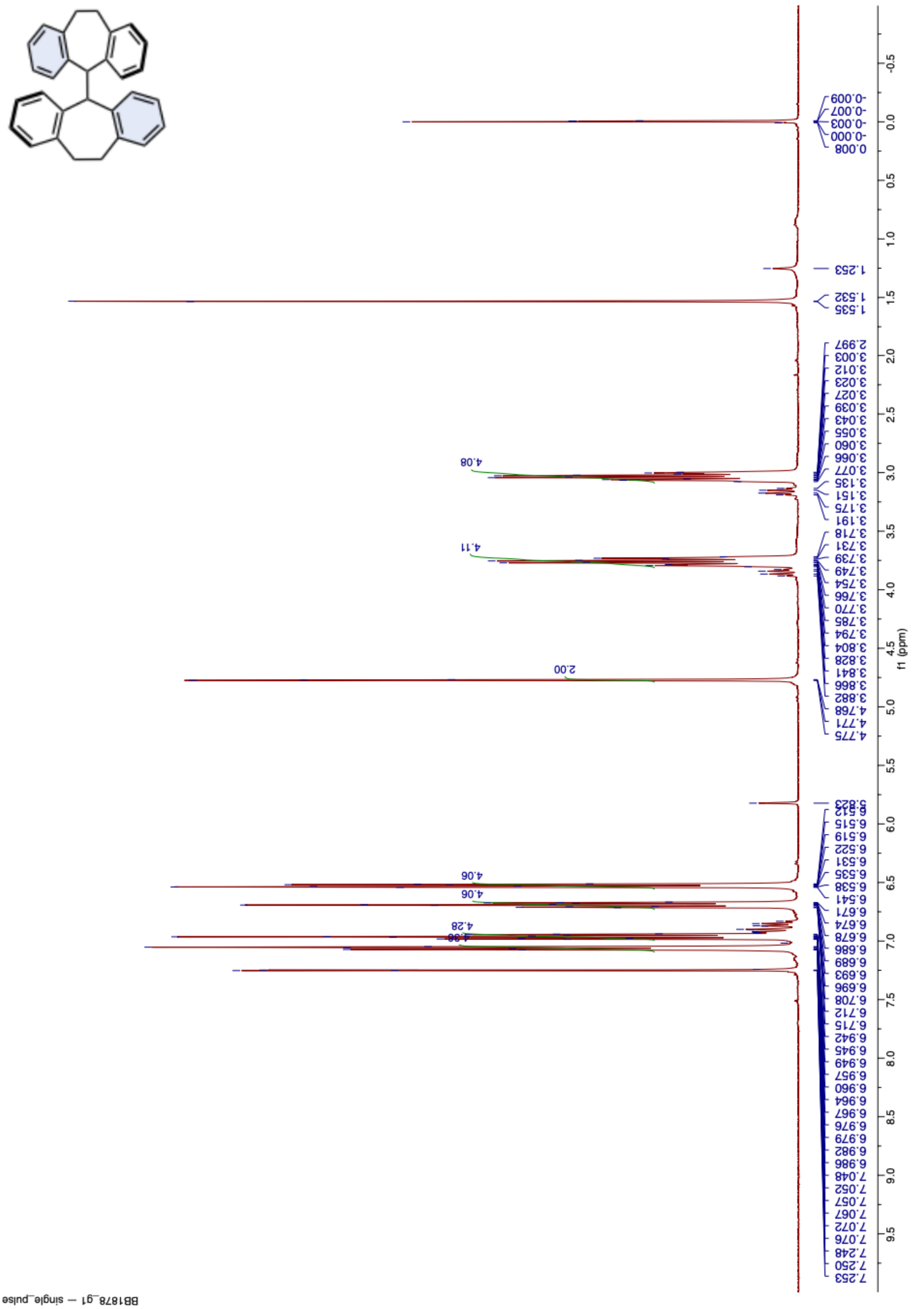

^{13}C NMR of **3R** (101 MHz, $CDCl_3$)

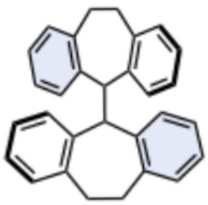

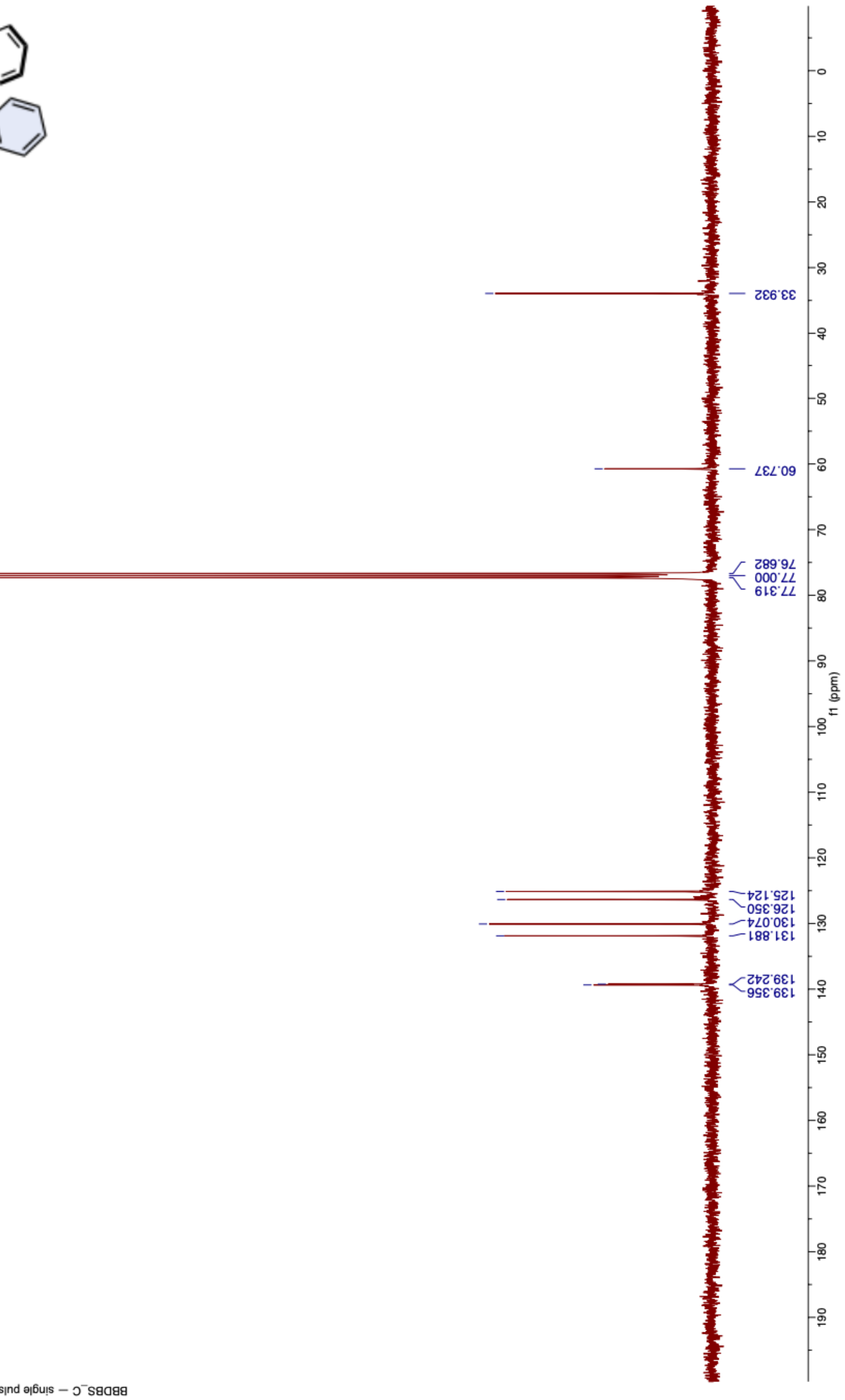

^{1}H NMR of **3S** (400 MHz, $CDCl_3$)

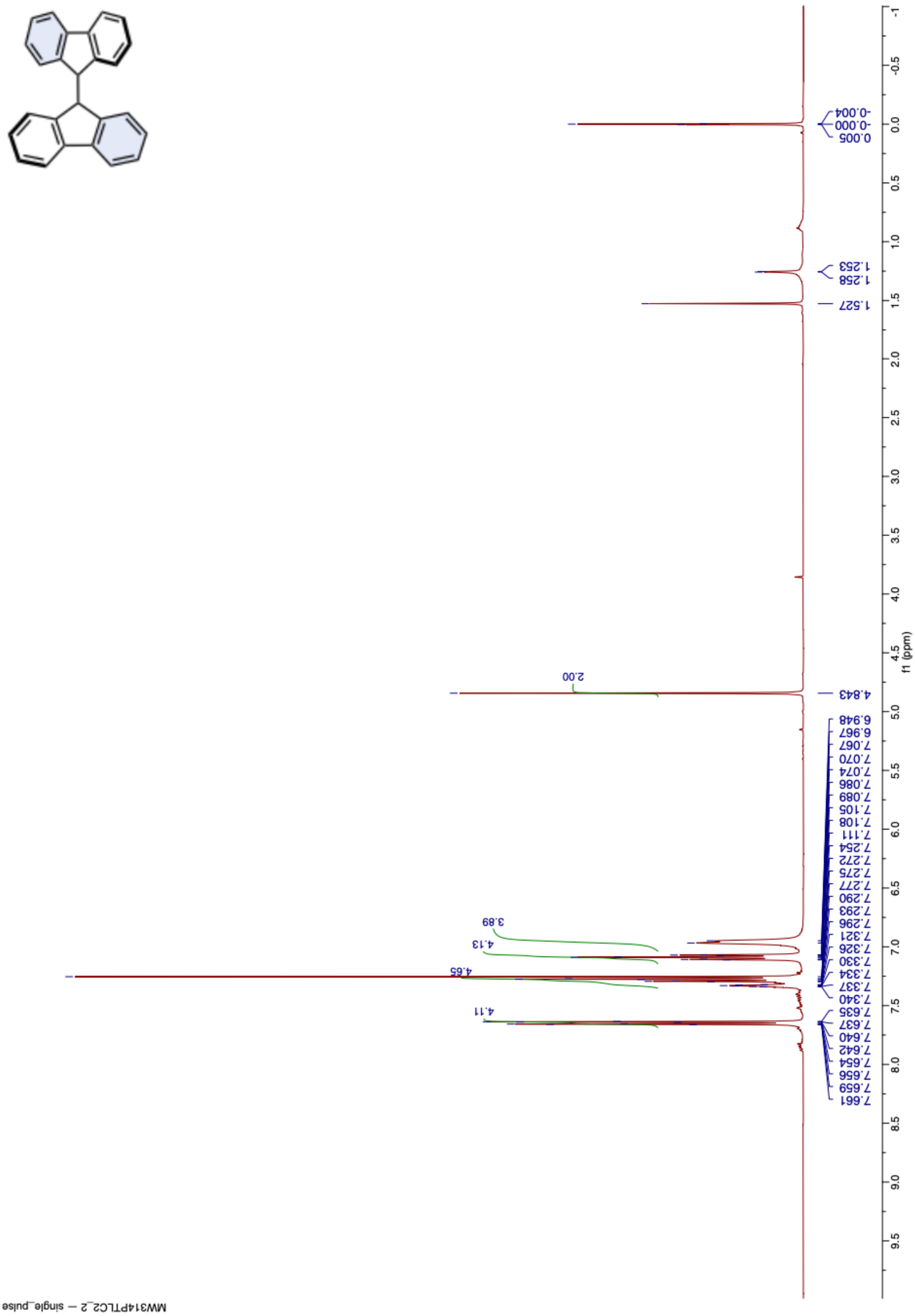

^{13}C NMR of **3S** (101 MHz, $CDCl_3$)

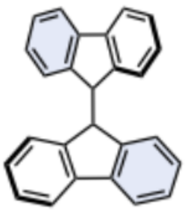

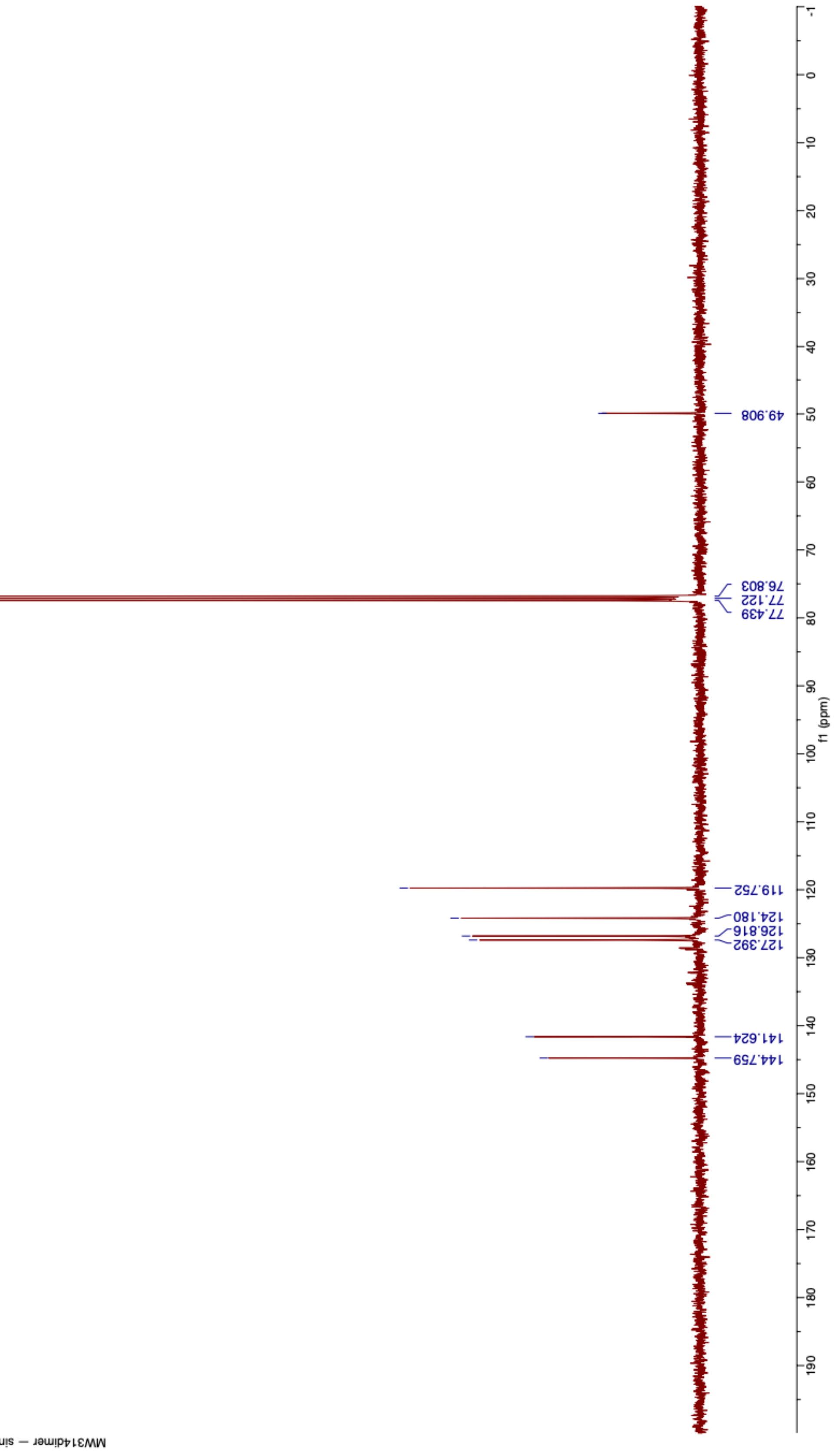

^{1}H NMR of **3T** (400 MHz, $CDCl_3$)

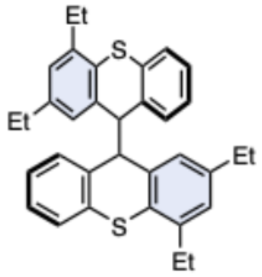

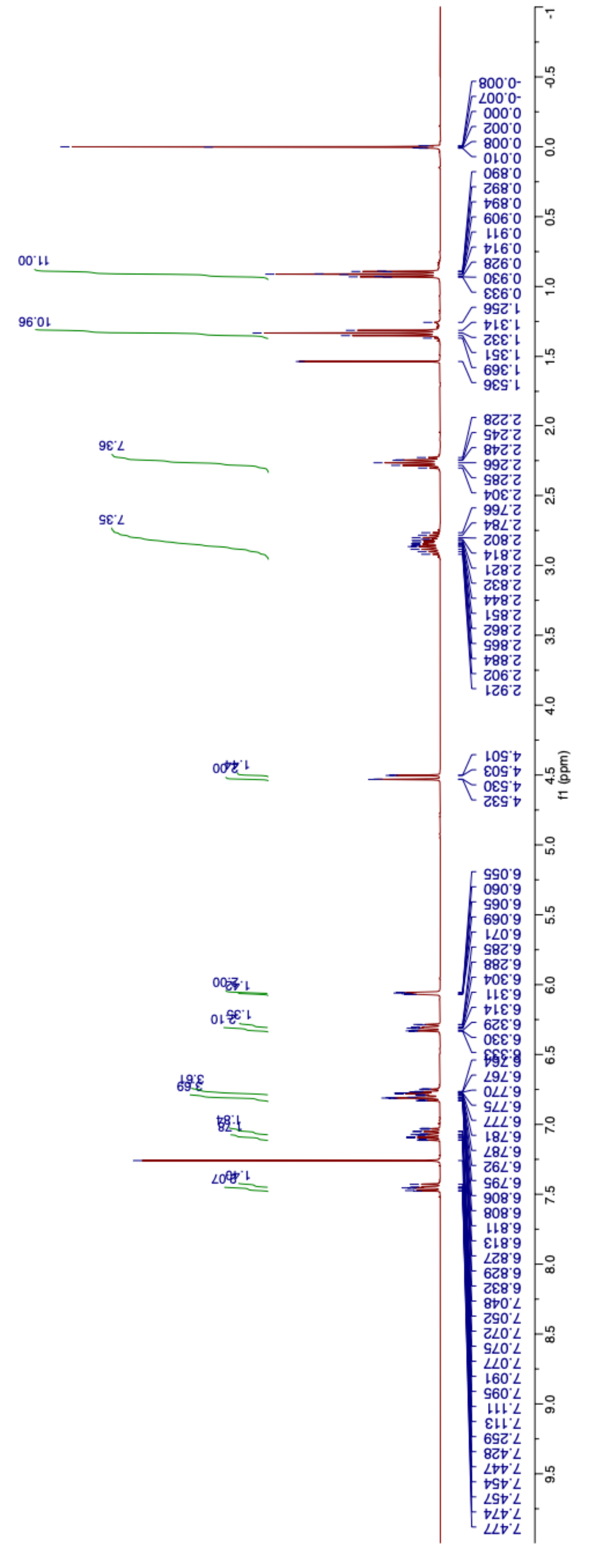

^{13}C NMR of **3T** (101 MHz, $CDCl_3$)

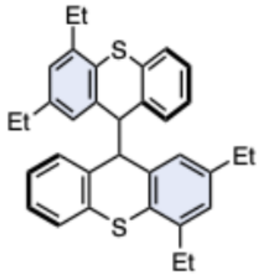

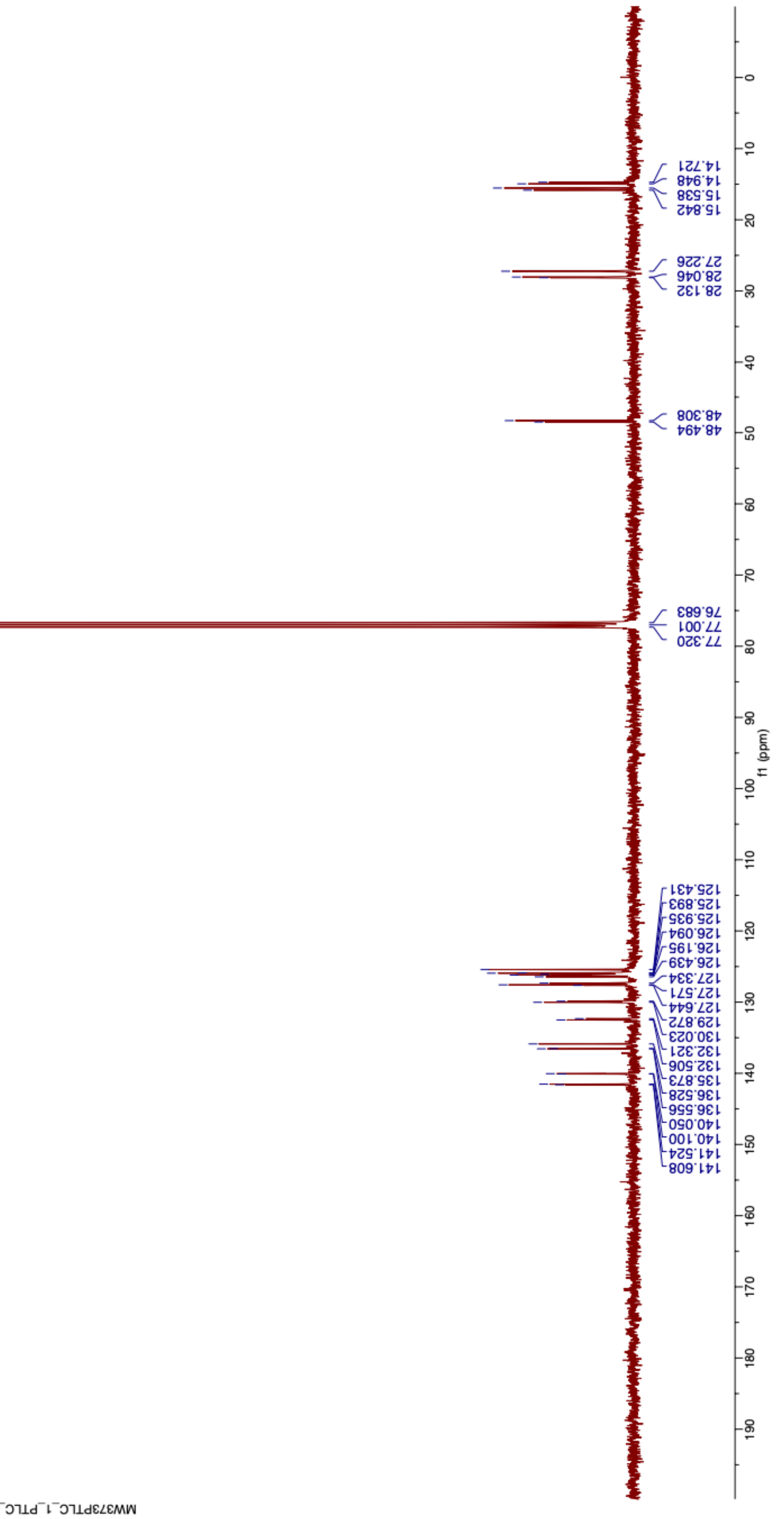

^{1}H NMR of **3U** (400 MHz, $CDCl_3$)

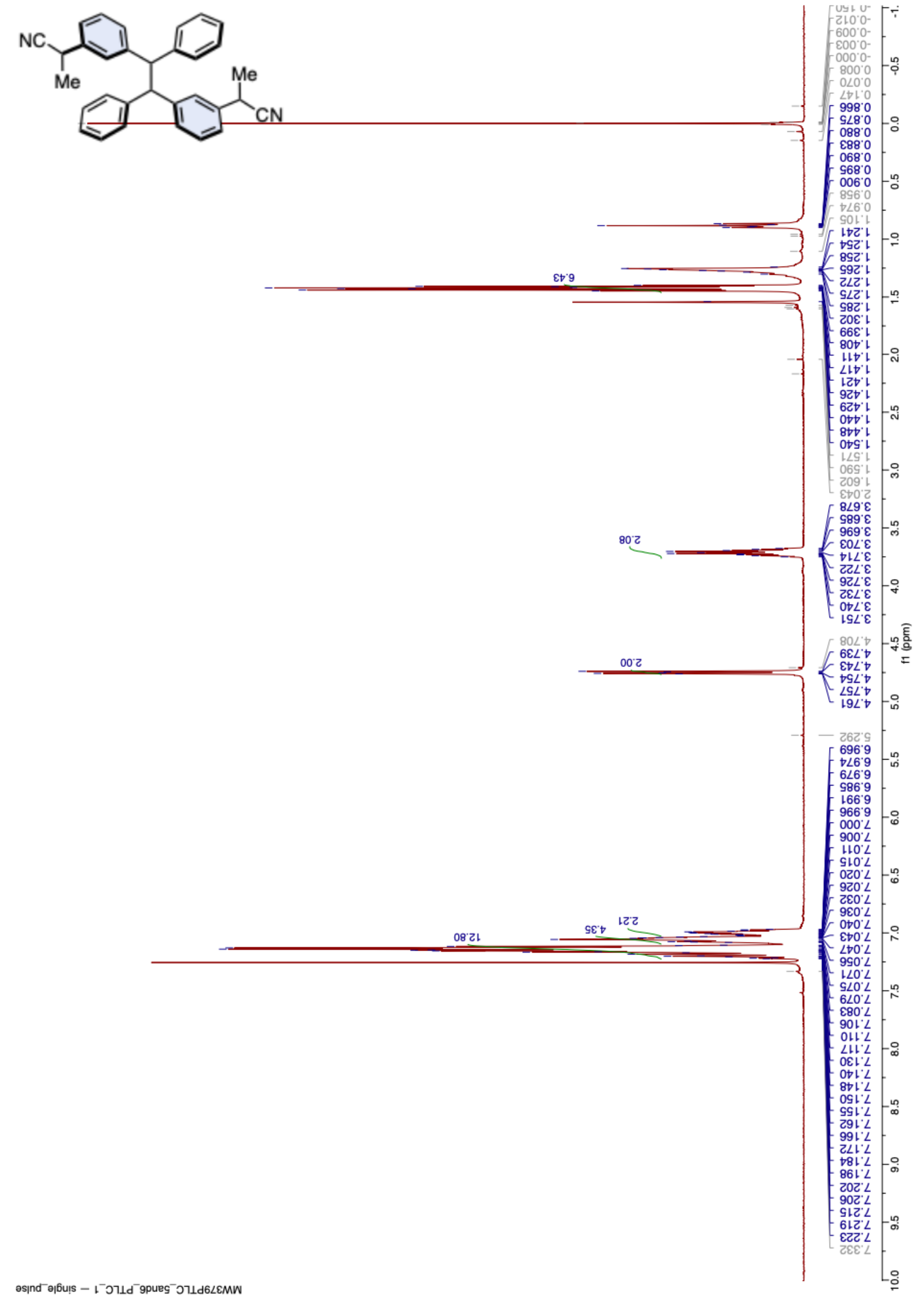

^{13}C NMR of **3U** (101 MHz, $CDCl_3$)

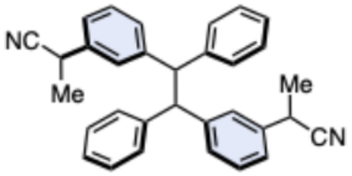

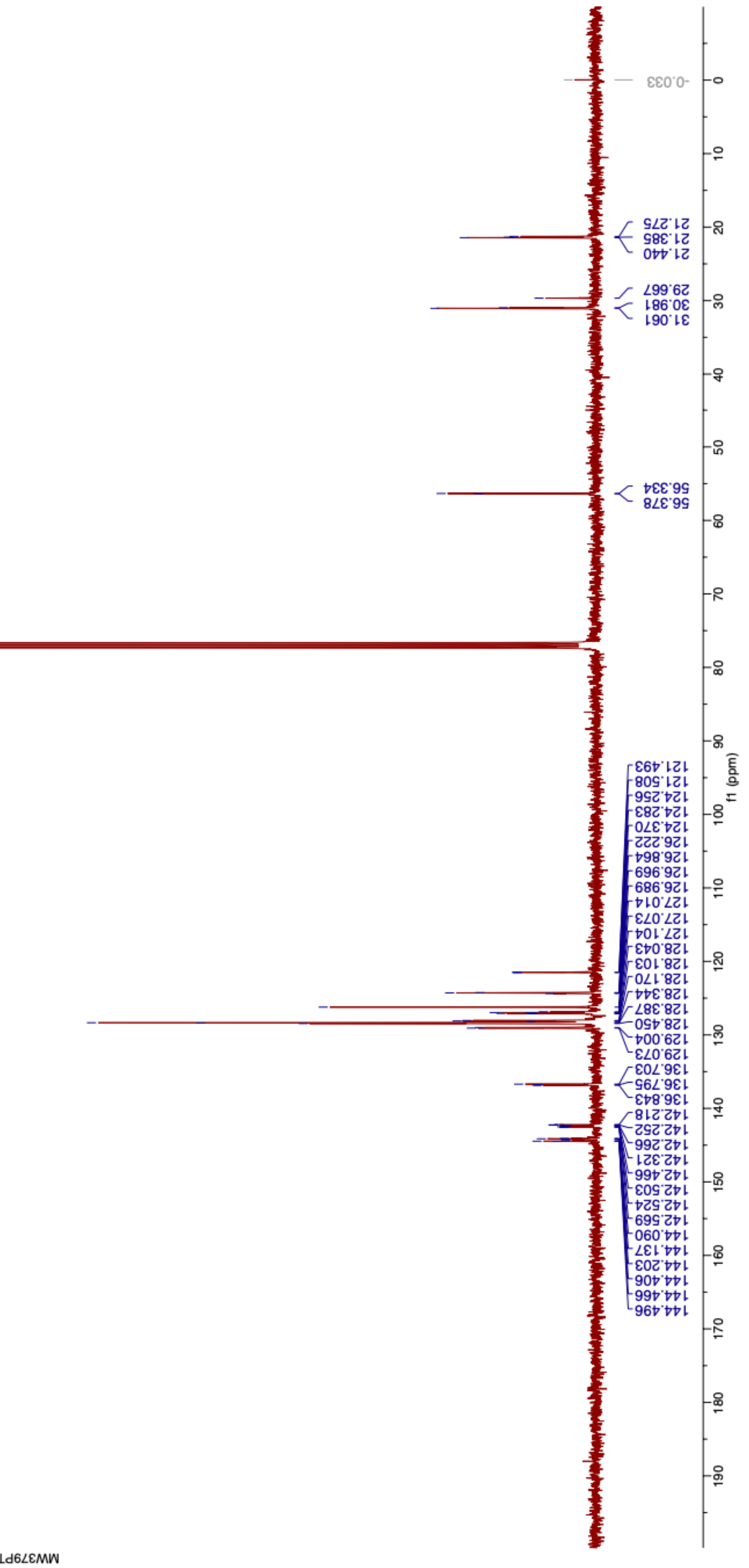

^{1}H NMR of **3V** (400 MHz, $CDCl_3$)

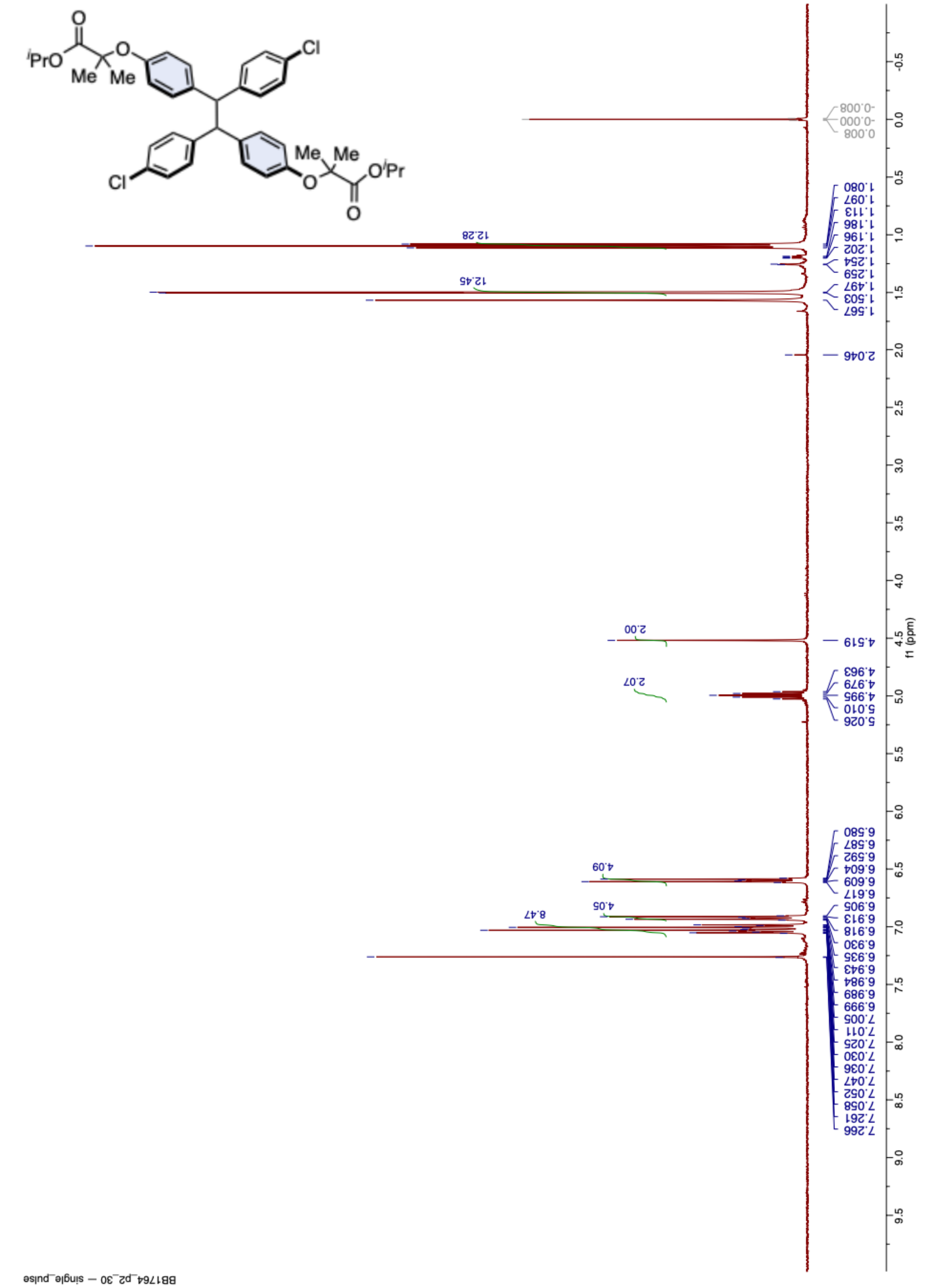

^{13}C NMR of **3V** (101 MHz, $CDCl_3$)

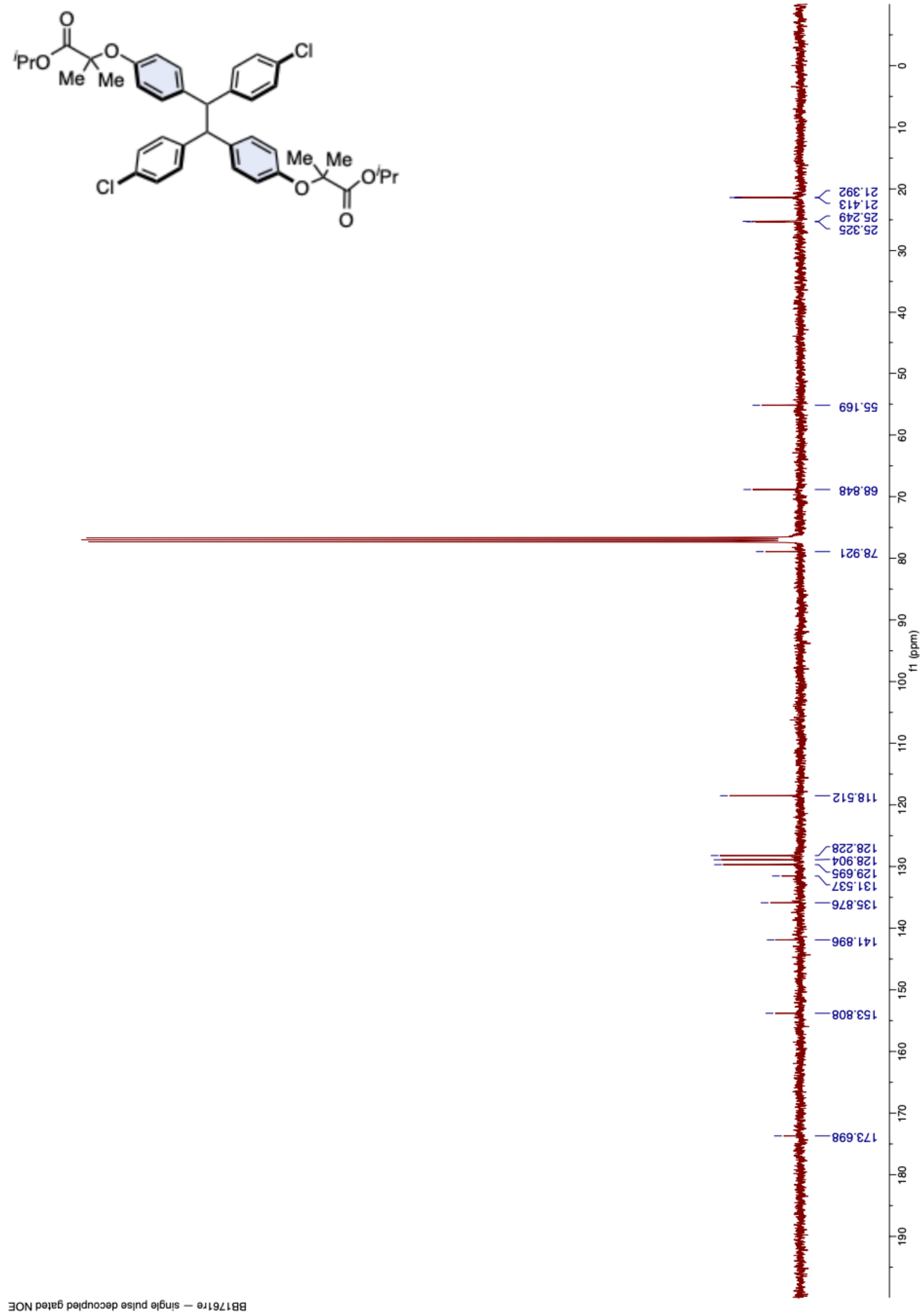

^{1}H NMR of **6A** (400 MHz, $CDCl_3$)

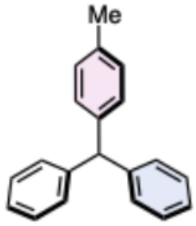

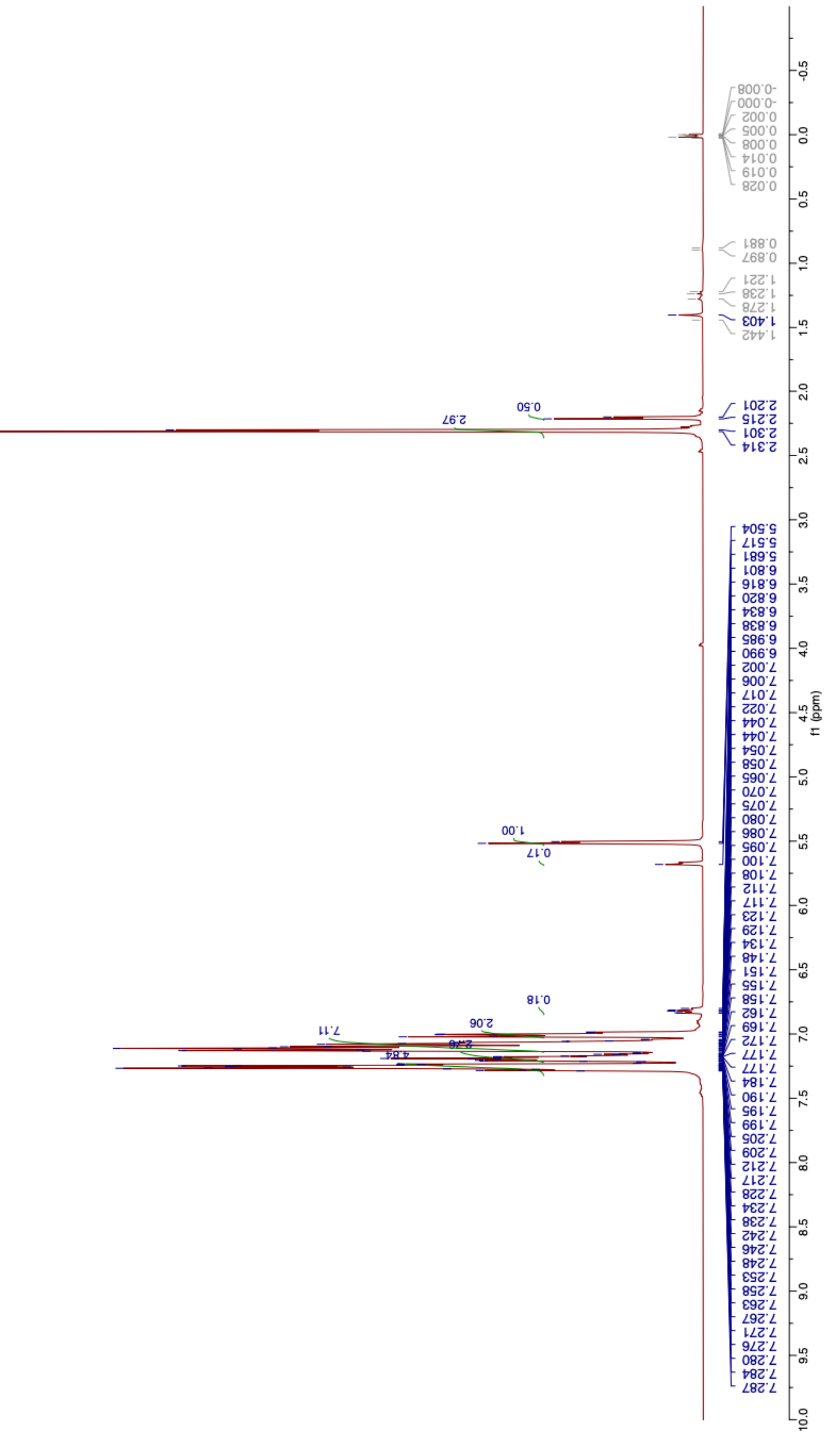

^{13}C NMR of **6A** (101 MHz, $CDCl_3$)

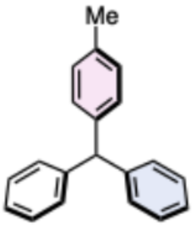

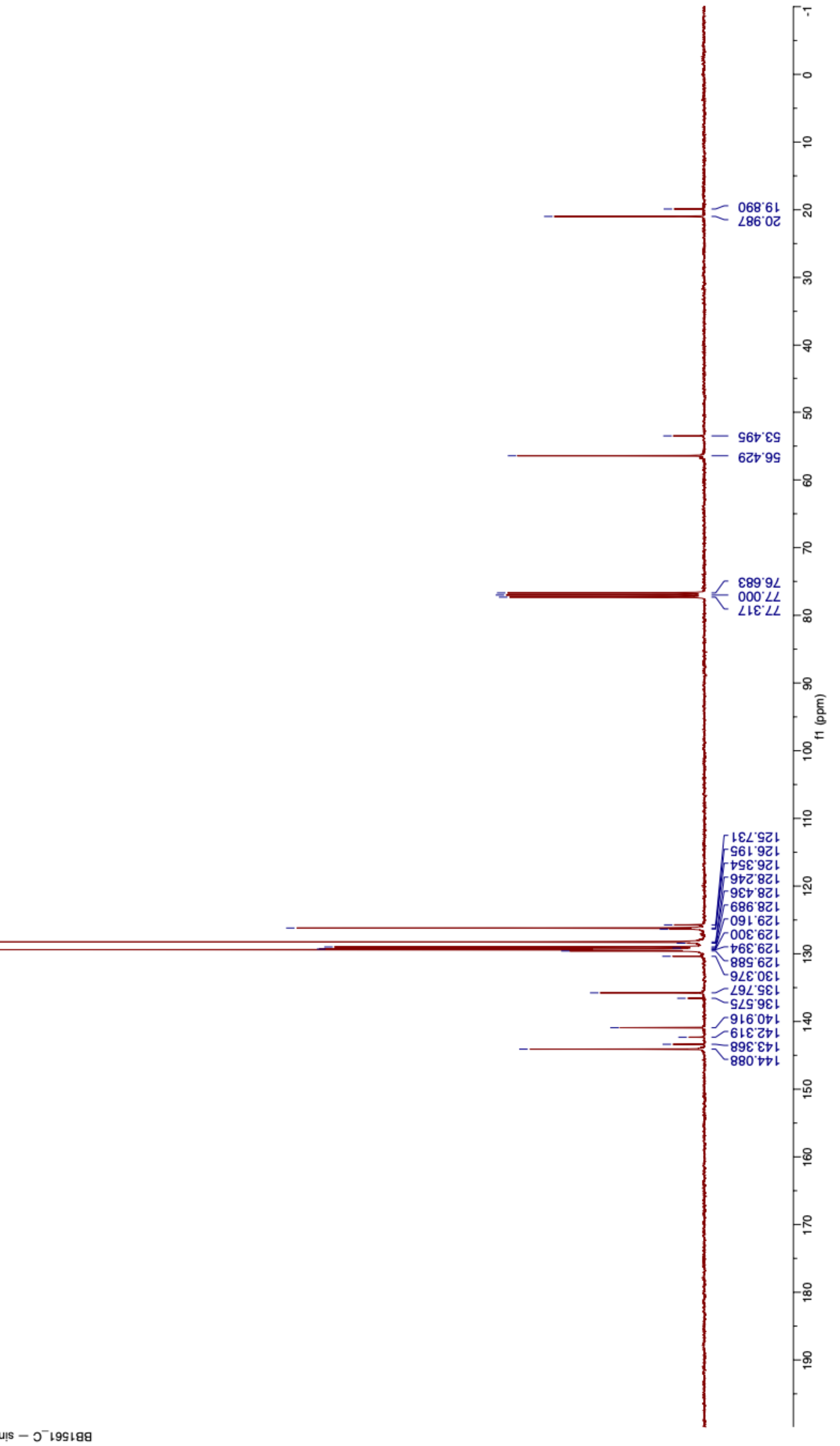

^{1}H NMR of **6B** (400 MHz, $CDCl_3$)

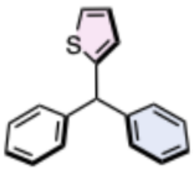

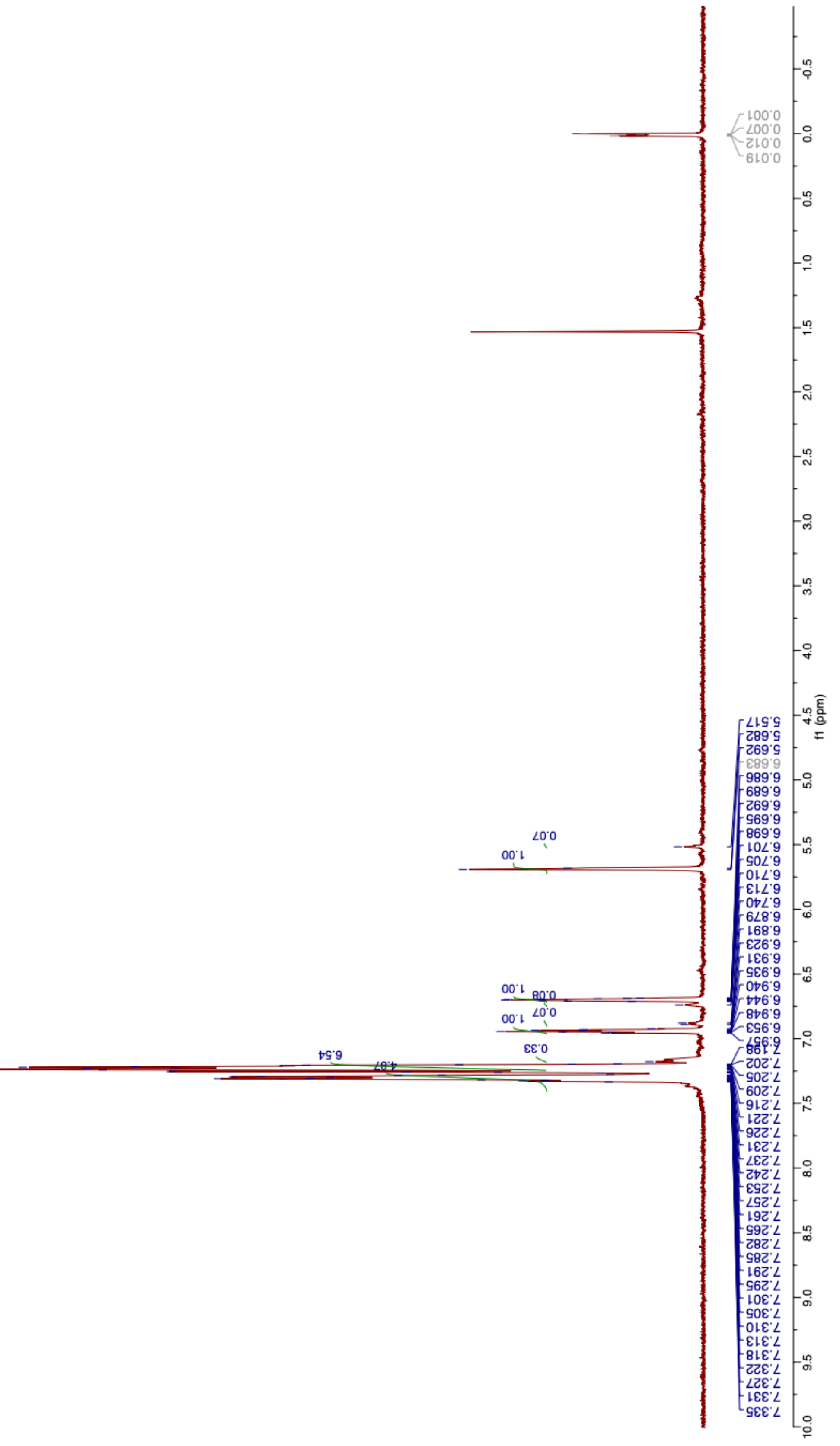

^{13}C NMR of **6B** (101 MHz, $CDCl_3$)

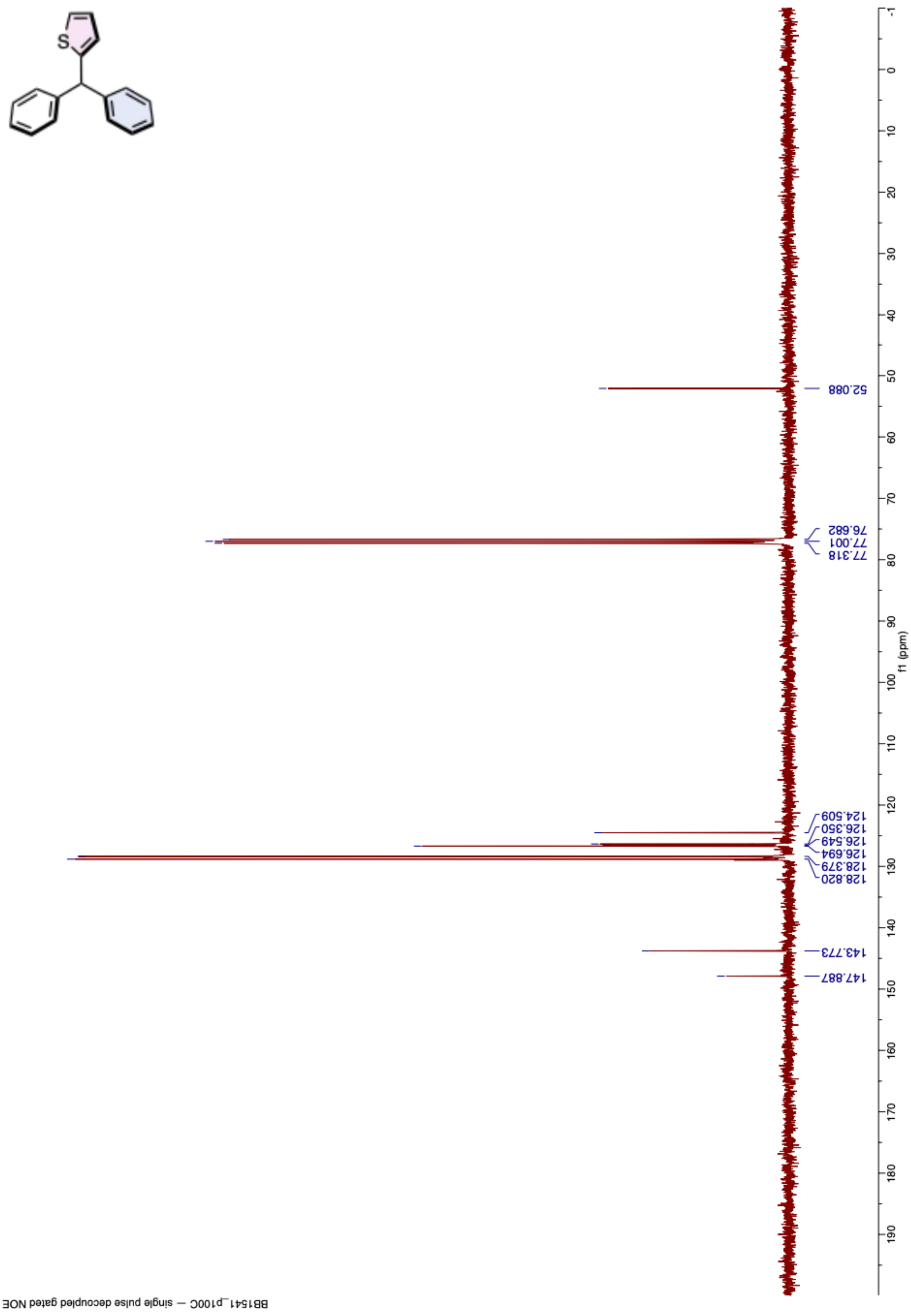

^{1}H NMR of **6C** (400 MHz, $CDCl_3$)

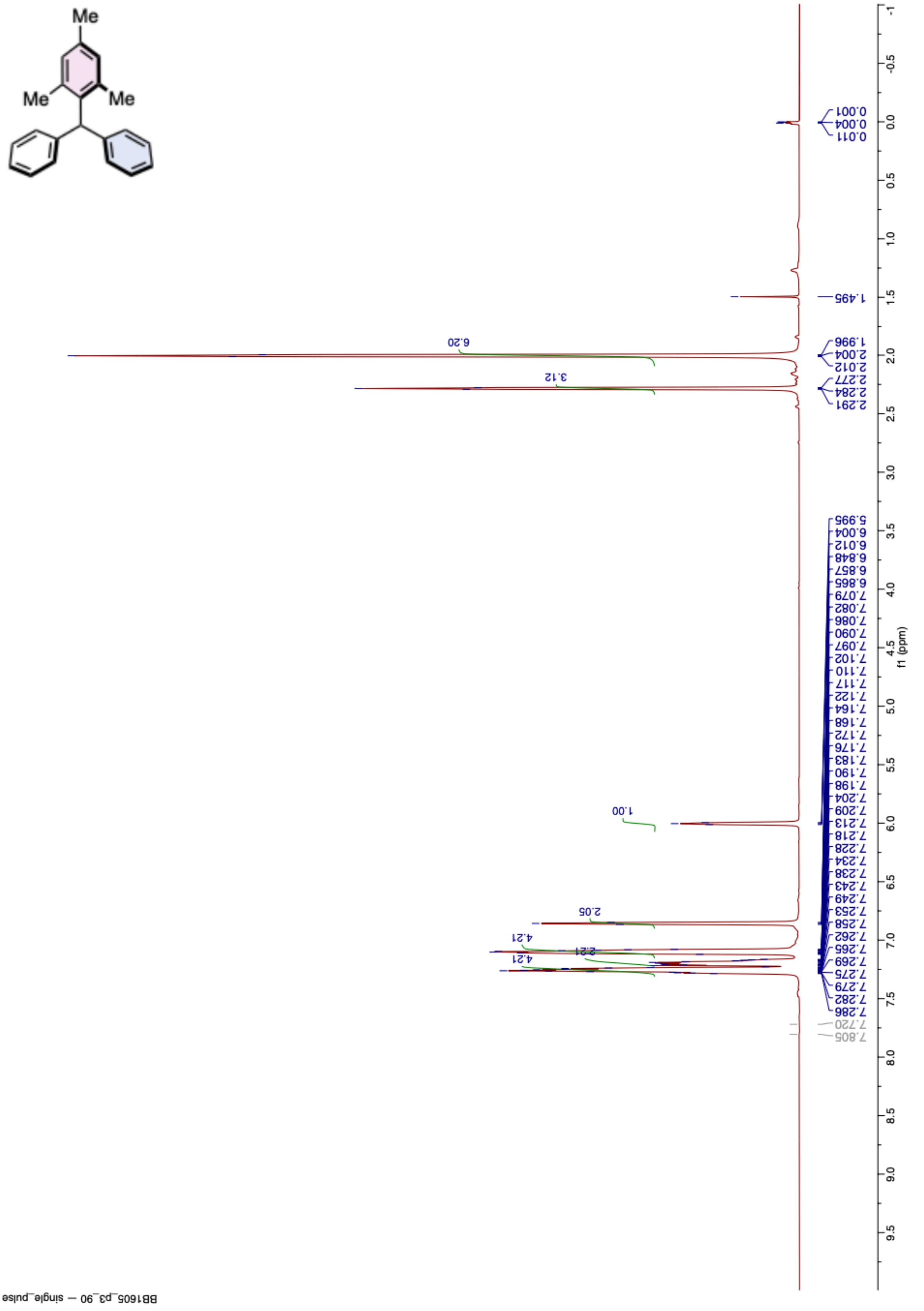

^{13}C NMR of **6C** (101 MHz, $CDCl_3$)

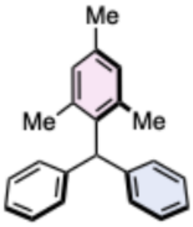

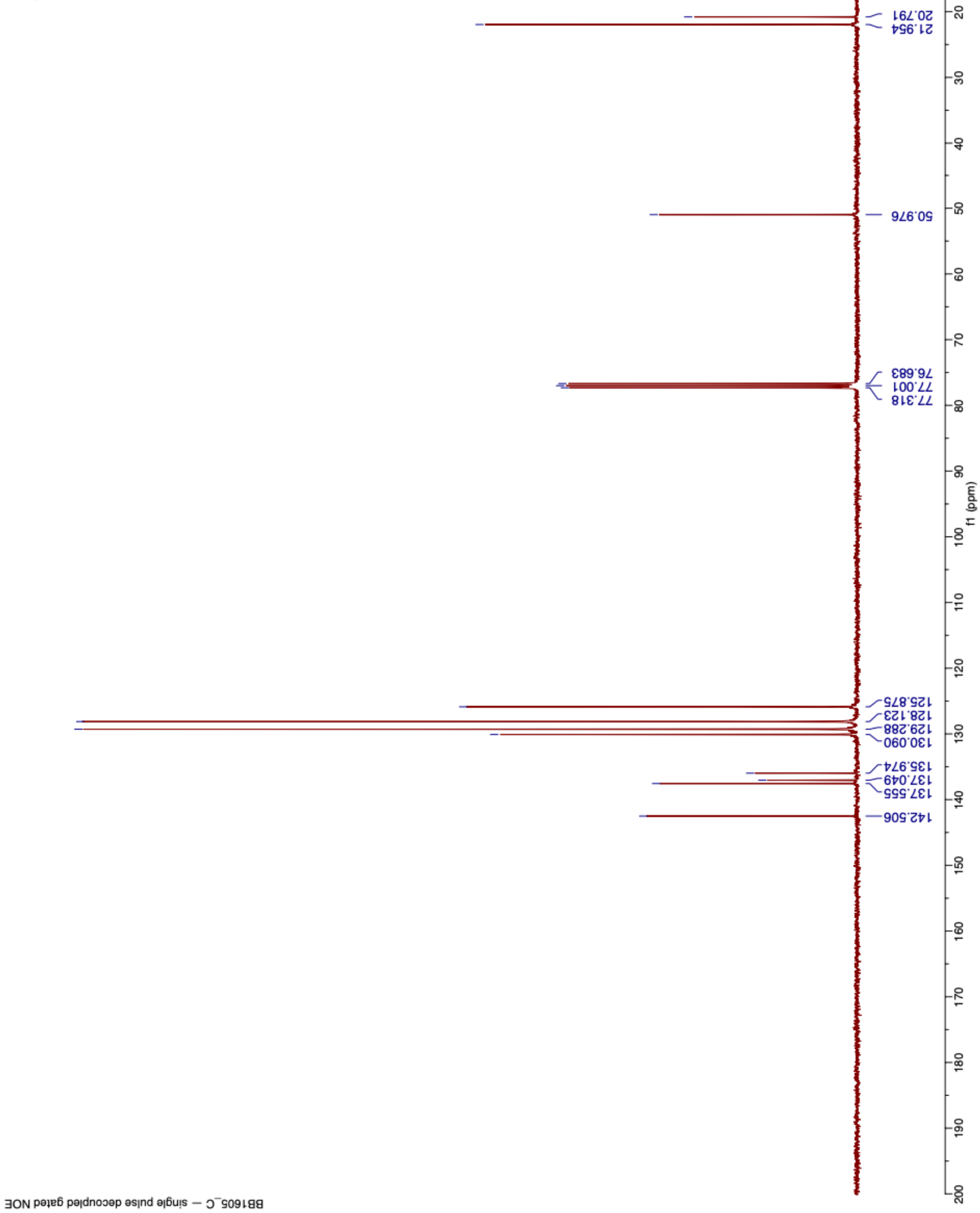

^{1}H NMR of **6D** (400 MHz, $CDCl_3$)

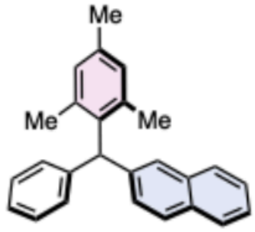

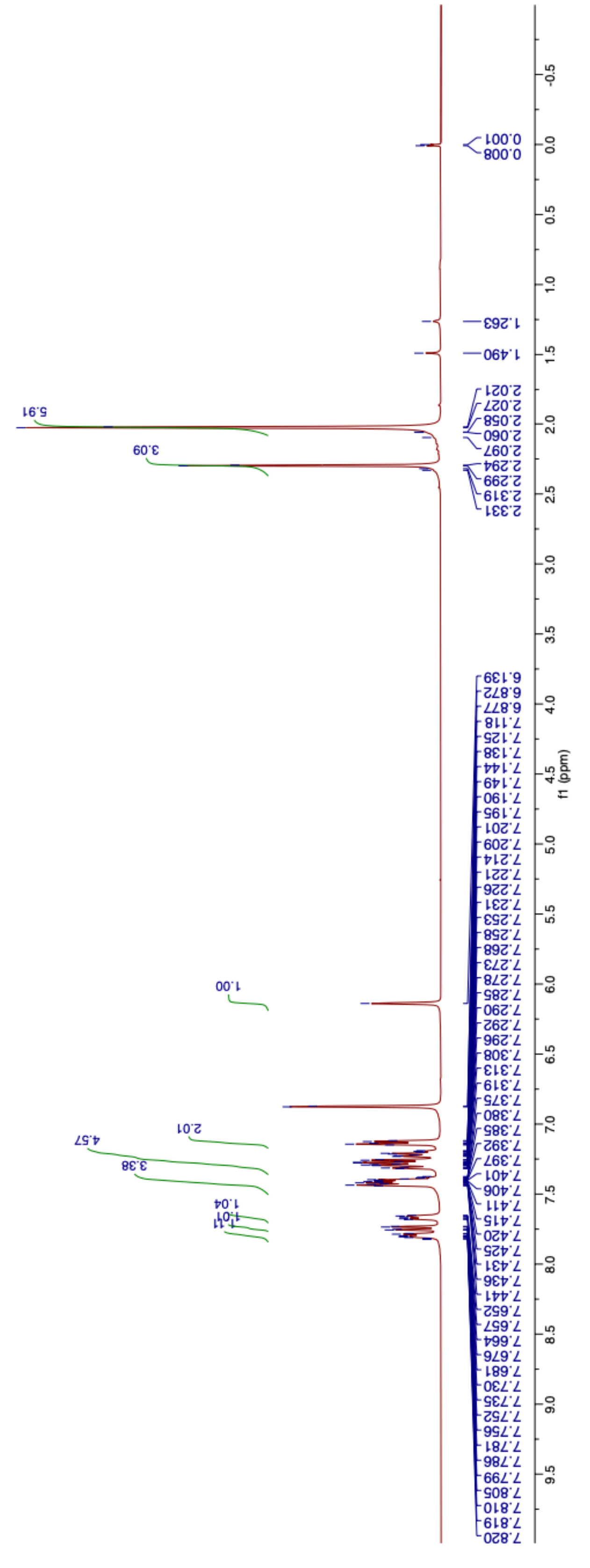

^{13}C NMR of **6D** (101 MHz, $CDCl_3$)

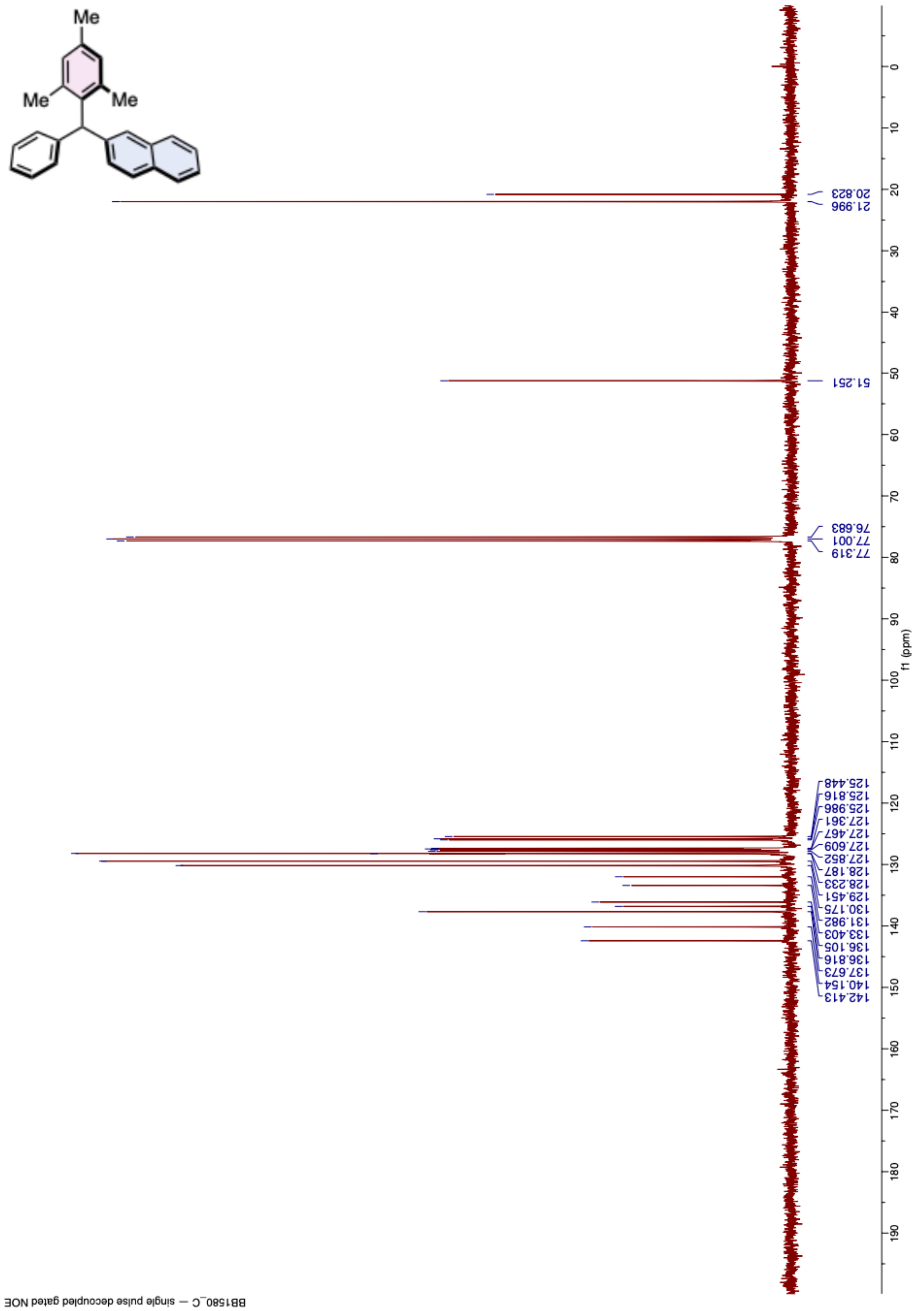

^{1}H NMR of **6E** (400 MHz, $CDCl_3$)

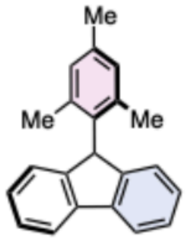

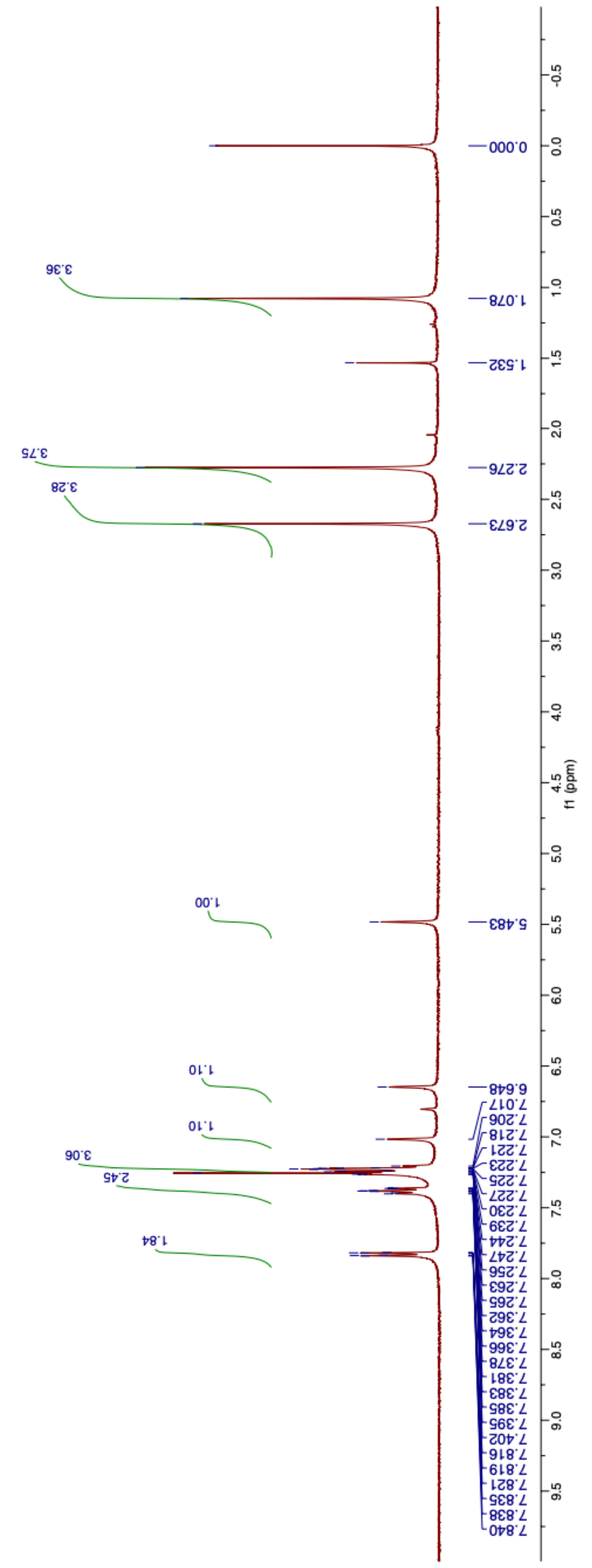

^{13}C NMR of **6E** (101 MHz, $CDCl_3$)

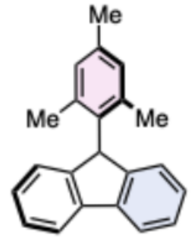

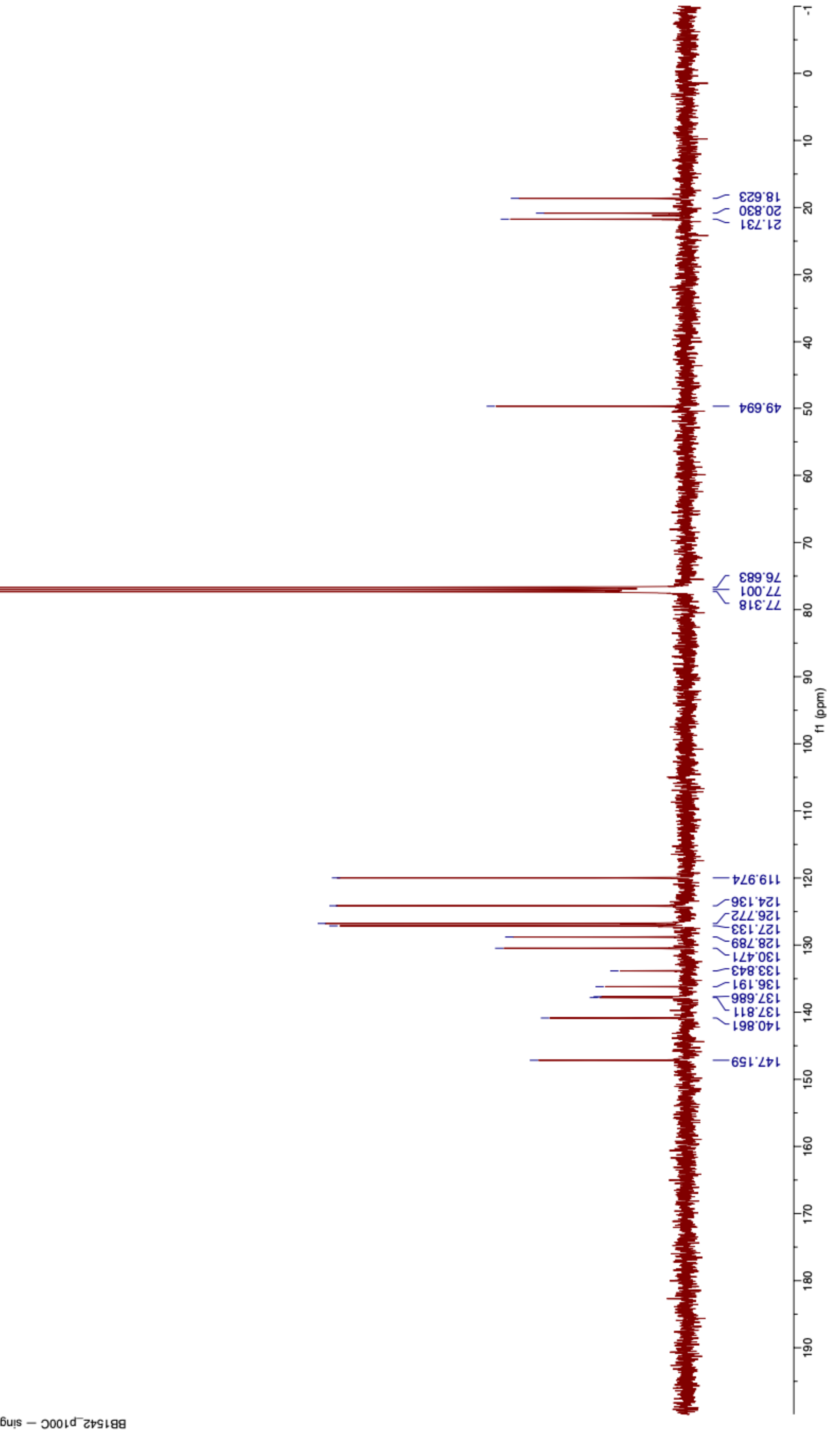

^{1}H NMR of **6F** (400 MHz, $CDCl_3$)

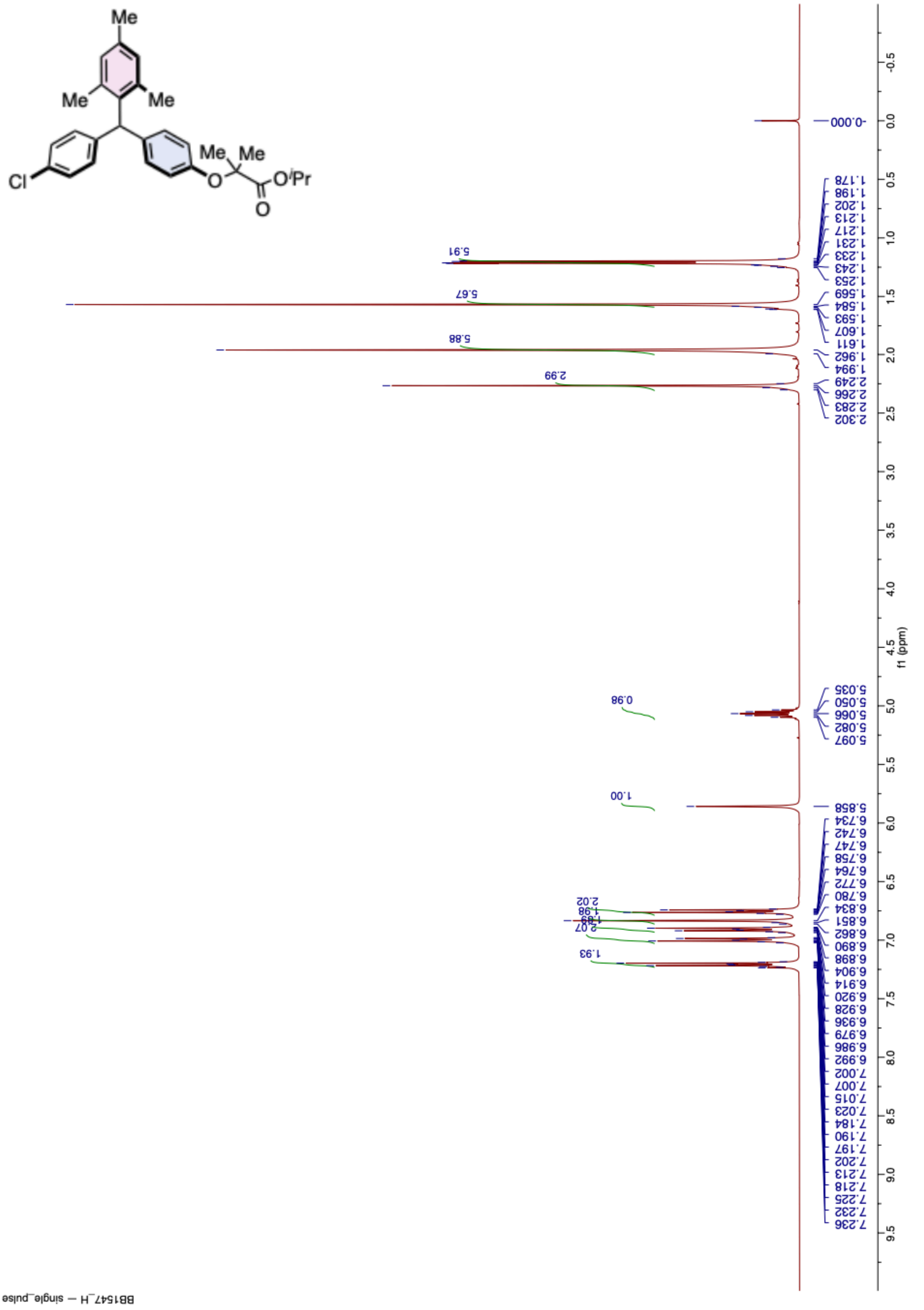

^{13}C NMR of **6F** (101 MHz, CDCl$_3$)

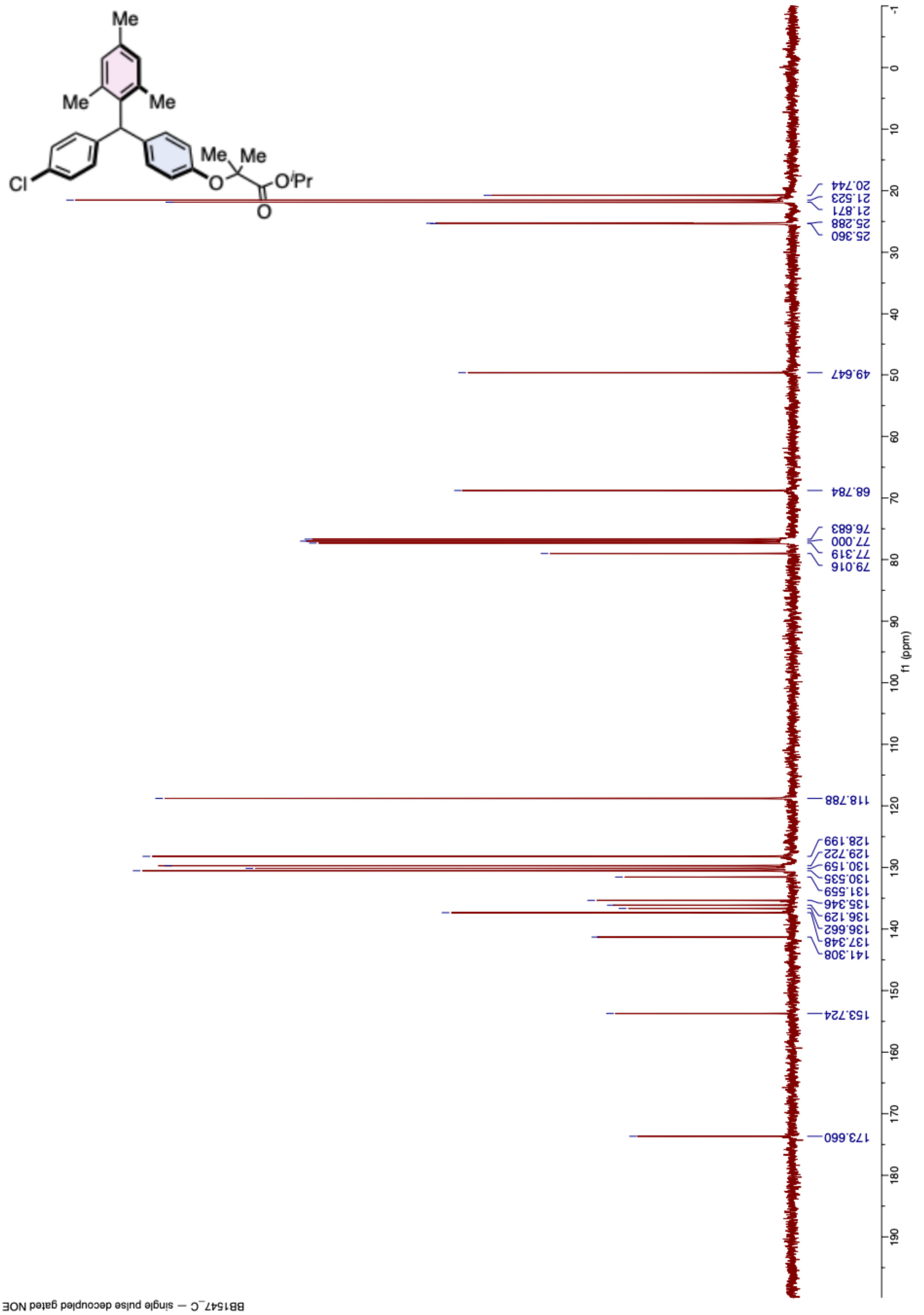

^{1}H NMR of **6G** (400 MHz, $CDCl_3$)

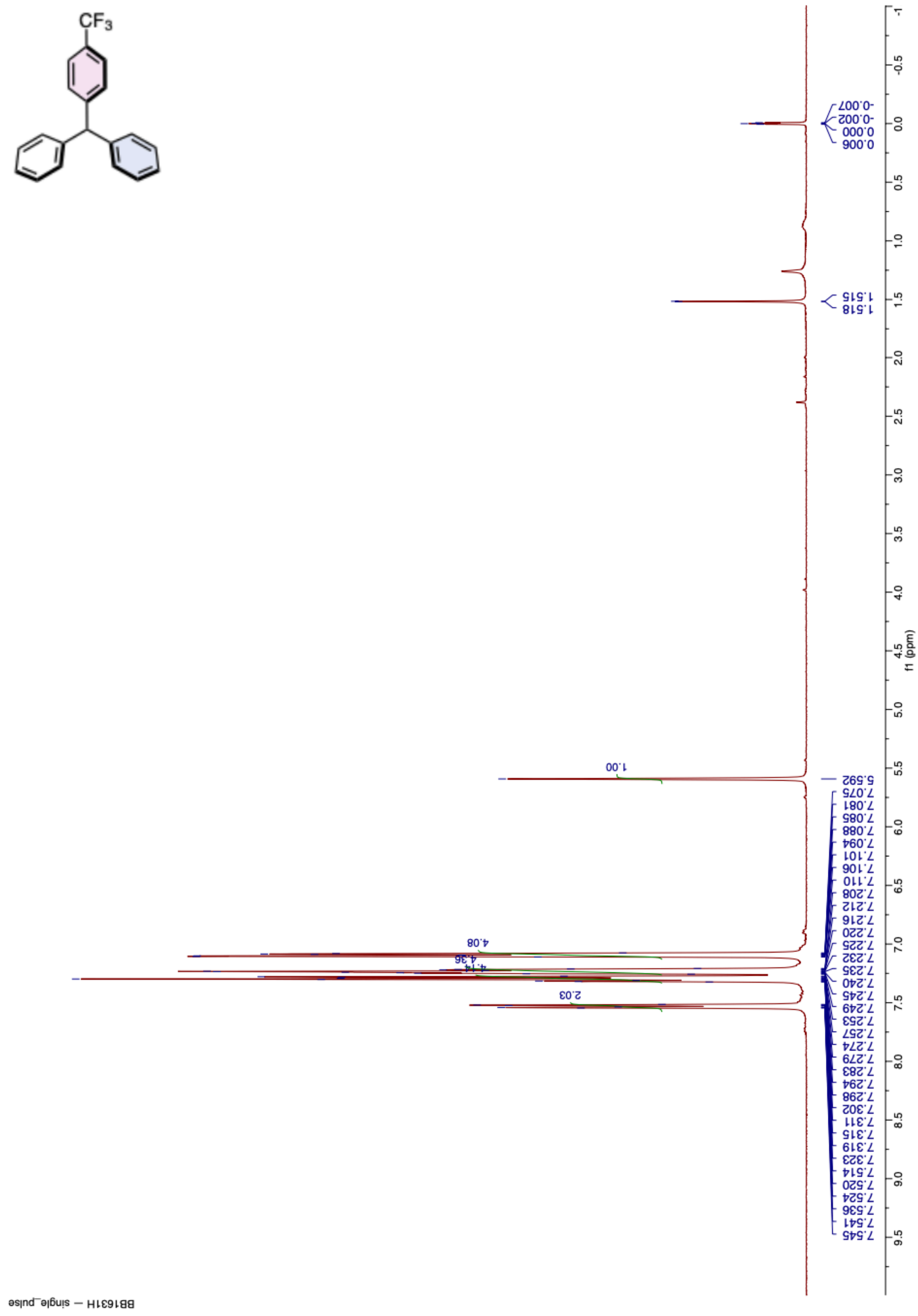

^{13}C NMR of **6G** (151 MHz, $CDCl_3$)

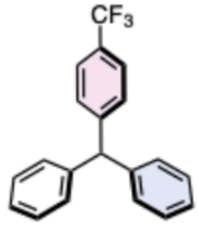

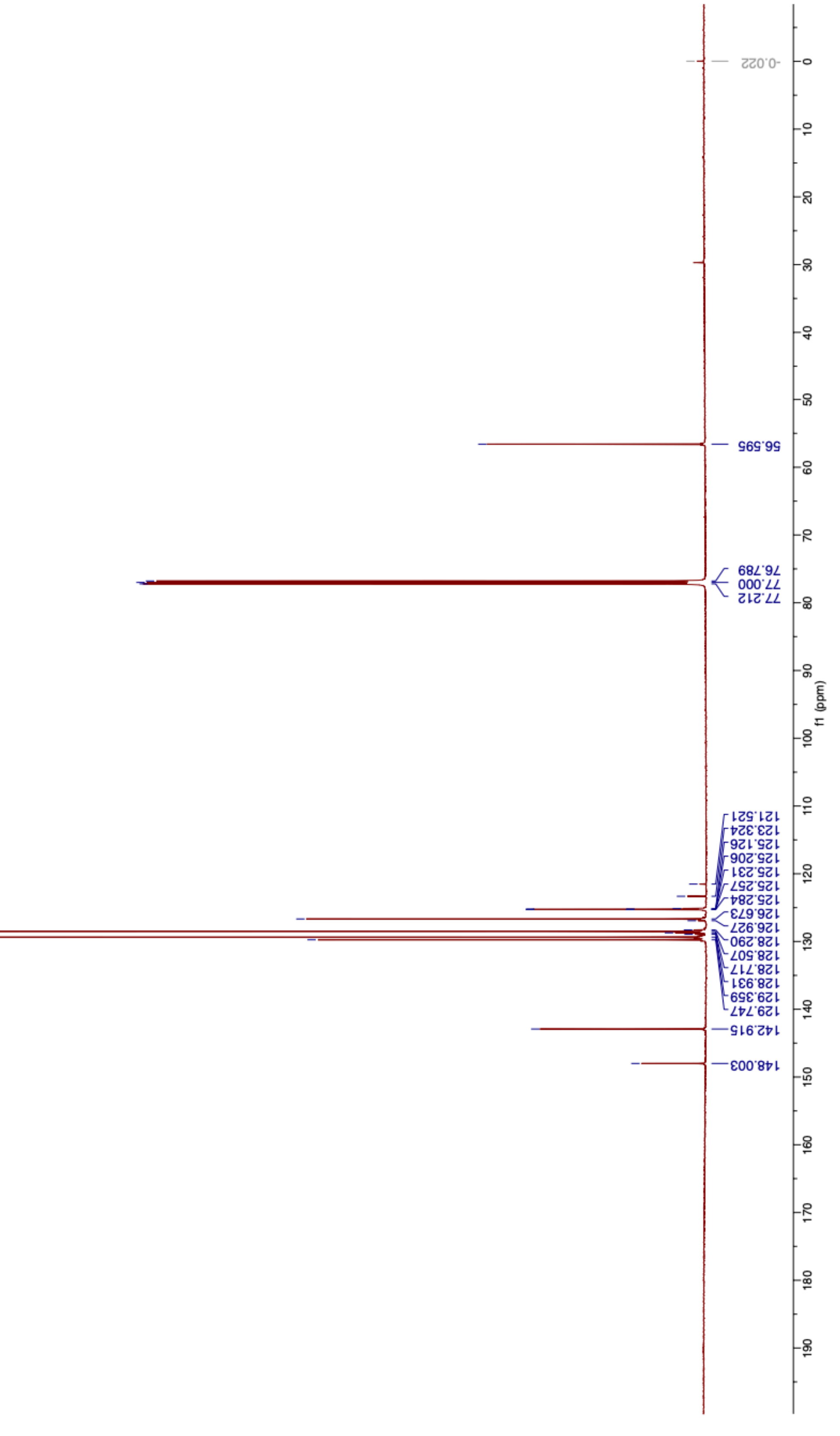

^{19}F NMR of **6G** (376 MHz, $CDCl_3$)

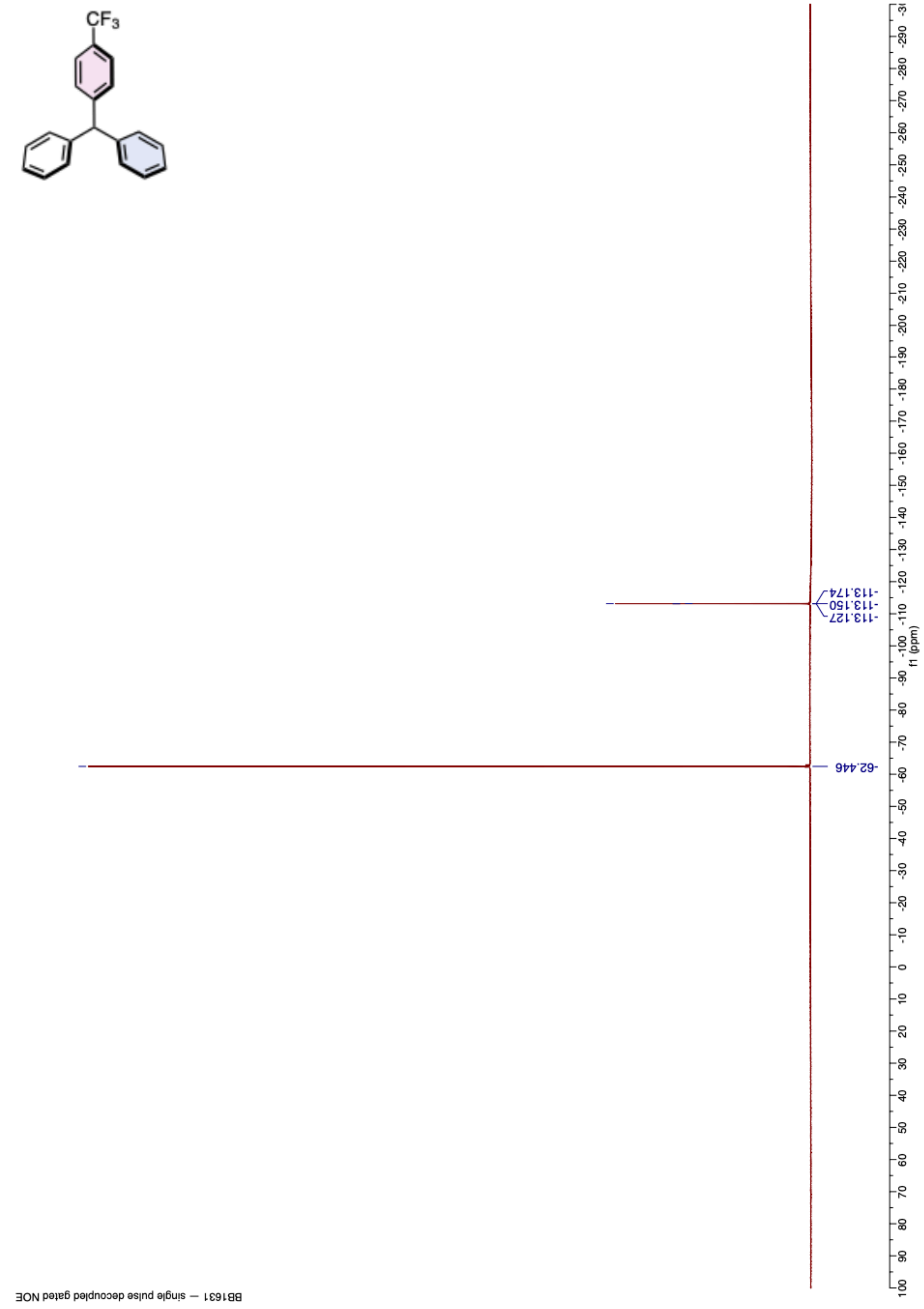

^{1}H NMR of **6H** (400 MHz, $CDCl_3$)

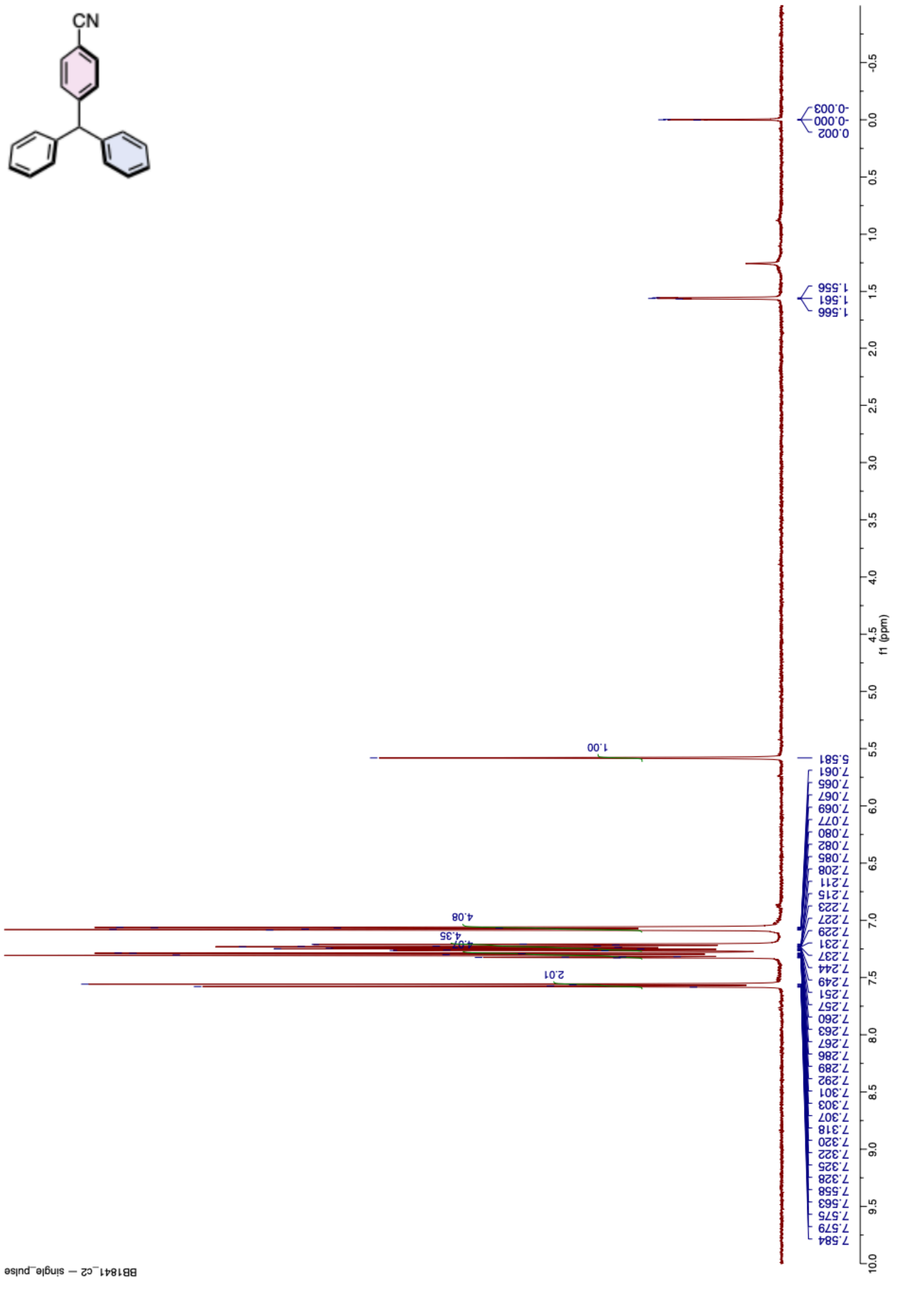

^{13}C NMR of **6H** (101 MHz, $CDCl_3$)

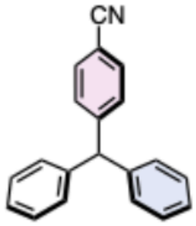

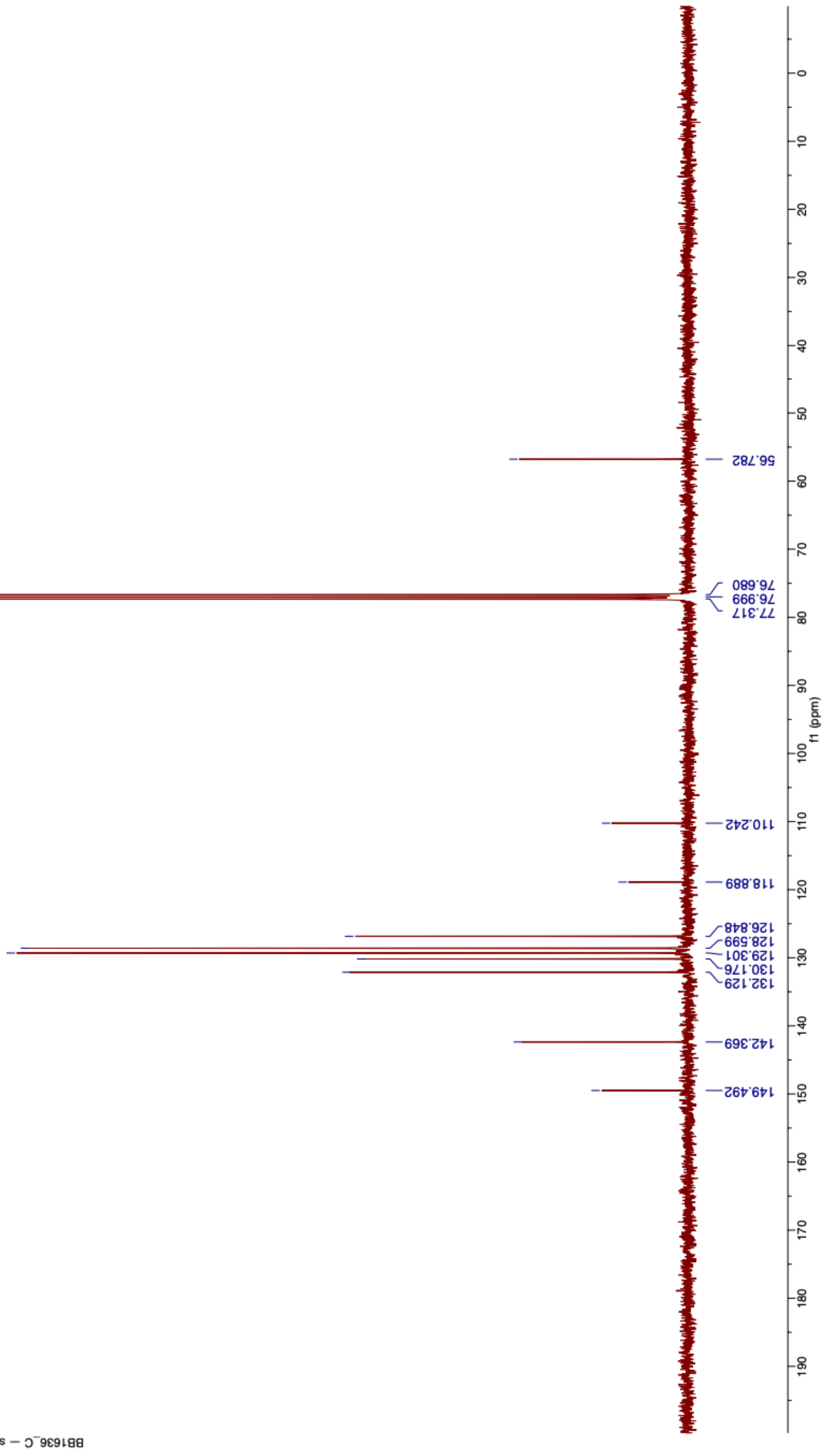

^{1}H NMR of **6l** (400 MHz, $CDCl_3$)

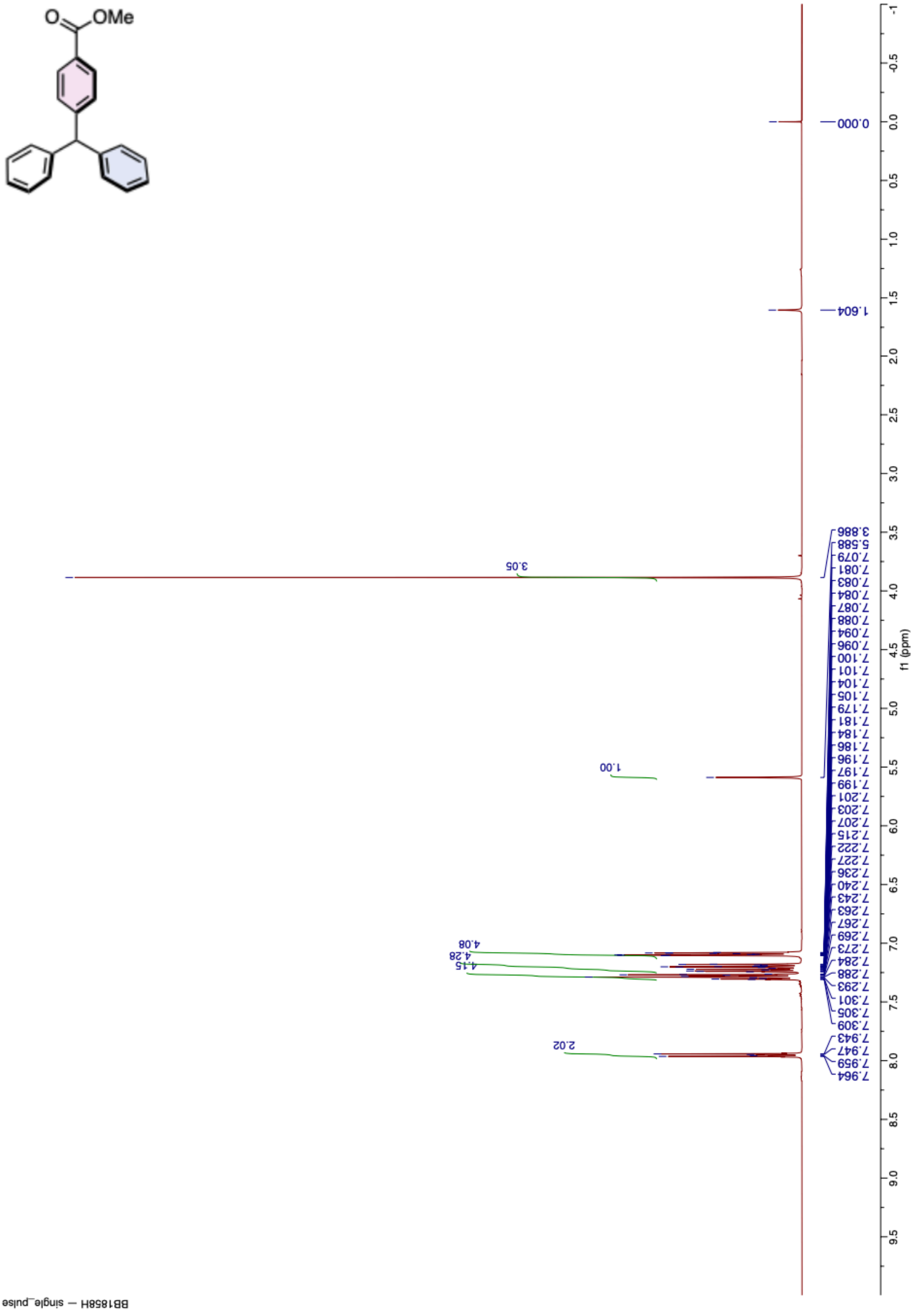

^{13}C NMR of **6l** (101 MHz, $CDCl_3$)

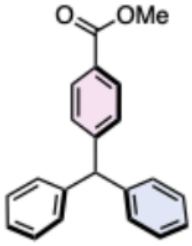

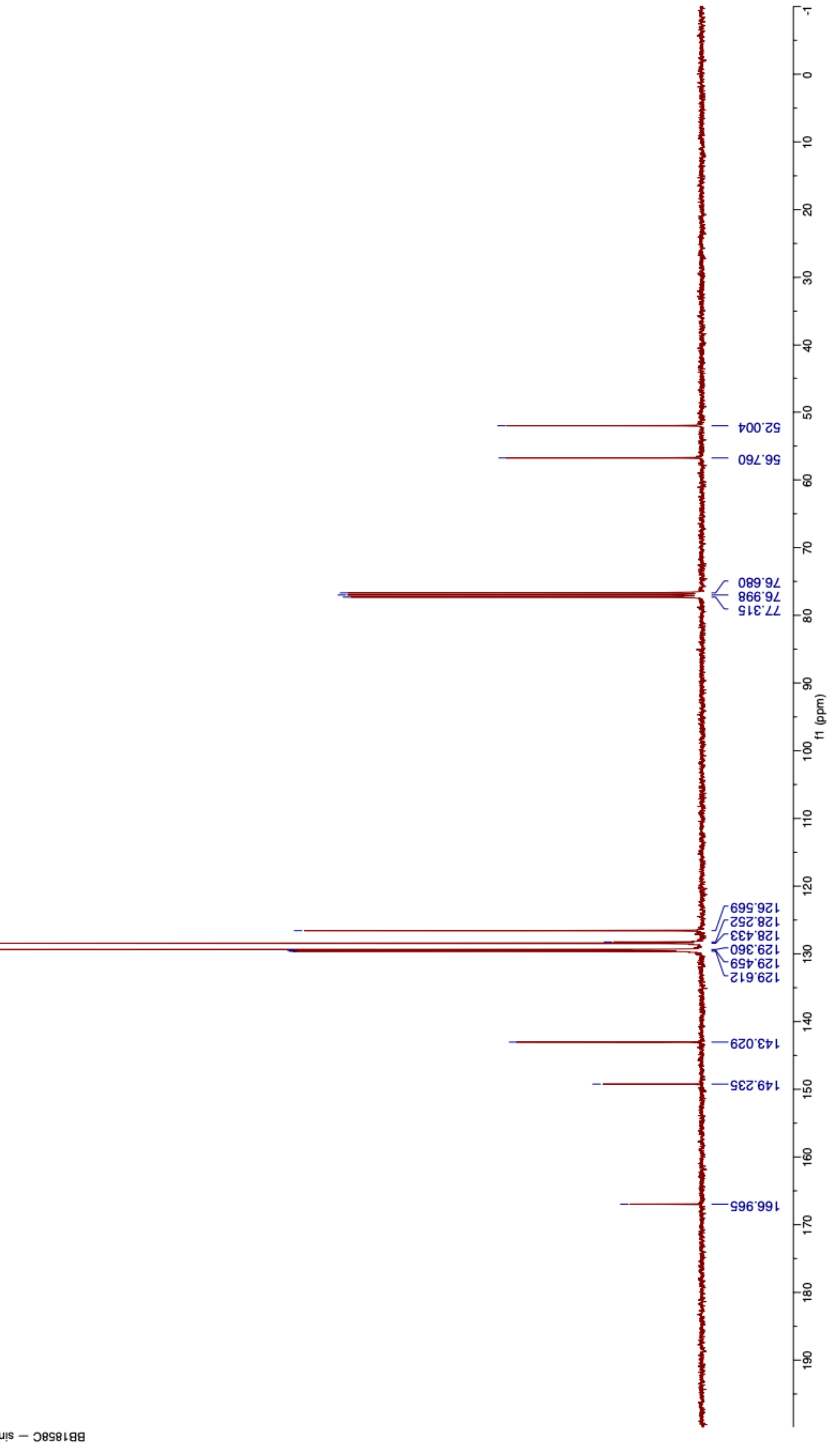

^{1}H NMR of **6J** (400 MHz, $CDCl_3$)

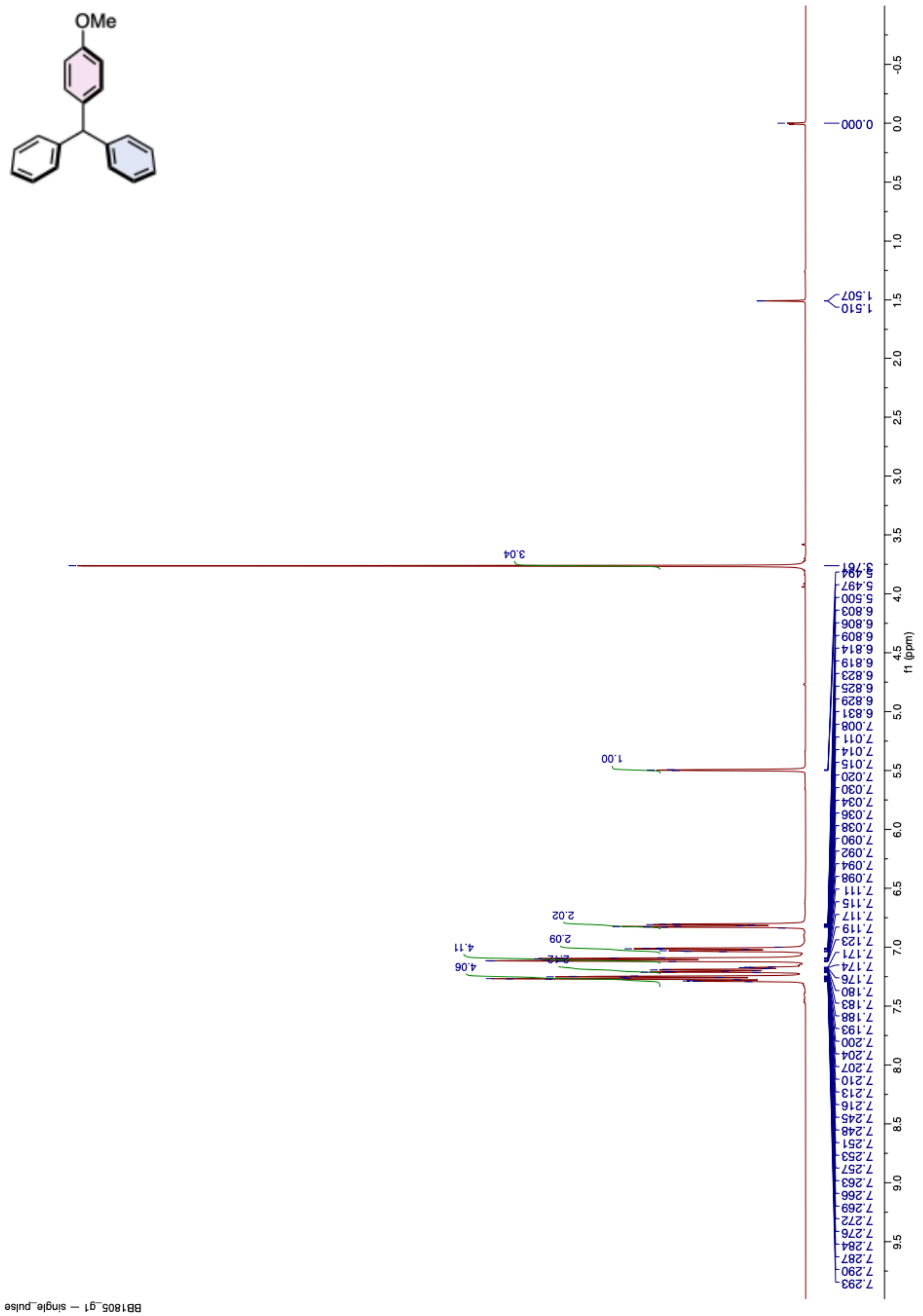

^{13}C NMR of **6J** (101 MHz, $CDCl_3$)

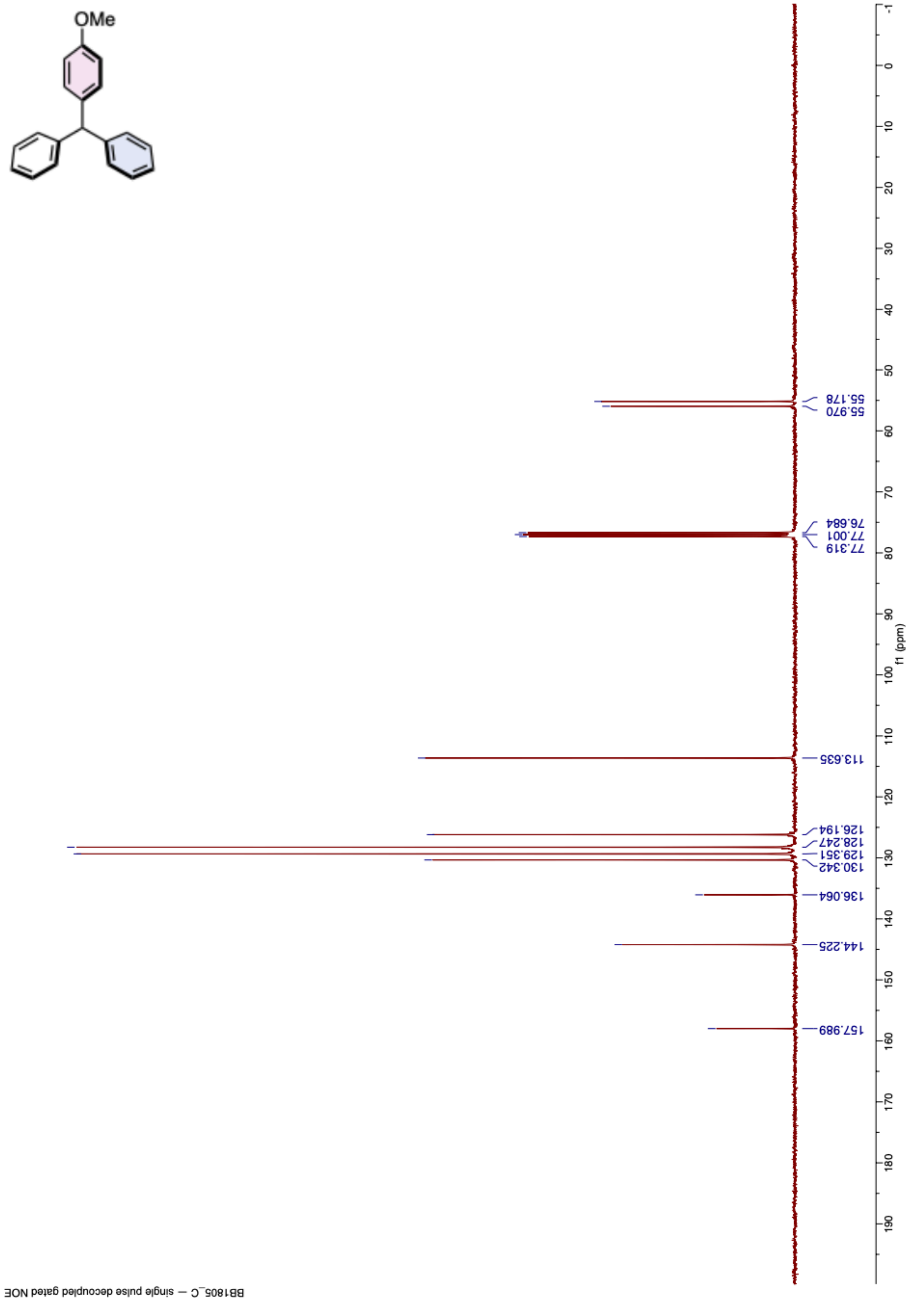

^{1}H NMR of **6K** (400 MHz, $CDCl_3$)

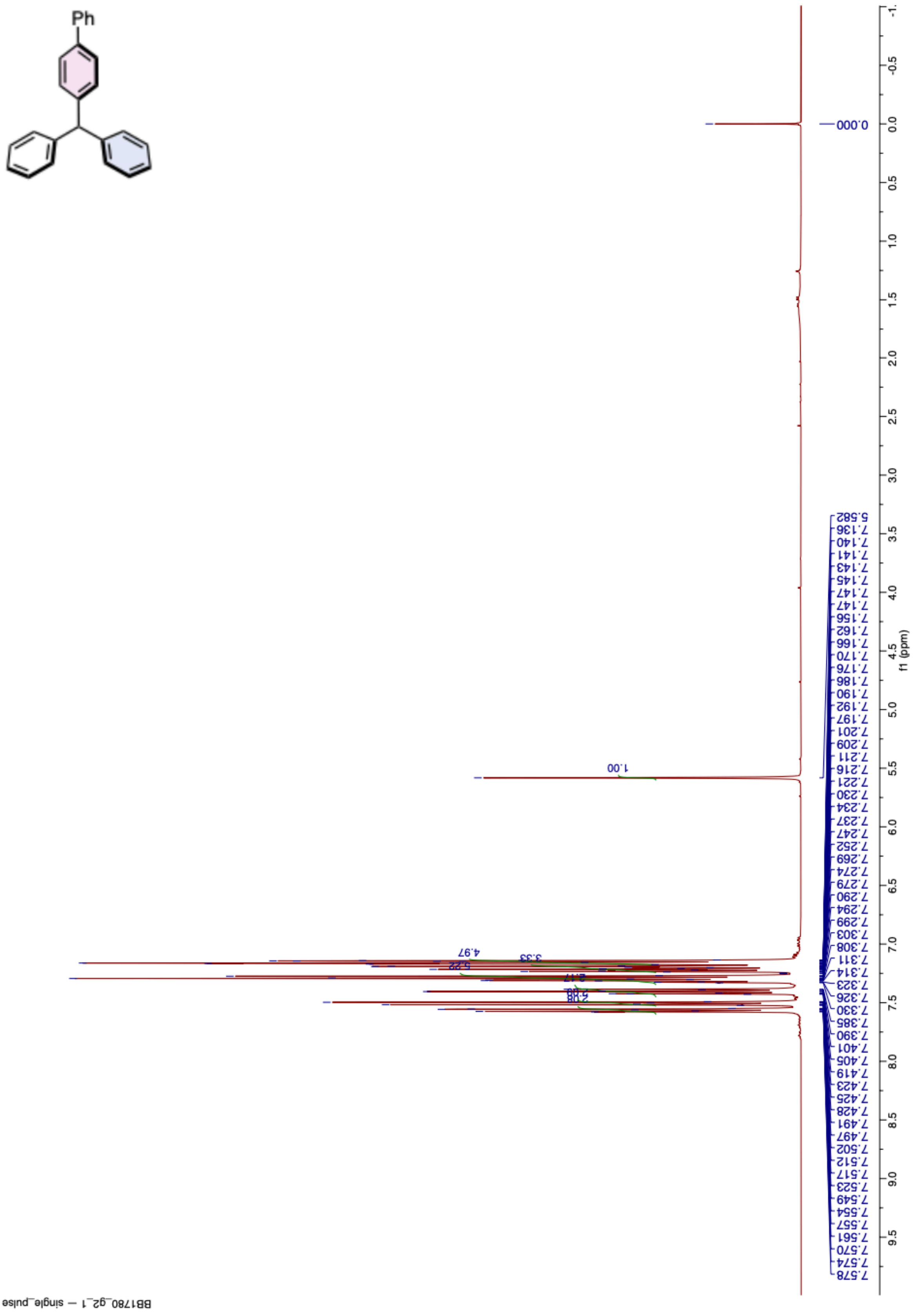

^{13}C NMR of **6K** (101 MHz, $CDCl_3$)

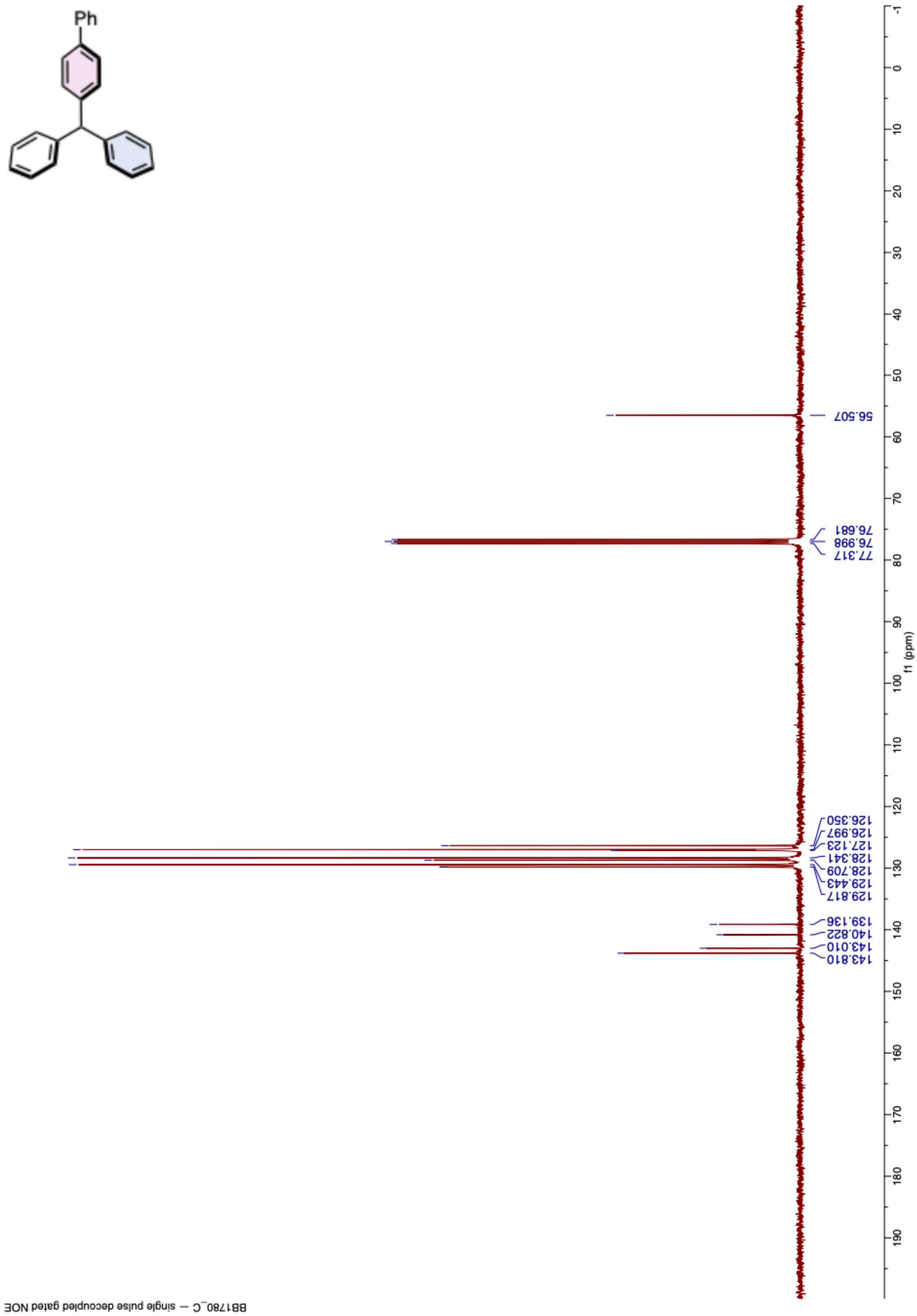

^{1}H NMR of **6L** (400 MHz, $CDCl_3$)

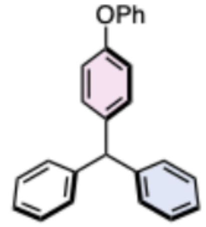

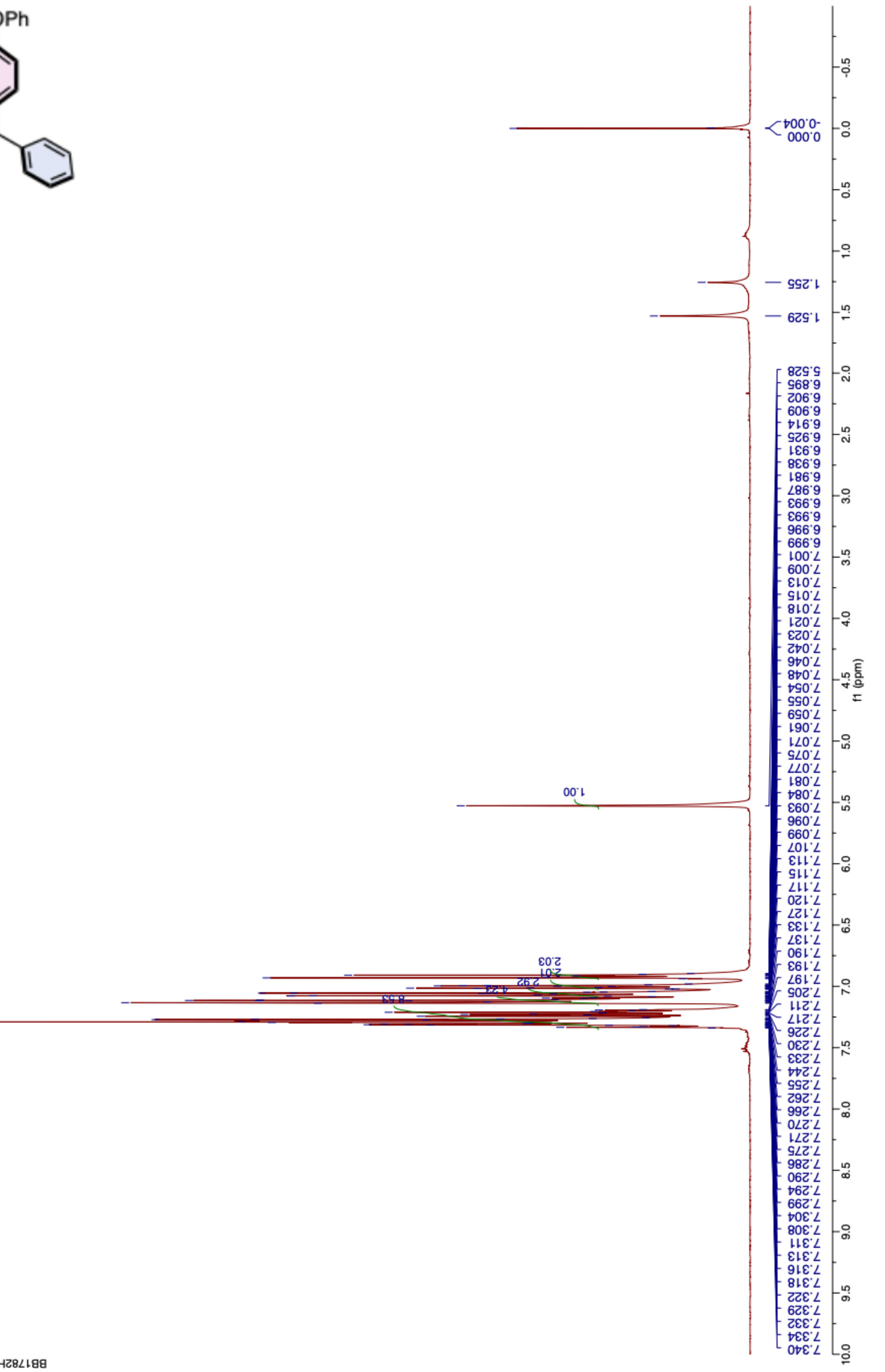

^{13}C NMR of **6L** (101 MHz, $CDCl_3$)

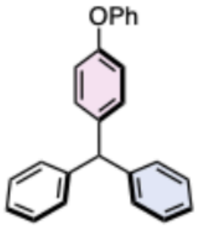

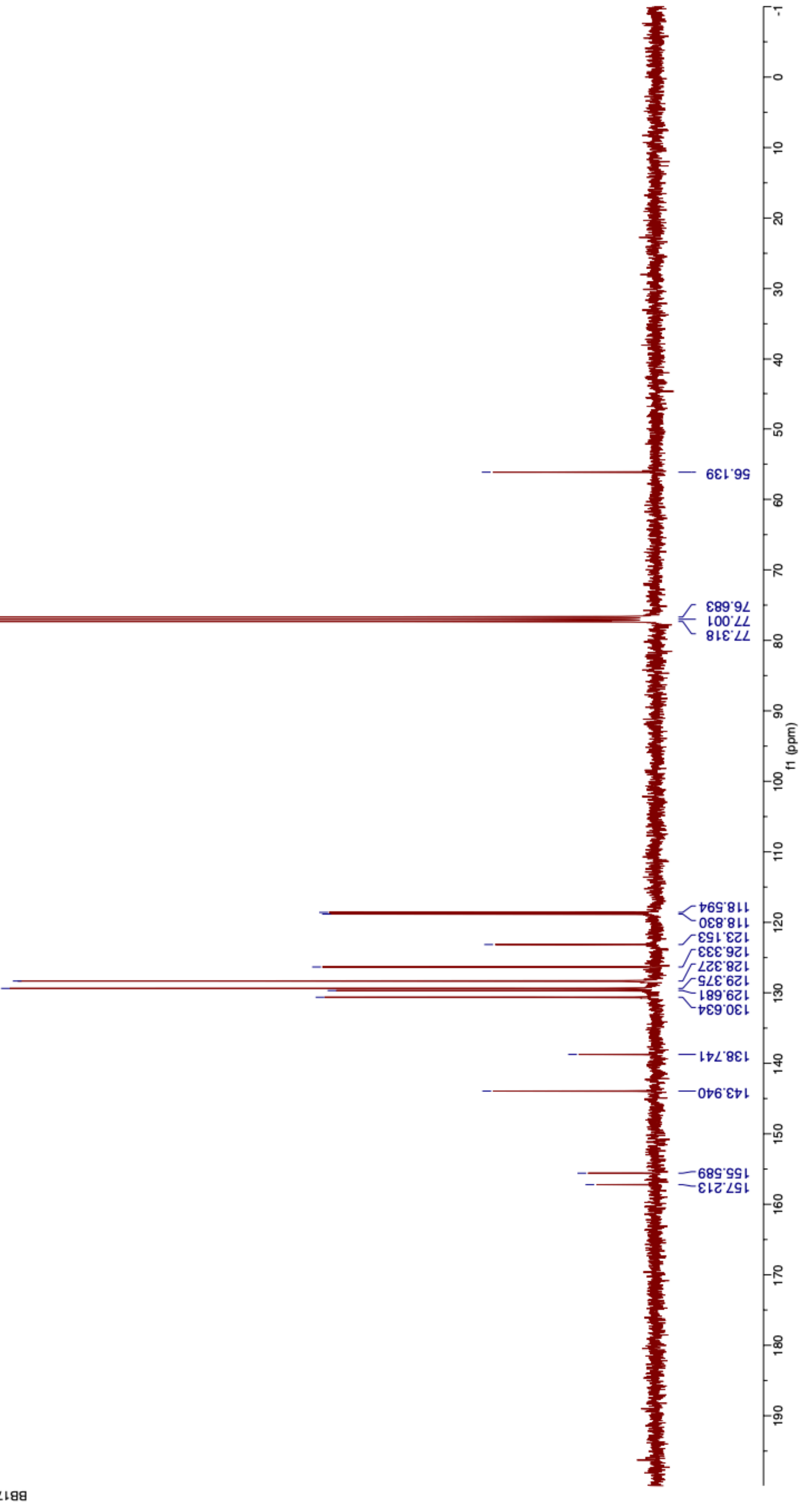

^{1}H NMR of **8** (400 MHz, $CDCl_3$)

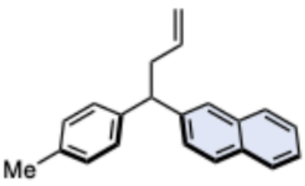

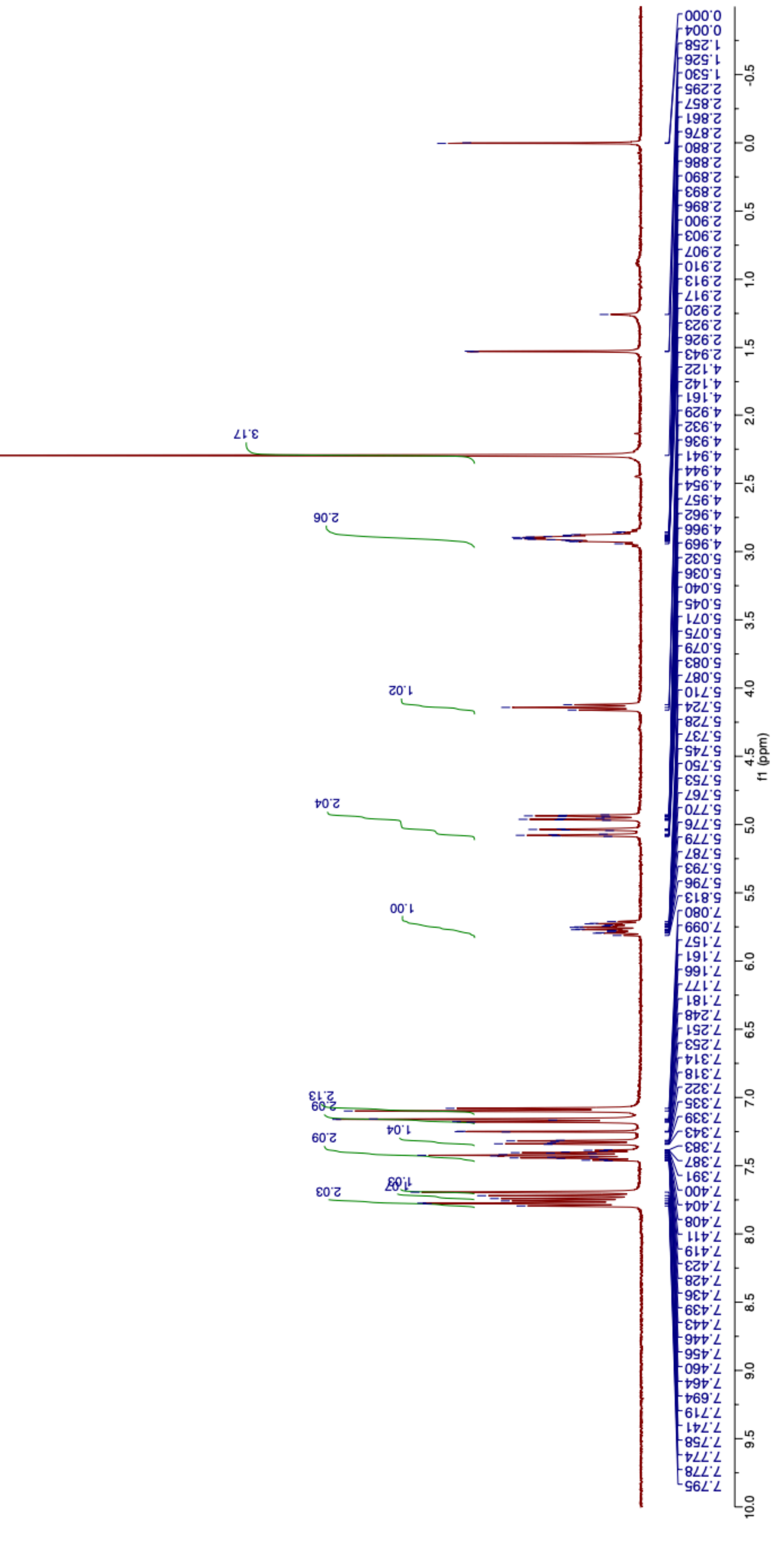

^{13}C NMR of **8** (101 MHz, $CDCl_3$)

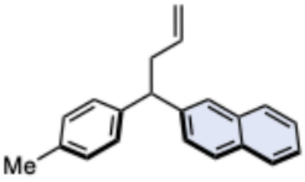

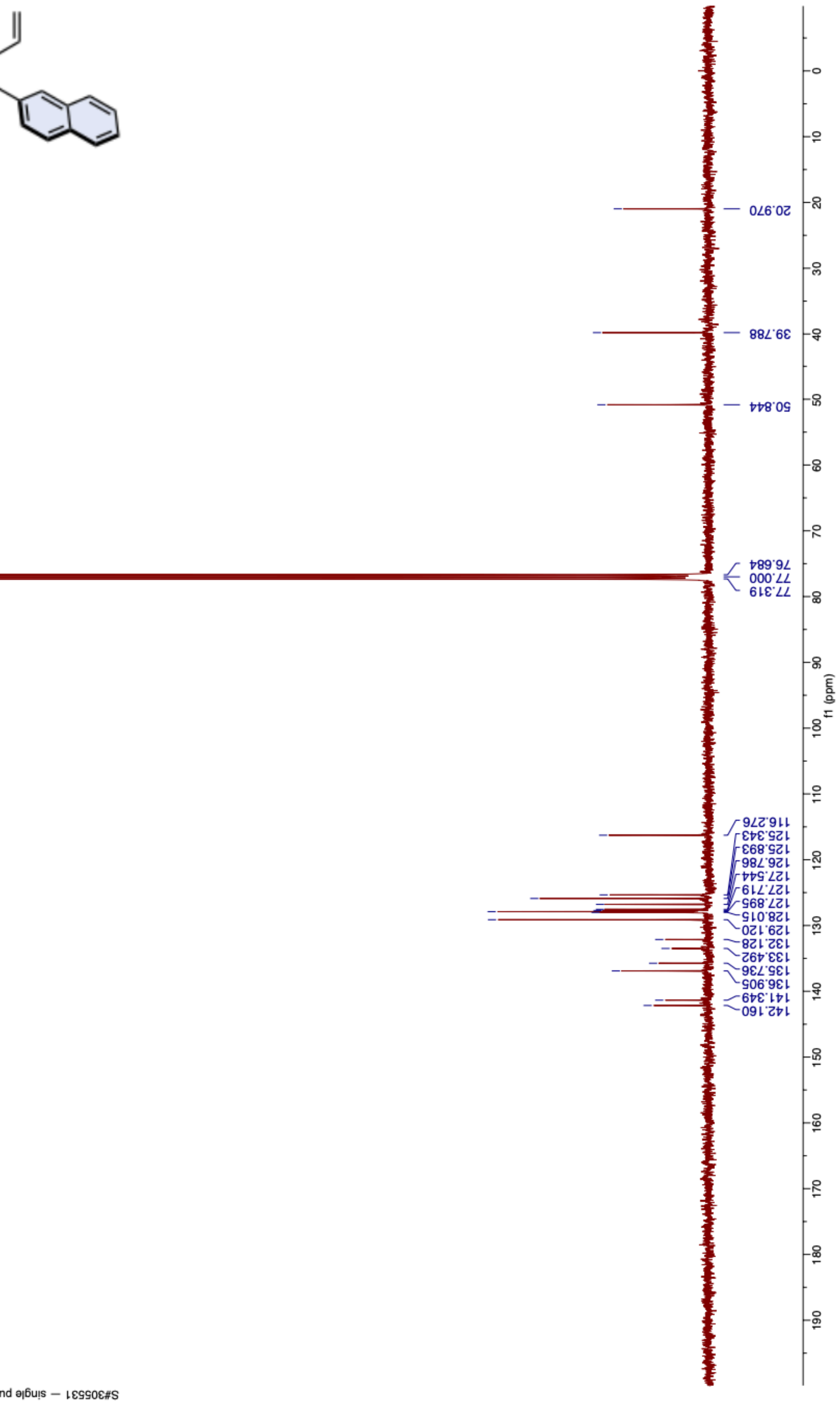

^{1}H NMR of **10** (400 MHz, $CDCl_3$)

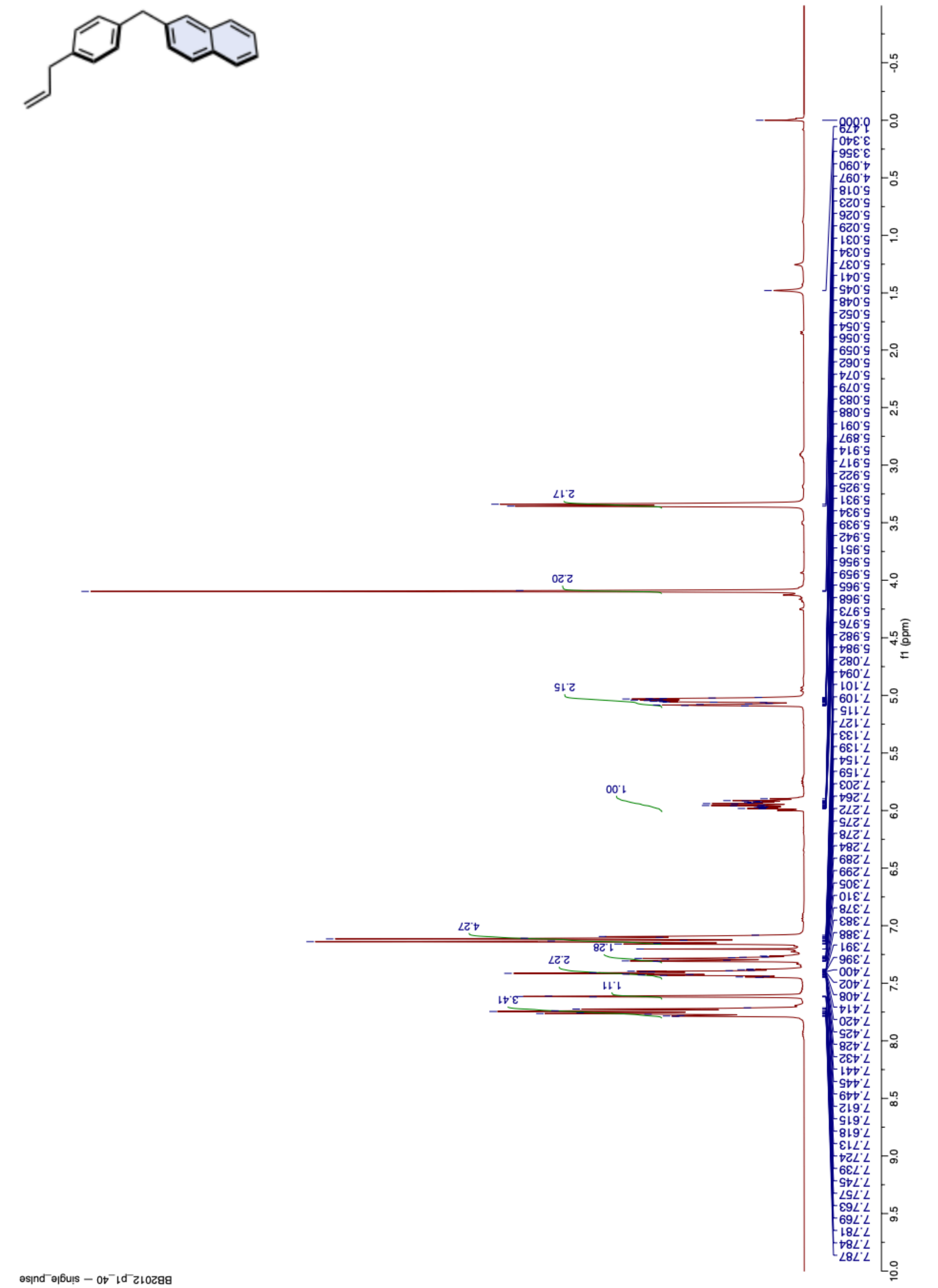

^{13}C NMR of **10** (101 MHz, $CDCl_3$)

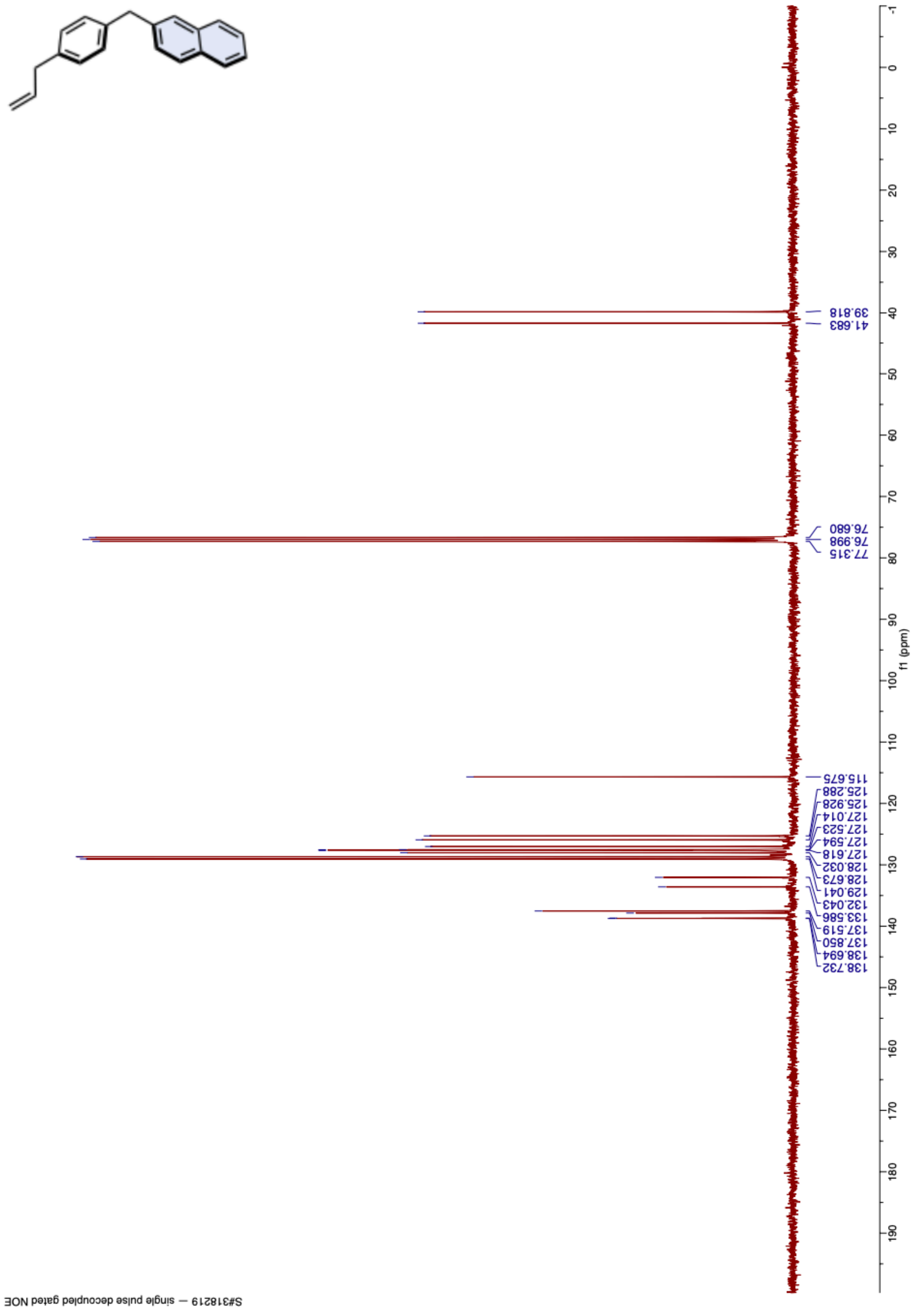

^{1}H NMR of **16** (400 MHz, $CDCl_3$)

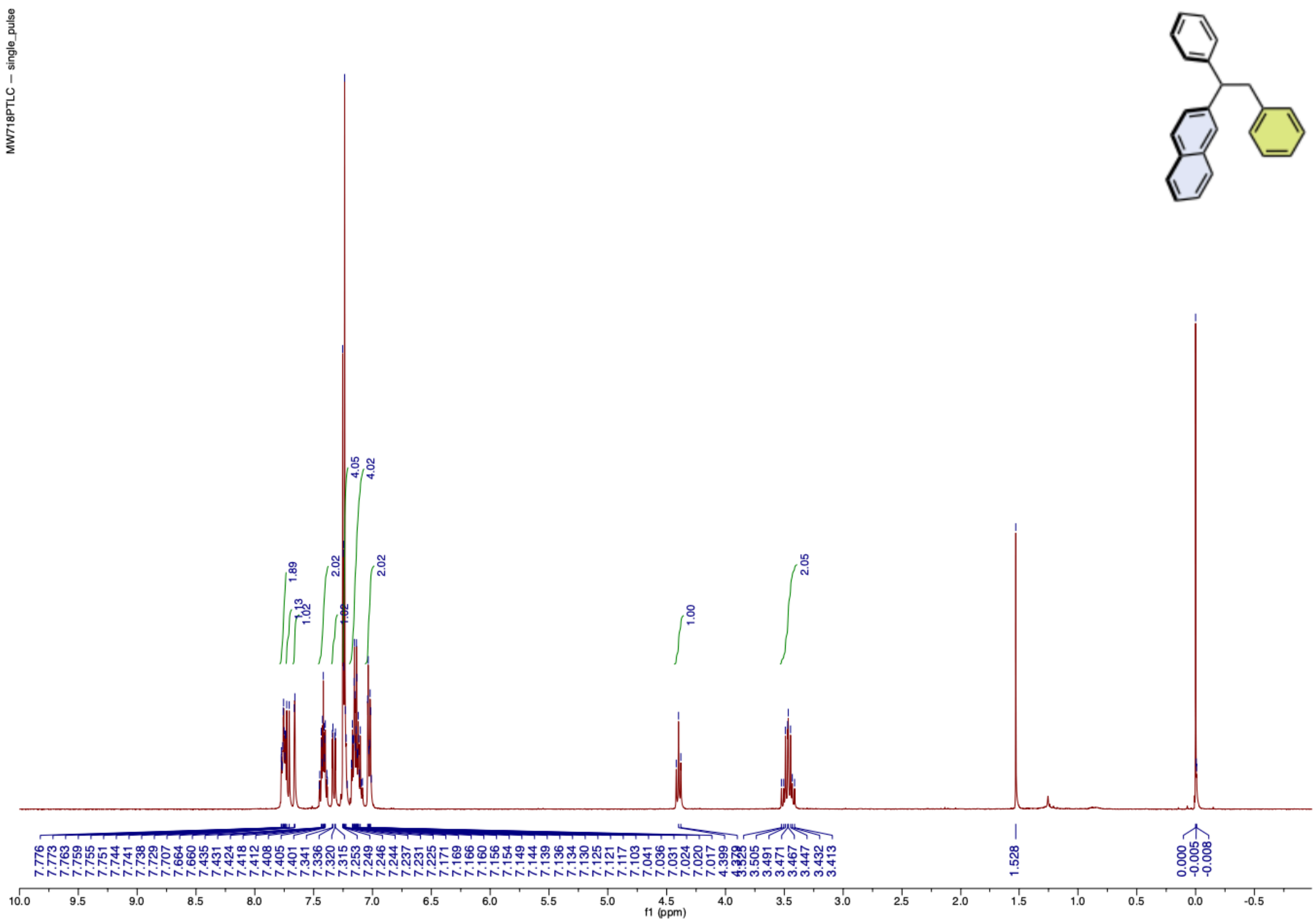
MW718PTLC — single_pulse
1.89
1.13
1.02
2.02
1.02
4.05
4.02
2.02
1.00
2.05
7.776
7.773
7.763
7.759
7.755
7.751
7.744
7.741
7.738
7.729
7.707
7.664
7.660
7.435
7.431
7.424
7.418
7.412
7.408
7.405
7.401
7.341
7.336
7.320
7.315
7.253
7.249
7.246
7.244
7.237
7.231
7.225
7.171
7.169
7.166
7.160
7.156
7.154
7.149
7.144
7.139
7.136
7.134
7.130
7.125
7.121
7.117
7.103
7.041
7.036
7.031
7.024
7.020
7.017
4.399
4.379
3.528
3.505
3.491
3.471
3.467
3.447
3.432
3.413
1.528
0.000
-0.005
-0.008
10.0
9.5
9.0
8.5
8.0
7.5
7.0
6.5
6.0
5.5
5.0
4.5
4.0
3.5
3.0
2.5
2.0
1.5
1.0
0.5
0.0
-0.5
f1 (ppm)

^{13}C NMR of **16** (101 MHz, $CDCl_3$)

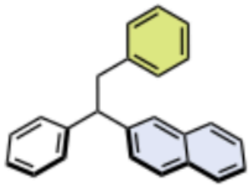

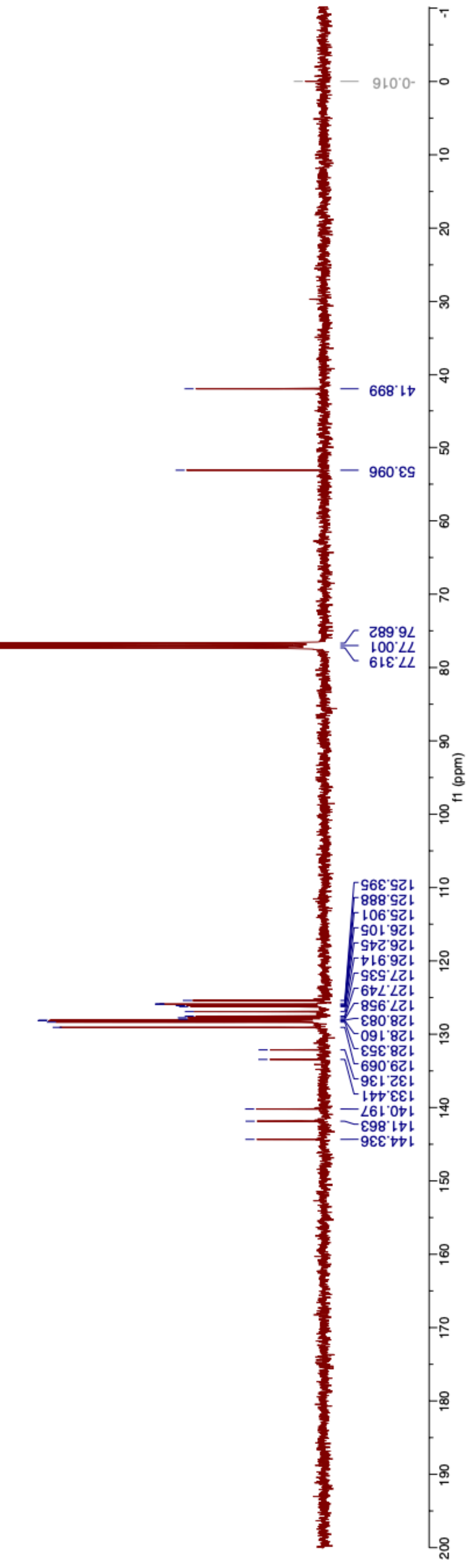

^{1}H NMR of **18** (400 MHz, $CDCl_3$)

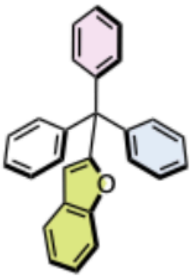

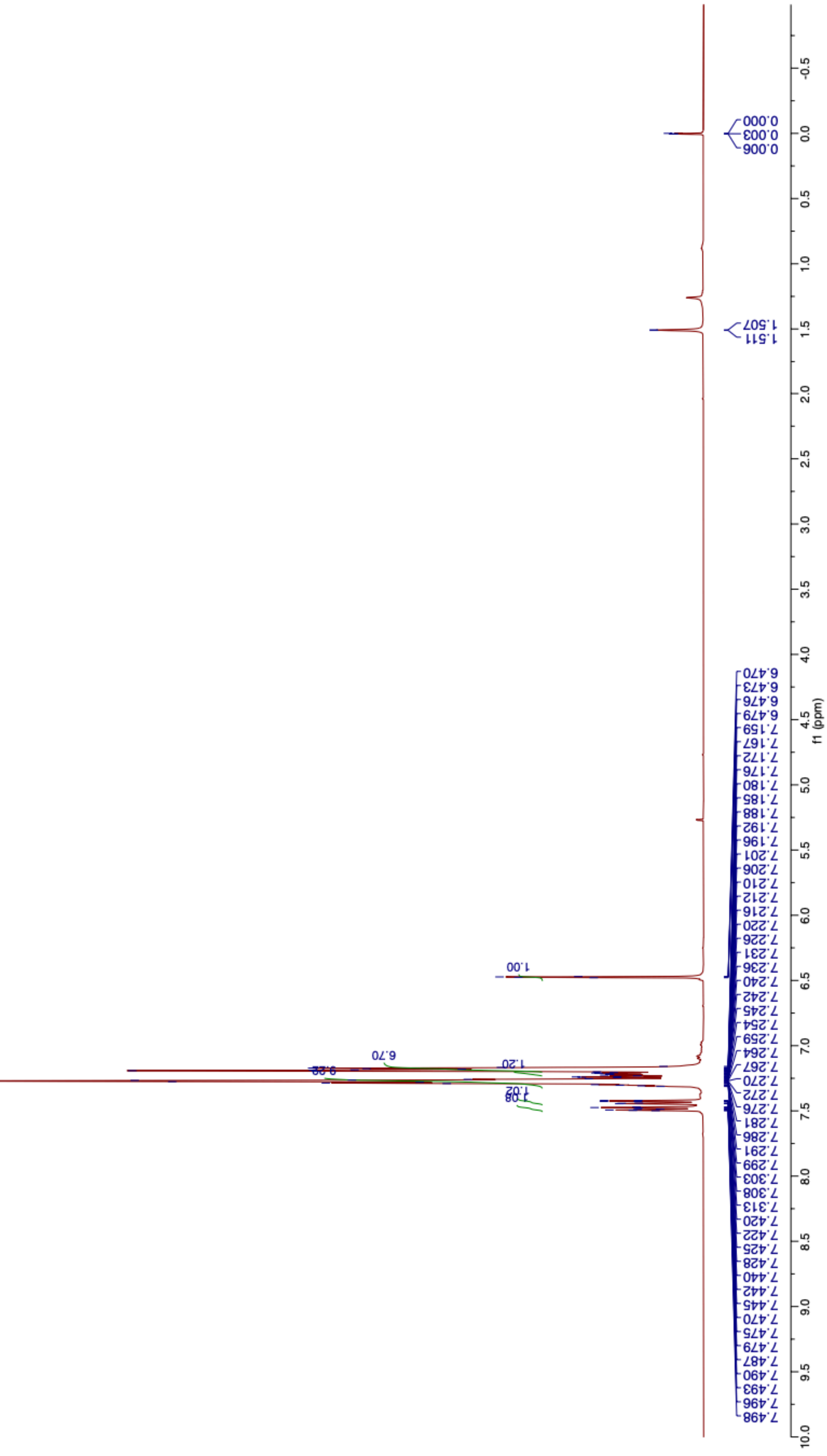

^{13}C NMR of **18** (101 MHz, $CDCl_3$)

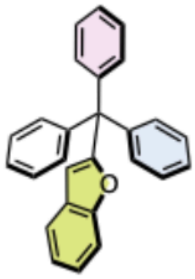

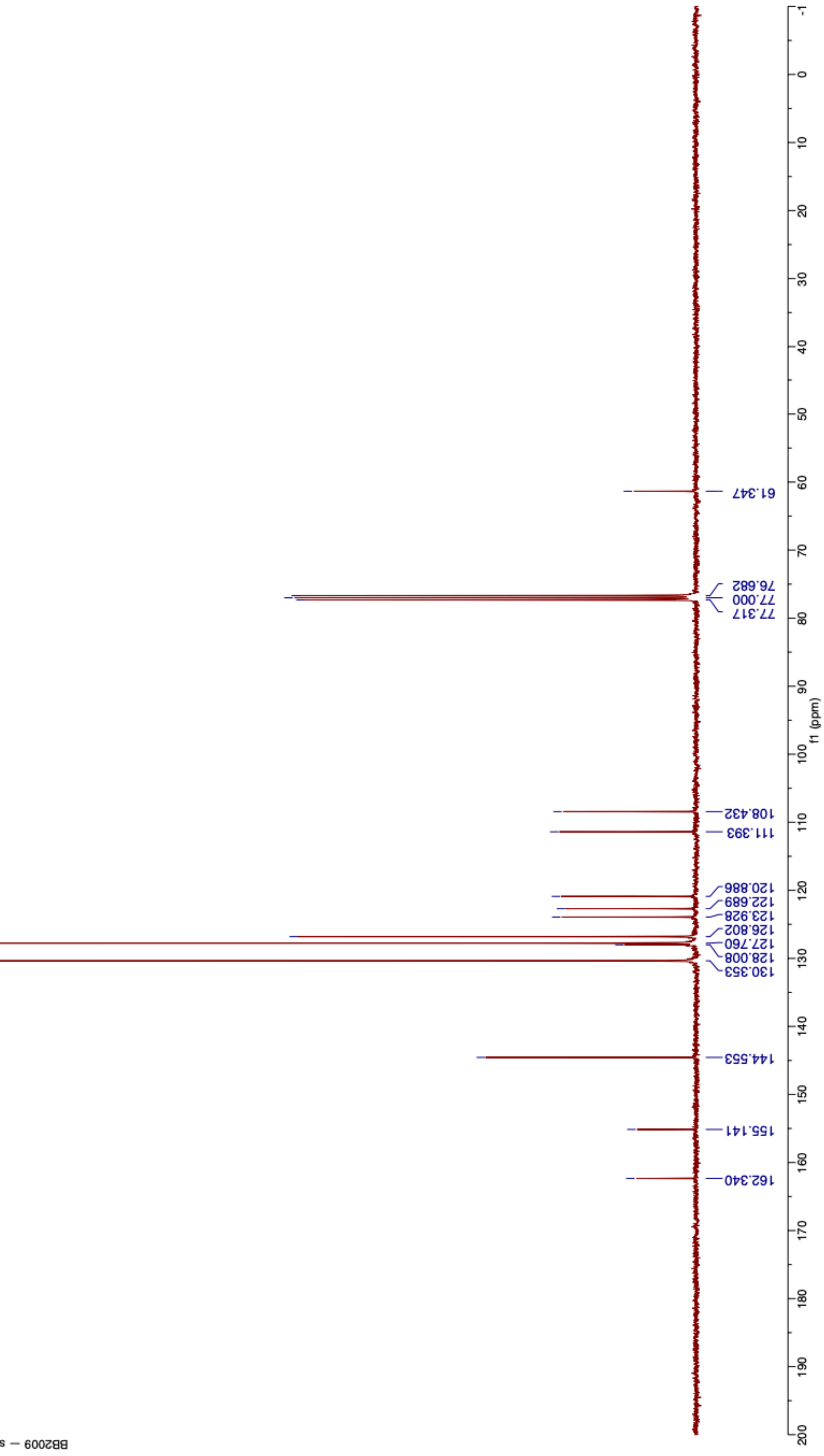

^{1}H NMR of **S3** (400 MHz, $CDCl_3$)

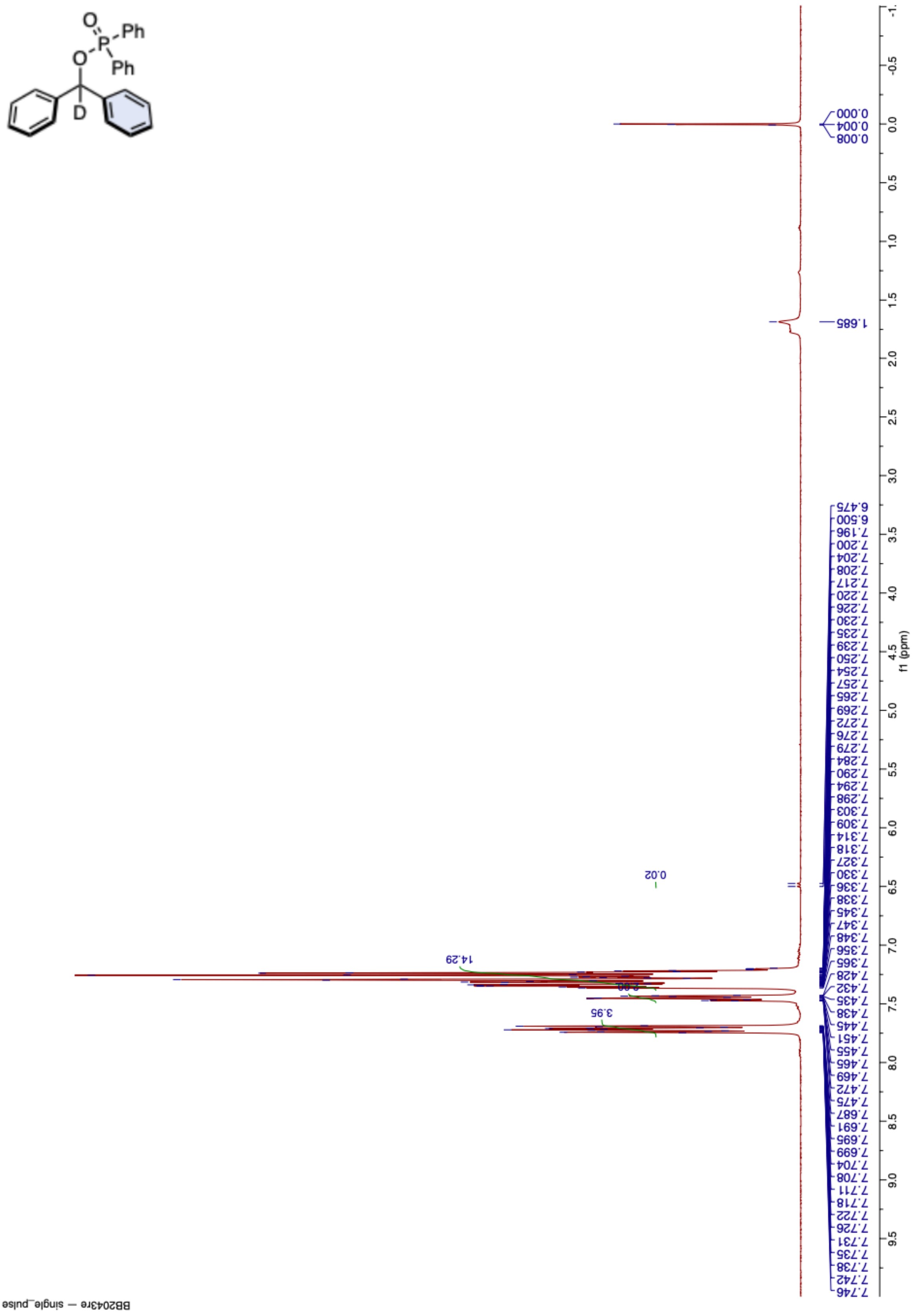

^{13}C NMR of **S3** (101 MHz, $CDCl_3$)

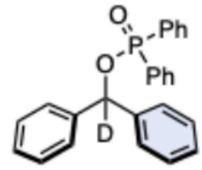

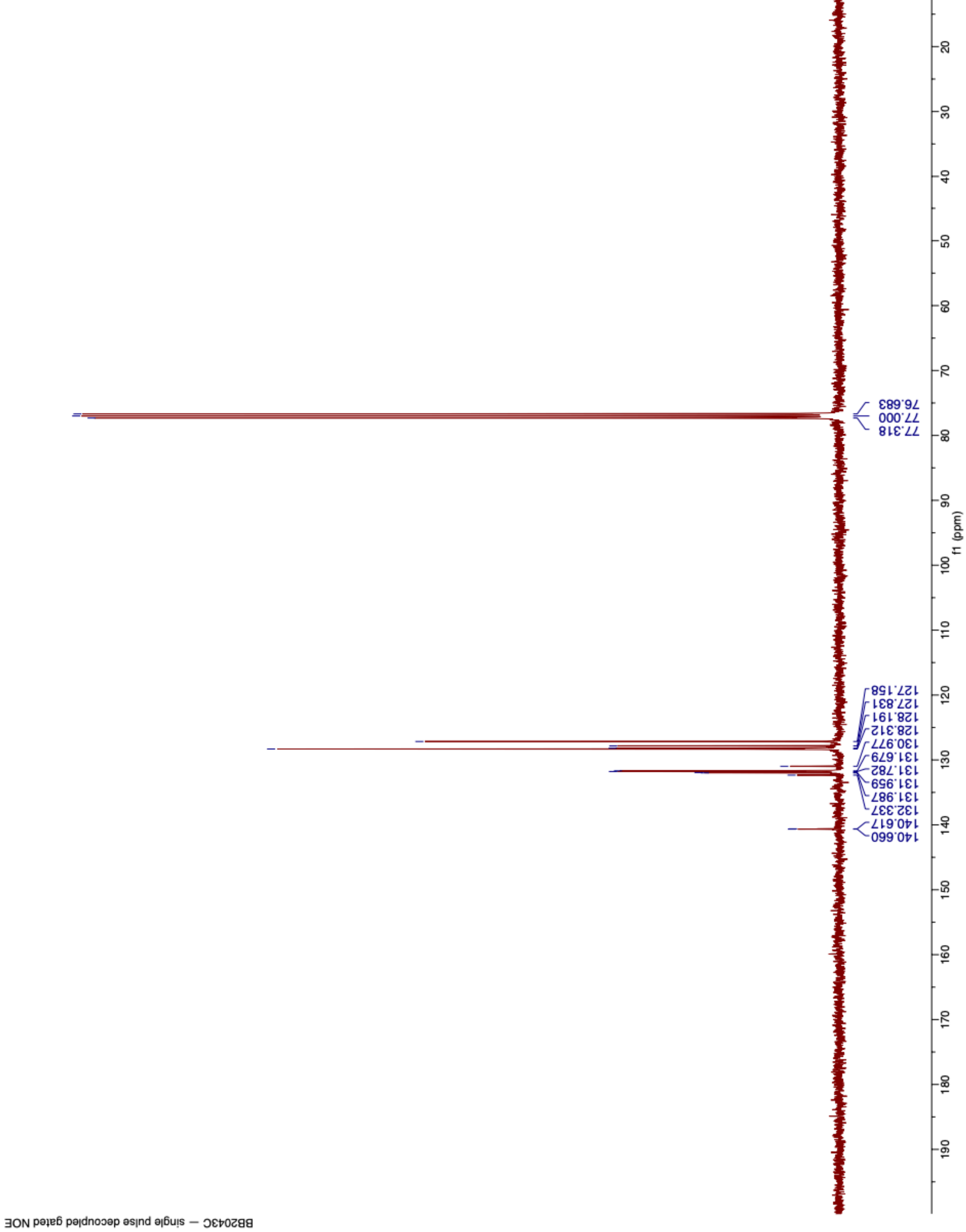

^{31}P NMR of **S3** (162 MHz, $CDCl_3$)

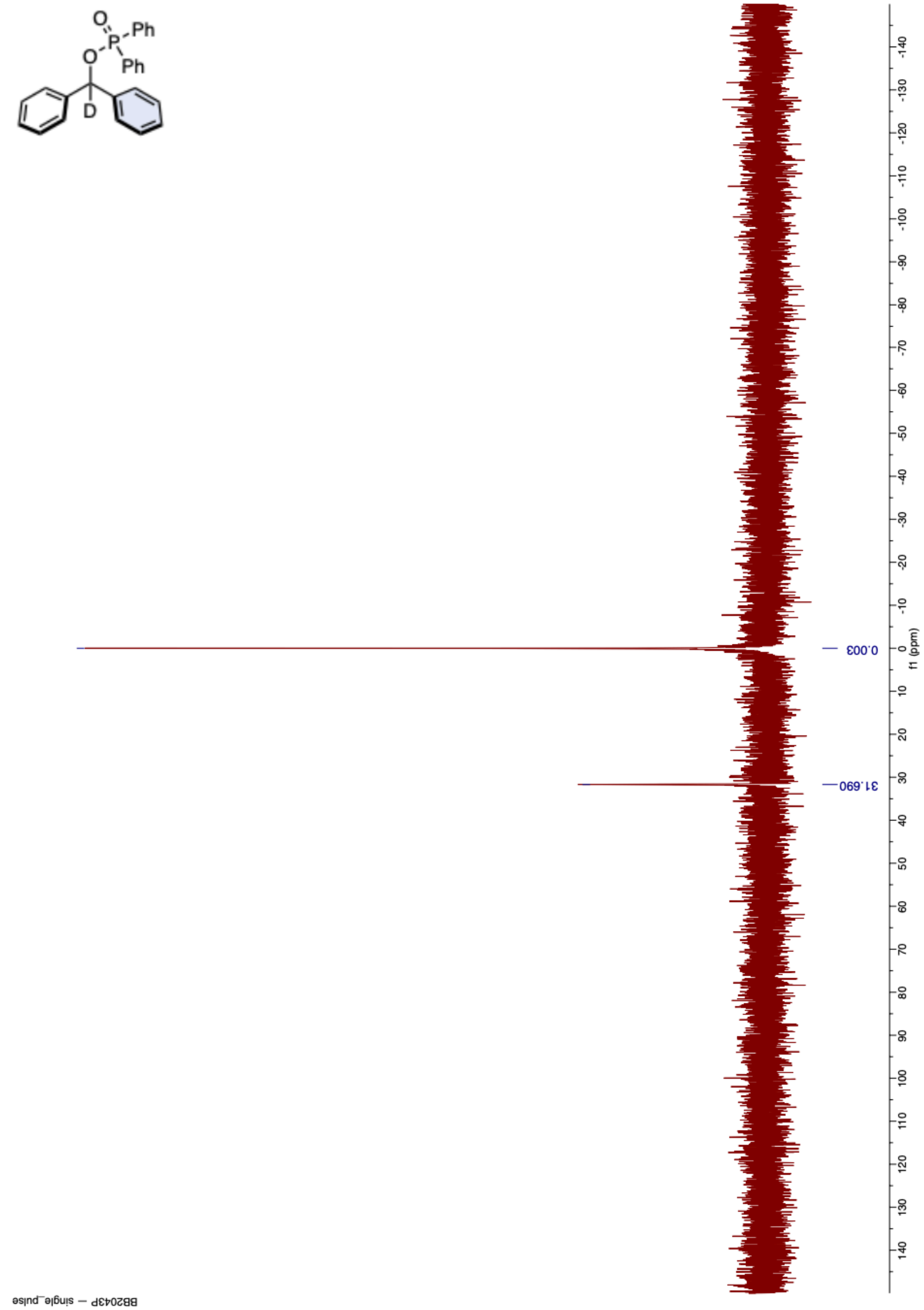

Supplement: SC-013-D2SC03720C-s001 [file SC-013-D2SC03720C-s001.pdf]
